# *Pseudomonas aeruginosa* urinary tract infections in hospitalized patients: Mortality and prognostic factors

**DOI:** 10.1371/journal.pone.0178178

**Published:** 2017-05-26

**Authors:** Jose Luis Lamas Ferreiro, Judith Álvarez Otero, Lucía González González, Luis Novoa Lamazares, Alexandra Arca Blanco, Jose Ramón Bermúdez Sanjurjo, Irene Rodríguez Conde, María Fernández Soneira, Javier de la Fuente Aguado

**Affiliations:** 1 Internal Medicine, Povisa Hospital, Vigo, Spain; 2 Microbiology, Povisa Hospital, Vigo, Spain; Universidade de Sao Paulo Faculdade de Medicina, BRAZIL

## Abstract

**Background:**

The aim of this study was to analyze the mortality and predictors of 30-day mortality among hospitalized patients with *Pseudomonas aeruginosa* urinary tract infection (PAUTI) and the impact of antibiotic treatment on survival.

**Methods:**

Patients admitted to our hospital with PAUTI or those diagnosed of PAUTI during hospitalization for other disease between September 2012 and September 2014 were included. Repeated episodes from the same patient were excluded. Database with demographic, clinical and laboratory ítems was created. Empirical and definitive antibiotic therapy, antimicrobial resistance and all-cause mortality at 30 and 90 days were included.

**Results:**

62 patients were included, with a mean age of 75 years. 51% were male. Mortality was 17.7% at 30 days and 33.9% at 90 days. Factors associated with reduced survival at 30 days were chronic liver disease with portal hypertension (P<0,01), diabetes mellitus (P = 0,04) chronic renal failure (P = 0,02), severe sepsis or septic shock (P<0,01), Charlson index > 3 (P = 0.02) and inadequated definitive antibiotic treatment (P<0,01). Independent risk factors for mortality in multivariate analysis were advanced chronic liver disease (HR 77,4; P<0,01), diabetes mellitus (HR 3,6; P = 0,04), chronic renal failure (HR 4,1; P = 0,03) and inadequated definitive antimicrobial treatment (HR 6,8; P = 0,01).

**Conclusions:**

PAUTI are associated with high mortality in hospitalized patients, which increases significantly in those with severe comorbidity such as chronic renal failure, advanced liver disease or diabetes mellitus. Inadequated antibiotic treatment is associated with poor outcome, which remarks the importance of adjusting empirical antibiotic treatment based on the microbiological susceptibility results.

## Introduction

Urinary tract infections are one of the most prevalent diseases in hospitalized patients, accounting for between 20 and 49% of all nosocomial infections [1, 2]. Within the hospital setting, 7–10% of urinary tract infections are caused by *Pseudomonas aeruginosa* (*P*. *aeruginosa*) [3, 4]. *P*. *aeruginosa* is a non-fermenter gram-negative bacilli with a large intrinsic resistance to multiple antibiotics. This characteristic, paired with its quick ability to acquire new antimicrobial resistance, makes this pathogen a growing problem in infectious disease pathology, especially when nosocomial in origen. There are no existing medical studies that explore possible factors associated with decreased survival in hospitalized patients with urinary tract infections by P. aeruginosa. Nor is the mortality of these patients known, except those associated with bacteremia, with an estimated mortality at 30 days from 5% to 33% [5–8]. Multiple factors have been associated with decreased survival in patients with bacteremia due to *P*. *aeruginosa*: age, low functional status, need for mechanical ventilation, central venous catheter, APACHE score or high Pitt score, inadequate antibiotic treatment, resistance to carbapenems, respiratory origin bacteremia, severe sepsis, shock, respiratory failure, polymicrobial infection, thrombocytopenia, bedsores, cirrhosis, chronic kidney disease, steroid use, cancer and AIDS [6–17]. Estimated comorbidity by the Charlson comorbidity index also shows an association between increased mortality in patients with chronic obstructive pulmonary disease (COPD) and respiratory infection by *P*. *aeruginosa*, as the BODE index similarly indicates [18]. The influence of an appropriate empiric treatment on the mortality of patients with *P*. *aeruginosa* infections remains controversial, with mixed results in various studies in patients with bacteremia [5, 6, 10, 11, 13, 16, 19, 20, 21]. Differences in the mortality impact of initial empiric antimicrobial treatment could be justified, in part, by the increased aggressiveness of infection depending on the origin of the bacteremia; making an effective empirical antimicrobial therapy in those with pneumonia or non biliary abdominal infections more relevant to good patient outcomes, however less impactful on urinary tract infections and infections of hepatobiliary origin.

The aim of this study was to determine mortality, and the factors associated with it, in hospitalized patients with urinary tract infections by *P*. *aeruginosa*, including the impact of antibiotic treatment on survival.

## Material and methods

This is an observational and retrospective study.

This study included all patients admitted to the hospital between September 1st, 2012 and September 1st, 2014 with urinary tract infections caused by *P*. *aeruginosa*, either as cause of admission or complication during hospitalization. In patients with repeated urinary tract infections caused by this bacteria, only the first episode was included. To identify patients with the aformentioned criterion, Microbiology´s service database of the hospital was used.

Repeated episodes in the same patient during the study period were excluded, regardless of recurrences by the same strain of *P*. *aeruginosa* or reinfection. Also, episodes of asymptomatic bacteriuria were omitted.

Both Speciality and Primary Care electronic medical records were reviewed and a database was created with the following variables that included:

- Demographic and anthropometric variables: age, sex, weight, height and BMI.
- Place of infection: Community, nosocomial (defined as occurring from the second day of admission or within 10 days after discharge) or health care-associated (defined as admission to the hospital in the previous 90 days, institutionalized patient, treatment in day hospital, dialysis or home hospitalization).
- Devices within 7 days prior to collection of urine culture: central and urinary catheter or gastrostomy
- Comorbidities: Hypertension, heart failure stage C (rated by ACC / AHA), heart disease of any etiology (including hypertensive, ischemic or valvular heart disease, heart failure, arrhythmias and cardiomyopathy), diabetes mellitus with or without target organ damage (neuropathy, nephropathy, retinopathy), estimated glomerular filtration rate calculated by the MDRD modified formula (categorizing the degree of renal failure as severe < 30 ml/min and moderate between 30–60 ml/min according to the KDOQI guidelines of the National Kidney Foundation), COPD confirmed by spirometry, peripheral arterial disease (Intermittent claudication, acute arterial ischemia, aortic aneurysm > 6 cm, peripheral by-pass), cerebrovascular disease (transient ischemic attack or ischemic stroke), cognitive impairment, solid tumor, leukemia or lymphoma, connective tissue disease, mild chronic liver disease (non portal hypertension) or severe with portal hypertension, AIDS, neutropenia (< 500 neutrophils at the time of withdrawal of urine culture), corticosteroids or immunosuppressive treatment. Charlson comorbidity index was calculated for each patient.
- Clinical and analytical features of each episode: measure of systolic and diastolic blood pressure, heart rate, temperature, serum leukocyte, hemoglobin, platelets, urea and creatinine in the urine culture collection day or in the nearest time. Presence of severe sepsis or septic shock criteria were evaluated.
- Microbiological features: polymicrobial or monomicrobial flora, bacteremia by the same strain of P. aeruginosa isolated in the urine culture and resistance pattern to differents antibiotics according to CMI criteria of CLSI. Strains with a pattern of antimicrobial multidrug resistance, those of which were insensitive to three or more antipseudomonal antibiotics were considered.
- Antibiotic treatment received by the patient during studied episode: adequate empirical antibiotic therapy (defined as treatment with one or more sensitive antimicrobials for one or many microorganisms involved depending of antibiogram), use of empirical antipseudomonal combination therapy, and adequacy of definitive treatment after available antibiogram.
- Clinical course of patients during the studied episode: all-cause mortality at 30 and 90 days

Dichotomous variables are expressed as numbers and percentages and are quantitative in mean and standard deviation.

Kaplan-Meier curves were used for the assessment of risk factors associated with mortality at 30 days, calculating the level of statistical significance using the log-rank test. Differences were considered statistically significant when P was less than 0.05.

The analysis of the factors independently associated with increased mortality was performed using Cox regression, including those variables associated with statistically significant reduced survival at 30 days by the log-rank test. SPSS 21 software was used for the statistical study.

The present study has been evaluated and approved by our institution (POVISA Hospital, registration number 160112-R1) and was carried out under the Guarantee of Confidentiality of the Information according to the Spanish Organic Law of Data Protection 15/99. All patient data were anonymized and deidentified prior to analysis.

## Results

62 urinary tract infections by *P*. *aeruginosa* in hospitalized patients were included (Table 1). The mean age was 75 +/- 12 years. 32 of 62 patients (51.6%) were male. The highest percentage of patients were admitted to hospital units in the medical area at the time of diagnosis [40/62 (64.5%)]. Most cases correspond to non-community origin infections [47/62 (75.8%)], being 43.5% nosocomial and 32.3% associated with health care. Most patients had associated comorbidity, most frequently: hypertension [34/62 (54.8%)], dyslipidemia [23/62 (37%)] and cognitive impairment [23/62 (37%)]. The average Charlson index was 3.6 +/- 2.5.

**Table 1:** Baseline characteristics of patients.

VARIABLE	N (%)
Age>65 years	50 (80,6%)
Male gender	32 (51,6%)
Nosocomial or nosohusial	47 (75,8%)
Hypertension	34 (54,8%)
Charlson index>3	30 (48,4%)
Dyslipidemia	23 (37,1%)
Cognitive impairment	23 (37,1%)
Diabetes mellitus	19 (30,6%)
Heart failure	15 (24,2%)
Immunosupresión	13 (21%)
Chronic renal failure	13 (21%)
Cancer	12 (19,4%)
Liver disease	8 (12,9%)

In 20 of 62 cases (32.3%) polymicrobial growth of flora in the urine culture was obtained, with the microorganisms most frequently associated being *Enterococcus faecalis* (14.5%), *Escherichia coli* (6.5%) and *Klebsiella pneumoniae* (3.2%). 5 of 62 cases (8%) were manifested as severe sepsis or septic shock. Only one patient had *P*. *aeruginosa* bacteremia (1.6%).

Regarding the type of antibiotic treatment administered, 15 of 62 patients (24,2%) received appropriate empirical antibiotic therapy, and only 3 patients (4.8%) were treated empirically with initial combination antipseudomonal therapy. 13 of 62 *P*. *aeruginosa* isolates (21%) presented an antimicrobial multidrug resistance pattern. Antipseudomonal antibiotics with less activity against *P*. *aeruginosa* strains isolated in our patients were aztreonam, levofloxacin, ciprofloxacin, and piperacillin/tazobactam, with 54%, 59%, 62% and 69% of sensitivity, respectively. The antibiotic with a better antipseudomonal profile was colistin (96% sensitivity). Final antibiotic treatment was inadequate in 15 of 62 cases (24,2%). Within this latter group, 6.7% of patients died before the antibiogram was available and 40% were discharged prior to microbiological report with a treatment regimen not corrected later.

**Fig 1:**
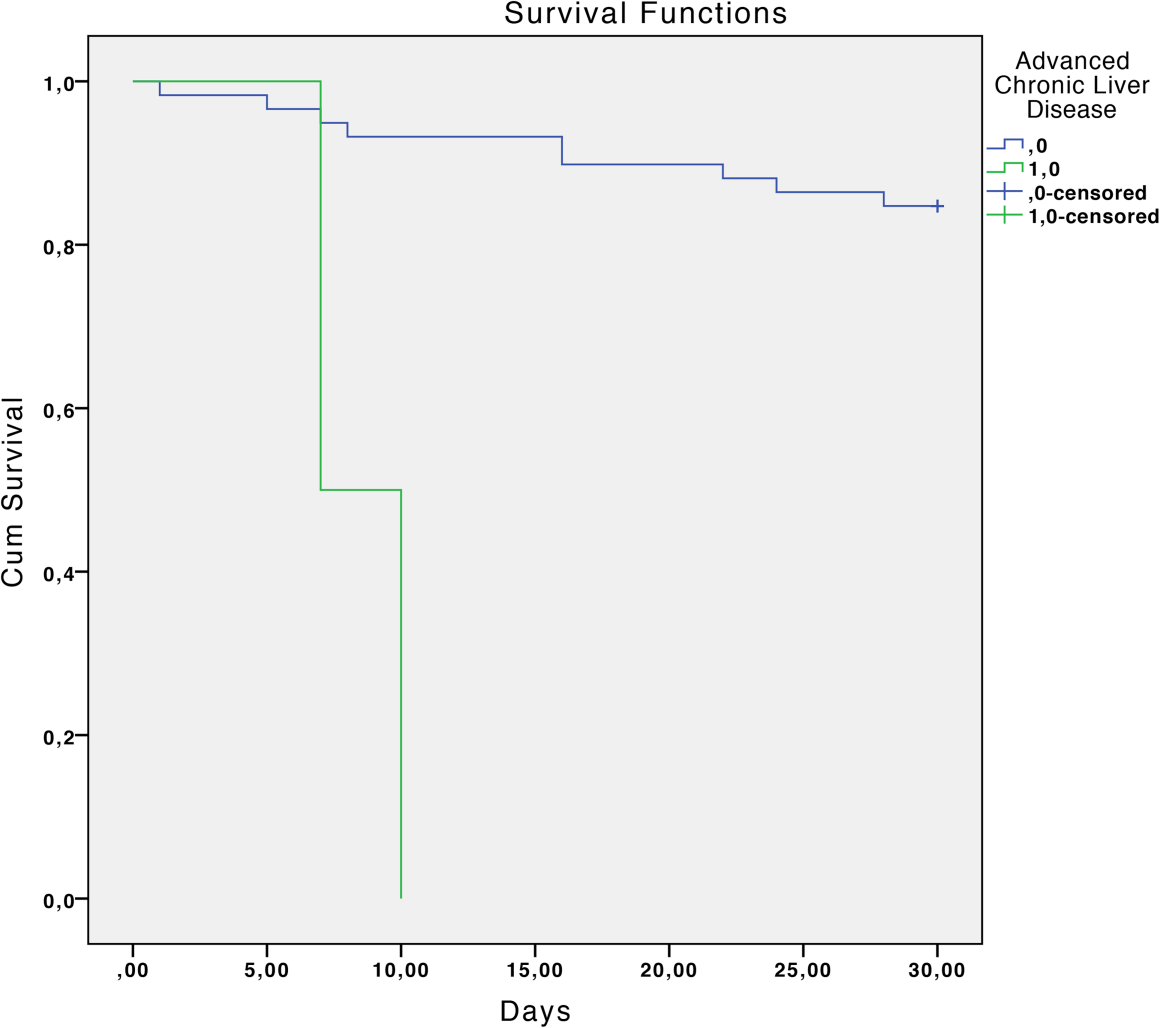
Kaplan-Meier 30-day survival curve according to advanced liver disease. P<0.01 (log-rank test).

**Fig 2:**
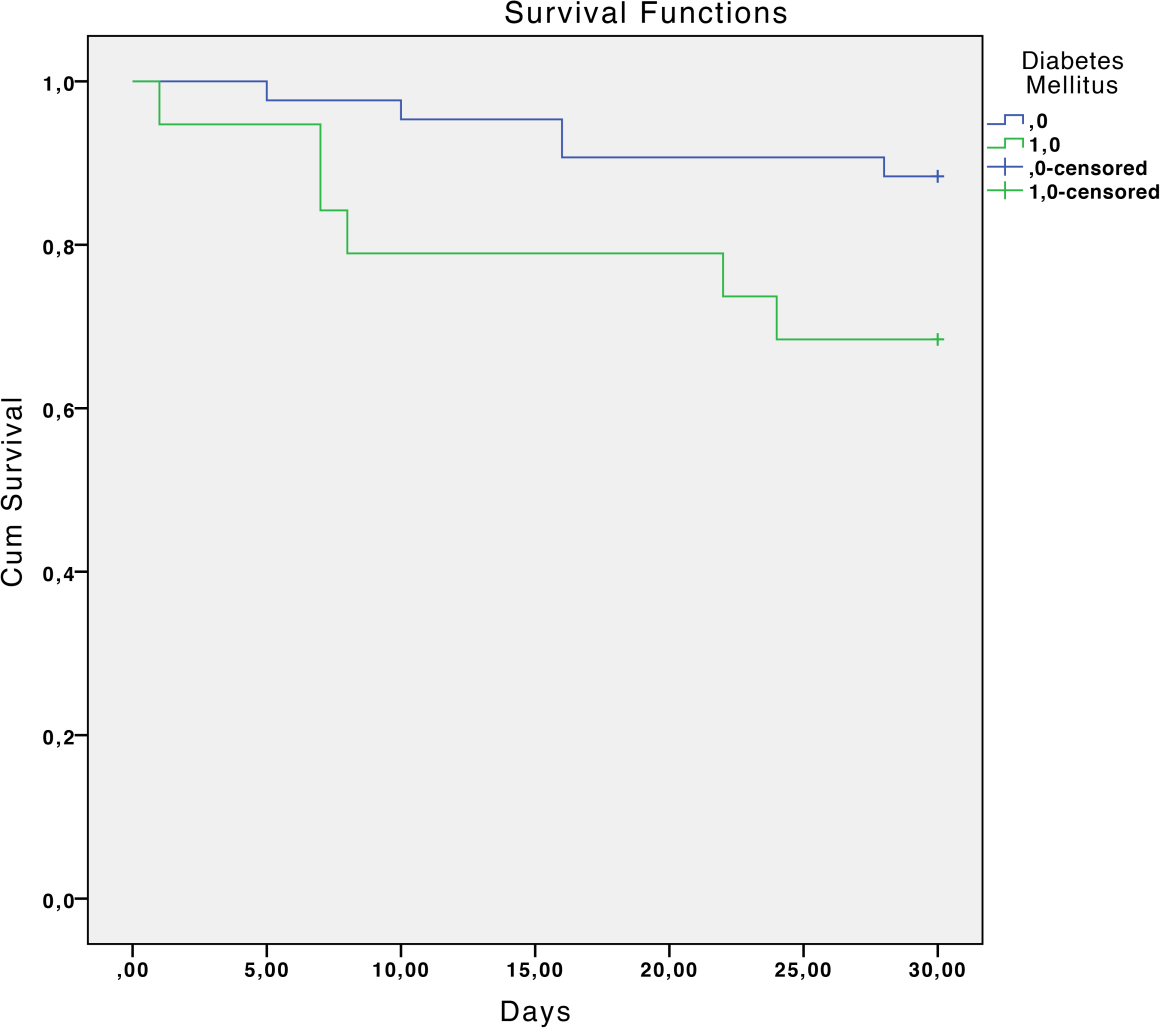
Kaplan-Meier 30-day survival curve according to diabetes mellitus. P = 0.04 (log-rank test).

**Fig 3:**
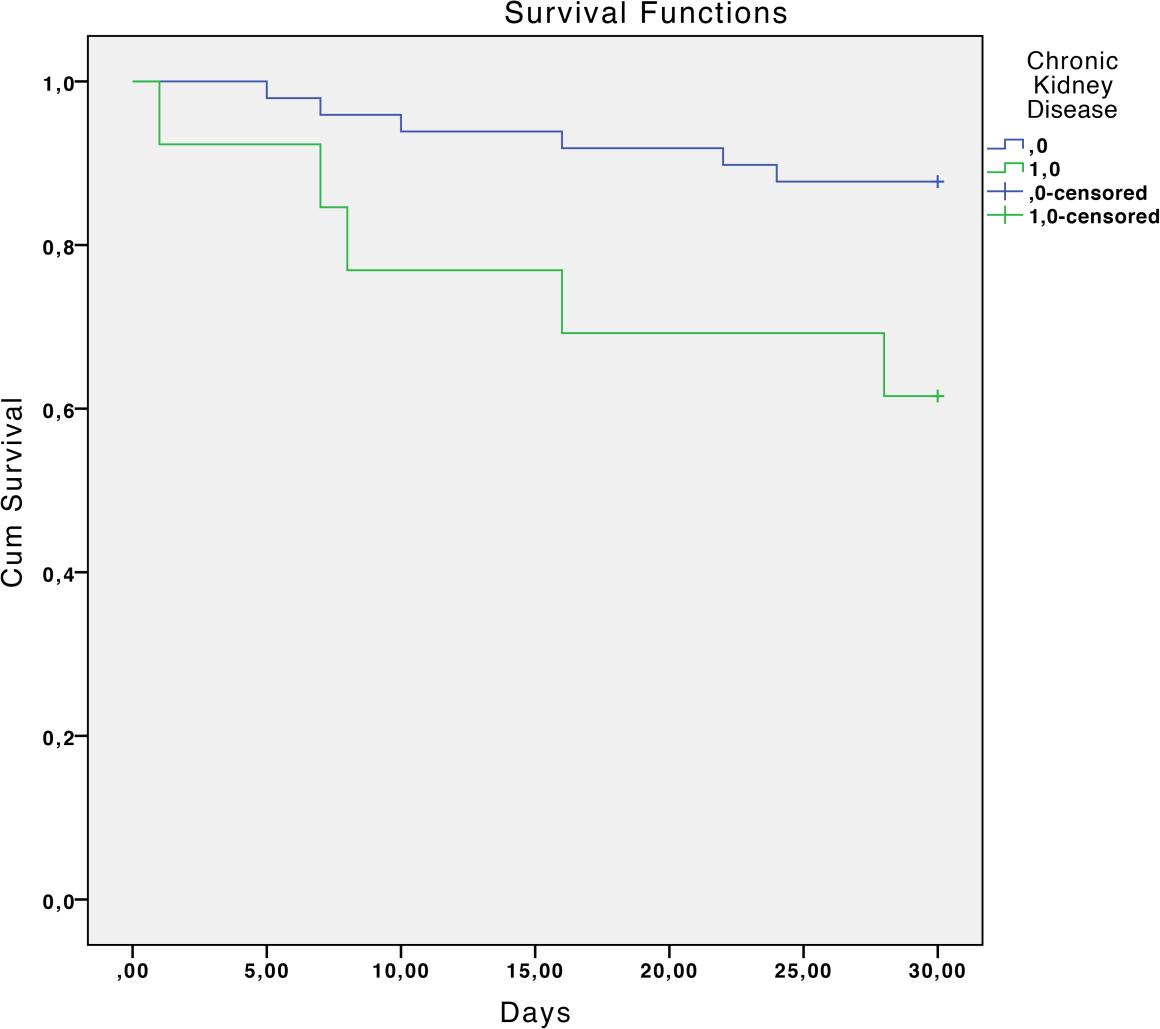
Kaplan-Meier 30-day survival curve according to chronic renal failure. P = 0.02 (log-rank test).

**Fig 4:**
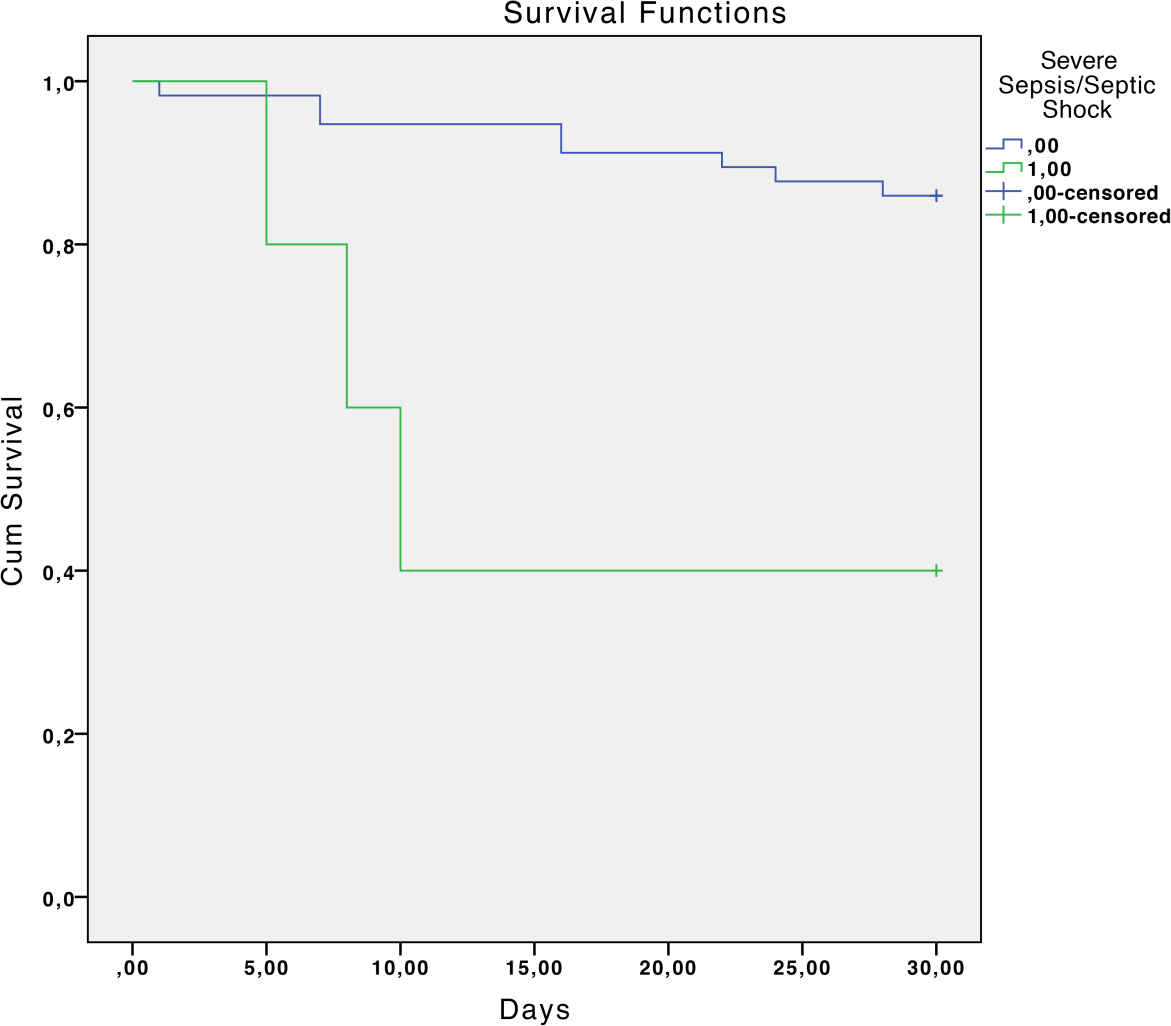
Kaplan-Meier 30-day survival curve according to severe sepsis or shock. P<0.01 (log-rank test).

**Fig 5:**
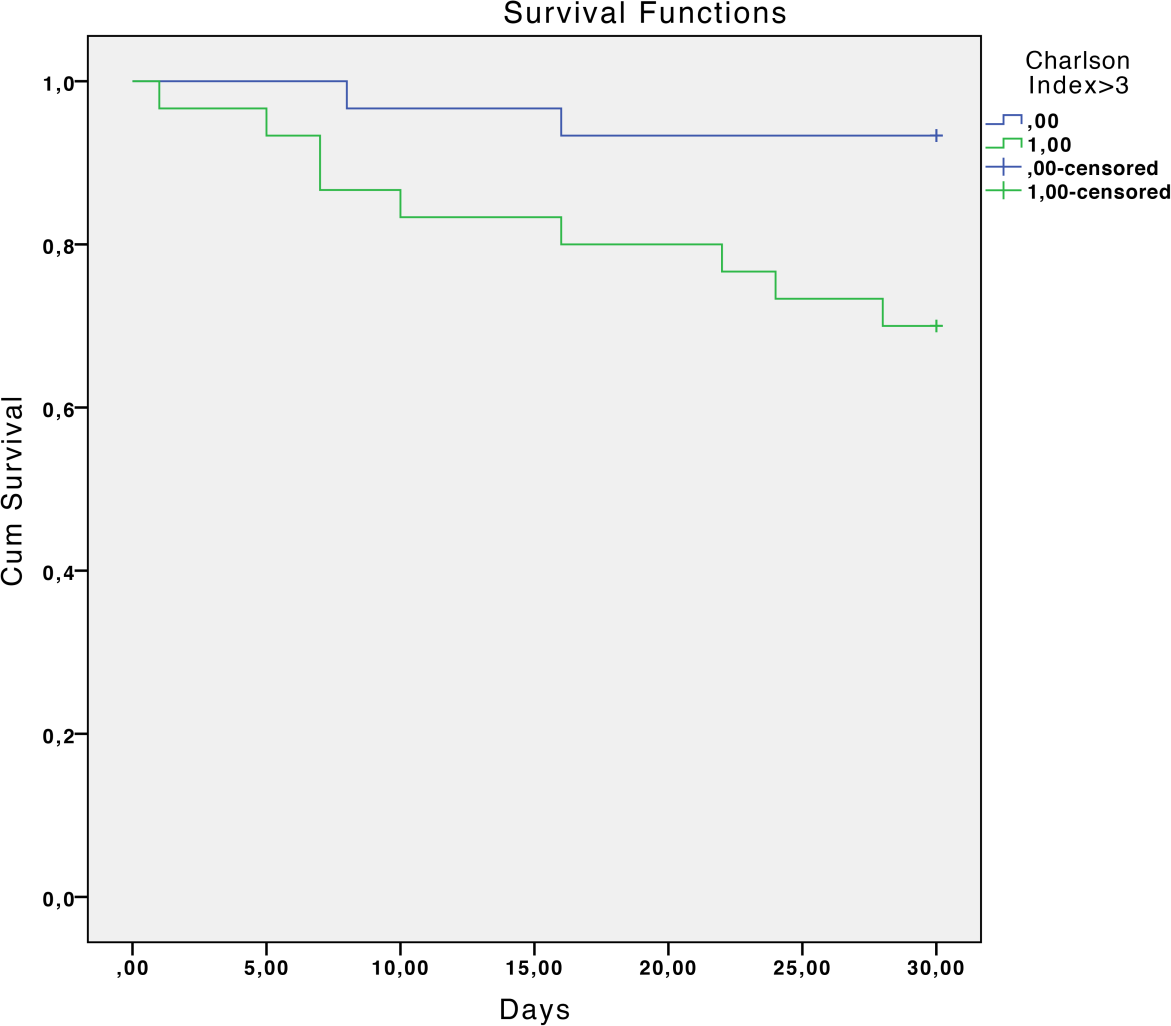
Kaplan-Meier 30-day survival curve according to Charlson index>3. P = 0.02 (log-rank test).

**Fig 6:**
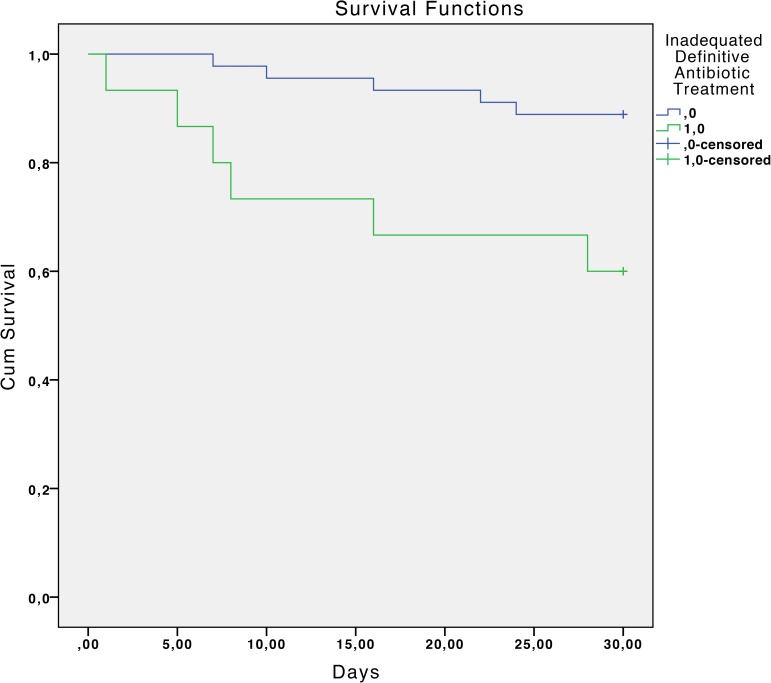
Kaplan-Meier 30-day survival curve according to inadequate antibiotic treatment. P<0.01 (log-rank test).

The overall mortality rate was 17.7% at 30 days and 33.9% at 90 days. Statistically significant associated factors of reduced survival at 30 days estimated by the Kaplan-Meier curves were: chronic liver disease with portal hypertension (P <0.01), diabetes mellitus (P = 0.04), chronic renal failure (P = 0.02), severe sepsis or septic shock (P <0.01), a Charlson index >3 (P = 0.02) and inadequate antibiotic treatment (P <0.01). (Table 2 and Figs 1–6).

**Table 2:** Mortality rates at 30 days.

VARIABLE		30-DAY MORTALITY	P (log-rank test)
**Age>65 years**	+	20%	0,3
	-	8,3%	
**Male gender**	+	18,7%	0,8
	-	16,7%	
**Nosocomial**	+	18,5%	0,8
	-	17,6%	
**Hypertension**	+	20,6%	0,5
	-	14,3%	
**Charlson index>3**	+	30%	<0,02
	-	6,7%	
**Dyslipidemia**	+	26,1%	0,1
	-	12,8%	
**Cognitive impairment**	+	26,1%	0,2
	-	12,8%	
**Diabetes mellitus**	+	31,6%	0,04
	-	11,6%	
**Heart failure**	+	33,3%	0,07
	-	12,8%	
**Immunosupresion**	+	30,8%	0,1
	-	14,3%	
**Chronic renal failure**	+	38,5%	0,02
	-	12,2%	
**Cancer**	+	25%	0,5
	-	16,3%	
**Advanced liver disease**	+	100%	<0,01
	-	15,3%	
**Inadequated empiric antibiotic treatment**	+	20%	0,9
	-	18,4%	
**Inadequated antibiotic treatment**	+	40%	<0,01
	-	11,1%	
**Multirresistant *P*. *aeruginosa***	+	23,5%	0,4
	-	15,6%	
**Severe sepsis or shock**	+	60%	0,01
	-	14%	

There were no differences in mortality depending on adequacy of empirical antimicrobial therapy, nor in the presence of multidrug resistance patterns in *P*. *aeruginosa* strains implicated in urinary tract infections. Although no deaths occurred within 30 days in the group treated with two antipseudomonal antibiotics, compared with 18% in the non-combination therapy group, the differences were not statistically significant.

In multivariate analysis, advanced chronic liver disease (P <0.01), diabetes mellitus (P = 0.04), chronic renal failure (P = 0.03), and definitive inadequate antibiotic treatment (P = 0.01) showed statistically significant association with increased mortality at 30 days (Table 3).

**Table 3:** Independent risk factors associated with mortality at 30 days (multivariate analysis made by Cox regression).

VARIABLE		30-DAY MORTALITY	P	HR (CI 95%)
**Advanced liver disease**	+	100%	<0,01	77,4(8,3–718,2)
	-	15,3%		
**Diabetes mellitus**	+	31,6%	0,04	3,6 (1,009–13,3)
	-	11,6%		
**Chronic renal failure**	+	38,5%	0,03	4,1 (1,07–16)
	-	12,2%		
**Inadequated antibiotic treatment**	+	40%	0,01	6,8 (1,5–29,5)
	-	11,1%		

## Discussion

To our knowledge, this study is the first in which the mortality of patients admitted with *P*. *aeruginosa* urinary tract infections is evaluated, regardless of the presence or absence of associated bacteremia, showing high mortality at 30 and 90 days of diagnosis (17.7% and 33.9% respectively). It is also the first scientific paper published until today in which risk factors associated with decreased survival in hospitalized patients with urinary tract infections are set for this microorganism.

As in previous studies, most conducted in patients with associated bacteremia, this study shows the prognostic role played by presence of comorbidity in these patients [6, 8, 14, 18], being independent risk factors for death at 30 days advanced liver disease, diabetes mellitus and chronic renal failure.

Referring to antibiotic treatment and its influence on the evolution, two important aspects must be emphasized. First, although there are many publications suggesting the contrary, from the point of view of empirical antibiotic therapy an increase in mortality in those patients for whom the empirical antimicrobial treatment was incorrect was not appreciated.

This could be explained by the absence of severity in the initial presentation of the infectious process in most of these patients, being the frequency of severe sepsis or septic shock of 8% at the onset, which has permitted a greater margin for the modification of treatment with no threat to the patient’s life. The association of two empirically antipseudomonal drugs also was not associated with improved survival, but the number of patients treated with combination empiric therapy was so small that this limits the validity of this conclusion.

Second, although the empirical antibiotic treatment has not demonstrated a relationship with mortality, when the final antimicrobial treatment was analyzed, it was observed that inadequate antibiotic therapy was an independent risk factor for mortality at 30 days. As in this study, Chamot et al analyzed in 2003 the antimicrobial treatment performed in 115 episodes of *P*. *aeruginosa* bacteremia associated with clinical features of systemic inflammatory response syndrome, and observed that in 19.4% of cases the final antibiotic treatment performed was inappropriate after susceptibility testing was available, with a mortality rate greater than 50% in the group of patients with inadequate treatment, being statistically significantly higher comparing to the group of patients treated properly [17]. In another study published the same year, Kim et al presented a set of 136 patients with *P*. *aeruginosa* bacteremia, of which 20% received inadequate definitive antimicrobial therapy, with 75% mortality rate at 30 days in those with inappropriate therapies (compared with mortality of 29% in those treated with appropriate antibiotics; P <0.001) [16]. Similar studies have been conducted in patients with infectious diseases caused by other microorganisms such as *Staphylococcus aureus*, *Enterobacteriaceae*, and etcetera; also with high rates of incorrect definitive antimicrobial treatment, demonstrating a significant impact on survival when that variable was assessed [22, 23].

Therefore, it is important to stress the importance of always adjusting the prescribed treatment according to the antibiogram, regardless of the initial evolution that the patient has presented. A clear example of this is that a high percentage of the inappropriately treated patients in this study were discharged before results of susceptibility testing were available, which is presumably a good initial clinical course in this group of patients despite the high percentage of deaths within 30 days of diagnosis.

When discussing the limitations of this study, it must first be emphasized that this is a retrospective analysis, which reduces the accuracy in collecting certain variables since all data are not available in some cases. So, among other things, because of this a detailed analysis of the underlying causes of death of patients in the specified period has become more difficult, making it difficult to establish in some cases if the death was due to an infectious process itself or other complications triggered by infection or not. Moreover, the number of cases is not large enough to obtain statistically significant results in any of the comparisons, such as combination or individual antipseudomonal treatment, or to make a stratified data analysis, which would be interesting, for example, for analysis of mortality related to inadequate empirical antibiotic treatment for those patients in whom the infection process was initially presented as severe sepsis or septic shock.

In summary, we can state that urinary tract infections by P. aeruginosa are associated with high mortality in hospitalized patients, which increases significantly in those with severe concomitant diseases such as chronic renal failure, advanced liver disease or diabetes mellitus. Appropriate antibiotic treatment of these infections significantly determines an optimal clinical evolution, so sensitivity testing and adjusting empirical antimicrobial therapy based on their outcome is essential. It is necessary to develop prospective studies with a larger number of patients to solve the many uncertainties that still exist in such a prevalent disease in hospitals.

## Supporting information

S1 File Descriptive statistics and frequency tables. (PDF)

S2 File Multivariate analysis (Cox regression). (PDF)

S3 File Survival analysis (Kaplan-Meier curves and log-rank test). (PDF)
