# Supplementary material for: Pseudomonas aeruginosa urinary tract infections in hospitalized patients: Mortality and prognostic factors
Source: PLoS One. 2017 May 26;12(5):e0178178. doi: 10.1371/journal.pone.0178178 (PMC5446154; doi:10.1371/journal.pone.0178178)
Supplement: S1 File — (PDF) [file pone.0178178.s001.pdf]

Frequency Tables and Descriptive Statistics

		Age	BMI	Days of Hospital Stay	Days of ICU Stay	Baseline Creatinine	Number of Previous Antibiotics	Number of Previous Antibiotic Families
N	Valid	62	40	62	62	62	62	62
	Lost	0	22	0	0	0	0	0
Mean		75,371	27,23	49,661	3,597	1,181	2,161	2,065
Median		78,500	25,50	15,500	,000	,900	2,000	2,000
Standard Deviation		12,1914	7,846	188,8042	9,8936	,8290	1,9350	1,5563
Minimum		44,0	14	1,0	,0	,3	,0	,0
Maximum		99,0	54	1486,0	41,0	3,8	9,0	6,0

		SBP	DBP	HR	Tª	LEUCOCITES	NEUTROPHILS	HB
N	Valid	59	59	54	61	61	40	9
	Lost	3	3	8	1	1	22	53
Mean		119,932	71,780	88,593	37,128	11393,279	8088,150	11,4444
Median		120,000	70,000	87,500	36,900	8720,000	6275,000	11,0000
Standard Deviation		23,9568	15,1488	22,0433	1,2553	6410,9359	6181,2438	1,58990
Minimum		79,0	44,0	40,0	35,0	3300,0	6,0	10,00
Maximum		180,0	110,0	144,0	39,5	32930,0	30300,0	15,00

		PLATELETS	CREATININE
N	Valid	61	60
	Lost	1	2
Mean		290213,115	1,618
Median		261000,000	,950
Standard Deviation		199594,515	1,4258
Minimum		95000,0	,3
Maximum		1573000,0	6,2

Enterococcus spp

		Frequency	Percent	Valid Percent	Cumulative Percent
Valid	,00	52	83,9	83,9	83,9
	1,00	10	16,1	16,1	100,0
	Total	62	100,0	100,0	

E. faecalis

		Frequency	Percent	Valid Percent	Cumulative Percent
Valid	,00	53	85,5	85,5	85,5
	1,00	9	14,5	14,5	100,0
	Total	62	100,0	100,0	

E. faecium

		Frequency	Percent	Valid Percent	Cumulative Percent
Valid	,00	61	98,4	98,4	98,4
	1,00	1	1,6	1,6	100,0
	Total	62	100,0	100,0	

S. aureus

		Frequency	Percent	Valid Percent	Cumulative Percent
Valid	,00	61	98,4	98,4	98,4
	1,00	1	1,6	1,6	100,0
	Total	62	100,0	100,0	

E. colli

		Frequency	Percent	Valid Percent	Cumulative Percent
Valid	,00	58	93,5	93,5	93,5
	1,00	4	6,5	6,5	100,0
	Total	62	100,0	100,0	

Klebsiella spp

		Frequency	Percent	Valid Percent	Cumulative Percent
Valid	,00	60	96,8	96,8	96,8
	1,00	2	3,2	3,2	100,0
	Total	62	100,0	100,0	

Citrobacter spp

		Frequency	Percent	Valid Percent	Cumulative Percent
Valid	,00	61	98,4	98,4	98,4
	1,00	1	1,6	1,6	100,0
	Total	62	100,0	100,0	

Morganella spp

		Frequency	Percent	Valid Percent	Cumulative Percent
Valid	,00	61	98,4	98,4	98,4
	1,00	1	1,6	1,6	100,0
	Total	62	100,0	100,0	

Enterobacter spp

		Frequency	Percent	Valid Percent	Cumulative Percent
Valid	,00	60	96,8	96,8	96,8
	1,00	2	3,2	3,2	100,0
	Total	62	100,0	100,0	

Candida spp

		Frequency	Percent	Valid Percent	Cumulative Percent
Valid	,00	61	98,4	98,4	98,4
	1,00	1	1,6	1,6	100,0
	Total	62	100,0	100,0	

Mortality_1_month

		Frequency	Percent	Valid Percent	Cumulative Percent
Valid	,00	51	82,3	82,3	82,3
	1,00	11	17,7	17,7	100,0
	Total	62	100,0	100,0	

Mortality_3_months

		Frequency	Percent	Valid Percent	Cumulative Percent
Valid	,00	41	66,1	66,1	66,1
	1,00	21	33,9	33,9	100,0
	Total	62	100,0	100,0	

Carbapenem Resistant

		Frequency	Percent	Valid Percent	Cumulative Percent
Valid	,0	50	80,6	80,6	80,6
	1,0	12	19,4	19,4	100,0
	Total	62	100,0	100,0	

Monomicrobian Culture

		Frequency	Percent	Valid Percent	Cumulative Percent
Valid	,00	20	32,3	32,3	32,3
	1,00	42	67,7	67,7	100,0
	Total	62	100,0	100,0	

Adequated Empirical Antibiotic Treatment

		Frequency	Percent	Valid Percent	Cumulative Percent
Valid	,0	38	61,3	71,7	71,7
	1,0	15	24,2	28,3	100,0
	Total	53	85,5	100,0	
Missing	System	9	14,5		
Total		62	100,0		

Female Sex

		Frequency	Percent	Valid Percent	Cumulative Percent
Valid	0	32	51,6	51,6	51,6
	1	30	48,4	48,4	100,0
	Total	62	100,0	100,0	

Malnutrition

		Frequency	Percent	Valid Percent	Cumulative Percent
Valid	,0	43	69,4	78,2	78,2
	1,0	12	19,4	21,8	100,0
	Total	55	88,7	100,0	
Missing	System	7	11,3		
Total		62	100,0		

Obesity

		Frequency	Percent	Valid Percent	Cumulative Percent
Valid	,0	39	62,9	72,2	72,2
	1,0	15	24,2	27,8	100,0
	Total	54	87,1	100,0	
Missing	System	8	12,9		
Total		62	100,0		

Intensive Care Unit

		Frequency	Percent	Valid Percent	Cumulative Percent
Valid	,0	48	77,4	77,4	77,4
	1,0	14	22,6	22,6	100,0
	Total	62	100,0	100,0	

NOSOCOMIAL

		Frequency	Percent	Valid Percent	Cumulative Percent
Valid	,0	34	54,8	55,7	55,7
	1,0	27	43,5	44,3	100,0
	Total	61	98,4	100,0	
Missing	System	1	1,6		
Total		62	100,0		

Health Care Associated

		Frequency	Percent	Valid Percent	Cumulative Percent
Valid	,0	41	66,1	67,2	67,2
	1,0	20	32,3	32,8	100,0
	Total	61	98,4	100,0	
Missing	System	1	1,6		
Total		62	100,0		

Institucionalized

		Frequency	Percent	Valid Percent	Cumulative Percent
Valid	,0	57	91,9	95,0	95,0
	1,0	3	4,8	5,0	100,0
	Total	60	96,8	100,0	
Missing	System	2	3,2		
Total		62	100,0		

Central Venous Catheter

		Frequency	Percent	Valid Percent	Cumulative Percent
Valid	,0	55	88,7	88,7	88,7
	1,0	7	11,3	11,3	100,0
	Total	62	100,0	100,0	

Mechanical Ventilation

		Frequency	Percent	Valid Percent	Cumulative Percent
Valid	,0	57	91,9	91,9	91,9
	1,0	5	8,1	8,1	100,0
	Total	62	100,0	100,0	

Urinary Catheter

		Frequency	Percent	Valid Percent	Cumulative Percent
Valid	,0	19	30,6	30,6	30,6
	1,0	43	69,4	69,4	100,0
	Total	62	100,0	100,0	

Gastrostomy

		Frequency	Percent	Valid Percent	Cumulative Percent
Valid	,0	56	90,3	90,3	90,3
	1,0	6	9,7	9,7	100,0
	Total	62	100,0	100,0	

Cognitive Impairment

		Frequency	Percent	Valid Percent	Cumulative Percent
Valid	,0	39	62,9	62,9	62,9
	1,0	23	37,1	37,1	100,0
	Total	62	100,0	100,0	

Solid Organ Cancer

		Frequency	Percent	Valid Percent	Cumulative Percent
Valid	,0	48	77,4	77,4	77,4
	1,0	14	22,6	22,6	100,0
	Total	62	100,0	100,0	

Leukemia

		Frequency	Percent	Valid Percent	Cumulative Percent
Valid	,0	61	98,4	98,4	98,4
	1,0	1	1,6	1,6	100,0
	Total	62	100,0	100,0	

Liver Disease

		Frequency	Percent	Valid Percent	Cumulative Percent
Valid	,0	54	87,1	87,1	87,1
	1,0	8	12,9	12,9	100,0
	Total	62	100,0	100,0	

Lymphoma

		Frequency	Percent	Valid Percent	Cumulative Percent
Valid	,0	62	100,0	100,0	100,0

Metastasic Cancer

		Frequency	Percent	Valid Percent	Cumulative Percent
Valid	,0	58	93,5	93,5	93,5
	1,0	4	6,5	6,5	100,0
	Total	62	100,0	100,0	

Conective Tissue Disease

		Frequency	Percent	Valid Percent	Cumulative Percent
Valid	,0	60	96,8	96,8	96,8
	1,0	2	3,2	3,2	100,0
	Total	62	100,0	100,0	

Advanced Chronic Liver Disease

		Frequency	Percent	Valid Percent	Cumulative Percent
Valid	,0	59	95,2	96,7	96,7
	1,0	2	3,2	3,3	100,0
	Total	61	98,4	100,0	
Missing	System	1	1,6		
Total		62	100,0		

Diabetes Mellitus

		Frequency	Percent	Valid Percent	Cumulative Percent
Valid	,0	43	69,4	69,4	69,4
	1,0	19	30,6	30,6	100,0
	Total	62	100,0	100,0	

Diabetes Mellitus with Target Organ Damage

		Frequency	Percent	Valid Percent	Cumulative Percent
Valid	,0	55	88,7	88,7	88,7
	1,0	7	11,3	11,3	100,0
	Total	62	100,0	100,0	

Hypertension

		Frequency	Percent	Valid Percent	Cumulative Percent
Valid	,0	28	45,2	45,2	45,2
	1,0	34	54,8	54,8	100,0
	Total	62	100,0	100,0	

Dyslipidemia

		Frequency	Percent	Valid Percent	Cumulative Percent
Valid	,0	39	62,9	62,9	62,9
	1,0	23	37,1	37,1	100,0
	Total	62	100,0	100,0	

Respiratory Disease

		Frequency	Percent	Valid Percent	Cumulative Percent
Valid	,0	48	77,4	77,4	77,4
	1,0	14	22,6	22,6	100,0
	Total	62	100,0	100,0	

COPD

		Frequency	Percent	Valid Percent	Cumulative Percent
Valid	,0	54	87,1	87,1	87,1
	1,0	8	12,9	12,9	100,0
	Total	62	100,0	100,0	

Heart Disease

		Frequency	Percent	Valid Percent	Cumulative Percent
Valid	,0	34	54,8	54,8	54,8
	1,0	28	45,2	45,2	100,0
	Total	62	100,0	100,0	

Heart Failure

		Frequency	Percent	Valid Percent	Cumulative Percent
Valid	,0	47	75,8	75,8	75,8
	1,0	15	24,2	24,2	100,0
	Total	62	100,0	100,0	

Myocardial Infarction

		Frequency	Percent	Valid Percent	Cumulative Percent
Valid	,0	52	83,9	83,9	83,9
	1,0	10	16,1	16,1	100,0
	Total	62	100,0	100,0	

Peripheral Arterial Disease

		Frequency	Percent	Valid Percent	Cumulative Percent
Valid	,0	51	82,3	82,3	82,3
	1,0	11	17,7	17,7	100,0
	Total	62	100,0	100,0	

Cerebrovascular Disease

		Frequency	Percent	Valid Percent	Cumulative Percent
Valid	,0	44	71,0	71,0	71,0
	1,0	18	29,0	29,0	100,0
	Total	62	100,0	100,0	

Hemiplegia

		Frequency	Percent	Valid Percent	Cumulative Percent
Valid	,0	55	88,7	88,7	88,7
	1,0	7	11,3	11,3	100,0
	Total	62	100,0	100,0	

Ulcus

		Frequency	Percent	Valid Percent	Cumulative Percent
Valid	,0	54	87,1	87,1	87,1
	1,0	8	12,9	12,9	100,0
	Total	62	100,0	100,0	

Chronic Kidney Disease (Moderate/Severe)

		Frequency	Percent	Valid Percent	Cumulative Percent
Valid	,0	53	85,5	85,5	85,5
	1,0	9	14,5	14,5	100,0
	Total	62	100,0	100,0	

Chronic Kidney Disease

		Frequency	Percent	Valid Percent	Cumulative Percent
Valid	,0	49	79,0	79,0	79,0
	1,0	13	21,0	21,0	100,0
	Total	62	100,0	100,0	

Dialysis

		Frequency	Percent	Valid Percent	Cumulative Percent
Valid	,0	62	100,0	100,0	100,0

HIV

		Frequency	Percent	Valid Percent	Cumulative Percent
Valid	,0	62	100,0	100,0	100,0

AIDS

		Frequency	Percent	Valid Percent	Cumulative Percent
Valid	,0	62	100,0	100,0	100,0

Immunosupression (Neutropenia/Solid Organ Transplant/Inmunosupressive Treatment)

		Frequency	Percent	Valid Percent	Cumulative Percent
Valid	,00	49	79,0	79,0	79,0
	1,00	13	21,0	21,0	100,0
	Total	62	100,0	100,0	

Previous 30 Days Surgery

		Frequency	Percent	Valid Percent	Cumulative Percent
Valid	,0	46	74,2	76,7	76,7
	1,0	14	22,6	23,3	100,0
	Total	60	96,8	100,0	
Missing	System	2	3,2		
Total		62	100,0		

Antibiotic Treatment in Previous 90 Days

		Frequency	Percent	Valid Percent	Cumulative Percent
Valid	,0	9	14,5	15,3	15,3
	1,0	50	80,6	84,7	100,0
	Total	59	95,2	100,0	
Missing	System	3	4,8		
Total		62	100,0		

Hypotension

		Frequency	Percent	Valid Percent	Cumulative Percent
Valid	,0	50	80,6	82,0	82,0
	1,0	11	17,7	18,0	100,0
	Total	61	98,4	100,0	
Missing	System	1	1,6		
Total		62	100,0		

Tachycardia

		Frequency	Percent	Valid Percent	Cumulative Percent
Valid	,0	41	66,1	74,5	74,5
	1,0	14	22,6	25,5	100,0
	Total	55	88,7	100,0	
Missing	System	7	11,3		
Total		62	100,0		

Fever

		Frequency	Percent	Valid Percent	Cumulative Percent
Valid	,0	36	58,1	58,1	58,1
	1,0	26	41,9	41,9	100,0
	Total	62	100,0	100,0	

Empirical Antipseudomonic Treatment

		Frequency	Percent	Valid Percent	Cumulative Percent
Valid	,00	38	61,3	61,3	61,3
	1,00	24	38,7	38,7	100,0
	Total	62	100,0	100,0	

Empirical Antipseudomonic Bitherapy

		Frequency	Percent	Valid Percent	Cumulative Percent
Valid	,00	59	95,2	95,2	95,2
	1,00	3	4,8	4,8	100,0
	Total	62	100,0	100,0	

Antibiotic Bitherapy

		Frequency	Percent	Valid Percent	Cumulative Percent
Valid	,00	48	77,4	77,4	77,4
	1,00	14	22,6	22,6	100,0
	Total	62	100,0	100,0	

Multiressistant Pseudomonas

		Frequency	Percent	Valid Percent	Cumulative Percent
Valid	,0	44	71,0	77,2	77,2
	1,0	13	21,0	22,8	100,0
	Total	57	91,9	100,0	
Missing	System	5	8,1		
Total		62	100,0		

Charlson Index>2

		Frequency	Percent	Valid Percent	Cumulative Percent
Valid	,00	23	37,1	38,3	38,3
	1,00	37	59,7	61,7	100,0
	Total	60	96,8	100,0	
Missing	System	2	3,2		
Total		62	100,0		

Nosocomial and Healthcare Associated

		Frequency	Percent	Valid Percent	Cumulative Percent
Valid	,00	14	22,6	23,3	23,3
	1,00	46	74,2	76,7	100,0
	Total	60	96,8	100,0	
Missing	System	2	3,2		
Total		62	100,0		

Age>65 years

		Frequency	Percent	Valid Percent	Cumulative Percent
Valid	,00	12	19,4	19,4	19,4
	1,00	50	80,6	80,6	100,0
	Total	62	100,0	100,0	

Age>75 years

		Frequency	Percent	Valid Percent	Cumulative Percent
Valid	,00	26	41,9	41,9	41,9
	1,00	36	58,1	58,1	100,0
	Total	62	100,0	100,0	

Severe Sepsis/Septic Shock

		Frequency	Percent	Valid Percent	Cumulative Percent
Valid	,00	57	91,9	91,9	91,9
	1,00	5	8,1	8,1	100,0
	Total	62	100,0	100,0	

Chronic Kidney Disease (mild)

		Frequency	Percent	Valid Percent	Cumulative Percent
Valid	,00	52	83,9	83,9	83,9
	1,00	10	16,1	16,1	100,0
	Total	62	100,0	100,0	

Charlson Index>3

		Frequency	Percent	Valid Percent	Cumulative Percent
Valid	,00	30	48,4	50,0	50,0
	1,00	30	48,4	50,0	100,0
	Total	60	96,8	100,0	
Missing	System	2	3,2		
Total		62	100,0		

Inadequated Definitive Antibiotic Treatment

		Frequency	Percent	Valid Percent	Cumulative Percent
Valid	,0	45	72,6	75,0	75,0
	1,0	15	24,2	25,0	100,0
	Total	60	96,8	100,0	
Missing	System	2	3,2		
Total		62	100,0		

NOTE: 0 means no, 1 means yes in the frecuency tables
