# Supplementary material for: Pseudomonas aeruginosa urinary tract infections in hospitalized patients: Mortality and prognostic factors
Source: PLoS One. 2017 May 26;12(5):e0178178. doi: 10.1371/journal.pone.0178178 (PMC5446154; doi:10.1371/journal.pone.0178178)
Supplement: S2 File — (PDF) [file pone.0178178.s002.pdf]

MULTIVARIATE ANALYSIS (COX REGRESSION)

Case Processing Summary

		N	Percent
Cases available in analysis	Event[a]	11	17,7%
	Censored	47	75,8%
	Total	58	93,5%
Cases dropped	Cases with missing values	4	6,5%
	Cases with negative time	0	0,0%
	Censored cases before the earliest event in a stratum	0	0,0%
	Total	4	6,5%
Total		62	100,0%

a. Dependent Variable: Supervivencia_días_primer_mes

Block 0: Beginning Block

Variables not in the Equation[a]

	Score	df	Sig.
Advanced Chronic Liver Disease	19,212	1	,000
Diabetes Mellitus	3,813	1	,051
Chronic Kidney Disease	6,991	1	,008
Inadequated Definitive Antibiotic Treatment	6,723	1	,010
Severe Sepsis/Septic Shock	9,154	1	,002
Charlson Index>3	6,027	1	,014

a. Residual Chi Square = 40,067 with 6 df Sig. = ,000

Block 1: Method = Forward Stepwise (Wald)

Omnibus Tests of Model Coefficients[e]

Step	-2 Log Likelihood	Overall (score)			Change From Previous Step			Change From Previous …	
		Chi-square	df	Sig.	Chi-square	df	Sig.	Chi-square	df
1[a]	80,673	19,212	1	,000	6,709	1	,010	6,709	1
2[b]	73,334	27,746	2	,000	7,340	1	,007	14,048	2
3[c]	68,451	33,021	3	,000	4,882	1	,027	18,931	3
4[d]	64,545	35,488	4	,000	3,906	1	,048	22,837	4

Omnibus Tests of Model Coefficients[e]

Step	Change …
	Sig.
1[a]	,010
2[b]	,001
3[c]	,000
4[d]	,000

a. Variable(s) Entered at Step Number 1: Advanced Chronic Liver Disease

b. Variable(s) Entered at Step Number 2: Chronic Kidney Disease

c. Variable(s) Entered at Step Number 3: Inadequated Definitive Antibiotic Treatment

d. Variable(s) Entered at Step Number 4: Diabetes Mellitus

e. Beginning Block Number 1. Method = Forward Stepwise (Wald)

Variables in the Equation

		B	S.E.	Wald	df	Sig.	Exp(B)	95,0% CI	
								Lower	Upper
Step 1	Advanced Chronic Liver Disease	2,817	,874	10,377	1	,001	16,720	3,013	92,791
Step 2	Advanced Chronic Liver Disease	3,510	,958	13,412	1	,000	33,453	5,112	218,924
	Chronic Kidney Disease	1,882	,672	7,843	1	,005	6,569	1,759	24,527
Step 3	Advanced Chronic Liver Disease	4,082	1,039	15,431	1	,000	59,246	7,730	454,075
	Chronic Kidney Disease	1,424	,703	4,104	1	,043	4,154	1,047	16,471
	Inadequated Definitive Antibiotic Treatment	1,578	,740	4,544	1	,033	4,846	1,136	20,678
Step 4	Advanced Chronic Liver Disease	4,349	1,137	14,642	1	,000	77,410	8,343	718,216
	Diabetes Mellitus	1,300	,659	3,893	1	,048	3,669	1,009	13,345
	Chronic Kidney Disease	1,424	,688	4,283	1	,039	4,154	1,078	16,004
	Inadequated Definitive Antibiotic Treatment	1,926	,746	6,671	1	,010	6,863	1,591	29,599

Variables not in the Equation[a,b,c,d]

		Score	df	Sig.
Step 1	Diabetes Mellitus	3,383	1	,066
	Chronic Kidney Disease	10,423	1	,001
	Inadequated Definitive Antibiotic Treatment	10,387	1	,001
	Severe Sepsis/Septic Shock	2,057	1	,152
	Charlson Index>3	4,179	1	,041
Step 2	Diabetes Mellitus	1,804	1	,179
	Inadequated Definitive Antibiotic Treatment	5,276	1	,022
	Severe Sepsis/Septic Shock	2,261	1	,133
	Charlson Index>3	1,141	1	,285
Step 3	Diabetes Mellitus	4,330	1	,037
	Severe Sepsis/Septic Shock	1,818	1	,178
	Charlson Index>3	1,159	1	,282
Step 4	Severe Sepsis/Septic Shock	2,280	1	,131
	Charlson Index>3	,747	1	,387

a. Residual Chi Square = 24,873 with 5 df Sig. = ,000

b. Residual Chi Square = 13,862 with 4 df Sig. = ,008

c. Residual Chi Square = 9,409 with 3 df Sig. = ,024

d. Residual Chi Square = 4,900 with 2 df Sig. = ,086

Covariate Means

	Mean
Advanced Chronic Liver Disease	,034
Diabetes Mellitus	,310
Chronic Kidney Disease	,190
Inadequated Definitive Antibiotic Treatment	,259
Severe Sepsis/Septic Shock	,086
Charlson Index>3	,483
