# Supplementary material for: Pseudomonas aeruginosa urinary tract infections in hospitalized patients: Mortality and prognostic factors
Source: PLoS One. 2017 May 26;12(5):e0178178. doi: 10.1371/journal.pone.0178178 (PMC5446154; doi:10.1371/journal.pone.0178178)

SURVIVAL ANALYSIS (KAPLAN-MEIER CURVES AND LOG-RANK TEST)

CARBAPENEM RESISTANT (0=NO, 1=YES)

Case Processing Summary

Carbapenem Resistant	Total N	N of Events	Censored	
			N	Percent
,0	50	9	41	82,0%
1,0	12	2	10	83,3%
Overall	62	11	51	82,3%

Survival Table

Carbapenem Resistant		Time	Status	Cumulative Proportion Surviving at the Time		N of Cumulative Events	N of Remaining Cases
				Estimate	Std. Error		
,0	1	5,000	1,00	,980	,020	1	49
	2	7,000	1,00	.	.	2	48
	3	7,000	1,00	,940	,034	3	47
	4	8,000	1,00	,920	,038	4	46
	5	10,000	1,00	,900	,042	5	45
	6	16,000	1,00	,880	,046	6	44
	7	22,000	1,00	,860	,049	7	43
	8	24,000	1,00	,840	,052	8	42
	9	28,000	1,00	,820	,054	9	41
	10	30,000	,00	.	.	9	40
	11	30,000	,00	.	.	9	39
	12	30,000	,00	.	.	9	38
	13	30,000	,00	.	.	9	37
	14	30,000	,00	.	.	9	36
	15	30,000	,00	.	.	9	35
	16	30,000	,00	.	.	9	34
	17	30,000	,00	.	.	9	33
	18	30,000	,00	.	.	9	32
	19	30,000	,00	.	.	9	31
	20	30,000	,00	.	.	9	30
	21	30,000	,00	.	.	9	29
	22	30,000	,00	.	.	9	28
	23	30,000	,00	.	.	9	27
	24	30,000	,00	.	.	9	26
	25	30,000	,00	.	.	9	25
	26	30,000	,00	.	.	9	24
	27	30,000	,00	.	.	9	23
	28	30,000	,00	.	.	9	22
	29	30,000	,00	.	.	9	21
	30	30,000	,00	.	.	9	20
	31	30,000	,00	.	.	9	19
	32	30,000	,00	.	.	9	18
	33	30,000	,00	.	.	9	17

Survival Table

Carbapenem Resistant		Time	Status	Cumulative Proportion Surviving at the Time		N of Cumulative Events	N of Remaining Cases
				Estimate	Std. Error		
	34	30,000	,00	.	.	9	16
	35	30,000	,00	.	.	9	15
	36	30,000	,00	.	.	9	14
	37	30,000	,00	.	.	9	13
	38	30,000	,00	.	.	9	12
	39	30,000	,00	.	.	9	11
	40	30,000	,00	.	.	9	10
	41	30,000	,00	.	.	9	9
	42	30,000	,00	.	.	9	8
	43	30,000	,00	.	.	9	7
	44	30,000	,00	.	.	9	6
	45	30,000	,00	.	.	9	5
	46	30,000	,00	.	.	9	4
	47	30,000	,00	.	.	9	3
	48	30,000	,00	.	.	9	2
	49	30,000	,00	.	.	9	1
	50	30,000	,00	.	.	9	0
1,0	1	1,000	1,00	,917	,080	1	11
	2	16,000	1,00	,833	,108	2	10
	3	30,000	,00	.	.	2	9
	4	30,000	,00	.	.	2	8
	5	30,000	,00	.	.	2	7
	6	30,000	,00	.	.	2	6
	7	30,000	,00	.	.	2	5
	8	30,000	,00	.	.	2	4
	9	30,000	,00	.	.	2	3
	10	30,000	,00	.	.	2	2
	11	30,000	,00	.	.	2	1
	12	30,000	,00	.	.	2	0

Means and Medians for Survival Time

Carbapenem Resistant	Mean[a]				Median		
	Estimate	Std. Error	95% Confidence Interval		Estimate	Std. Error	95% …
			Lower Bound	Upper Bound			Lower Bound
,0	27,140	,992	25,197	29,083	.	.	.
1,0	26,417	2,476	21,563	31,270	.	.	.
Overall	27,000	,933	25,171	28,829	.	.	.

Means and Medians for Survival Time

Carbapenem Resistant	Median
	95% …
	Upper Bound
,0	.
1,0	.
Overall	.

a. Estimation is limited to the largest survival time if it is censored.

Overall Comparisons

	Chi-Square	df	Sig.
Log Rank (Mantel-Cox)	,004	1	,949

Test of equality of survival distributions for the different levels of Carbapenem Resistant

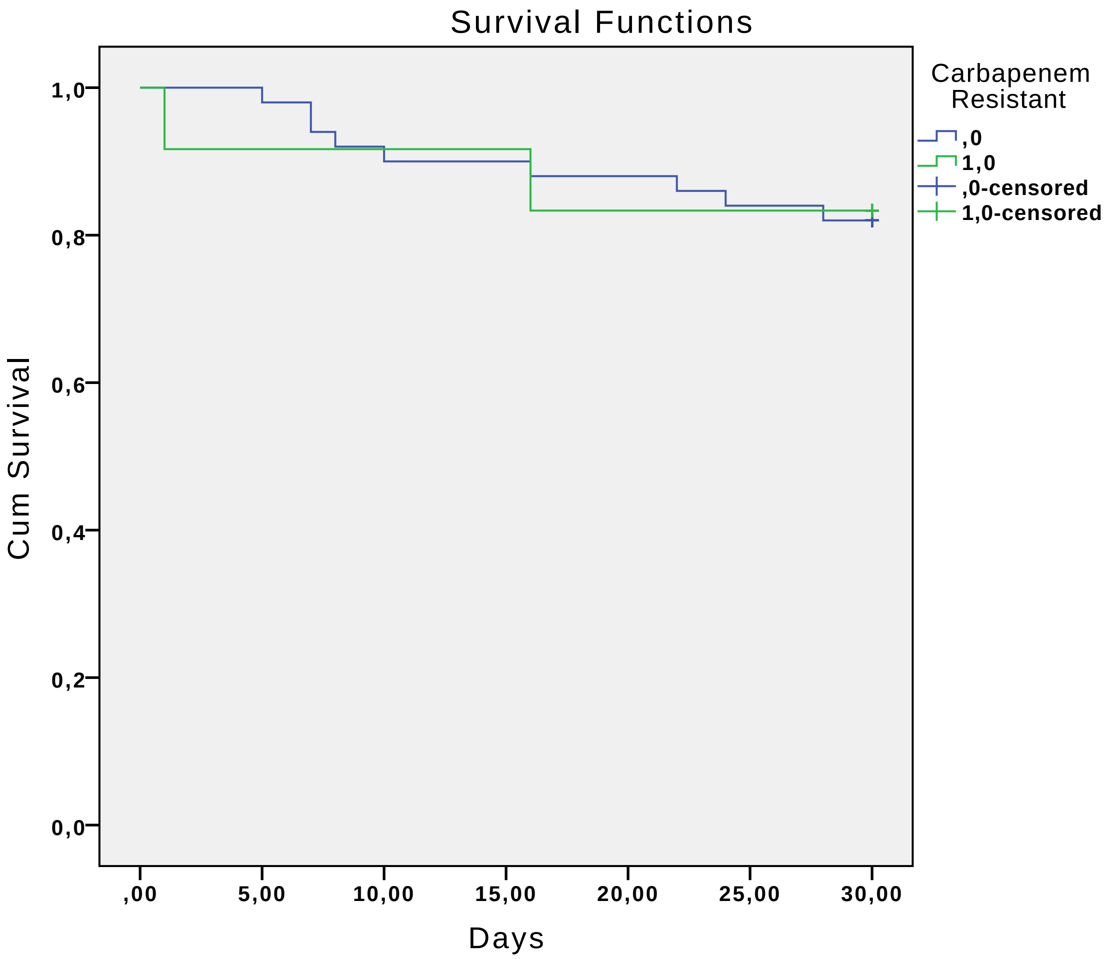

MONOMICROBIAN CULTURE (0=NO, 1=YES)

Case Processing Summary

Monomicrobian Culture	Total N	N of Events	Censored	
			N	Percent
,00	20	6	14	70,0%
1,00	42	5	37	88,1%
Overall	62	11	51	82,3%

Survival Table

Cultivo	Monomicrobiano	Time	Status	Cumulative Proportion Surviving at the Time		N of Cumulative Events	N of Remaining Cases
				Estimate	Std. Error		
,00	1	7,000	1,00	.	.	1	19
	2	7,000	1,00	,900	,067	2	18
	3	8,000	1,00	,850	,080	3	17
	4	10,000	1,00	,800	,089	4	16
	5	16,000	1,00	,750	,097	5	15
	6	24,000	1,00	,700	,102	6	14
	7	30,000	,00	.	.	6	13
	8	30,000	,00	.	.	6	12
	9	30,000	,00	.	.	6	11
	10	30,000	,00	.	.	6	10
	11	30,000	,00	.	.	6	9
	12	30,000	,00	.	.	6	8
	13	30,000	,00	.	.	6	7
	14	30,000	,00	.	.	6	6
	15	30,000	,00	.	.	6	5
	16	30,000	,00	.	.	6	4
	17	30,000	,00	.	.	6	3
	18	30,000	,00	.	.	6	2
	19	30,000	,00	.	.	6	1
	20	30,000	,00	.	.	6	0
1,00	1	1,000	1,00	,976	,024	1	41
	2	5,000	1,00	,952	,033	2	40
	3	16,000	1,00	,929	,040	3	39
	4	22,000	1,00	,905	,045	4	38
	5	28,000	1,00	,881	,050	5	37
	6	30,000	,00	.	.	5	36
	7	30,000	,00	.	.	5	35
	8	30,000	,00	.	.	5	34
	9	30,000	,00	.	.	5	33
	10	30,000	,00	.	.	5	32
	11	30,000	,00	.	.	5	31
	12	30,000	,00	.	.	5	30
	13	30,000	,00	.	.	5	29
	14	30,000	,00	.	.	5	28
	15	30,000	,00	.	.	5	27
	16	30,000	,00	.	.	5	26
	17	30,000	,00	.	.	5	25
	18	30,000	,00	.	.	5	24
	19	30,000	,00	.	.	5	23
	20	30,000	,00	.	.	5	22
	21	30,000	,00	.	.	5	21
	22	30,000	,00	.	.	5	20
	23	30,000	,00	.	.	5	19
	24	30,000	,00	.	.	5	18
	25	30,000	,00	.	.	5	17
	26	30,000	,00	.	.	5	16
	27	30,000	,00	.	.	5	15
	28	30,000	,00	.	.	5	14
	29	30,000	,00	.	.	5	13

Survival Table

Cultivo Monomicrobiano	Time	Status	Cumulative Proportion Surviving at the Time		N of Cumulative Events	N of Remaining Cases
			Estimate	Std. Error		
30	30,000	,00	.	.	5	12
31	30,000	,00	.	.	5	11
32	30,000	,00	.	.	5	10
33	30,000	,00	.	.	5	9
34	30,000	,00	.	.	5	8
35	30,000	,00	.	.	5	7
36	30,000	,00	.	.	5	6
37	30,000	,00	.	.	5	5
38	30,000	,00	.	.	5	4
39	30,000	,00	.	.	5	3
40	30,000	,00	.	.	5	2
41	30,000	,00	.	.	5	1
42	30,000	,00	.	.	5	0

Means and Medians for Survival Time

Monomicrobian Culture	Mean[a]				Median		
			95% Confidence Interval				95% …
	Estimate	Std. Error	Lower Bound	Upper Bound	Estimate	Std. Error	Lower Bound
,00	24,600	1,994	20,691	28,509	.	.	.
1,00	28,143	,948	26,285	30,001	.	.	.
Overall	27,000	,933	25,171	28,829	.	.	.

Means and Medians for Survival Time

Monomicrobian Culture	Median
	95% …
	Upper Bound
,00	.
1,00	.
Overall	.

a. Estimation is limited to the largest survival time if it is censored.

Overall Comparisons

	Chi-Square	df	Sig.
Log Rank (Mantel-Cox)	3,106	1	,078

Test of equality of survival distributions for the different levels of Monomicrobian Culture

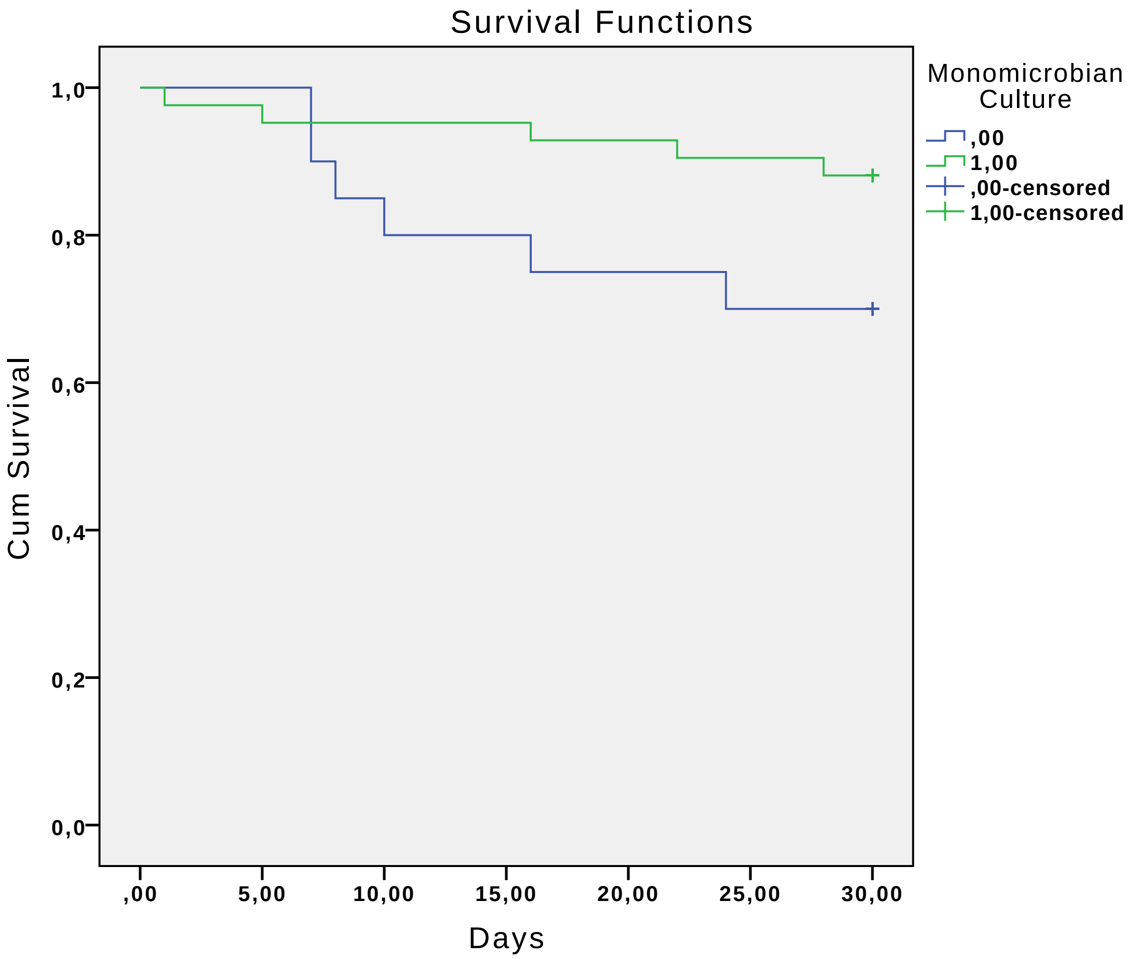

ADEQUATED EMPIRICAL ANTIBIOTIC TREATMENT (0=NO, 1=YES)

Case Processing Summary

Adequated Empirical Antibiotic Treatment	Total N	N of Events	Censored	
			N	Percent
,0	38	7	31	81,6%
1,0	15	3	12	80,0%
Overall	53	10	43	81,1%

Survival Table

Adequated Empirical Antibiotic Treatment		Time	Status	Cumulative Proportion Surviving at the Time		N of Cumulative Events
				Estimate	Std. Error	
,0	1	1,000	1,00	,974	,026	1
	2	5,000	1,00	,947	,036	2
	3	7,000	1,00	,921	,044	3
	4	8,000	1,00	,895	,050	4
	5	16,000	1,00	,868	,055	5
	6	24,000	1,00	,842	,059	6
	7	28,000	1,00	,816	,063	7
	8	30,000	,00	.	.	7
	9	30,000	,00	.	.	7
	10	30,000	,00	.	.	7
	11	30,000	,00	.	.	7
	12	30,000	,00	.	.	7
	13	30,000	,00	.	.	7
	14	30,000	,00	.	.	7
	15	30,000	,00	.	.	7
	16	30,000	,00	.	.	7
	17	30,000	,00	.	.	7
	18	30,000	,00	.	.	7
	19	30,000	,00	.	.	7
	20	30,000	,00	.	.	7
	21	30,000	,00	.	.	7
	22	30,000	,00	.	.	7
	23	30,000	,00	.	.	7
	24	30,000	,00	.	.	7
	25	30,000	,00	.	.	7
	26	30,000	,00	.	.	7
	27	30,000	,00	.	.	7
	28	30,000	,00	.	.	7
	29	30,000	,00	.	.	7
	30	30,000	,00	.	.	7
	31	30,000	,00	.	.	7
	32	30,000	,00	.	.	7
	33	30,000	,00	.	.	7
	34	30,000	,00	.	.	7
	35	30,000	,00	.	.	7
	36	30,000	,00	.	.	7
	37	30,000	,00	.	.	7
	38	30,000	,00	.	.	7
1,0	1	7,000	1,00	,933	,064	1
	2	10,000	1,00	,867	,088	2
	3	22,000	1,00	,800	,103	3
	4	30,000	,00	.	.	3
	5	30,000	,00	.	.	3
	6	30,000	,00	.	.	3
	7	30,000	,00	.	.	3
	8	30,000	,00	.	.	3
	9	30,000	,00	.	.	3
	10	30,000	,00	.	.	3
	11	30,000	,00	.	.	3

Survival Table

Adequated Empirical Antibiotic Treatment		N of Remaining Cases
,0	1	37
	2	36
	3	35
	4	34
	5	33
	6	32
	7	31
	8	30
	9	29
	10	28
	11	27
	12	26
	13	25
	14	24
	15	23
	16	22
	17	21
	18	20
	19	19
	20	18
	21	17
	22	16
	23	15
	24	14
	25	13
	26	12
	27	11
	28	10
	29	9
	30	8
	31	7
	32	6
	33	5
	34	4
	35	3
	36	2
	37	1
	38	0
1,0	1	14
	2	13
	3	12
	4	11
	5	10
	6	9
	7	8
	8	7
	9	6
	10	5
	11	4

Survival Table

Adequated Empirical Antibiotic Treatment	Time	Status	Cumulative Proportion Surviving at the Time		N of Cumulative Events
			Estimate	Std. Error	
12	30,000	,00	.	.	3
13	30,000	,00	.	.	3
14	30,000	,00	.	.	3
15	30,000	,00	.	.	3

Survival Table

Adequated Empirical Antibiotic Treatment	N of Remaining Cases
12	3
13	2
14	1
15	0

Means and Medians for Survival Time

Adequated Empirical Antibiotic Treatment	Mean[a]				Median		
	Estimate	Std. Error	95% Confidence Interval		Estimate	Std. Error	95% …
			Lower Bound	Upper Bound			Lower Bound
,0	26,816	1,270	24,326	29,305	.	.	.
1,0	26,600	1,909	22,859	30,341	.	.	.
Overall	26,755	1,059	24,679	28,830	.	.	.

Means and Medians for Survival Time

Adequated Empirical Antibiotic Treatment	Median
	95% …
	Upper Bound
,0	.
1,0	.
Overall	.

a. Estimation is limited to the largest survival time if it is censored.

Overall Comparisons

	Chi-Square	df	Sig.
Log Rank (Mantel-Cox)	,013	1	,908

Test of equality of survival distributions for the different levels of Adequated Empirical Antibiotic Treatment

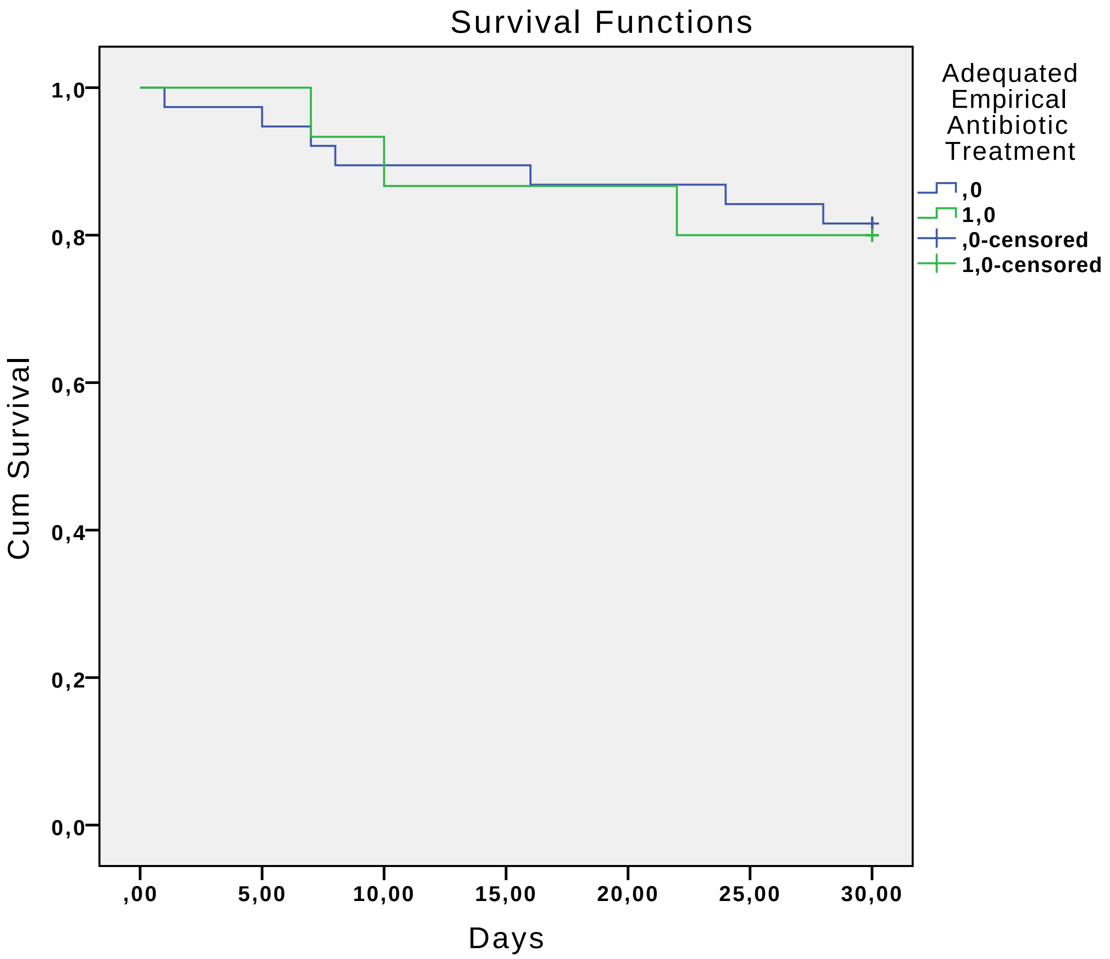

FEMALE SEX (0=NO, 1=YES)

Case Processing Summary

Female Sex	Total N	N of Events	Censored	
			N	Percent
,0	32	6	26	81,3%
1,0	30	5	25	83,3%
Overall	62	11	51	82,3%

Survival Table

Female Sex		Time	Status	Cumulative Proportion Surviving at the Time		N of Cumulative Events	N of Remaining Cases
				Estimate	Std. Error		
,0	1	1,000	1,00	,969	,031	1	31
	2	10,000	1,00	,938	,043	2	30
	3	16,000	1,00	.	.	3	29
	4	16,000	1,00	,875	,058	4	28
	5	24,000	1,00	,844	,064	5	27
	6	28,000	1,00	,813	,069	6	26
	7	30,000	,00	.	.	6	25
	8	30,000	,00	.	.	6	24
	9	30,000	,00	.	.	6	23
	10	30,000	,00	.	.	6	22
	11	30,000	,00	.	.	6	21
	12	30,000	,00	.	.	6	20
	13	30,000	,00	.	.	6	19
	14	30,000	,00	.	.	6	18
	15	30,000	,00	.	.	6	17
	16	30,000	,00	.	.	6	16
	17	30,000	,00	.	.	6	15
	18	30,000	,00	.	.	6	14
	19	30,000	,00	.	.	6	13
	20	30,000	,00	.	.	6	12
	21	30,000	,00	.	.	6	11
	22	30,000	,00	.	.	6	10
	23	30,000	,00	.	.	6	9
	24	30,000	,00	.	.	6	8
	25	30,000	,00	.	.	6	7
	26	30,000	,00	.	.	6	6
	27	30,000	,00	.	.	6	5
	28	30,000	,00	.	.	6	4
	29	30,000	,00	.	.	6	3
	30	30,000	,00	.	.	6	2
	31	30,000	,00	.	.	6	1
	32	30,000	,00	.	.	6	0
1,0	1	5,000	1,00	,967	,033	1	29
	2	7,000	1,00	.	.	2	28
	3	7,000	1,00	,900	,055	3	27
	4	8,000	1,00	,867	,062	4	26
	5	22,000	1,00	,833	,068	5	25
	6	30,000	,00	.	.	5	24
	7	30,000	,00	.	.	5	23
	8	30,000	,00	.	.	5	22
	9	30,000	,00	.	.	5	21
	10	30,000	,00	.	.	5	20
	11	30,000	,00	.	.	5	19
	12	30,000	,00	.	.	5	18
	13	30,000	,00	.	.	5	17
	14	30,000	,00	.	.	5	16
	15	30,000	,00	.	.	5	15
	16	30,000	,00	.	.	5	14
	17	30,000	,00	.	.	5	13

Survival Table

Female Sex	Time	Status	Cumulative Proportion Surviving at the Time		N of Cumulative Events	N of Remaining Cases
			Estimate	Std. Error		
18	30,000	,00	.	.	5	12
19	30,000	,00	.	.	5	11
20	30,000	,00	.	.	5	10
21	30,000	,00	.	.	5	9
22	30,000	,00	.	.	5	8
23	30,000	,00	.	.	5	7
24	30,000	,00	.	.	5	6
25	30,000	,00	.	.	5	5
26	30,000	,00	.	.	5	4
27	30,000	,00	.	.	5	3
28	30,000	,00	.	.	5	2
29	30,000	,00	.	.	5	1
30	30,000	,00	.	.	5	0

Means and Medians for Survival Time

Female Sex	Mean[a]				Median			
	Estimate	Std. Error	95% Confidence Interval		Estimate	Std. Error	95% Confidence Interval	
			Lower Bound	Upper Bound			Lower Bound	Upper Bound
,0	27,344	1,189	25,014	29,674	.	.	.	.
1,0	26,633	1,450	23,792	29,474	.	.	.	.
Overall	27,000	,933	25,171	28,829	.	.	.	.

a. Estimation is limited to the largest survival time if it is censored.

Overall Comparisons

	Chi-Square	df	Sig.
Log Rank (Mantel-Cox)	,019	1	,891

Test of equality of survival distributions for the different levels of Female Sex

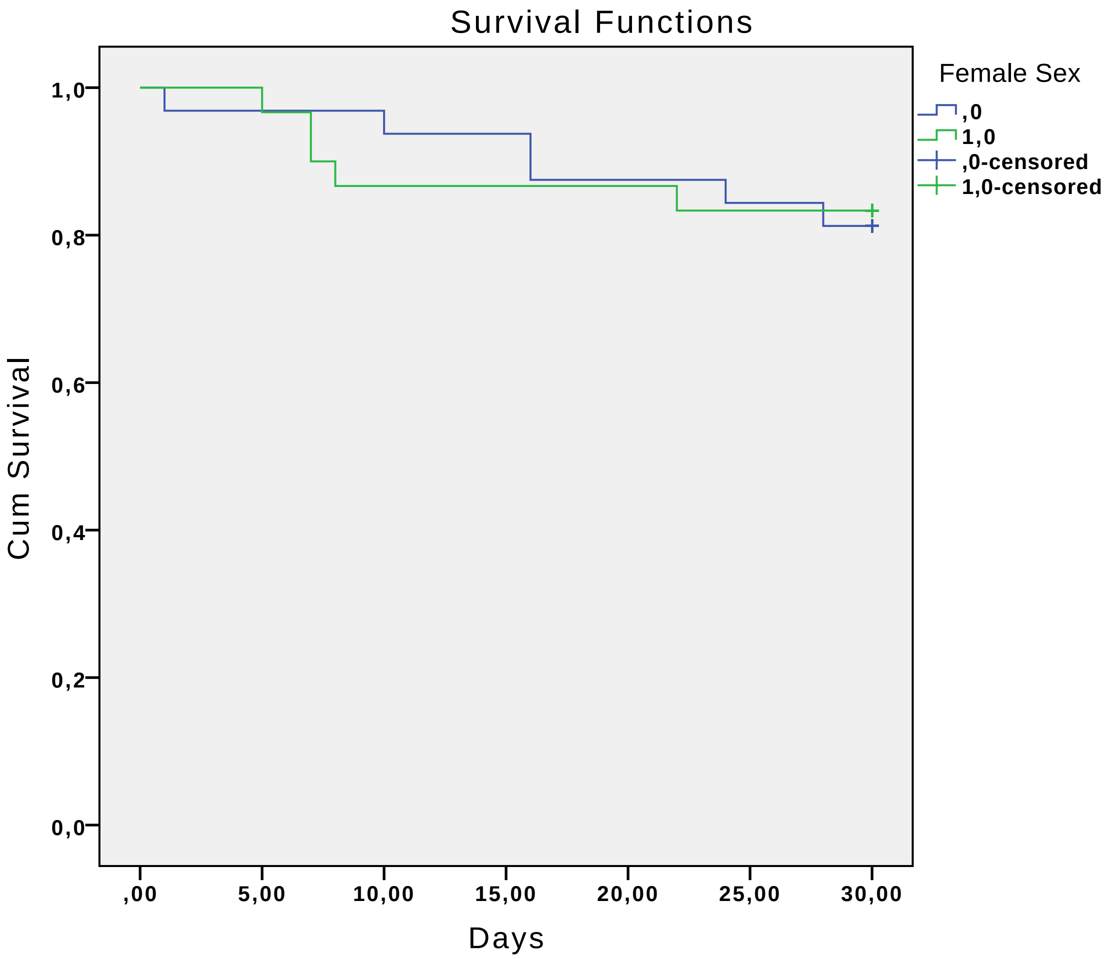

MALNUTRITION (0=NO, 1=YES)

Case Processing Summary

Malnutrition	Total N	N of Events	Censored	
			N	Percent
,0	43	8	35	81,4%
1,0	12	3	9	75,0%
Overall	55	11	44	80,0%

Survival Table

Malnutrition		Time	Status	Cumulative Proportion Surviving at the Time		N of Cumulative Events	N of Remaining Cases
				Estimate	Std. Error		
,0	1	1,000	1,00	,977	,023	1	42
	2	7,000	1,00	,953	,032	2	41
	3	8,000	1,00	,930	,039	3	40
	4	10,000	1,00	,907	,044	4	39
	5	16,000	1,00	.	.	5	38
	6	16,000	1,00	,860	,053	6	37
	7	24,000	1,00	,837	,056	7	36
	8	28,000	1,00	,814	,059	8	35
	9	30,000	,00	.	.	8	34
	10	30,000	,00	.	.	8	33
	11	30,000	,00	.	.	8	32
	12	30,000	,00	.	.	8	31
	13	30,000	,00	.	.	8	30
	14	30,000	,00	.	.	8	29
	15	30,000	,00	.	.	8	28
	16	30,000	,00	.	.	8	27
	17	30,000	,00	.	.	8	26
	18	30,000	,00	.	.	8	25
	19	30,000	,00	.	.	8	24
	20	30,000	,00	.	.	8	23
	21	30,000	,00	.	.	8	22
	22	30,000	,00	.	.	8	21
	23	30,000	,00	.	.	8	20
	24	30,000	,00	.	.	8	19
	25	30,000	,00	.	.	8	18
	26	30,000	,00	.	.	8	17
	27	30,000	,00	.	.	8	16
	28	30,000	,00	.	.	8	15
	29	30,000	,00	.	.	8	14
	30	30,000	,00	.	.	8	13
	31	30,000	,00	.	.	8	12
	32	30,000	,00	.	.	8	11
	33	30,000	,00	.	.	8	10
	34	30,000	,00	.	.	8	9
	35	30,000	,00	.	.	8	8
	36	30,000	,00	.	.	8	7
	37	30,000	,00	.	.	8	6
	38	30,000	,00	.	.	8	5
	39	30,000	,00	.	.	8	4
	40	30,000	,00	.	.	8	3
	41	30,000	,00	.	.	8	2
	42	30,000	,00	.	.	8	1
	43	30,000	,00	.	.	8	0
1,0	1	5,000	1,00	,917	,080	1	11
	2	7,000	1,00	,833	,108	2	10
	3	22,000	1,00	,750	,125	3	9
	4	30,000	,00	.	.	3	8
	5	30,000	,00	.	.	3	7
	6	30,000	,00	.	.	3	6

Survival Table

Malnutrition	Time	Status	Cumulative Proportion Surviving at the Time		N of Cumulative Events	N of Remaining Cases
			Estimate	Std. Error		
7	30,000	,00	.	.	3	5
8	30,000	,00	.	.	3	4
9	30,000	,00	.	.	3	3
10	30,000	,00	.	.	3	2
11	30,000	,00	.	.	3	1
12	30,000	,00	.	.	3	0

Means and Medians for Survival Time

Malnutrition	Mean[a]				Median			
	Estimate	Std. Error	95% Confidence Interval		Estimate	Std. Error	95% Confidence Interval	
			Lower Bound	Upper Bound			Lower Bound	Upper Bound
,0	26,977	1,114	24,794	29,159	.	.	.	.
1,0	25,333	2,578	20,281	30,385	.	.	.	.
Overall	26,618	1,040	24,579	28,658	.	.	.	.

a. Estimation is limited to the largest survival time if it is censored.

Overall Comparisons

	Chi-Square	df	Sig.
Log Rank (Mantel-Cox)	,300	1	,584

Test of equality of survival distributions for the different levels of Malnutrition

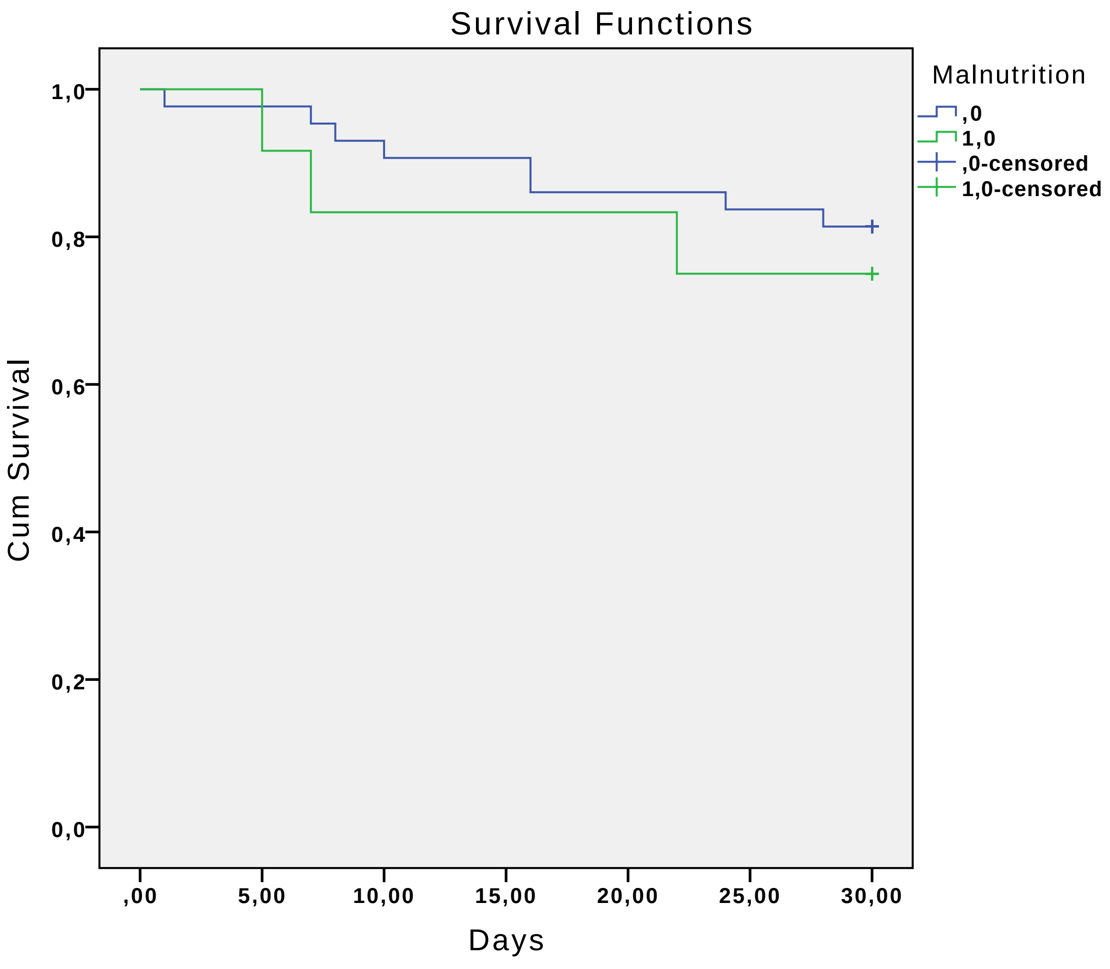

OBESITY (0=NO, 1=YES)

Case Processing Summary

Obesity	Total N	N of Events	Censored	
			N	Percent
,0	39	7	32	82,1%
1,0	15	3	12	80,0%
Overall	54	10	44	81,5%

Survival Table

Obesity		Time	Status	Cumulative Proportion Surviving at the Time		N of Cumulative Events	N of Remaining Cases
				Estimate	Std. Error		
,0	1	1,000	1,00	,974	,025	1	38
	2	5,000	1,00	,949	,035	2	37
	3	7,000	1,00	,923	,043	3	36
	4	10,000	1,00	,897	,049	4	35
	5	16,000	1,00	.	.	5	34
	6	16,000	1,00	,846	,058	6	33
	7	22,000	1,00	,821	,061	7	32
	8	30,000	,00	.	.	7	31
	9	30,000	,00	.	.	7	30
	10	30,000	,00	.	.	7	29
	11	30,000	,00	.	.	7	28
	12	30,000	,00	.	.	7	27
	13	30,000	,00	.	.	7	26
	14	30,000	,00	.	.	7	25
	15	30,000	,00	.	.	7	24
	16	30,000	,00	.	.	7	23
	17	30,000	,00	.	.	7	22
	18	30,000	,00	.	.	7	21
	19	30,000	,00	.	.	7	20
	20	30,000	,00	.	.	7	19
	21	30,000	,00	.	.	7	18
	22	30,000	,00	.	.	7	17
	23	30,000	,00	.	.	7	16
	24	30,000	,00	.	.	7	15
	25	30,000	,00	.	.	7	14
	26	30,000	,00	.	.	7	13
	27	30,000	,00	.	.	7	12
	28	30,000	,00	.	.	7	11
	29	30,000	,00	.	.	7	10
	30	30,000	,00	.	.	7	9
	31	30,000	,00	.	.	7	8
	32	30,000	,00	.	.	7	7
	33	30,000	,00	.	.	7	6
	34	30,000	,00	.	.	7	5
	35	30,000	,00	.	.	7	4
	36	30,000	,00	.	.	7	3
	37	30,000	,00	.	.	7	2
	38	30,000	,00	.	.	7	1
	39	30,000	,00	.	.	7	0

Survival Table

Obesity		Time	Status	Cumulative Proportion Surviving at the Time		N of Cumulative Events	N of Remaining Cases
				Estimate	Std. Error		
1,0	1	7,000	1,00	,933	,064	1	14
	2	8,000	1,00	,867	,088	2	13
	3	28,000	1,00	,800	,103	3	12
	4	30,000	,00	.	.	3	11
	5	30,000	,00	.	.	3	10
	6	30,000	,00	.	.	3	9
	7	30,000	,00	.	.	3	8
	8	30,000	,00	.	.	3	7
	9	30,000	,00	.	.	3	6
	10	30,000	,00	.	.	3	5
	11	30,000	,00	.	.	3	4
	12	30,000	,00	.	.	3	3
	13	30,000	,00	.	.	3	2
	14	30,000	,00	.	.	3	1
	15	30,000	,00	.	.	3	0

Means and Medians for Survival Time

Obesity	Mean[a]				Median			
	Estimate	Std. Error	95% Confidence Interval		Estimate	Std. Error	95% Confidence Interval	
			Lower Bound	Upper Bound			Lower Bound	Upper Bound
,0	26,590	1,255	24,129	29,050	.	.	.	.
1,0	26,867	1,966	23,013	30,720	.	.	.	.
Overall	26,667	1,059	24,592	28,742	.	.	.	.

a. Estimation is limited to the largest survival time if it is censored.

Overall Comparisons

	Chi-Square	df	Sig.
Log Rank (Mantel-Cox)	,021	1	,885

Test of equality of survival distributions for the different levels of Obesity

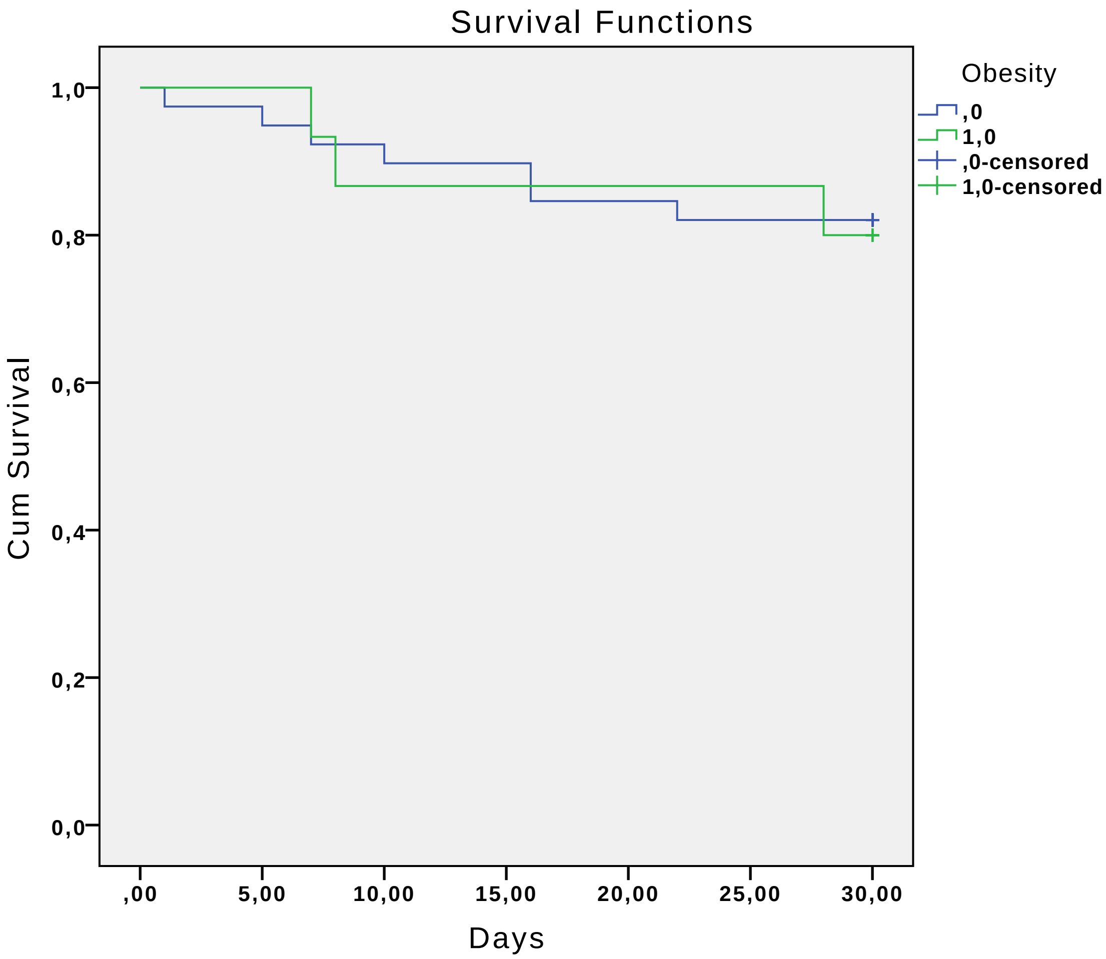

INTENSIVE CARE UNIT (0=NO, 1=YES)

Case Processing Summary

Intensive Care Unit	Total N	N of Events	Censored	
			N	Percent
,0	48	9	39	81,3%
1,0	14	2	12	85,7%
Overall	62	11	51	82,3%

Survival Table

Intensive Care Unit		Time	Status	Cumulative Proportion Surviving at the Time		N of Cumulative Events	N of Remaining Cases
				Estimate	Std. Error		
,0	1	1,000	1,00	,979	,021	1	47
	2	7,000	1,00	.	.	2	46
	3	7,000	1,00	,938	,035	3	45
	4	8,000	1,00	,917	,040	4	44
	5	10,000	1,00	,896	,044	5	43
	6	16,000	1,00	,875	,048	6	42
	7	22,000	1,00	,854	,051	7	41
	8	24,000	1,00	,833	,054	8	40
	9	28,000	1,00	,813	,056	9	39
	10	30,000	,00	.	.	9	38
	11	30,000	,00	.	.	9	37
	12	30,000	,00	.	.	9	36
	13	30,000	,00	.	.	9	35
	14	30,000	,00	.	.	9	34
	15	30,000	,00	.	.	9	33
	16	30,000	,00	.	.	9	32
	17	30,000	,00	.	.	9	31
	18	30,000	,00	.	.	9	30
	19	30,000	,00	.	.	9	29
	20	30,000	,00	.	.	9	28
	21	30,000	,00	.	.	9	27
	22	30,000	,00	.	.	9	26
	23	30,000	,00	.	.	9	25
	24	30,000	,00	.	.	9	24
	25	30,000	,00	.	.	9	23
	26	30,000	,00	.	.	9	22
	27	30,000	,00	.	.	9	21
	28	30,000	,00	.	.	9	20
	29	30,000	,00	.	.	9	19
	30	30,000	,00	.	.	9	18
	31	30,000	,00	.	.	9	17
	32	30,000	,00	.	.	9	16
	33	30,000	,00	.	.	9	15
	34	30,000	,00	.	.	9	14
	35	30,000	,00	.	.	9	13
	36	30,000	,00	.	.	9	12
	37	30,000	,00	.	.	9	11
	38	30,000	,00	.	.	9	10
	39	30,000	,00	.	.	9	9
	40	30,000	,00	.	.	9	8
	41	30,000	,00	.	.	9	7
	42	30,000	,00	.	.	9	6
	43	30,000	,00	.	.	9	5
	44	30,000	,00	.	.	9	4
	45	30,000	,00	.	.	9	3
	46	30,000	,00	.	.	9	2
	47	30,000	,00	.	.	9	1
	48	30,000	,00	.	.	9	0

Survival Table

Intensive Care Unit		Time	Status	Cumulative Proportion Surviving at the Time		N of Cumulative Events	N of Remaining Cases
				Estimate	Std. Error		
1,0	1	5,000	1,00	,929	,069	1	13
	2	16,000	1,00	,857	,094	2	12
	3	30,000	,00	.	.	2	11
	4	30,000	,00	.	.	2	10
	5	30,000	,00	.	.	2	9
	6	30,000	,00	.	.	2	8
	7	30,000	,00	.	.	2	7
	8	30,000	,00	.	.	2	6
	9	30,000	,00	.	.	2	5
	10	30,000	,00	.	.	2	4
	11	30,000	,00	.	.	2	3
	12	30,000	,00	.	.	2	2
	13	30,000	,00	.	.	2	1
	14	30,000	,00	.	.	2	0

Means and Medians for Survival Time

Intensive Care Unit	Mean[a]				Median		
			95% Confidence Interval				95% …
	Estimate	Std. Error	Lower Bound	Upper Bound	Estimate	Std. Error	Lower Bound
,0	26,938	1,069	24,842	29,033	.	.	.
1,0	27,214	1,906	23,478	30,951	.	.	.
Overall	27,000	,933	25,171	28,829	.	.	.

Means and Medians for Survival Time

Intensive Care Unit	Median
	95% …
	Upper Bound
,0	.
1,0	.
Overall	.

a. Estimation is limited to the largest survival time if it is censored.

Overall Comparisons

	Chi-Square	df	Sig.
Log Rank (Mantel-Cox)	,123	1	,726

Test of equality of survival distributions for the different levels of Intensive Care Unit

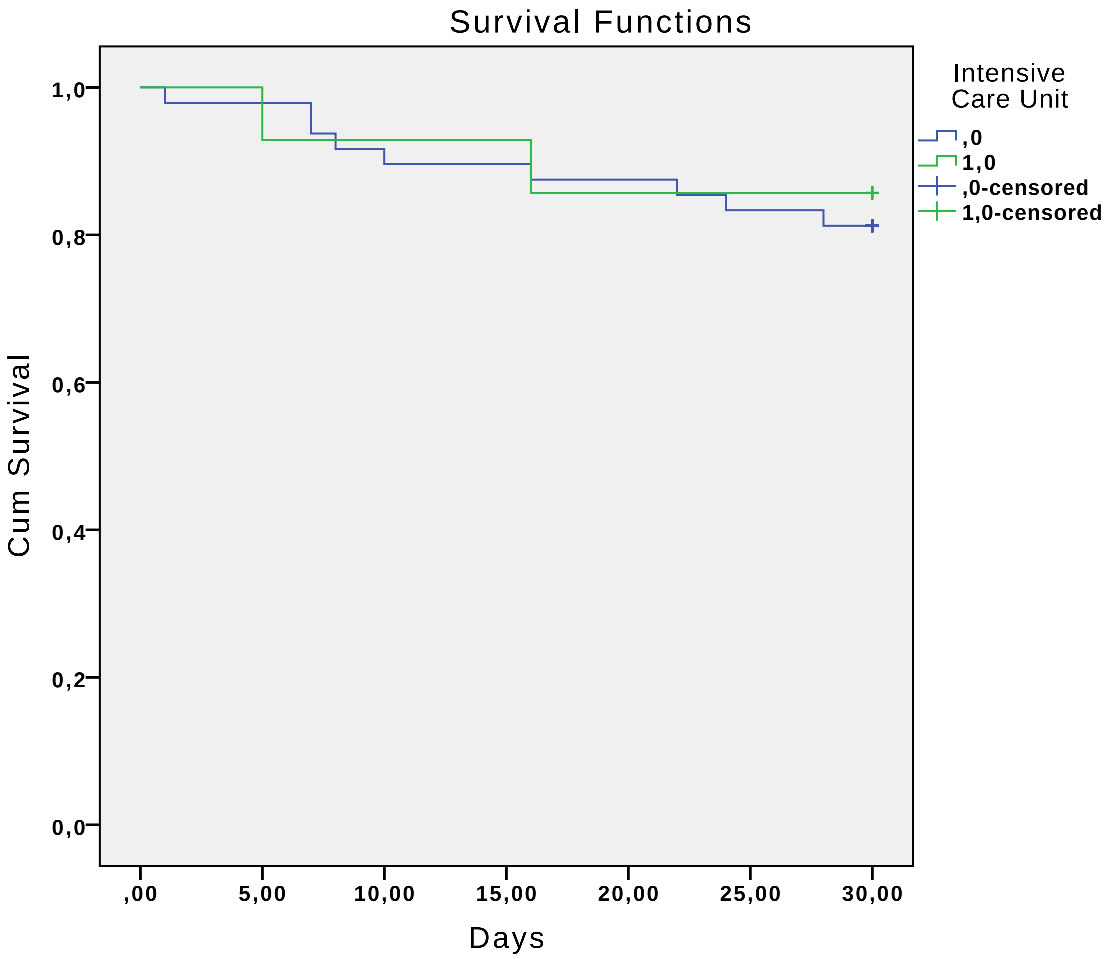

NOSOCOMIAL (0=NO, 1=YES)

Case Processing Summary

NOSOCOMIAL	Total N	N of Events	Censored	
			N	Percent
,0	34	6	28	82,4%
1,0	27	5	22	81,5%
Overall	61	11	50	82,0%

Survival Table

NOSOCOMIAL		Time	Status	Cumulative Proportion Surviving at the Time		N of Cumulative Events	N of Remaining Cases
				Estimate	Std. Error		
,0	1	1,000	1,00	,971	,029	1	33
	2	7,000	1,00	,941	,040	2	32
	3	10,000	1,00	,912	,049	3	31
	4	22,000	1,00	,882	,055	4	30
	5	24,000	1,00	,853	,061	5	29
	6	28,000	1,00	,824	,065	6	28
	7	30,000	,00	.	.	6	27
	8	30,000	,00	.	.	6	26
	9	30,000	,00	.	.	6	25
	10	30,000	,00	.	.	6	24
	11	30,000	,00	.	.	6	23
	12	30,000	,00	.	.	6	22
	13	30,000	,00	.	.	6	21
	14	30,000	,00	.	.	6	20
	15	30,000	,00	.	.	6	19
	16	30,000	,00	.	.	6	18
	17	30,000	,00	.	.	6	17
	18	30,000	,00	.	.	6	16
	19	30,000	,00	.	.	6	15
	20	30,000	,00	.	.	6	14
	21	30,000	,00	.	.	6	13
	22	30,000	,00	.	.	6	12
	23	30,000	,00	.	.	6	11
	24	30,000	,00	.	.	6	10
	25	30,000	,00	.	.	6	9
	26	30,000	,00	.	.	6	8
	27	30,000	,00	.	.	6	7
	28	30,000	,00	.	.	6	6
	29	30,000	,00	.	.	6	5
	30	30,000	,00	.	.	6	4
	31	30,000	,00	.	.	6	3
	32	30,000	,00	.	.	6	2
	33	30,000	,00	.	.	6	1
	34	30,000	,00	.	.	6	0
1,0	1	5,000	1,00	,963	,036	1	26
	2	7,000	1,00	,926	,050	2	25
	3	8,000	1,00	,889	,060	3	24
	4	16,000	1,00	.	.	4	23
	5	16,000	1,00	,815	,075	5	22
	6	30,000	,00	.	.	5	21
	7	30,000	,00	.	.	5	20
	8	30,000	,00	.	.	5	19
	9	30,000	,00	.	.	5	18
	10	30,000	,00	.	.	5	17
	11	30,000	,00	.	.	5	16
	12	30,000	,00	.	.	5	15
	13	30,000	,00	.	.	5	14
	14	30,000	,00	.	.	5	13
	15	30,000	,00	.	.	5	12

Survival Table

NOSOCOMIAL	Time	Status	Cumulative Proportion Surviving at the Time – Estimate	Cumulative Proportion Surviving at the Time – Std. Error	N of Cumulative Events	N of Remaining Cases
16	30,000	,00	.	.	5	11
17	30,000	,00	.	.	5	10
18	30,000	,00	.	.	5	9
19	30,000	,00	.	.	5	8
20	30,000	,00	.	.	5	7
21	30,000	,00	.	.	5	6
22	30,000	,00	.	.	5	5
23	30,000	,00	.	.	5	4
24	30,000	,00	.	.	5	3
25	30,000	,00	.	.	5	2
26	30,000	,00	.	.	5	1
27	30,000	,00	.	.	5	0

Means and Medians for Survival Time

NOSOCOMIAL	Mean[a]				Median		
			95% Confidence Interval				95% …
	Estimate	Std. Error	Lower Bound	Upper Bound	Estimate	Std. Error	Lower Bound
,0	27,412	1,193	25,073	29,751	.	.	.
1,0	26,370	1,515	23,400	29,341	.	.	.
Overall	26,951	,947	25,095	28,807	.	.	.

Means and Medians for Survival Time

NOSOCOMIAL	Median
	95% …
	Upper Bound
,0	.
1,0	.
Overall	.

a. Estimation is limited to the largest survival time if it is censored.

Overall Comparisons

	Chi-Square	df	Sig.
Log Rank (Mantel-Cox)	,017	1	,895

Test of equality of survival distributions for the different levels of NOSOCOMIAL.

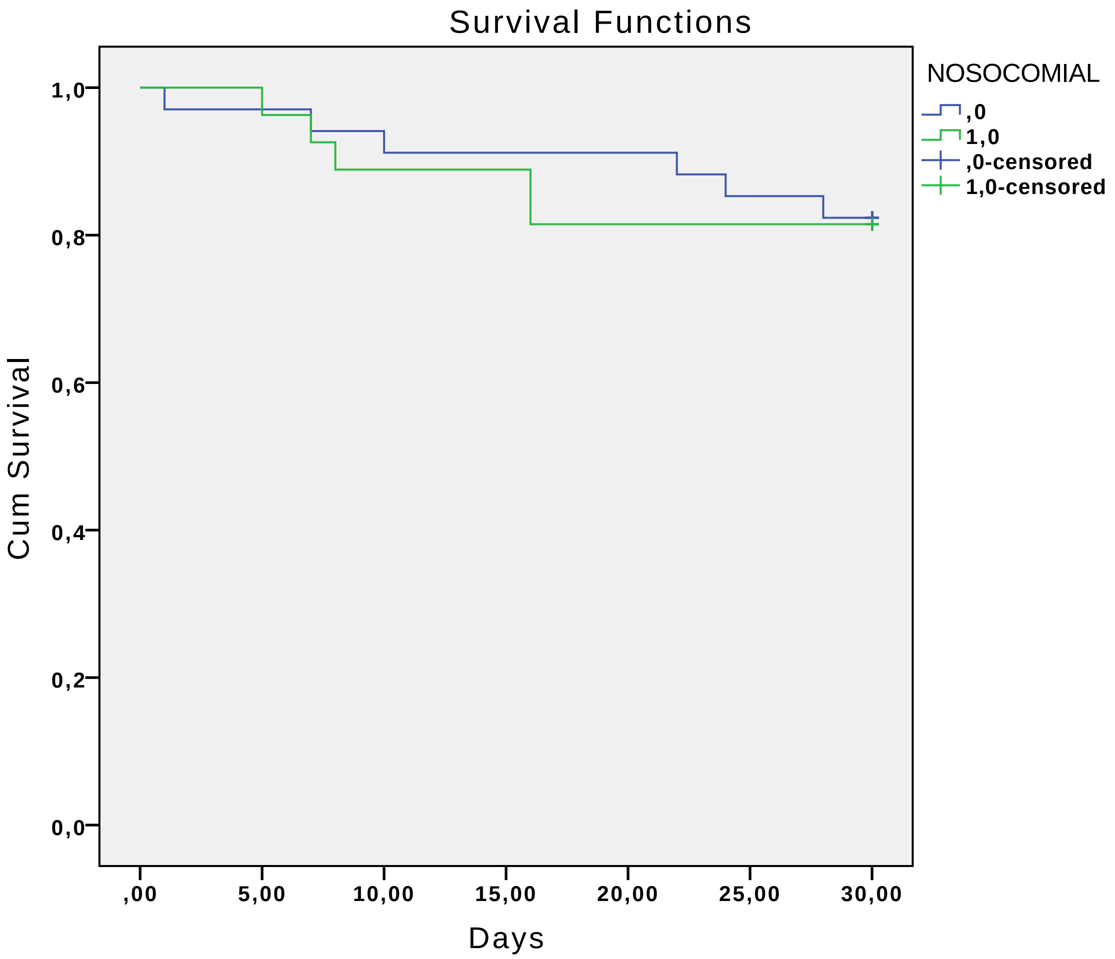

HEALTHCARE ASSOCIATED (0=NO, 1=YES)

Case Processing Summary

Healthcare Associated	Total N	N of Events	Censored	
			N	Percent
,0	41	5	36	87,8%
1,0	20	5	15	75,0%
Overall	61	10	51	83,6%

Survival Table

Healthcare Associated		Time	Status	Cumulative Proportion Surviving at the Time		N of Cumulative Events	N of Remaining Cases
				Estimate	Std. Error		
,0	1	7,000	1,00	,976	,024	1	40
	2	8,000	1,00	,951	,034	2	39
	3	16,000	1,00	.	.	3	38
	4	16,000	1,00	,902	,046	4	37
	5	22,000	1,00	,878	,051	5	36
	6	30,000	,00	.	.	5	35
	7	30,000	,00	.	.	5	34
	8	30,000	,00	.	.	5	33
	9	30,000	,00	.	.	5	32
	10	30,000	,00	.	.	5	31
	11	30,000	,00	.	.	5	30
	12	30,000	,00	.	.	5	29
	13	30,000	,00	.	.	5	28
	14	30,000	,00	.	.	5	27
	15	30,000	,00	.	.	5	26
	16	30,000	,00	.	.	5	25
	17	30,000	,00	.	.	5	24
	18	30,000	,00	.	.	5	23
	19	30,000	,00	.	.	5	22
	20	30,000	,00	.	.	5	21
	21	30,000	,00	.	.	5	20
	22	30,000	,00	.	.	5	19
	23	30,000	,00	.	.	5	18
	24	30,000	,00	.	.	5	17
	25	30,000	,00	.	.	5	16
	26	30,000	,00	.	.	5	15
	27	30,000	,00	.	.	5	14
	28	30,000	,00	.	.	5	13
	29	30,000	,00	.	.	5	12
	30	30,000	,00	.	.	5	11
	31	30,000	,00	.	.	5	10
	32	30,000	,00	.	.	5	9
	33	30,000	,00	.	.	5	8
	34	30,000	,00	.	.	5	7
	35	30,000	,00	.	.	5	6
	36	30,000	,00	.	.	5	5
	37	30,000	,00	.	.	5	4
	38	30,000	,00	.	.	5	3
	39	30,000	,00	.	.	5	2
	40	30,000	,00	.	.	5	1
	41	30,000	,00	.	.	5	0
1,0	1	1,000	1,00	,950	,049	1	19
	2	7,000	1,00	,900	,067	2	18
	3	10,000	1,00	,850	,080	3	17
	4	24,000	1,00	,800	,089	4	16
	5	28,000	1,00	,750	,097	5	15
	6	30,000	,00	.	.	5	14
	7	30,000	,00	.	.	5	13
	8	30,000	,00	.	.	5	12

Survival Table

Healthcare Associated	Time	Status	Cumulative Proportion Surviving at the Time		N of Cumulative Events	N of Remaining Cases
			Estimate	Std. Error		
9	30,000	,00	.	.	5	11
10	30,000	,00	.	.	5	10
11	30,000	,00	.	.	5	9
12	30,000	,00	.	.	5	8
13	30,000	,00	.	.	5	7
14	30,000	,00	.	.	5	6
15	30,000	,00	.	.	5	5
16	30,000	,00	.	.	5	4
17	30,000	,00	.	.	5	3
18	30,000	,00	.	.	5	2
19	30,000	,00	.	.	5	1
20	30,000	,00	.	.	5	0

Means and Medians for Survival Time

Healthcare Associated	Mean[a]				Median		
	Estimate	Std. Error	95% Confidence Interval		Estimate	Std. Error	95% …
			Lower Bound	Upper Bound			Lower Bound
,0	28,024	,882	26,295	29,754	.	.	.
1,0	26,000	1,930	22,217	29,783	.	.	.
Overall	27,361	,876	25,644	29,077	.	.	.

Means and Medians for Survival Time

Healthcare Associated	Median
	95% …
	Upper Bound
,0	.
1,0	.
Overall	.

a. Estimation is limited to the largest survival time if it is censored.

Overall Comparisons

	Chi-Square	df	Sig.
Log Rank (Mantel-Cox)	1,622	1	,203

Test of equality of survival distributions for the different levels of Healthcare Associated

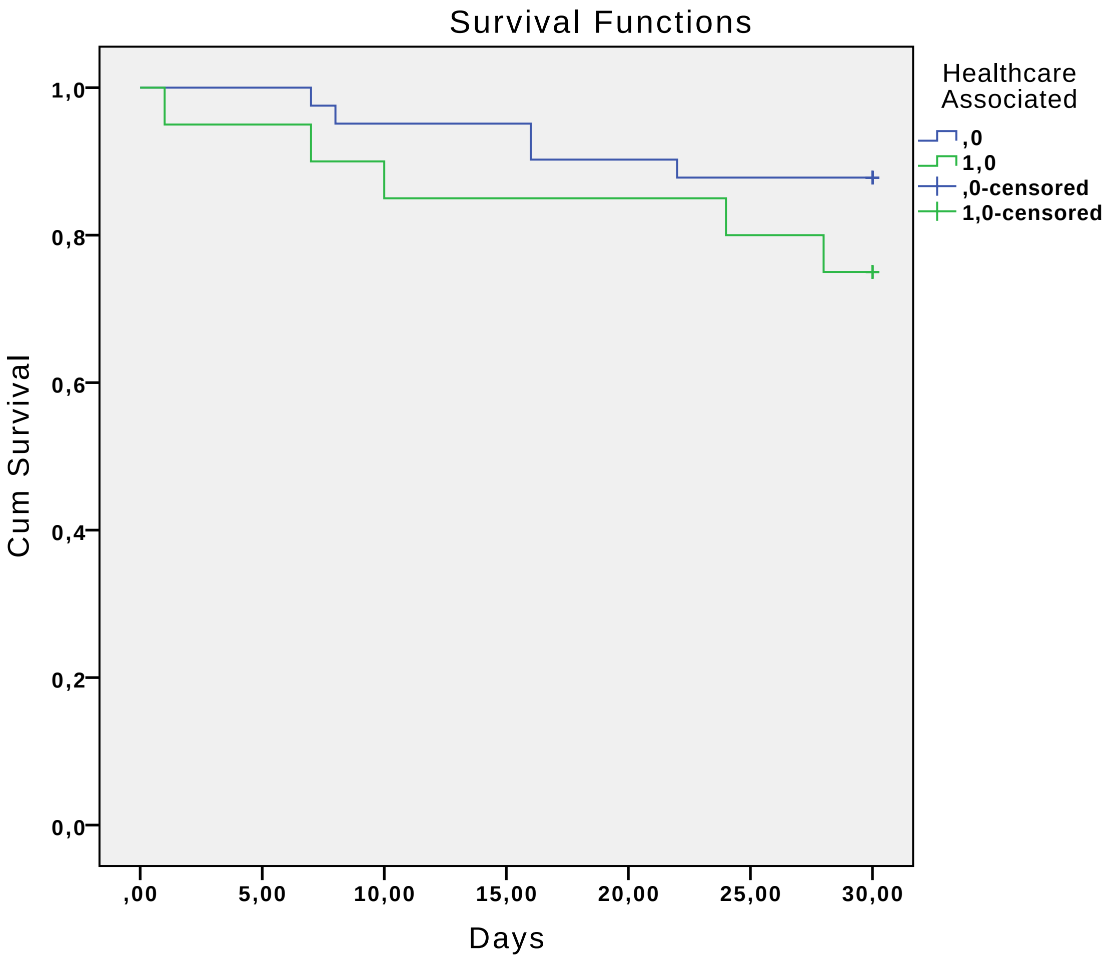

INSTITUCIONALIZED (0=NO, 1=YES)

Warnings

No statistics are computed because all cases are censored.

Case Processing Summary

Institucionalized	Total N	N of Events	Censored	
			N	Percent
,0	57	10	47	82,5%
1,0	3	0	3	100,0%
Overall	60	10	50	83,3%

Survival Table

Institucionalized		Time	Status	Cumulative Proportion Surviving at the Time		N of Cumulative Events	N of Remaining Cases
				Estimate	Std. Error		
,0	1	1,000	1,00	,982	,017	1	56
	2	7,000	1,00	.	.	2	55
	3	7,000	1,00	,947	,030	3	54
	4	8,000	1,00	,930	,034	4	53
	5	10,000	1,00	,912	,037	5	52
	6	16,000	1,00	.	.	6	51
	7	16,000	1,00	,877	,043	7	50
	8	22,000	1,00	,860	,046	8	49
	9	24,000	1,00	,842	,048	9	48
	10	28,000	1,00	,825	,050	10	47
	11	30,000	,00	.	.	10	46
	12	30,000	,00	.	.	10	45
	13	30,000	,00	.	.	10	44
	14	30,000	,00	.	.	10	43
	15	30,000	,00	.	.	10	42
	16	30,000	,00	.	.	10	41
	17	30,000	,00	.	.	10	40
	18	30,000	,00	.	.	10	39
	19	30,000	,00	.	.	10	38
	20	30,000	,00	.	.	10	37
	21	30,000	,00	.	.	10	36
	22	30,000	,00	.	.	10	35
	23	30,000	,00	.	.	10	34
	24	30,000	,00	.	.	10	33
	25	30,000	,00	.	.	10	32
	26	30,000	,00	.	.	10	31
	27	30,000	,00	.	.	10	30
	28	30,000	,00	.	.	10	29
	29	30,000	,00	.	.	10	28
	30	30,000	,00	.	.	10	27
	31	30,000	,00	.	.	10	26
	32	30,000	,00	.	.	10	25
	33	30,000	,00	.	.	10	24
	34	30,000	,00	.	.	10	23
	35	30,000	,00	.	.	10	22
	36	30,000	,00	.	.	10	21
	37	30,000	,00	.	.	10	20
	38	30,000	,00	.	.	10	19
	39	30,000	,00	.	.	10	18
	40	30,000	,00	.	.	10	17
	41	30,000	,00	.	.	10	16
	42	30,000	,00	.	.	10	15
	43	30,000	,00	.	.	10	14
	44	30,000	,00	.	.	10	13
	45	30,000	,00	.	.	10	12
	46	30,000	,00	.	.	10	11
	47	30,000	,00	.	.	10	10
	48	30,000	,00	.	.	10	9
	49	30,000	,00	.	.	10	8

Survival Table

Institucionalized		Time	Status	Cumulative Proportion Surviving at the Time		N of Cumulative Events	N of Remaining Cases
				Estimate	Std. Error		
	50	30,000	,00	.	.	10	7
	51	30,000	,00	.	.	10	6
	52	30,000	,00	.	.	10	5
	53	30,000	,00	.	.	10	4
	54	30,000	,00	.	.	10	3
	55	30,000	,00	.	.	10	2
	56	30,000	,00	.	.	10	1
	57	30,000	,00	.	.	10	0
1,0	1	30,000	,00	.	.	0	2
	2	30,000	,00	.	.	0	1

Overall Comparisons

	Chi-Square	df	Sig.
Log Rank (Mantel-Cox)	,575	1	,448

Test of equality of survival distributions for the different levels of INSTITUCIÓN.

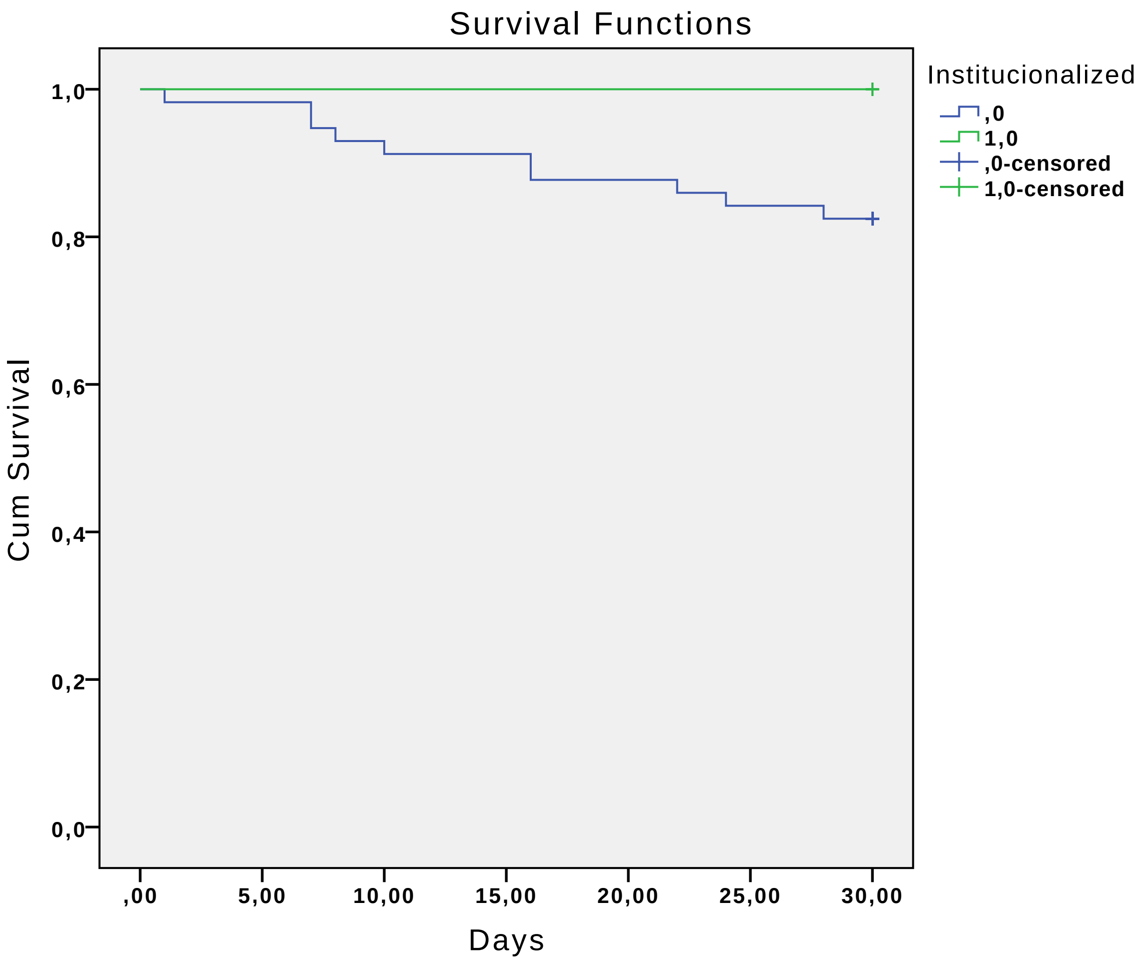

CENTRAL VENOUS CATHETER (0=NO, 1=YES)

Case Processing Summary

Central Venous Catheter	Total N	N of Events	Censored	
			N	Percent
,0	55	10	45	81,8%
1,0	7	1	6	85,7%
Overall	62	11	51	82,3%

Survival Table

Central Venous Catheter		Time	Status	Cumulative Proportion Surviving at the Time		N of Cumulative Events	N of Remaining Cases
				Estimate	Std. Error		
,0	1	1,000	1,00	,982	,018	1	54
	2	7,000	1,00	.	.	2	53
	3	7,000	1,00	,945	,031	3	52
	4	8,000	1,00	,927	,035	4	51
	5	10,000	1,00	,909	,039	5	50
	6	16,000	1,00	.	.	6	49
	7	16,000	1,00	,873	,045	7	48
	8	22,000	1,00	,855	,048	8	47
	9	24,000	1,00	,836	,050	9	46
	10	28,000	1,00	,818	,052	10	45
	11	30,000	,00	.	.	10	44
	12	30,000	,00	.	.	10	43
	13	30,000	,00	.	.	10	42
	14	30,000	,00	.	.	10	41
	15	30,000	,00	.	.	10	40
	16	30,000	,00	.	.	10	39
	17	30,000	,00	.	.	10	38
	18	30,000	,00	.	.	10	37
	19	30,000	,00	.	.	10	36
	20	30,000	,00	.	.	10	35
	21	30,000	,00	.	.	10	34
	22	30,000	,00	.	.	10	33
	23	30,000	,00	.	.	10	32
	24	30,000	,00	.	.	10	31
	25	30,000	,00	.	.	10	30
	26	30,000	,00	.	.	10	29
	27	30,000	,00	.	.	10	28
	28	30,000	,00	.	.	10	27
	29	30,000	,00	.	.	10	26
	30	30,000	,00	.	.	10	25
	31	30,000	,00	.	.	10	24
	32	30,000	,00	.	.	10	23
	33	30,000	,00	.	.	10	22
	34	30,000	,00	.	.	10	21
	35	30,000	,00	.	.	10	20
	36	30,000	,00	.	.	10	19
	37	30,000	,00	.	.	10	18
	38	30,000	,00	.	.	10	17
	39	30,000	,00	.	.	10	16
	40	30,000	,00	.	.	10	15
	41	30,000	,00	.	.	10	14
	42	30,000	,00	.	.	10	13

Survival Table

Central Venous Catheter		Time	Status	Cumulative Proportion Surviving at the Time		N of Cumulative Events	N of Remaining Cases
				Estimate	Std. Error		
	43	30,000	,00	.	.	10	12
	44	30,000	,00	.	.	10	11
	45	30,000	,00	.	.	10	10
	46	30,000	,00	.	.	10	9
	47	30,000	,00	.	.	10	8
	48	30,000	,00	.	.	10	7
	49	30,000	,00	.	.	10	6
	50	30,000	,00	.	.	10	5
	51	30,000	,00	.	.	10	4
	52	30,000	,00	.	.	10	3
	53	30,000	,00	.	.	10	2
	54	30,000	,00	.	.	10	1
	55	30,000	,00	.	.	10	0
1,0	1	5,000	1,00	,857	,132	1	6
	2	30,000	,00	.	.	1	5
	3	30,000	,00	.	.	1	4
	4	30,000	,00	.	.	1	3
	5	30,000	,00	.	.	1	2
	6	30,000	,00	.	.	1	1
	7	30,000	,00	.	.	1	0

Means and Medians for Survival Time

Central Venous Catheter	Mean[a]				Median		
			95% Confidence Interval				95% …
	Estimate	Std. Error	Lower Bound	Upper Bound	Estimate	Std. Error	Lower Bound
,0	27,073	,963	25,184	28,961	.	.	.
1,0	26,429	3,307	19,948	32,909	.	.	.
Overall	27,000	,933	25,171	28,829	.	.	.

Means and Medians for Survival Time

Central Venous Catheter	Median
	95% …
	Upper Bound
,0	.
1,0	.
Overall	.

a. Estimation is limited to the largest survival time if it is censored.

Overall Comparisons

	Chi-Square	df	Sig.
Log Rank (Mantel-Cox)	,034	1	,853

Test of equality of survival distributions for the different levels of Central Venous Catheter

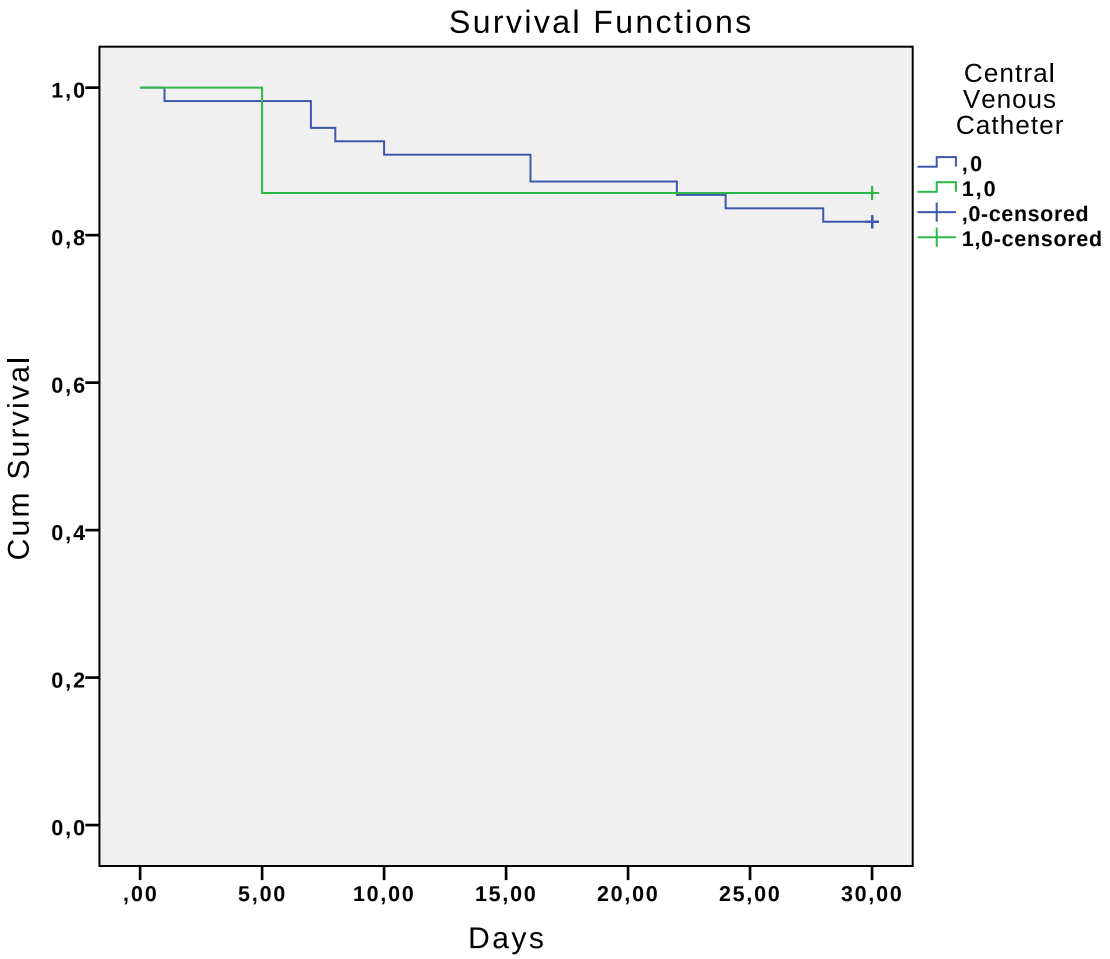

MECHANICAL VENTILATION (0=NO, 1=YES)

Case Processing Summary

Mechanical Ventilation	Total N	N of Events	Censored	
			N	Percent
,0	57	10	47	82,5%
1,0	5	1	4	80,0%
Overall	62	11	51	82,3%

Survival Table

Mechanical Ventilation		Time	Status	Cumulative Proportion Surviving at the Time		N of Cumulative Events	N of Remaining Cases
				Estimate	Std. Error		
,0	1	1,000	1,00	,982	,017	1	56
	2	7,000	1,00	.	.	2	55
	3	7,000	1,00	,947	,030	3	54
	4	8,000	1,00	,930	,034	4	53
	5	10,000	1,00	,912	,037	5	52
	6	16,000	1,00	.	.	6	51
	7	16,000	1,00	,877	,043	7	50
	8	22,000	1,00	,860	,046	8	49
	9	24,000	1,00	,842	,048	9	48
	10	28,000	1,00	,825	,050	10	47
	11	30,000	,00	.	.	10	46
	12	30,000	,00	.	.	10	45
	13	30,000	,00	.	.	10	44
	14	30,000	,00	.	.	10	43
	15	30,000	,00	.	.	10	42
	16	30,000	,00	.	.	10	41
	17	30,000	,00	.	.	10	40
	18	30,000	,00	.	.	10	39
	19	30,000	,00	.	.	10	38
	20	30,000	,00	.	.	10	37
	21	30,000	,00	.	.	10	36
	22	30,000	,00	.	.	10	35
	23	30,000	,00	.	.	10	34
	24	30,000	,00	.	.	10	33
	25	30,000	,00	.	.	10	32
	26	30,000	,00	.	.	10	31
	27	30,000	,00	.	.	10	30
	28	30,000	,00	.	.	10	29
	29	30,000	,00	.	.	10	28
	30	30,000	,00	.	.	10	27
	31	30,000	,00	.	.	10	26
	32	30,000	,00	.	.	10	25
	33	30,000	,00	.	.	10	24
	34	30,000	,00	.	.	10	23
	35	30,000	,00	.	.	10	22
	36	30,000	,00	.	.	10	21
	37	30,000	,00	.	.	10	20
	38	30,000	,00	.	.	10	19
	39	30,000	,00	.	.	10	18
	40	30,000	,00	.	.	10	17
	41	30,000	,00	.	.	10	16
	42	30,000	,00	.	.	10	15
	43	30,000	,00	.	.	10	14
	44	30,000	,00	.	.	10	13
	45	30,000	,00	.	.	10	12
	46	30,000	,00	.	.	10	11
	47	30,000	,00	.	.	10	10
	48	30,000	,00	.	.	10	9
	49	30,000	,00	.	.	10	8

Survival Table

Mechanical Ventilation		Time	Status	Cumulative Proportion Surviving at the Time		N of Cumulative Events	N of Remaining Cases
				Estimate	Std. Error		
	50	30,000	,00	.	.	10	7
	51	30,000	,00	.	.	10	6
	52	30,000	,00	.	.	10	5
	53	30,000	,00	.	.	10	4
	54	30,000	,00	.	.	10	3
	55	30,000	,00	.	.	10	2
	56	30,000	,00	.	.	10	1
	57	30,000	,00	.	.	10	0
1,0	1	5,000	1,00	,800	,179	1	4
	2	30,000	,00	.	.	1	3
	3	30,000	,00	.	.	1	2
	4	30,000	,00	.	.	1	1
	5	30,000	,00	.	.	1	0

Means and Medians for Survival Time

Mechanical Ventilation	Mean[a]				Median		
			95% Confidence Interval				95% …
	Estimate	Std. Error	Lower Bound	Upper Bound	Estimate	Std. Error	Lower Bound
,0	27,175	,932	25,348	29,003	.	.	.
1,0	25,000	4,472	16,235	33,765	.	.	.
Overall	27,000	,933	25,171	28,829	.	.	.

Means and Medians for Survival Time

Mechanical Ventilation	Median
	95% …
	Upper Bound
,0	.
1,0	.
Overall	.

a. Estimation is limited to the largest survival time if it is censored.

Overall Comparisons

	Chi-Square	df	Sig.
Log Rank (Mantel-Cox)	,052	1	,820

Test of equality of survival distributions for the different levels of Mechanical Ventilation

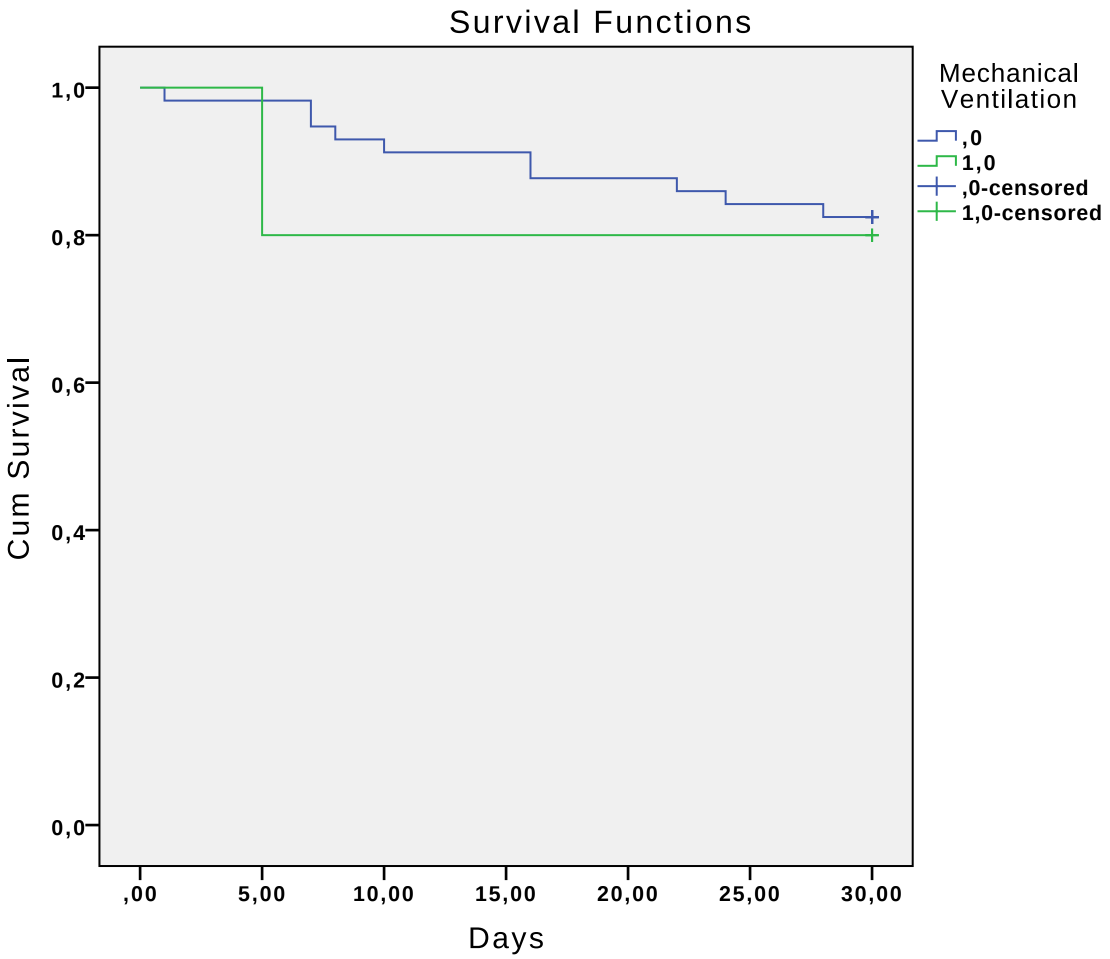

URINARY CATHETER (0=NO, 1=YES)

Case Processing Summary

Urinary Catheter	Total N	N of Events	Censored	
			N	Percent
,0	19	3	16	84,2%
1,0	43	8	35	81,4%
Overall	62	11	51	82,3%

Survival Table

Urinary Catheter		Time	Status	Cumulative Proportion Surviving at the Time		N of Cumulative Events	N of Remaining Cases
				Estimate	Std. Error		
,0	1	7,000	1,00	,947	,051	1	18
	2	10,000	1,00	,895	,070	2	17
	3	24,000	1,00	,842	,084	3	16
	4	30,000	,00	.	.	3	15
	5	30,000	,00	.	.	3	14
	6	30,000	,00	.	.	3	13
	7	30,000	,00	.	.	3	12
	8	30,000	,00	.	.	3	11
	9	30,000	,00	.	.	3	10
	10	30,000	,00	.	.	3	9
	11	30,000	,00	.	.	3	8
	12	30,000	,00	.	.	3	7
	13	30,000	,00	.	.	3	6
	14	30,000	,00	.	.	3	5
	15	30,000	,00	.	.	3	4
	16	30,000	,00	.	.	3	3
	17	30,000	,00	.	.	3	2
	18	30,000	,00	.	.	3	1
	19	30,000	,00	.	.	3	0
1,0	1	1,000	1,00	,977	,023	1	42
	2	5,000	1,00	,953	,032	2	41
	3	7,000	1,00	,930	,039	3	40
	4	8,000	1,00	,907	,044	4	39
	5	16,000	1,00	.	.	5	38
	6	16,000	1,00	,860	,053	6	37
	7	22,000	1,00	,837	,056	7	36
	8	28,000	1,00	,814	,059	8	35
	9	30,000	,00	.	.	8	34
	10	30,000	,00	.	.	8	33
	11	30,000	,00	.	.	8	32
	12	30,000	,00	.	.	8	31
	13	30,000	,00	.	.	8	30
	14	30,000	,00	.	.	8	29
	15	30,000	,00	.	.	8	28
	16	30,000	,00	.	.	8	27
	17	30,000	,00	.	.	8	26
	18	30,000	,00	.	.	8	25
	19	30,000	,00	.	.	8	24
	20	30,000	,00	.	.	8	23
	21	30,000	,00	.	.	8	22
	22	30,000	,00	.	.	8	21
	23	30,000	,00	.	.	8	20
	24	30,000	,00	.	.	8	19
	25	30,000	,00	.	.	8	18
	26	30,000	,00	.	.	8	17
	27	30,000	,00	.	.	8	16
	28	30,000	,00	.	.	8	15
	29	30,000	,00	.	.	8	14
	30	30,000	,00	.	.	8	13

Survival Table

Urinary Catheter	Time	Status	Cumulative Proportion Surviving at the Time		N of Cumulative Events	N of Remaining Cases
			Estimate	Std. Error		
31	30,000	,00	.	.	8	12
32	30,000	,00	.	.	8	11
33	30,000	,00	.	.	8	10
34	30,000	,00	.	.	8	9
35	30,000	,00	.	.	8	8
36	30,000	,00	.	.	8	7
37	30,000	,00	.	.	8	6
38	30,000	,00	.	.	8	5
39	30,000	,00	.	.	8	4
40	30,000	,00	.	.	8	3
41	30,000	,00	.	.	8	2
42	30,000	,00	.	.	8	1
43	30,000	,00	.	.	8	0

Means and Medians for Survival Time

Urinary Catheter	Mean[a]				Median		
	Estimate	Std. Error	95% Confidence Interval		Estimate	Std. Error	95% …
			Lower Bound	Upper Bound			Lower Bound
,0	27,421	1,524	24,434	30,408	.	.	.
1,0	26,814	1,163	24,534	29,094	.	.	.
Overall	27,000	,933	25,171	28,829	.	.	.

Means and Medians for Survival Time

Urinary Catheter	Median
	95% …
	Upper Bound
,0	.
1,0	.
Overall	.

a. Estimation is limited to the largest survival time if it is censored.

Overall Comparisons

	Chi-Square	df	Sig.
Log Rank (Mantel-Cox)	,078	1	,780

Test of equality of survival distributions for the different levels of Urinary Catheter

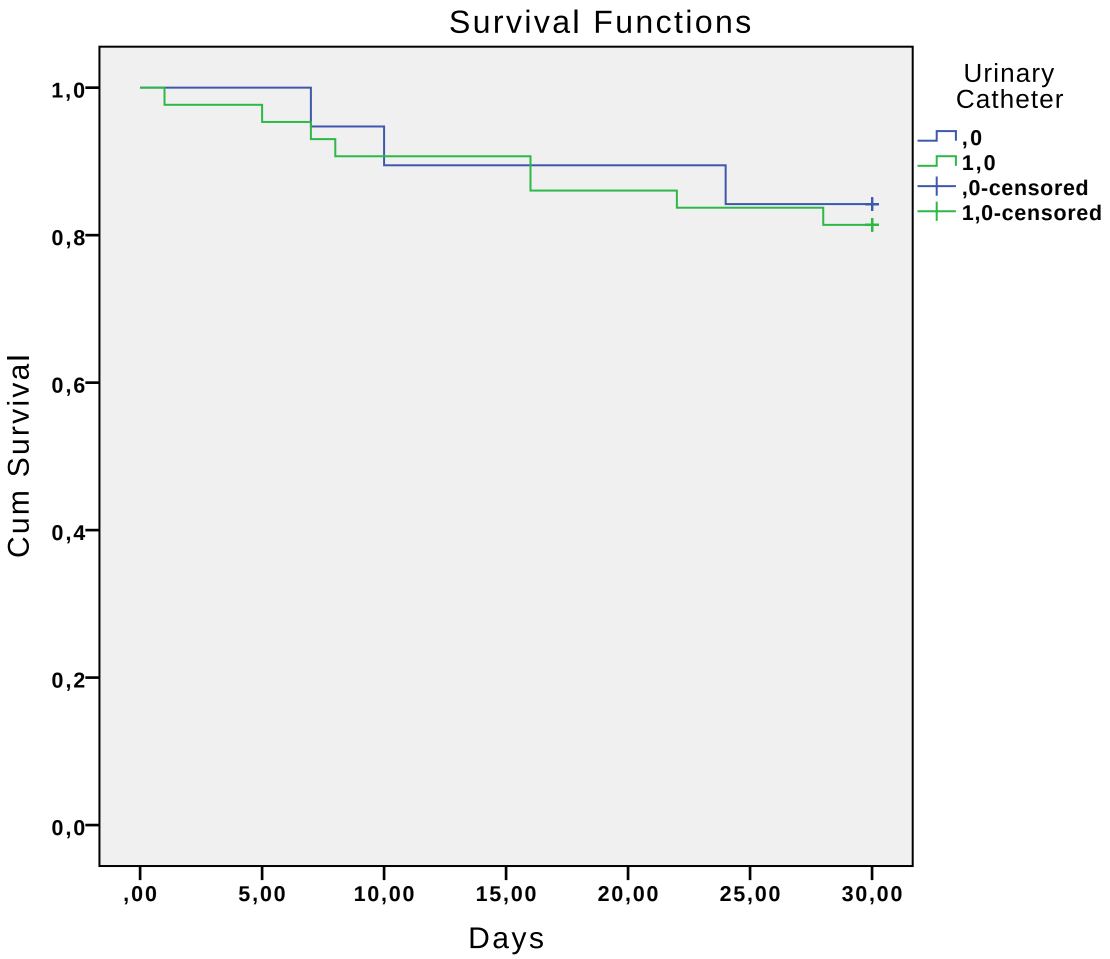

GASTROSTOMY (0=NO, 1=YES)

Case Processing Summary

Gastrostomy	Total N	N of Events	Censored	
			N	Percent
,0	56	10	46	82,1%
1,0	6	1	5	83,3%
Overall	62	11	51	82,3%

Survival Table

Gastrostomy		Time	Status	Cumulative Proportion Surviving at the Time		N of Cumulative Events	N of Remaining Cases
				Estimate	Std. Error		
,0	1	1,000	1,00	,982	,018	1	55
	2	7,000	1,00	.	.	2	54
	3	7,000	1,00	,946	,030	3	53
	4	8,000	1,00	,929	,034	4	52
	5	10,000	1,00	,911	,038	5	51
	6	16,000	1,00	.	.	6	50
	7	16,000	1,00	,875	,044	7	49
	8	22,000	1,00	,857	,047	8	48
	9	24,000	1,00	,839	,049	9	47
	10	28,000	1,00	,821	,051	10	46
	11	30,000	,00	.	.	10	45
	12	30,000	,00	.	.	10	44
	13	30,000	,00	.	.	10	43
	14	30,000	,00	.	.	10	42
	15	30,000	,00	.	.	10	41
	16	30,000	,00	.	.	10	40
	17	30,000	,00	.	.	10	39
	18	30,000	,00	.	.	10	38
	19	30,000	,00	.	.	10	37
	20	30,000	,00	.	.	10	36
	21	30,000	,00	.	.	10	35
	22	30,000	,00	.	.	10	34
	23	30,000	,00	.	.	10	33
	24	30,000	,00	.	.	10	32
	25	30,000	,00	.	.	10	31
	26	30,000	,00	.	.	10	30
	27	30,000	,00	.	.	10	29
	28	30,000	,00	.	.	10	28
	29	30,000	,00	.	.	10	27
	30	30,000	,00	.	.	10	26
	31	30,000	,00	.	.	10	25
	32	30,000	,00	.	.	10	24
	33	30,000	,00	.	.	10	23
	34	30,000	,00	.	.	10	22
	35	30,000	,00	.	.	10	21
	36	30,000	,00	.	.	10	20
	37	30,000	,00	.	.	10	19
	38	30,000	,00	.	.	10	18
	39	30,000	,00	.	.	10	17
	40	30,000	,00	.	.	10	16
	41	30,000	,00	.	.	10	15
	42	30,000	,00	.	.	10	14
	43	30,000	,00	.	.	10	13
	44	30,000	,00	.	.	10	12
	45	30,000	,00	.	.	10	11
	46	30,000	,00	.	.	10	10
	47	30,000	,00	.	.	10	9
	48	30,000	,00	.	.	10	8
	49	30,000	,00	.	.	10	7

Survival Table

Gastrostomy		Time	Status	Cumulative Proportion Surviving at the Time		N of Cumulative Events	N of Remaining Cases
				Estimate	Std. Error		
	50	30,000	,00	.	.	10	6
	51	30,000	,00	.	.	10	5
	52	30,000	,00	.	.	10	4
	53	30,000	,00	.	.	10	3
	54	30,000	,00	.	.	10	2
	55	30,000	,00	.	.	10	1
	56	30,000	,00	.	.	10	0
1,0	1	5,000	1,00	,833	,152	1	5
	2	30,000	,00	.	.	1	4
	3	30,000	,00	.	.	1	3
	4	30,000	,00	.	.	1	2
	5	30,000	,00	.	.	1	1
	6	30,000	,00	.	.	1	0

Means and Medians for Survival Time

Gastrostomy	Mean[a]				Median		
			95% Confidence Interval				95% …
	Estimate	Std. Error	Lower Bound	Upper Bound	Estimate	Std. Error	Lower Bound
,0	27,125	,948	25,268	28,982	.	.	.
1,0	25,833	3,804	18,378	33,288	.	.	.
Overall	27,000	,933	25,171	28,829	.	.	.

Means and Medians for Survival Time

Gastrostomy	Median
	95% …
	Upper Bound
,0	.
1,0	.
Overall	.

a. Estimation is limited to the largest survival time if it is censored.

Overall Comparisons

	Chi-Square	df	Sig.
Log Rank (Mantel-Cox)	,000	1	,998

Test of equality of survival distributions for the different levels of Gastrostomy

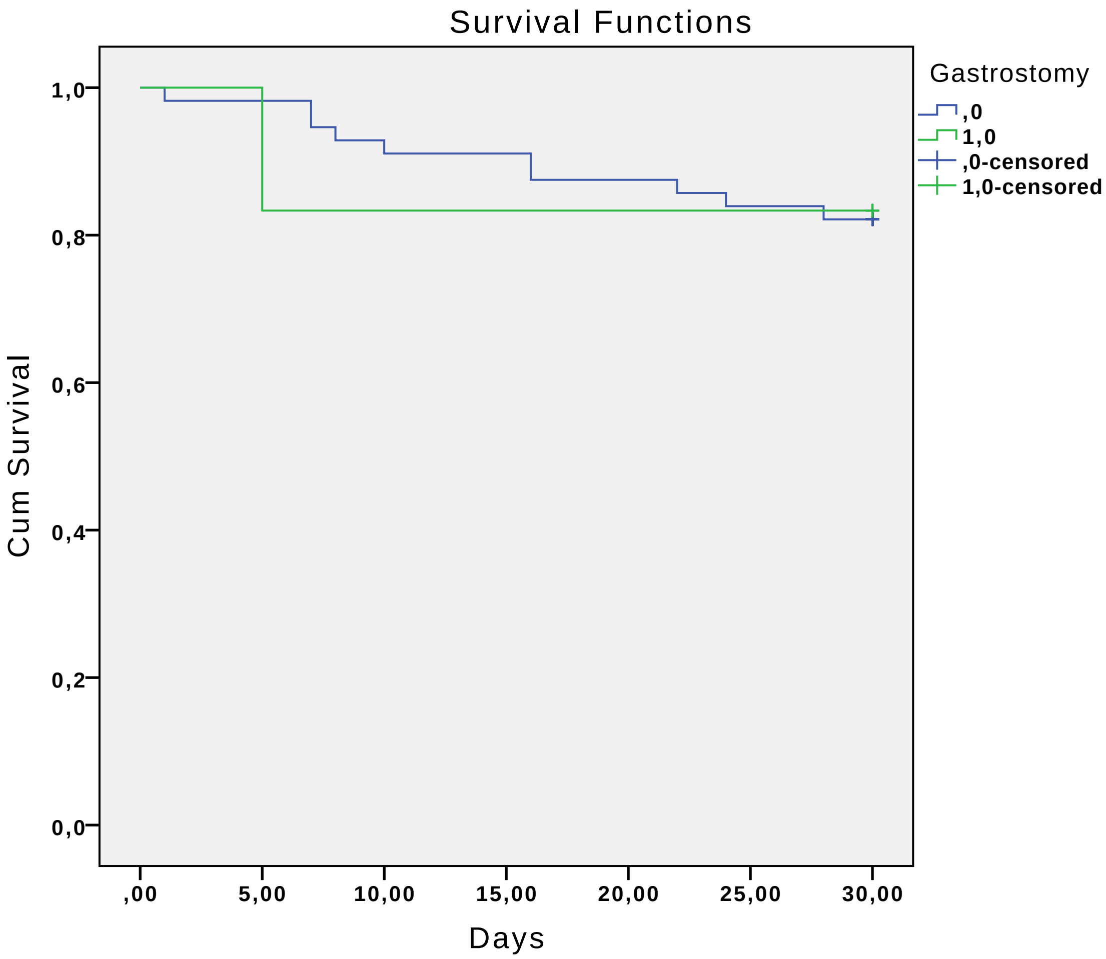

COGNITIVE IMPAIRMENT (0=NO, 1=YES)

Case Processing Summary

Cognitive Impairment	Total N	N of Events	Censored	
			N	Percent
,0	39	5	34	87,2%
1,0	23	6	17	73,9%
Overall	62	11	51	82,3%

Survival Table

Cognitive Impairment		Time	Status	Cumulative Proportion Surviving at the Time		N of Cumulative Events	N of Remaining Cases
				Estimate	Std. Error		
,0	1	1,000	1,00	,974	,025	1	38
	2	7,000	1,00	.	.	2	37
	3	7,000	1,00	,923	,043	3	36
	4	10,000	1,00	,897	,049	4	35
	5	16,000	1,00	,872	,054	5	34
	6	30,000	,00	.	.	5	33
	7	30,000	,00	.	.	5	32
	8	30,000	,00	.	.	5	31
	9	30,000	,00	.	.	5	30
	10	30,000	,00	.	.	5	29
	11	30,000	,00	.	.	5	28
	12	30,000	,00	.	.	5	27
	13	30,000	,00	.	.	5	26
	14	30,000	,00	.	.	5	25
	15	30,000	,00	.	.	5	24
	16	30,000	,00	.	.	5	23
	17	30,000	,00	.	.	5	22
	18	30,000	,00	.	.	5	21
	19	30,000	,00	.	.	5	20
	20	30,000	,00	.	.	5	19
	21	30,000	,00	.	.	5	18
	22	30,000	,00	.	.	5	17
	23	30,000	,00	.	.	5	16
	24	30,000	,00	.	.	5	15
	25	30,000	,00	.	.	5	14
	26	30,000	,00	.	.	5	13
	27	30,000	,00	.	.	5	12
	28	30,000	,00	.	.	5	11
	29	30,000	,00	.	.	5	10
	30	30,000	,00	.	.	5	9
	31	30,000	,00	.	.	5	8
	32	30,000	,00	.	.	5	7
	33	30,000	,00	.	.	5	6
	34	30,000	,00	.	.	5	5
	35	30,000	,00	.	.	5	4
	36	30,000	,00	.	.	5	3
	37	30,000	,00	.	.	5	2
	38	30,000	,00	.	.	5	1
	39	30,000	,00	.	.	5	0
1,0	1	5,000	1,00	,957	,043	1	22
	2	8,000	1,00	,913	,059	2	21
	3	16,000	1,00	,870	,070	3	20
	4	22,000	1,00	,826	,079	4	19
	5	24,000	1,00	,783	,086	5	18
	6	28,000	1,00	,739	,092	6	17
	7	30,000	,00	.	.	6	16
	8	30,000	,00	.	.	6	15
	9	30,000	,00	.	.	6	14
	10	30,000	,00	.	.	6	13

Survival Table

Cognitive Impairment	Time	Status	Cumulative Proportion Surviving at the Time		N of Cumulative Events	N of Remaining Cases
			Estimate	Std. Error		
11	30,000	,00	.	.	6	12
12	30,000	,00	.	.	6	11
13	30,000	,00	.	.	6	10
14	30,000	,00	.	.	6	9
15	30,000	,00	.	.	6	8
16	30,000	,00	.	.	6	7
17	30,000	,00	.	.	6	6
18	30,000	,00	.	.	6	5
19	30,000	,00	.	.	6	4
20	30,000	,00	.	.	6	3
21	30,000	,00	.	.	6	2
22	30,000	,00	.	.	6	1
23	30,000	,00	.	.	6	0

Means and Medians for Survival Time

Cognitive Impairment	Mean[a]				Median		
	Estimate	Std. Error	95% Confidence Interval		Estimate	Std. Error	95% …
			Lower Bound	Upper Bound			Lower Bound
,0	27,205	1,200	24,853	29,557	.	.	.
1,0	26,652	1,475	23,761	29,544	.	.	.
Overall	27,000	,933	25,171	28,829	.	.	.

Means and Medians for Survival Time

Cognitive Impairment	Median
	95% …
	Upper Bound
,0	.
1,0	.
Overall	.

a. Estimation is limited to the largest survival time if it is censored.

Overall Comparisons

	Chi-Square	df	Sig.
Log Rank (Mantel-Cox)	1,489	1	,222

Test of equality of survival distributions for the different levels of Cognitive Impairment

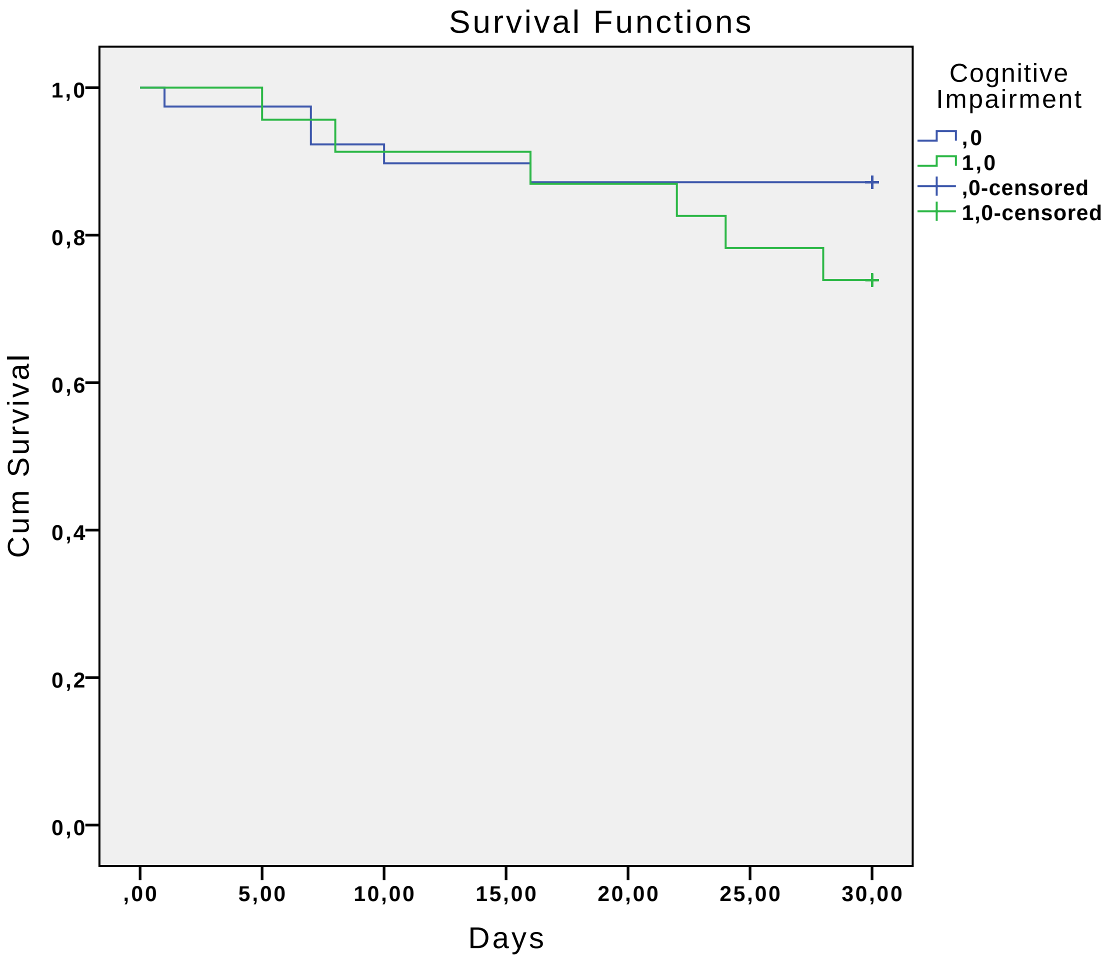

SOLID ORGAN CANCER (0=NO, 1=YES)

Case Processing Summary

Solid Organ Cancer	Total N	N of Events	Censored	
			N	Percent
,0	48	8	40	83,3%
1,0	14	3	11	78,6%
Overall	62	11	51	82,3%

Survival Table

Solid Organ Cancer		Time	Status	Cumulative Proportion Surviving at the Time		N of Cumulative Events	N of Remaining Cases
				Estimate	Std. Error		
,0	1	1,000	1,00	,979	,021	1	47
	2	5,000	1,00	,958	,029	2	46
	3	7,000	1,00	.	.	3	45
	4	7,000	1,00	,917	,040	4	44
	5	8,000	1,00	,896	,044	5	43
	6	16,000	1,00	.	.	6	42
	7	16,000	1,00	,854	,051	7	41
	8	28,000	1,00	,833	,054	8	40
	9	30,000	,00	.	.	8	39
	10	30,000	,00	.	.	8	38
	11	30,000	,00	.	.	8	37
	12	30,000	,00	.	.	8	36
	13	30,000	,00	.	.	8	35
	14	30,000	,00	.	.	8	34
	15	30,000	,00	.	.	8	33
	16	30,000	,00	.	.	8	32
	17	30,000	,00	.	.	8	31
	18	30,000	,00	.	.	8	30
	19	30,000	,00	.	.	8	29
	20	30,000	,00	.	.	8	28
	21	30,000	,00	.	.	8	27
	22	30,000	,00	.	.	8	26
	23	30,000	,00	.	.	8	25
	24	30,000	,00	.	.	8	24
	25	30,000	,00	.	.	8	23
	26	30,000	,00	.	.	8	22
	27	30,000	,00	.	.	8	21
	28	30,000	,00	.	.	8	20
	29	30,000	,00	.	.	8	19
	30	30,000	,00	.	.	8	18
	31	30,000	,00	.	.	8	17
	32	30,000	,00	.	.	8	16
	33	30,000	,00	.	.	8	15
	34	30,000	,00	.	.	8	14
	35	30,000	,00	.	.	8	13
	36	30,000	,00	.	.	8	12
	37	30,000	,00	.	.	8	11
	38	30,000	,00	.	.	8	10
	39	30,000	,00	.	.	8	9
	40	30,000	,00	.	.	8	8
	41	30,000	,00	.	.	8	7
	42	30,000	,00	.	.	8	6
	43	30,000	,00	.	.	8	5
	44	30,000	,00	.	.	8	4
	45	30,000	,00	.	.	8	3
	46	30,000	,00	.	.	8	2
	47	30,000	,00	.	.	8	1
	48	30,000	,00	.	.	8	0

Survival Table

Solid Organ Cancer		Time	Status	Cumulative Proportion Surviving at the Time		N of Cumulative Events	N of Remaining Cases
				Estimate	Std. Error		
1,0	1	10,000	1,00	,929	,069	1	13
	2	22,000	1,00	,857	,094	2	12
	3	24,000	1,00	,786	,110	3	11
	4	30,000	,00	.	.	3	10
	5	30,000	,00	.	.	3	9
	6	30,000	,00	.	.	3	8
	7	30,000	,00	.	.	3	7
	8	30,000	,00	.	.	3	6
	9	30,000	,00	.	.	3	5
	10	30,000	,00	.	.	3	4
	11	30,000	,00	.	.	3	3
	12	30,000	,00	.	.	3	2
	13	30,000	,00	.	.	3	1
	14	30,000	,00	.	.	3	0

Means and Medians for Survival Time

Solid Organ Cancer	Mean[a]				Median		
			95% Confidence Interval				95% ...
	Estimate	Std. Error	Lower Bound	Upper Bound	Estimate	Std. Error	Lower Bound
,0	26,833	1,126	24,626	29,041	.	.	.
1,0	27,571	1,459	24,711	30,432	.	.	.
Overall	27,000	,933	25,171	28,829	.	.	.

Means and Medians for Survival Time

Solid Organ Cancer	Median
	95% ...
	Upper Bound
,0	.
1,0	.
Overall	.

a. Estimation is limited to the largest survival time if it is censored.

Overall Comparisons

	Chi-Square	df	Sig.
Log Rank (Mantel-Cox)	,103	1	,748

Test of equality of survival distributions for the different levels of Solid Organ Cancer

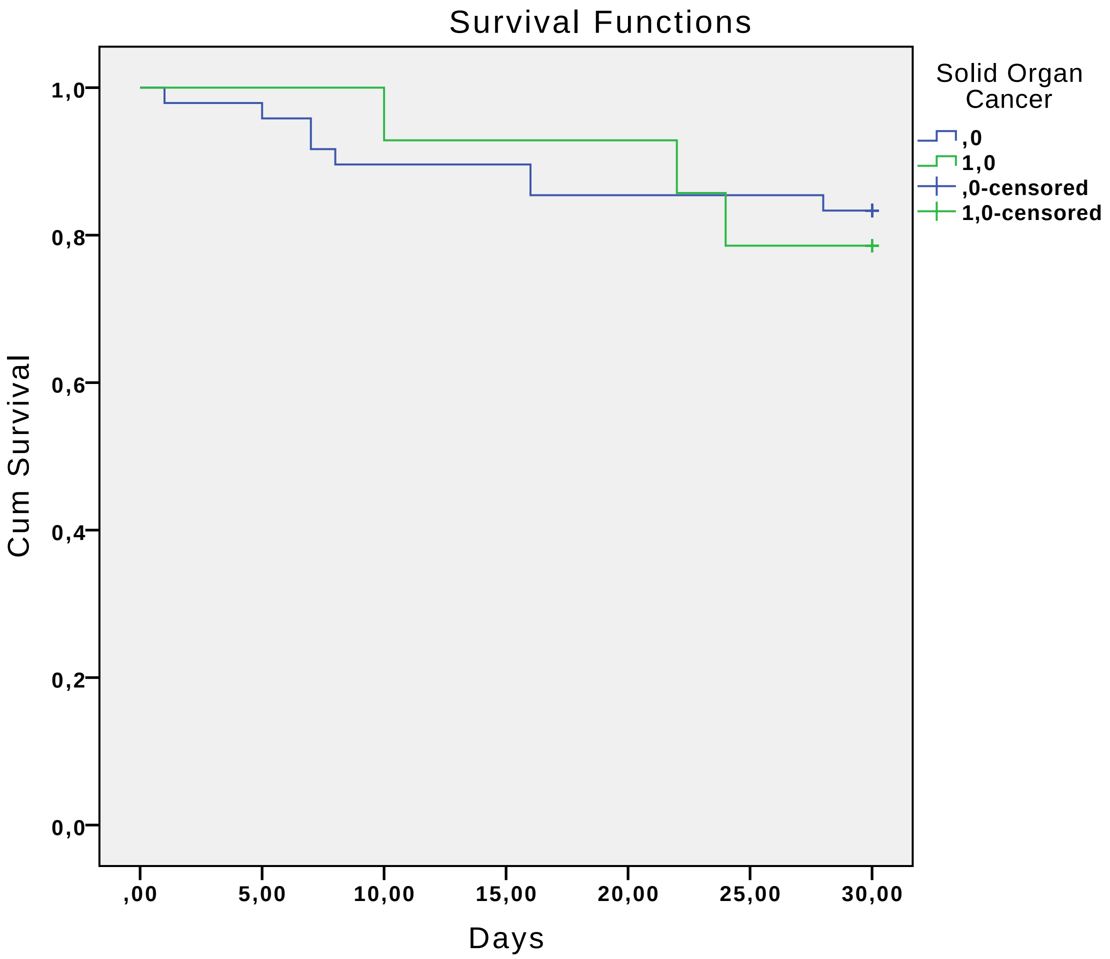

LEUKEMIA (0=NO, 1=YES)

Warnings

No statistics are computed because all cases are censored.

Case Processing Summary

Leukemia	Total N	N of Events	Censored	
			N	Percent
,0	61	11	50	82,0%
1,0	1	0	1	100,0%
Overall	62	11	51	82,3%

Survival Table

Leukemia		Time	Status	Cumulative Proportion Surviving at the Time		N of Cumulative Events	N of Remaining Cases
				Estimate	Std. Error		
,0	1	1,000	1,00	,984	,016	1	60
	2	5,000	1,00	,967	,023	2	59
	3	7,000	1,00	.	.	3	58
	4	7,000	1,00	,934	,032	4	57
	5	8,000	1,00	,918	,035	5	56
	6	10,000	1,00	,902	,038	6	55
	7	16,000	1,00	.	.	7	54
	8	16,000	1,00	,869	,043	8	53
	9	22,000	1,00	,852	,045	9	52
	10	24,000	1,00	,836	,047	10	51
	11	28,000	1,00	,820	,049	11	50
	12	30,000	,00	.	.	11	49
	13	30,000	,00	.	.	11	48
	14	30,000	,00	.	.	11	47
	15	30,000	,00	.	.	11	46
	16	30,000	,00	.	.	11	45
	17	30,000	,00	.	.	11	44
	18	30,000	,00	.	.	11	43
	19	30,000	,00	.	.	11	42
	20	30,000	,00	.	.	11	41
	21	30,000	,00	.	.	11	40
	22	30,000	,00	.	.	11	39
	23	30,000	,00	.	.	11	38
	24	30,000	,00	.	.	11	37
	25	30,000	,00	.	.	11	36
	26	30,000	,00	.	.	11	35
	27	30,000	,00	.	.	11	34
	28	30,000	,00	.	.	11	33
	29	30,000	,00	.	.	11	32
	30	30,000	,00	.	.	11	31
	31	30,000	,00	.	.	11	30
	32	30,000	,00	.	.	11	29
	33	30,000	,00	.	.	11	28
	34	30,000	,00	.	.	11	27
	35	30,000	,00	.	.	11	26
	36	30,000	,00	.	.	11	25
	37	30,000	,00	.	.	11	24
	38	30,000	,00	.	.	11	23
	39	30,000	,00	.	.	11	22
	40	30,000	,00	.	.	11	21
	41	30,000	,00	.	.	11	20
	42	30,000	,00	.	.	11	19
	43	30,000	,00	.	.	11	18
	44	30,000	,00	.	.	11	17
	45	30,000	,00	.	.	11	16
	46	30,000	,00	.	.	11	15
	47	30,000	,00	.	.	11	14
	48	30,000	,00	.	.	11	13
	49	30,000	,00	.	.	11	12

Survival Table

Leukemia	Time	Status	Cumulative Proportion Surviving at the Time		N of Cumulative Events	N of Remaining Cases
			Estimate	Std. Error		
50	30,000	,00	.	.	11	11
51	30,000	,00	.	.	11	10
52	30,000	,00	.	.	11	9
53	30,000	,00	.	.	11	8
54	30,000	,00	.	.	11	7
55	30,000	,00	.	.	11	6
56	30,000	,00	.	.	11	5
57	30,000	,00	.	.	11	4
58	30,000	,00	.	.	11	3
59	30,000	,00	.	.	11	2
60	30,000	,00	.	.	11	1
61	30,000	,00	.	.	11	0

Overall Comparisons

	Chi-Square	df	Sig.
Log Rank (Mantel-Cox)	,198	1	,657

Test of equality of survival distributions for the different levels of Leukemia

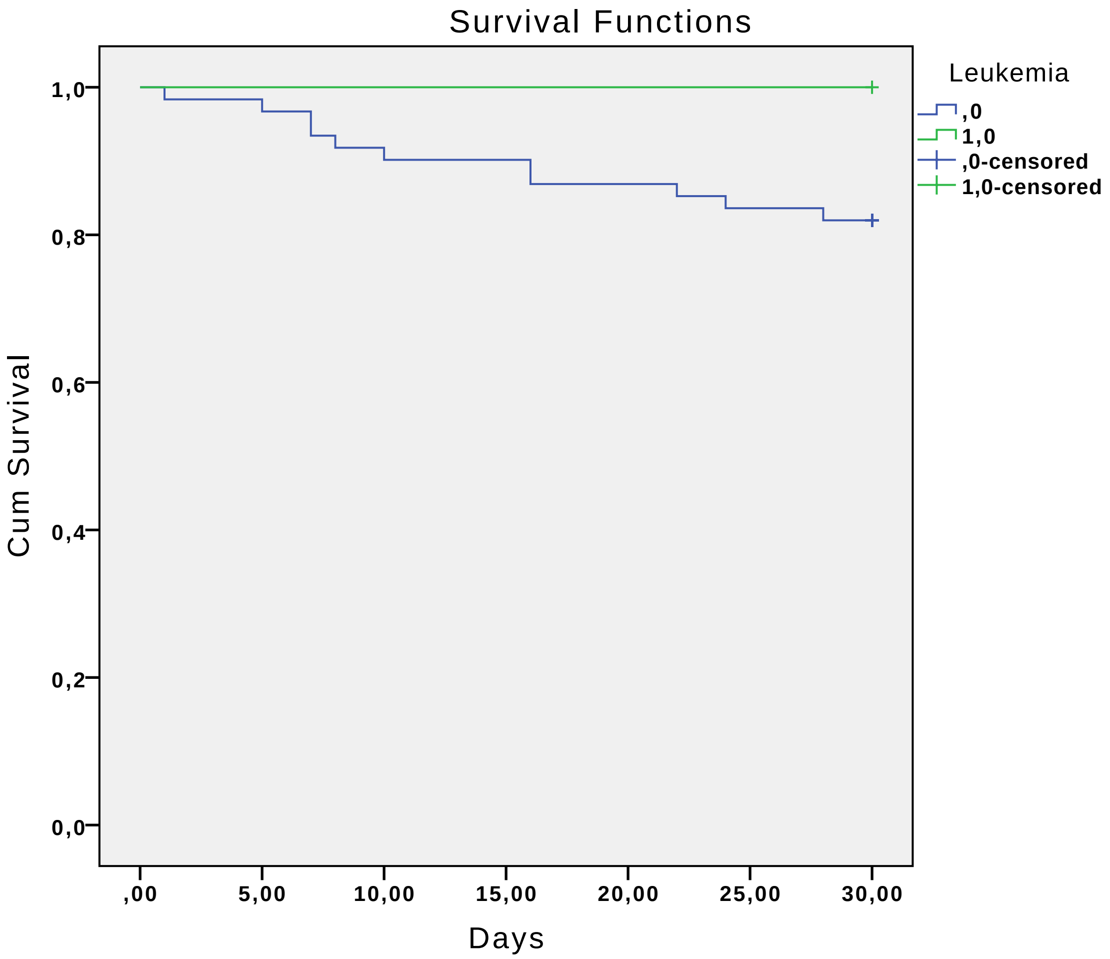

LIVER DISEASE (0=NO, 1=YES)

Case Processing Summary

Liver Disease	Total N	N of Events	Censored	
			N	Percent
,0	54	8	46	85,2%
1,0	8	3	5	62,5%
Overall	62	11	51	82,3%

Survival Table

Liver Disease		Time	Status	Cumulative Proportion Surviving at the Time		N of Cumulative Events	N of Remaining Cases
				Estimate	Std. Error		
,0	1	1,000	1,00	,981	,018	1	53
	2	7,000	1,00	,963	,026	2	52
	3	8,000	1,00	,944	,031	3	51
	4	16,000	1,00	.	.	4	50
	5	16,000	1,00	,907	,039	5	49
	6	22,000	1,00	,889	,043	6	48
	7	24,000	1,00	,870	,046	7	47
	8	28,000	1,00	,852	,048	8	46
	9	30,000	,00	.	.	8	45
	10	30,000	,00	.	.	8	44
	11	30,000	,00	.	.	8	43
	12	30,000	,00	.	.	8	42
	13	30,000	,00	.	.	8	41
	14	30,000	,00	.	.	8	40
	15	30,000	,00	.	.	8	39
	16	30,000	,00	.	.	8	38
	17	30,000	,00	.	.	8	37
	18	30,000	,00	.	.	8	36
	19	30,000	,00	.	.	8	35
	20	30,000	,00	.	.	8	34
	21	30,000	,00	.	.	8	33
	22	30,000	,00	.	.	8	32
	23	30,000	,00	.	.	8	31
	24	30,000	,00	.	.	8	30
	25	30,000	,00	.	.	8	29
	26	30,000	,00	.	.	8	28
	27	30,000	,00	.	.	8	27
	28	30,000	,00	.	.	8	26
	29	30,000	,00	.	.	8	25
	30	30,000	,00	.	.	8	24
	31	30,000	,00	.	.	8	23
	32	30,000	,00	.	.	8	22
	33	30,000	,00	.	.	8	21
	34	30,000	,00	.	.	8	20
	35	30,000	,00	.	.	8	19
	36	30,000	,00	.	.	8	18
	37	30,000	,00	.	.	8	17
	38	30,000	,00	.	.	8	16
	39	30,000	,00	.	.	8	15
	40	30,000	,00	.	.	8	14
	41	30,000	,00	.	.	8	13
	42	30,000	,00	.	.	8	12

Survival Table

Liver Disease		Time	Status	Cumulative Proportion Surviving at the Time: Estimate	Cumulative Proportion Surviving at the Time: Std. Error	N of Cumulative Events	N of Remaining Cases
	43	30,000	,00	.	.	8	11
	44	30,000	,00	.	.	8	10
	45	30,000	,00	.	.	8	9
	46	30,000	,00	.	.	8	8
	47	30,000	,00	.	.	8	7
	48	30,000	,00	.	.	8	6
	49	30,000	,00	.	.	8	5
	50	30,000	,00	.	.	8	4
	51	30,000	,00	.	.	8	3
	52	30,000	,00	.	.	8	2
	53	30,000	,00	.	.	8	1
	54	30,000	,00	.	.	8	0
1,0	1	5,000	1,00	,875	,117	1	7
	2	7,000	1,00	,750	,153	2	6
	3	10,000	1,00	,625	,171	3	5
	4	30,000	,00	.	.	3	4
	5	30,000	,00	.	.	3	3
	6	30,000	,00	.	.	3	2
	7	30,000	,00	.	.	3	1
	8	30,000	,00	.	.	3	0

Means and Medians for Survival Time

Liver Disease	Mean[a]				Median		
	Estimate	Std. Error	95% Confidence Interval: Lower Bound	95% Confidence Interval: Upper Bound	Estimate	Std. Error	95% …: Lower Bound
,0	27,815	,847	26,155	29,475	.	.	.
1,0	21,500	3,905	13,846	29,154	.	.	.
Overall	27,000	,933	25,171	28,829	.	.	.

Means and Medians for Survival Time

Liver Disease	Median: 95% …: Upper Bound
,0	.
1,0	.
Overall	.

a. Estimation is limited to the largest survival time if it is censored.

Overall Comparisons

	Chi-Square	df	Sig.
Log Rank (Mantel-Cox)	3,267	1	,071

Test of equality of survival distributions for the different levels of Liver Disease

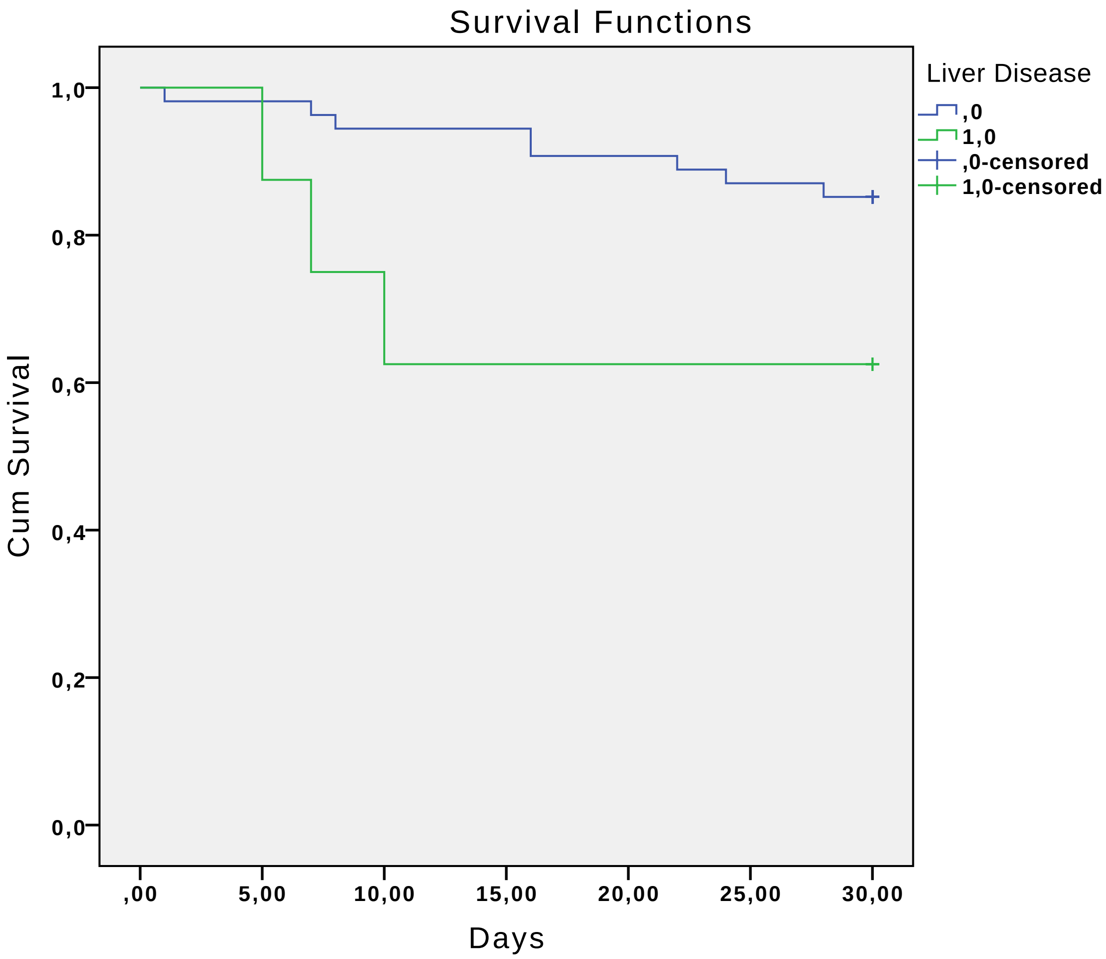

LYMPHOMA (0=NO, 1=YES)

Warnings

No comparison analysis is performed because the factor variable has only one value for every stratum.

Case Processing Summary

Lymphoma	Total N	N of Events	Censored	
			N	Percent
,0	62	11	51	82,3%
Overall	62	11	51	82,3%

Survival Table

Lymphoma		Time	Status	Cumulative Proportion Surviving at the Time		N of Cumulative Events	N of Remaining Cases
				Estimate	Std. Error		
,0	1	1,000	1,00	,984	,016	1	61
	2	5,000	1,00	,968	,022	2	60
	3	7,000	1,00	.	.	3	59
	4	7,000	1,00	,935	,031	4	58
	5	8,000	1,00	,919	,035	5	57
	6	10,000	1,00	,903	,038	6	56
	7	16,000	1,00	.	.	7	55
	8	16,000	1,00	,871	,043	8	54
	9	22,000	1,00	,855	,045	9	53
	10	24,000	1,00	,839	,047	10	52
	11	28,000	1,00	,823	,049	11	51
	12	30,000	,00	.	.	11	50
	13	30,000	,00	.	.	11	49
	14	30,000	,00	.	.	11	48
	15	30,000	,00	.	.	11	47
	16	30,000	,00	.	.	11	46
	17	30,000	,00	.	.	11	45
	18	30,000	,00	.	.	11	44
	19	30,000	,00	.	.	11	43
	20	30,000	,00	.	.	11	42
	21	30,000	,00	.	.	11	41
	22	30,000	,00	.	.	11	40
	23	30,000	,00	.	.	11	39
	24	30,000	,00	.	.	11	38
	25	30,000	,00	.	.	11	37
	26	30,000	,00	.	.	11	36
	27	30,000	,00	.	.	11	35
	28	30,000	,00	.	.	11	34
	29	30,000	,00	.	.	11	33
	30	30,000	,00	.	.	11	32
	31	30,000	,00	.	.	11	31
	32	30,000	,00	.	.	11	30
	33	30,000	,00	.	.	11	29
	34	30,000	,00	.	.	11	28
	35	30,000	,00	.	.	11	27
	36	30,000	,00	.	.	11	26
	37	30,000	,00	.	.	11	25
	38	30,000	,00	.	.	11	24
	39	30,000	,00	.	.	11	23
	40	30,000	,00	.	.	11	22
	41	30,000	,00	.	.	11	21
	42	30,000	,00	.	.	11	20
	43	30,000	,00	.	.	11	19
	44	30,000	,00	.	.	11	18
	45	30,000	,00	.	.	11	17
	46	30,000	,00	.	.	11	16
	47	30,000	,00	.	.	11	15
	48	30,000	,00	.	.	11	14
	49	30,000	,00	.	.	11	13

Survival Table

Lymphoma	Time	Status	Cumulative Proportion Surviving at the Time		N of Cumulative Events	N of Remaining Cases
			Estimate	Std. Error		
50	30,000	,00	.	.	11	12
51	30,000	,00	.	.	11	11
52	30,000	,00	.	.	11	10
53	30,000	,00	.	.	11	9
54	30,000	,00	.	.	11	8
55	30,000	,00	.	.	11	7
56	30,000	,00	.	.	11	6
57	30,000	,00	.	.	11	5
58	30,000	,00	.	.	11	4
59	30,000	,00	.	.	11	3
60	30,000	,00	.	.	11	2
61	30,000	,00	.	.	11	1
62	30,000	,00	.	.	11	0

Means and Medians for Survival Time

Lymphoma	Mean[a]				Median			
	Estimate	Std. Error	95% Confidence Interval		Estimate	Std. Error	95% Confidence Interval	
			Lower Bound	Upper Bound			Lower Bound	Upper Bound
,0	27,000	,933	25,171	28,829	.	.	.	.
Overall	27,000	,933	25,171	28,829	.	.	.	.

a. Estimation is limited to the largest survival time if it is censored.

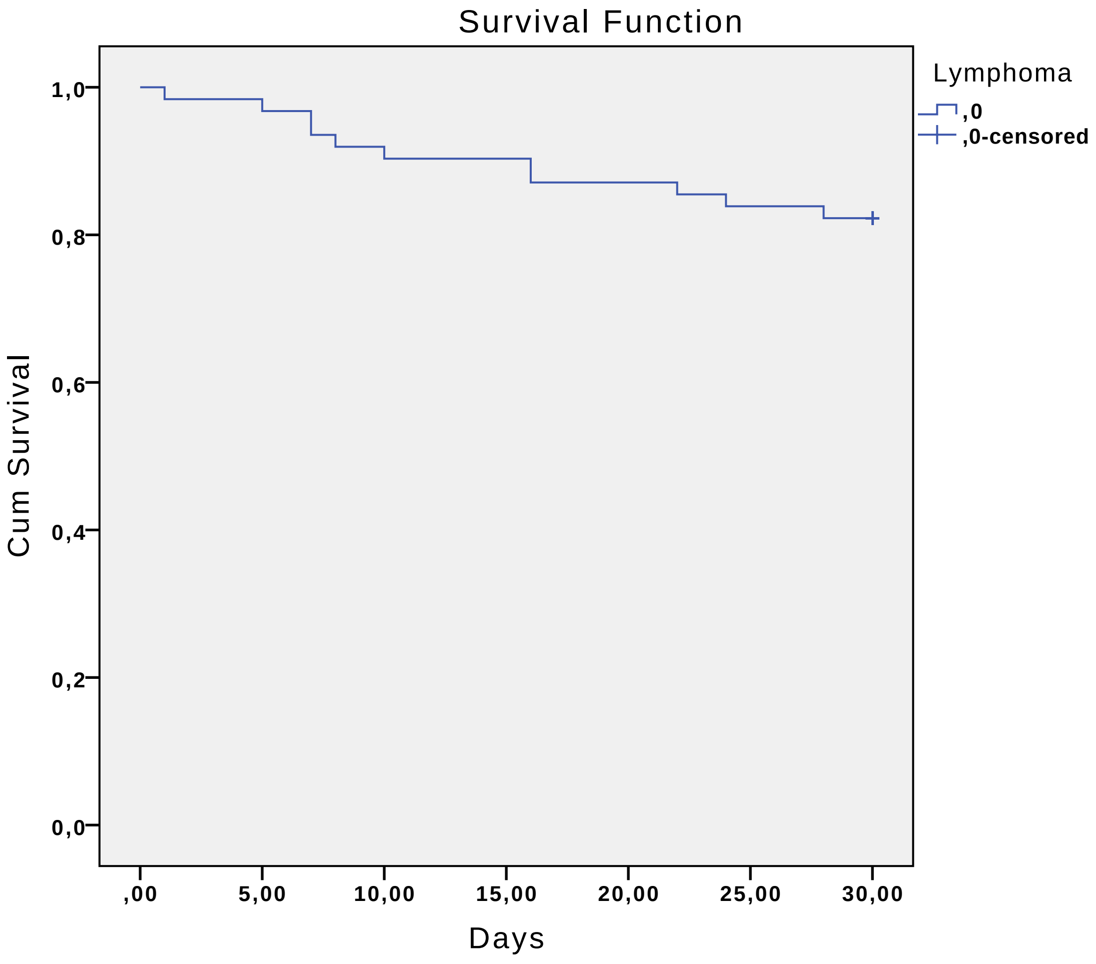

METASTASIC CANCER (0=NO, 1=YES)

Warnings

No statistics are computed because all cases are censored.

Case Processing Summary

Metastasic Cancer	Total N	N of Events	Censored	
			N	Percent
,0	58	11	47	81,0%
1,0	4	0	4	100,0%
Overall	62	11	51	82,3%

Survival Table

Metastasic Cancer		Time	Status	Cumulative Proportion Surviving at the Time		N of Cumulative Events	N of Remaining Cases
				Estimate	Std. Error		
,0	1	1,000	1,00	,983	,017	1	57
	2	5,000	1,00	,966	,024	2	56
	3	7,000	1,00	.	.	3	55
	4	7,000	1,00	,931	,033	4	54
	5	8,000	1,00	,914	,037	5	53
	6	10,000	1,00	,897	,040	6	52
	7	16,000	1,00	.	.	7	51
	8	16,000	1,00	,862	,045	8	50
	9	22,000	1,00	,845	,048	9	49
	10	24,000	1,00	,828	,050	10	48
	11	28,000	1,00	,810	,051	11	47
	12	30,000	,00	.	.	11	46
	13	30,000	,00	.	.	11	45
	14	30,000	,00	.	.	11	44
	15	30,000	,00	.	.	11	43
	16	30,000	,00	.	.	11	42
	17	30,000	,00	.	.	11	41
	18	30,000	,00	.	.	11	40
	19	30,000	,00	.	.	11	39
	20	30,000	,00	.	.	11	38
	21	30,000	,00	.	.	11	37
	22	30,000	,00	.	.	11	36
	23	30,000	,00	.	.	11	35
	24	30,000	,00	.	.	11	34
	25	30,000	,00	.	.	11	33
	26	30,000	,00	.	.	11	32
	27	30,000	,00	.	.	11	31
	28	30,000	,00	.	.	11	30
	29	30,000	,00	.	.	11	29
	30	30,000	,00	.	.	11	28
	31	30,000	,00	.	.	11	27
	32	30,000	,00	.	.	11	26
	33	30,000	,00	.	.	11	25
	34	30,000	,00	.	.	11	24
	35	30,000	,00	.	.	11	23

Survival Table

Metastasic Cancer		Time	Status	Cumulative Proportion Surviving at the Time		N of Cumulative Events	N of Remaining Cases
				Estimate	Std. Error		
	36	30,000	,00	.	.	11	22
	37	30,000	,00	.	.	11	21
	38	30,000	,00	.	.	11	20
	39	30,000	,00	.	.	11	19
	40	30,000	,00	.	.	11	18
	41	30,000	,00	.	.	11	17
	42	30,000	,00	.	.	11	16
	43	30,000	,00	.	.	11	15
	44	30,000	,00	.	.	11	14
	45	30,000	,00	.	.	11	13
	46	30,000	,00	.	.	11	12
	47	30,000	,00	.	.	11	11
	48	30,000	,00	.	.	11	10
	49	30,000	,00	.	.	11	9
	50	30,000	,00	.	.	11	8
	51	30,000	,00	.	.	11	7
	52	30,000	,00	.	.	11	6
	53	30,000	,00	.	.	11	5
	54	30,000	,00	.	.	11	4
	55	30,000	,00	.	.	11	3
	56	30,000	,00	.	.	11	2
	57	30,000	,00	.	.	11	1
	58	30,000	,00	.	.	11	0
1,0	1	30,000	,00	.	.	0	3
	2	30,000	,00	.	.	0	2
	3	30,000	,00	.	.	0	1

Overall Comparisons

	Chi-Square	df	Sig.
Log Rank (Mantel-Cox)	,836	1	,361

Test of equality of survival distributions for the different levels of Metastasic Cancer

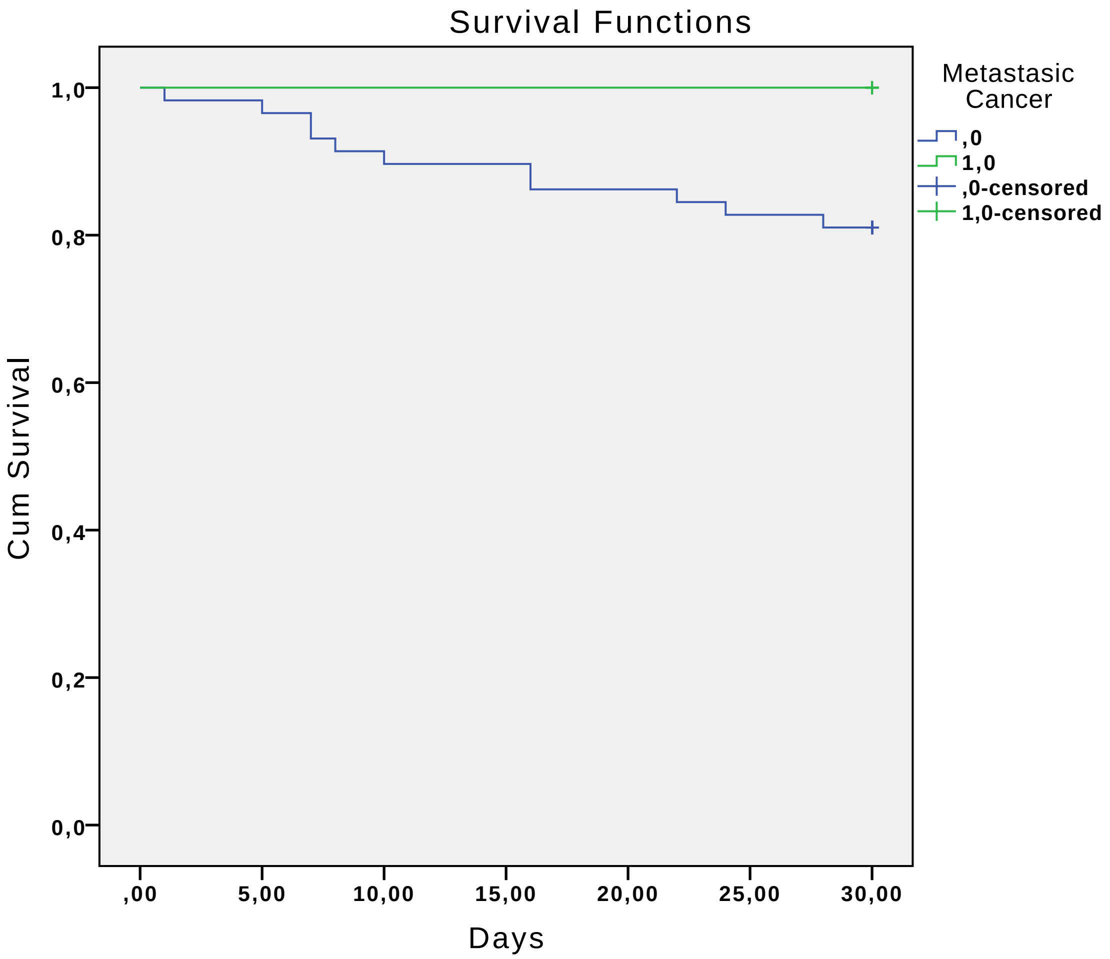

CONECTIVE TISSUE DISEASE (0=NO, 1=YES)

Case Processing Summary

Conective Tissue Disease	Total N	N of Events	Censored	
			N	Percent
,0	60	10	50	83,3%
1,0	2	1	1	50,0%
Overall	62	11	51	82,3%

Survival Table

Conective Tissue Disease		Time	Status	Cumulative Proportion Surviving at the Time		N of Cumulative Events	N of Remaining Cases
				Estimate	Std. Error		
,0	1	1,000	1,00	,983	,017	1	59
	2	5,000	1,00	,967	,023	2	58
	3	7,000	1,00	,950	,028	3	57
	4	8,000	1,00	,933	,032	4	56
	5	10,000	1,00	,917	,036	5	55
	6	16,000	1,00	.	.	6	54
	7	16,000	1,00	,883	,041	7	53
	8	22,000	1,00	,867	,044	8	52
	9	24,000	1,00	,850	,046	9	51
	10	28,000	1,00	,833	,048	10	50
	11	30,000	,00	.	.	10	49
	12	30,000	,00	.	.	10	48
	13	30,000	,00	.	.	10	47
	14	30,000	,00	.	.	10	46
	15	30,000	,00	.	.	10	45
	16	30,000	,00	.	.	10	44
	17	30,000	,00	.	.	10	43
	18	30,000	,00	.	.	10	42
	19	30,000	,00	.	.	10	41
	20	30,000	,00	.	.	10	40
	21	30,000	,00	.	.	10	39
	22	30,000	,00	.	.	10	38
	23	30,000	,00	.	.	10	37
	24	30,000	,00	.	.	10	36
	25	30,000	,00	.	.	10	35
	26	30,000	,00	.	.	10	34
	27	30,000	,00	.	.	10	33
	28	30,000	,00	.	.	10	32
	29	30,000	,00	.	.	10	31
	30	30,000	,00	.	.	10	30
	31	30,000	,00	.	.	10	29
	32	30,000	,00	.	.	10	28
	33	30,000	,00	.	.	10	27
	34	30,000	,00	.	.	10	26
	35	30,000	,00	.	.	10	25
	36	30,000	,00	.	.	10	24
	37	30,000	,00	.	.	10	23
	38	30,000	,00	.	.	10	22
	39	30,000	,00	.	.	10	21
	40	30,000	,00	.	.	10	20
	41	30,000	,00	.	.	10	19
	42	30,000	,00	.	.	10	18
	43	30,000	,00	.	.	10	17
	44	30,000	,00	.	.	10	16
	45	30,000	,00	.	.	10	15
	46	30,000	,00	.	.	10	14
	47	30,000	,00	.	.	10	13
	48	30,000	,00	.	.	10	12
	49	30,000	,00	.	.	10	11

Survival Table

Conective Tissue Disease		Time	Status	Cumulative Proportion Surviving at the Time		N of Cumulative Events	N of Remaining Cases
				Estimate	Std. Error		
	50	30,000	,00	.	.	10	10
	51	30,000	,00	.	.	10	9
	52	30,000	,00	.	.	10	8
	53	30,000	,00	.	.	10	7
	54	30,000	,00	.	.	10	6
	55	30,000	,00	.	.	10	5
	56	30,000	,00	.	.	10	4
	57	30,000	,00	.	.	10	3
	58	30,000	,00	.	.	10	2
	59	30,000	,00	.	.	10	1
	60	30,000	,00	.	.	10	0
1,0	1	7,000	1,00	,500	,354	1	1
	2	30,000	,00	.	.	1	0

Means and Medians for Survival Time

Conective Tissue Disease	Mean[a]				Median		
			95% Confidence Interval				95% …
	Estimate	Std. Error	Lower Bound	Upper Bound	Estimate	Std. Error	Lower Bound
,0	27,283	,902	25,514	29,052	.	.	.
1,0	18,500	8,132	2,562	34,438	7,000	.	.
Overall	27,000	,933	25,171	28,829	.	.	.

Means and Medians for Survival Time

Conective Tissue Disease	Median
	95% …
	Upper Bound
,0	.
1,0	.
Overall	.

a. Estimation is limited to the largest survival time if it is censored.

Overall Comparisons

	Chi-Square	df	Sig.
Log Rank (Mantel-Cox)	2,193	1	,139

Test of equality of survival distributions for the different levels of Conective Tissue Disease

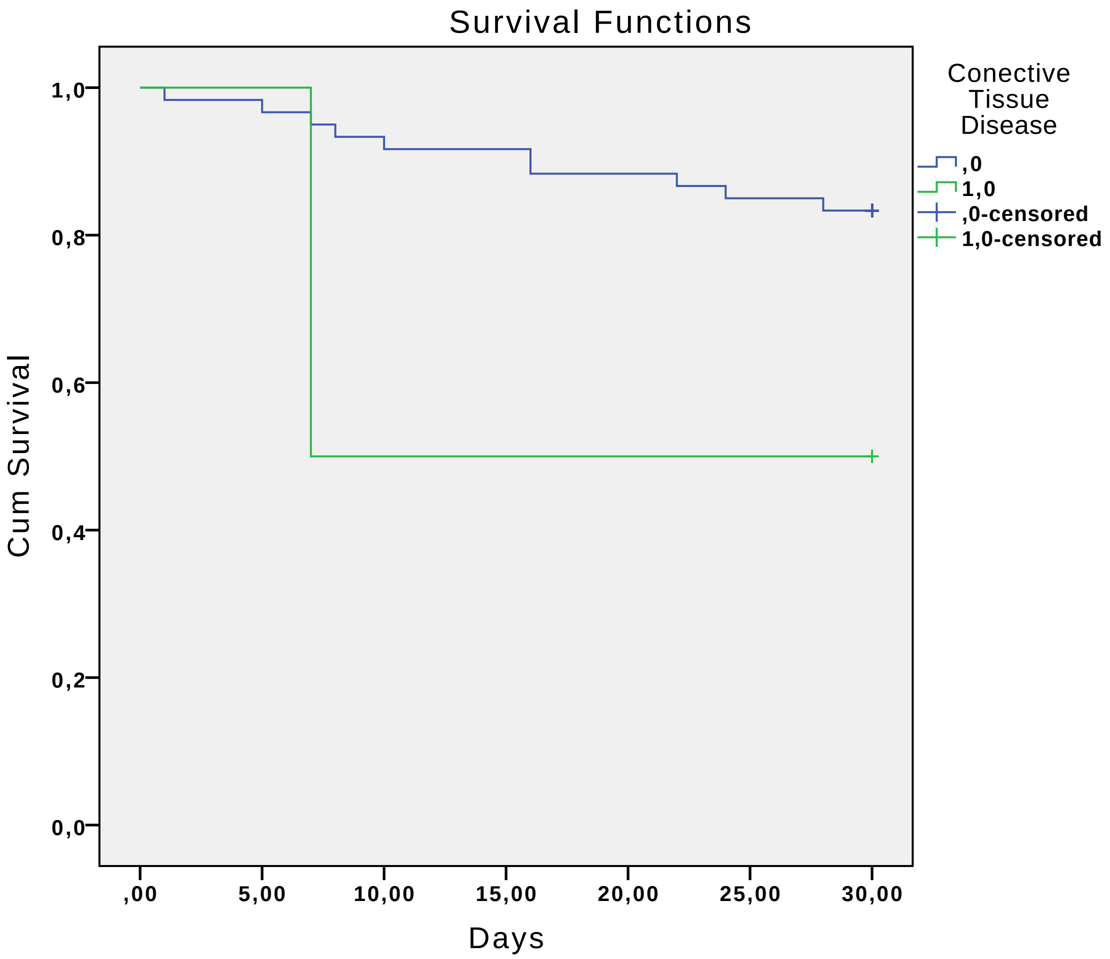

ADVANCED CHRONIC LIVER DISEASE (0=NO, 1=YES)

Case Processing Summary

Advanced Chronic Liver Disease	Total N	N of Events	Censored	
			N	Percent
,0	59	9	50	84,7%
1,0	2	2	0	0,0%
Overall	61	11	50	82,0%

Survival Table

Advanced Chronic Liver Disease		Time	Status	Cumulative Proportion Surviving at the Time		N of Cumulative Events	N of Remaining Cases
				Estimate	Std. Error		
,0	1	1,000	1,00	,983	,017	1	58
	2	5,000	1,00	,966	,024	2	57
	3	7,000	1,00	,949	,029	3	56
	4	8,000	1,00	,932	,033	4	55
	5	16,000	1,00	.	.	5	54
	6	16,000	1,00	,898	,039	6	53
	7	22,000	1,00	,881	,042	7	52
	8	24,000	1,00	,864	,045	8	51
	9	28,000	1,00	,847	,047	9	50
	10	30,000	,00	.	.	9	49
	11	30,000	,00	.	.	9	48
	12	30,000	,00	.	.	9	47
	13	30,000	,00	.	.	9	46
	14	30,000	,00	.	.	9	45
	15	30,000	,00	.	.	9	44
	16	30,000	,00	.	.	9	43
	17	30,000	,00	.	.	9	42
	18	30,000	,00	.	.	9	41
	19	30,000	,00	.	.	9	40
	20	30,000	,00	.	.	9	39
	21	30,000	,00	.	.	9	38
	22	30,000	,00	.	.	9	37
	23	30,000	,00	.	.	9	36
	24	30,000	,00	.	.	9	35
	25	30,000	,00	.	.	9	34
	26	30,000	,00	.	.	9	33
	27	30,000	,00	.	.	9	32
	28	30,000	,00	.	.	9	31
	29	30,000	,00	.	.	9	30
	30	30,000	,00	.	.	9	29
	31	30,000	,00	.	.	9	28
	32	30,000	,00	.	.	9	27
	33	30,000	,00	.	.	9	26
	34	30,000	,00	.	.	9	25
	35	30,000	,00	.	.	9	24
	36	30,000	,00	.	.	9	23
	37	30,000	,00	.	.	9	22
	38	30,000	,00	.	.	9	21
	39	30,000	,00	.	.	9	20
	40	30,000	,00	.	.	9	19
	41	30,000	,00	.	.	9	18
	42	30,000	,00	.	.	9	17
	43	30,000	,00	.	.	9	16
	44	30,000	,00	.	.	9	15
	45	30,000	,00	.	.	9	14
	46	30,000	,00	.	.	9	13
	47	30,000	,00	.	.	9	12
	48	30,000	,00	.	.	9	11
	49	30,000	,00	.	.	9	10

Survival Table

Advanced Chronic Liver Disease		Time	Status	Cumulative Proportion Surviving at the Time		N of Cumulative Events	N of Remaining Cases
				Estimate	Std. Error		
	50	30,000	,00	.	.	9	9
	51	30,000	,00	.	.	9	8
	52	30,000	,00	.	.	9	7
	53	30,000	,00	.	.	9	6
	54	30,000	,00	.	.	9	5
	55	30,000	,00	.	.	9	4
	56	30,000	,00	.	.	9	3
	57	30,000	,00	.	.	9	2
	58	30,000	,00	.	.	9	1
	59	30,000	,00	.	.	9	0
1,0	1	7,000	1,00	,500	,354	1	1
	2	10,000	1,00	,000	,000	2	0

Means and Medians for Survival Time

Advanced Chronic Liver Disease	Mean[a]				Median		
			95% Confidence Interval				95% …
	Estimate	Std. Error	Lower Bound	Upper Bound	Estimate	Std. Error	Lower Bound
,0	27,576	,869	25,873	29,279	.	.	.
1,0	8,500	1,500	5,560	11,440	7,000	.	.
Overall	26,951	,947	25,095	28,807	.	.	.

Means and Medians for Survival Time

Advanced Chronic Liver Disease	Median
	95% …
	Upper Bound
,0	.
1,0	.
Overall	.

a. Estimation is limited to the largest survival time if it is censored.

Overall Comparisons

	Chi-Square	df	Sig.
Log Rank (Mantel-Cox)	20,551	1	,000

Test of equality of survival distributions for the different levels of Advanced Chronic Liver Disease

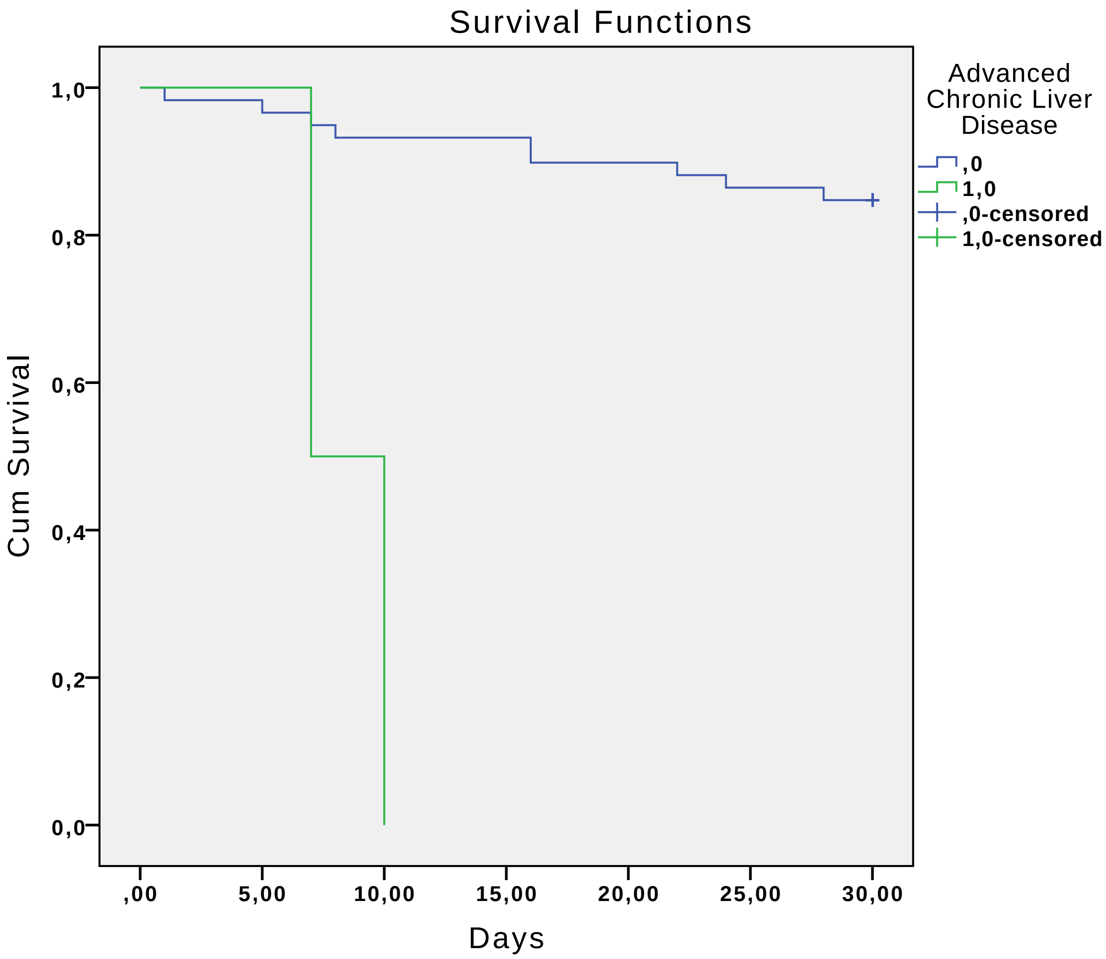

DIABETES MELLITUS (0=NO, 1=YES)

Case Processing Summary

Diabetes Mellitus	Total N	N of Events	Censored	
			N	Percent
,0	43	5	38	88,4%
1,0	19	6	13	68,4%
Overall	62	11	51	82,3%

Survival Table

Diabetes Mellitus		Time	Status	Cumulative Proportion Surviving at the Time		N of Cumulative Events	N of Remaining Cases
				Estimate	Std. Error		
,0	1	5,000	1,00	,977	,023	1	42
	2	10,000	1,00	,953	,032	2	41
	3	16,000	1,00	.	.	3	40
	4	16,000	1,00	,907	,044	4	39
	5	28,000	1,00	,884	,049	5	38
	6	30,000	,00	.	.	5	37
	7	30,000	,00	.	.	5	36
	8	30,000	,00	.	.	5	35
	9	30,000	,00	.	.	5	34
	10	30,000	,00	.	.	5	33
	11	30,000	,00	.	.	5	32
	12	30,000	,00	.	.	5	31
	13	30,000	,00	.	.	5	30
	14	30,000	,00	.	.	5	29
	15	30,000	,00	.	.	5	28
	16	30,000	,00	.	.	5	27
	17	30,000	,00	.	.	5	26
	18	30,000	,00	.	.	5	25
	19	30,000	,00	.	.	5	24
	20	30,000	,00	.	.	5	23
	21	30,000	,00	.	.	5	22
	22	30,000	,00	.	.	5	21
	23	30,000	,00	.	.	5	20
	24	30,000	,00	.	.	5	19
	25	30,000	,00	.	.	5	18
	26	30,000	,00	.	.	5	17
	27	30,000	,00	.	.	5	16
	28	30,000	,00	.	.	5	15
	29	30,000	,00	.	.	5	14
	30	30,000	,00	.	.	5	13
	31	30,000	,00	.	.	5	12
	32	30,000	,00	.	.	5	11
	33	30,000	,00	.	.	5	10
	34	30,000	,00	.	.	5	9
	35	30,000	,00	.	.	5	8
	36	30,000	,00	.	.	5	7
	37	30,000	,00	.	.	5	6
	38	30,000	,00	.	.	5	5
	39	30,000	,00	.	.	5	4
	40	30,000	,00	.	.	5	3
	41	30,000	,00	.	.	5	2
	42	30,000	,00	.	.	5	1
	43	30,000	,00	.	.	5	0
1,0	1	1,000	1,00	,947	,051	1	18
	2	7,000	1,00	.	.	2	17
	3	7,000	1,00	,842	,084	3	16
	4	8,000	1,00	,789	,094	4	15
	5	22,000	1,00	,737	,101	5	14
	6	24,000	1,00	,684	,107	6	13

Survival Table

Diabetes Mellitus	Time	Status	Cumulative Proportion Surviving at the Time		N of Cumulative Events	N of Remaining Cases
			Estimate	Std. Error		
7	30,000	,00	.	.	6	12
8	30,000	,00	.	.	6	11
9	30,000	,00	.	.	6	10
10	30,000	,00	.	.	6	9
11	30,000	,00	.	.	6	8
12	30,000	,00	.	.	6	7
13	30,000	,00	.	.	6	6
14	30,000	,00	.	.	6	5
15	30,000	,00	.	.	6	4
16	30,000	,00	.	.	6	3
17	30,000	,00	.	.	6	2
18	30,000	,00	.	.	6	1
19	30,000	,00	.	.	6	0

Means and Medians for Survival Time

Diabetes Mellitus	Mean[a]				Median		
			95% Confidence Interval				95% ...
	Estimate	Std. Error	Lower Bound	Upper Bound	Estimate	Std. Error	Lower Bound
,0	28,256	,835	26,619	29,893	.	.	.
1,0	24,158	2,254	19,740	28,576	.	.	.
Overall	27,000	,933	25,171	28,829	.	.	.

Means and Medians for Survival Time

Diabetes Mellitus	Median
	95% ...
	Upper Bound
,0	.
1,0	.
Overall	.

a. Estimation is limited to the largest survival time if it is censored.

Overall Comparisons

	Chi-Square	df	Sig.
Log Rank (Mantel-Cox)	3,917	1	,048

Test of equality of survival distributions for the different levels of Diabetes Mellitus

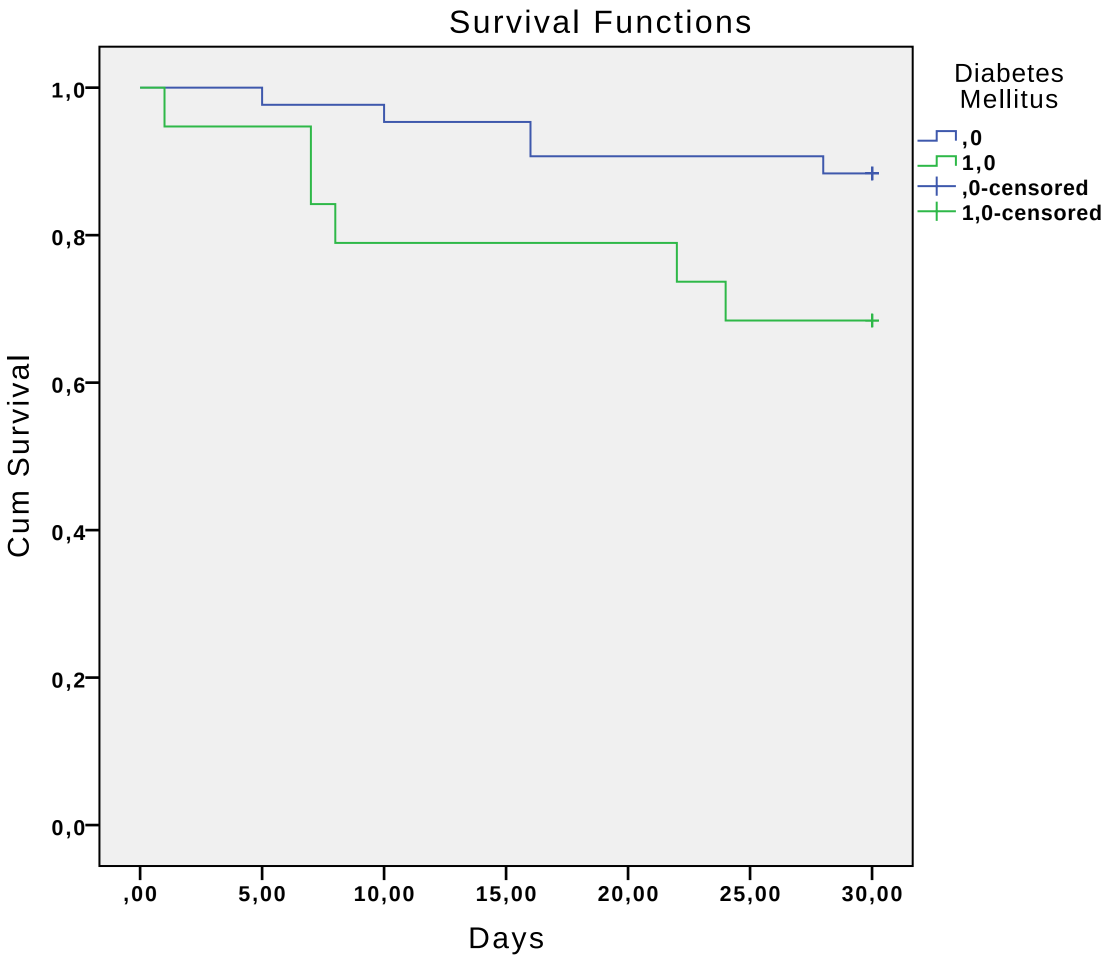

DIABETES MELLITUS WITH TARGET ORGAN DAMAGE (0=NO, 1=YES)

Case Processing Summary

Diabetes Mellitus with Target Organ Damage	Total N	N of Events	Censored	
			N	Percent
,0	55	9	46	83,6%
1,0	7	2	5	71,4%
Overall	62	11	51	82,3%

Survival Table

Diabetes Mellitus with Target Organ Damage		Time	Status	Cumulative Proportion Surviving at the Time		N of Cumulative Events
				Estimate	Std. Error	
,0	1	1,000	1,00	,982	,018	1
	2	5,000	1,00	,964	,025	2
	3	7,000	1,00	,945	,031	3
	4	8,000	1,00	,927	,035	4
	5	10,000	1,00	,909	,039	5
	6	16,000	1,00	.	.	6
	7	16,000	1,00	,873	,045	7
	8	22,000	1,00	,855	,048	8
	9	28,000	1,00	,836	,050	9
	10	30,000	,00	.	.	9
	11	30,000	,00	.	.	9
	12	30,000	,00	.	.	9
	13	30,000	,00	.	.	9
	14	30,000	,00	.	.	9
	15	30,000	,00	.	.	9
	16	30,000	,00	.	.	9
	17	30,000	,00	.	.	9
	18	30,000	,00	.	.	9
	19	30,000	,00	.	.	9
	20	30,000	,00	.	.	9
	21	30,000	,00	.	.	9
	22	30,000	,00	.	.	9
	23	30,000	,00	.	.	9
	24	30,000	,00	.	.	9
	25	30,000	,00	.	.	9
	26	30,000	,00	.	.	9
	27	30,000	,00	.	.	9
	28	30,000	,00	.	.	9
	29	30,000	,00	.	.	9
	30	30,000	,00	.	.	9
	31	30,000	,00	.	.	9
	32	30,000	,00	.	.	9
	33	30,000	,00	.	.	9
	34	30,000	,00	.	.	9
	35	30,000	,00	.	.	9
	36	30,000	,00	.	.	9
	37	30,000	,00	.	.	9
	38	30,000	,00	.	.	9
	39	30,000	,00	.	.	9
	40	30,000	,00	.	.	9
	41	30,000	,00	.	.	9
	42	30,000	,00	.	.	9
	43	30,000	,00	.	.	9
	44	30,000	,00	.	.	9
	45	30,000	,00	.	.	9
	46	30,000	,00	.	.	9
	47	30,000	,00	.	.	9
	48	30,000	,00	.	.	9
	49	30,000	,00	.	.	9

Survival Table

Diabetes Mellitus with Target Organ Damage		N of Remaining Cases
,0	1	54
	2	53
	3	52
	4	51
	5	50
	6	49
	7	48
	8	47
	9	46
	10	45
	11	44
	12	43
	13	42
	14	41
	15	40
	16	39
	17	38
	18	37
	19	36
	20	35
	21	34
	22	33
	23	32
	24	31
	25	30
	26	29
	27	28
	28	27
	29	26
	30	25
	31	24
	32	23
	33	22
	34	21
	35	20
	36	19
	37	18
	38	17
	39	16
	40	15
	41	14
	42	13
	43	12
	44	11
	45	10
	46	9
	47	8
	48	7
	49	6

Survival Table

Diabetes Mellitus with Target Organ Damage		Time	Status	Cumulative Proportion Surviving at the Time: Estimate	Cumulative Proportion Surviving at the Time: Std. Error	N of Cumulative Events
	50	30,000	,00	.	.	9
	51	30,000	,00	.	.	9
	52	30,000	,00	.	.	9
	53	30,000	,00	.	.	9
	54	30,000	,00	.	.	9
	55	30,000	,00	.	.	9
1,0	1	7,000	1,00	,857	,132	1
	2	24,000	1,00	,714	,171	2
	3	30,000	,00	.	.	2
	4	30,000	,00	.	.	2
	5	30,000	,00	.	.	2
	6	30,000	,00	.	.	2
	7	30,000	,00	.	.	2

Survival Table

Diabetes Mellitus with Target Organ Damage		N of Remaining Cases
	50	5
	51	4
	52	3
	53	2
	54	1
	55	0
1,0	1	6
	2	5
	3	4
	4	3
	5	2
	6	1
	7	0

Means and Medians for Survival Time

Diabetes Mellitus with Target Organ Damage	Mean[a]				Median		
			95% Confidence Interval				95% …
	Estimate	Std. Error	Lower Bound	Upper Bound	Estimate	Std. Error	Lower Bound
,0	27,145	,978	25,229	29,061	.	.	.
1,0	25,857	3,013	19,951	31,763	.	.	.
Overall	27,000	,933	25,171	28,829	.	.	.

Means and Medians for Survival Time

Diabetes Mellitus with Target Organ Damage	Median
	95% …
	Upper Bound
,0	.
1,0	.
Overall	.

a. Estimation is limited to the largest survival time if it is censored.

Overall Comparisons

	Chi-Square	df	Sig.
Log Rank (Mantel-Cox)	,594	1	,441

Test of equality of survival distributions for the different levels of Diabetes Mellitus with Target Organ Damage

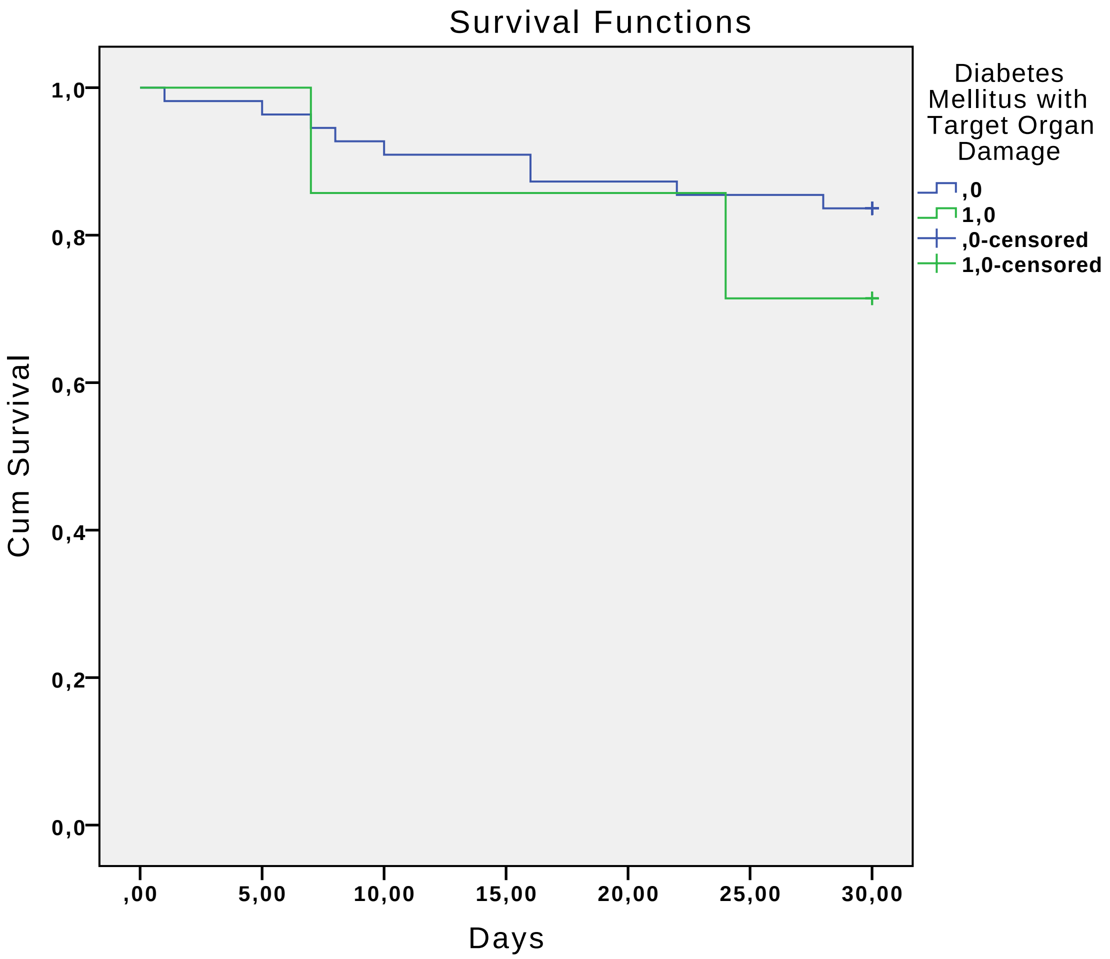

HYPERTENSION (0=NO, 1=YES)

Case Processing Summary

Hypertension	Total N	N of Events	Censored	
			N	Percent
,0	28	4	24	85,7%
1,0	34	7	27	79,4%
Overall	62	11	51	82,3%

Survival Table

Hypertension		Time	Status	Cumulative Proportion Surviving at the Time		N of Cumulative Events	N of Remaining Cases
				Estimate	Std. Error		
,0	1	5,000	1,00	,964	,035	1	27
	2	7,000	1,00	,929	,049	2	26
	3	10,000	1,00	,893	,058	3	25
	4	16,000	1,00	,857	,066	4	24
	5	30,000	,00	.	.	4	23
	6	30,000	,00	.	.	4	22
	7	30,000	,00	.	.	4	21
	8	30,000	,00	.	.	4	20
	9	30,000	,00	.	.	4	19
	10	30,000	,00	.	.	4	18
	11	30,000	,00	.	.	4	17
	12	30,000	,00	.	.	4	16
	13	30,000	,00	.	.	4	15
	14	30,000	,00	.	.	4	14
	15	30,000	,00	.	.	4	13
	16	30,000	,00	.	.	4	12
	17	30,000	,00	.	.	4	11
	18	30,000	,00	.	.	4	10
	19	30,000	,00	.	.	4	9
	20	30,000	,00	.	.	4	8
	21	30,000	,00	.	.	4	7
	22	30,000	,00	.	.	4	6
	23	30,000	,00	.	.	4	5
	24	30,000	,00	.	.	4	4
	25	30,000	,00	.	.	4	3
	26	30,000	,00	.	.	4	2
	27	30,000	,00	.	.	4	1
	28	30,000	,00	.	.	4	0
1,0	1	1,000	1,00	,971	,029	1	33
	2	7,000	1,00	,941	,040	2	32
	3	8,000	1,00	,912	,049	3	31
	4	16,000	1,00	,882	,055	4	30
	5	22,000	1,00	,853	,061	5	29
	6	24,000	1,00	,824	,065	6	28
	7	28,000	1,00	,794	,069	7	27
	8	30,000	,00	.	.	7	26
	9	30,000	,00	.	.	7	25
	10	30,000	,00	.	.	7	24
	11	30,000	,00	.	.	7	23
	12	30,000	,00	.	.	7	22
	13	30,000	,00	.	.	7	21
	14	30,000	,00	.	.	7	20
	15	30,000	,00	.	.	7	19
	16	30,000	,00	.	.	7	18
	17	30,000	,00	.	.	7	17
	18	30,000	,00	.	.	7	16
	19	30,000	,00	.	.	7	15
	20	30,000	,00	.	.	7	14
	21	30,000	,00	.	.	7	13

Survival Table

Hypertension	Time	Status	Cumulative Proportion Surviving at the Time		N of Cumulative Events	N of Remaining Cases
			Estimate	Std. Error		
22	30,000	,00	.	.	7	12
23	30,000	,00	.	.	7	11
24	30,000	,00	.	.	7	10
25	30,000	,00	.	.	7	9
26	30,000	,00	.	.	7	8
27	30,000	,00	.	.	7	7
28	30,000	,00	.	.	7	6
29	30,000	,00	.	.	7	5
30	30,000	,00	.	.	7	4
31	30,000	,00	.	.	7	3
32	30,000	,00	.	.	7	2
33	30,000	,00	.	.	7	1
34	30,000	,00	.	.	7	0

Means and Medians for Survival Time

Hypertension	Mean[a]				Median		
	Estimate	Std. Error	95% Confidence Interval		Estimate	Std. Error	95% …
			Lower Bound	Upper Bound			Lower Bound
,0	27,071	1,388	24,351	29,791	.	.	.
1,0	26,941	1,260	24,471	29,411	.	.	.
Overall	27,000	,933	25,171	28,829	.	.	.

Means and Medians for Survival Time

Hypertension	Median
	95% …
	Upper Bound
,0	.
1,0	.
Overall	.

a. Estimation is limited to the largest survival time if it is censored.

Overall Comparisons

	Chi-Square	df	Sig.
Log Rank (Mantel-Cox)	,345	1	,557

Test of equality of survival distributions for the different levels of Hypertension

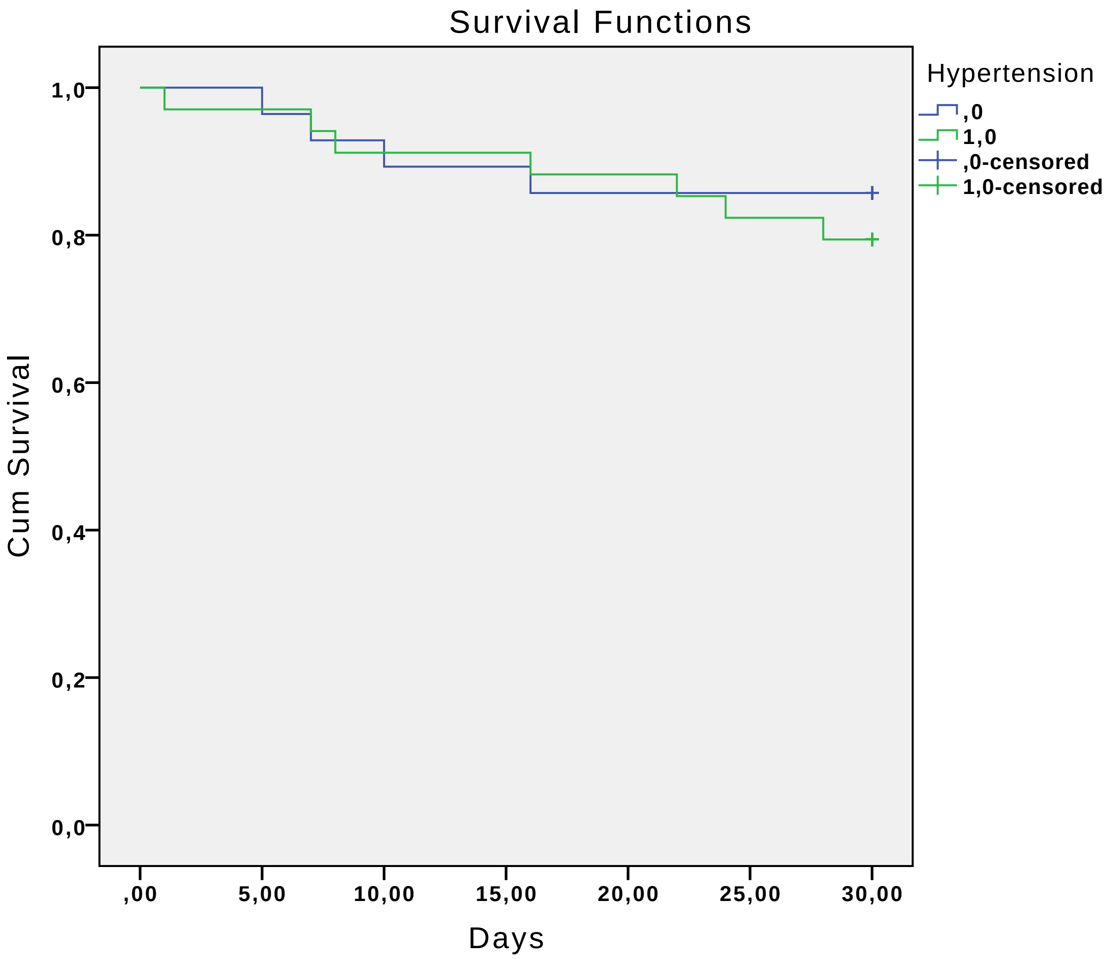

DYSLIPIDEMIA (0=NO, 1=YES)

Case Processing Summary

Dyslipidemia	Total N	N of Events	Censored	
			N	Percent
,0	39	5	34	87,2%
1,0	23	6	17	73,9%
Overall	62	11	51	82,3%

Survival Table

Dyslipidemia		Time	Status	Cumulative Proportion Surviving at the Time		N of Cumulative Events	N of Remaining Cases
				Estimate	Std. Error		
,0	1	5,000	1,00	,974	,025	1	38
	2	7,000	1,00	,949	,035	2	37
	3	10,000	1,00	,923	,043	3	36
	4	22,000	1,00	,897	,049	4	35
	5	28,000	1,00	,872	,054	5	34
	6	30,000	,00	.	.	5	33
	7	30,000	,00	.	.	5	32
	8	30,000	,00	.	.	5	31
	9	30,000	,00	.	.	5	30
	10	30,000	,00	.	.	5	29
	11	30,000	,00	.	.	5	28
	12	30,000	,00	.	.	5	27
	13	30,000	,00	.	.	5	26
	14	30,000	,00	.	.	5	25
	15	30,000	,00	.	.	5	24
	16	30,000	,00	.	.	5	23
	17	30,000	,00	.	.	5	22
	18	30,000	,00	.	.	5	21
	19	30,000	,00	.	.	5	20
	20	30,000	,00	.	.	5	19
	21	30,000	,00	.	.	5	18
	22	30,000	,00	.	.	5	17
	23	30,000	,00	.	.	5	16
	24	30,000	,00	.	.	5	15
	25	30,000	,00	.	.	5	14
	26	30,000	,00	.	.	5	13
	27	30,000	,00	.	.	5	12
	28	30,000	,00	.	.	5	11
	29	30,000	,00	.	.	5	10
	30	30,000	,00	.	.	5	9
	31	30,000	,00	.	.	5	8
	32	30,000	,00	.	.	5	7
	33	30,000	,00	.	.	5	6
	34	30,000	,00	.	.	5	5
	35	30,000	,00	.	.	5	4
	36	30,000	,00	.	.	5	3
	37	30,000	,00	.	.	5	2
	38	30,000	,00	.	.	5	1
	39	30,000	,00	.	.	5	0
1,0	1	1,000	1,00	,957	,043	1	22
	2	7,000	1,00	,913	,059	2	21
	3	8,000	1,00	,870	,070	3	20
	4	16,000	1,00	.	.	4	19
	5	16,000	1,00	,783	,086	5	18
	6	24,000	1,00	,739	,092	6	17
	7	30,000	,00	.	.	6	16
	8	30,000	,00	.	.	6	15
	9	30,000	,00	.	.	6	14
	10	30,000	,00	.	.	6	13

Survival Table

Dyslipidemia	Time	Status	Cumulative Proportion Surviving at the Time		N of Cumulative Events	N of Remaining Cases
			Estimate	Std. Error		
11	30,000	,00	.	.	6	12
12	30,000	,00	.	.	6	11
13	30,000	,00	.	.	6	10
14	30,000	,00	.	.	6	9
15	30,000	,00	.	.	6	8
16	30,000	,00	.	.	6	7
17	30,000	,00	.	.	6	6
18	30,000	,00	.	.	6	5
19	30,000	,00	.	.	6	4
20	30,000	,00	.	.	6	3
21	30,000	,00	.	.	6	2
22	30,000	,00	.	.	6	1
23	30,000	,00	.	.	6	0

Means and Medians for Survival Time

Dyslipidemia	Mean[a]				Median		
			95% Confidence Interval				95% …
	Estimate	Std. Error	Lower Bound	Upper Bound	Estimate	Std. Error	Lower Bound
,0	28,000	,982	26,076	29,924	.	.	.
1,0	25,304	1,832	21,714	28,894	.	.	.
Overall	27,000	,933	25,171	28,829	.	.	.

Means and Medians for Survival Time

Dyslipidemia	Median
	95% …
	Upper Bound
,0	.
1,0	.
Overall	.

a. Estimation is limited to the largest survival time if it is censored.

Overall Comparisons

	Chi-Square	df	Sig.
Log Rank (Mantel-Cox)	1,780	1	,182

Test of equality of survival distributions for the different levels of Dyslipidemia

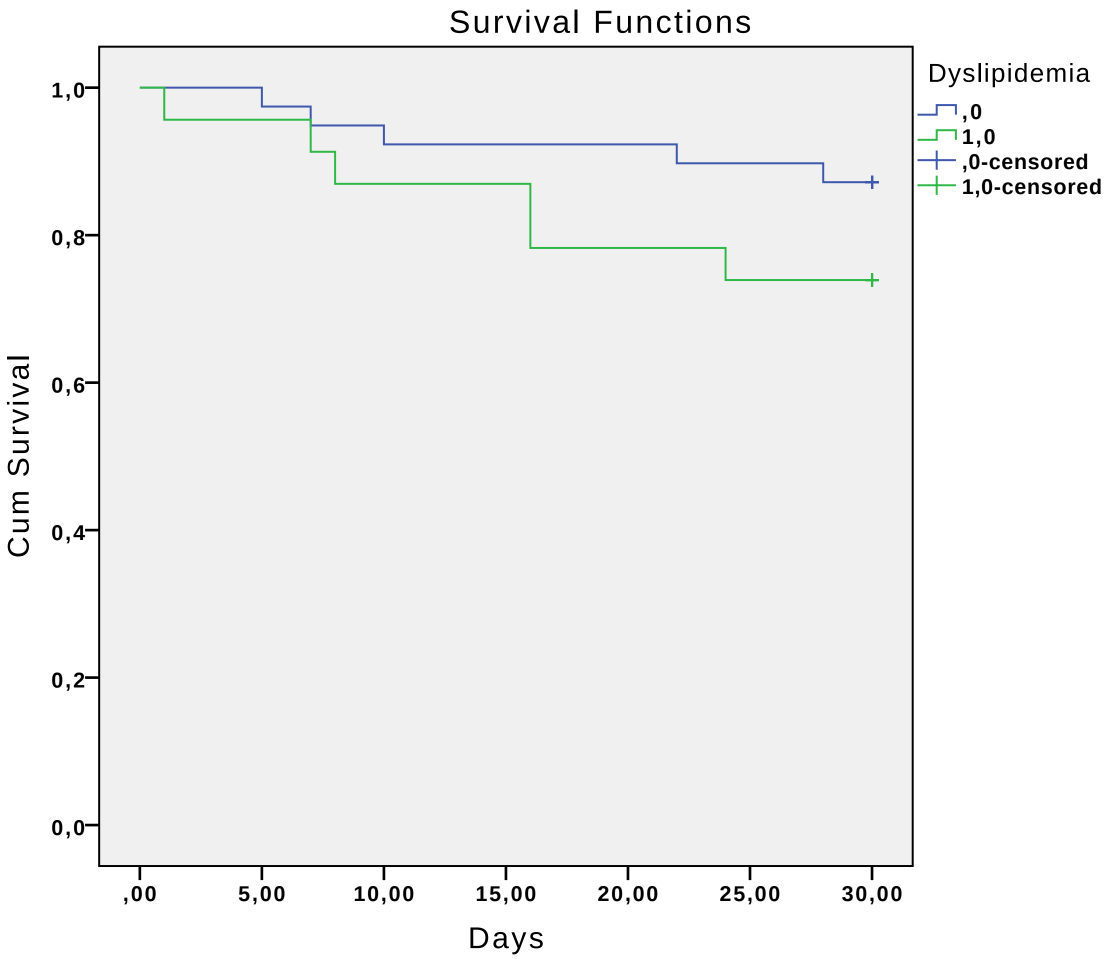

RESPIRATORY DISEASE (0=NO, 1=YES)

Case Processing Summary

Respiratory Disease	Total N	N of Events	Censored	
			N	Percent
,0	48	7	41	85,4%
1,0	14	4	10	71,4%
Overall	62	11	51	82,3%

Survival Table

Respiratory Disease		Time	Status	Cumulative Proportion Surviving at the Time		N of Cumulative Events	N of Remaining Cases
				Estimate	Std. Error		
,0	1	5,000	1,00	,979	,021	1	47
	2	7,000	1,00	,958	,029	2	46
	3	8,000	1,00	,938	,035	3	45
	4	16,000	1,00	.	.	4	44
	5	16,000	1,00	,896	,044	5	43
	6	22,000	1,00	,875	,048	6	42
	7	24,000	1,00	,854	,051	7	41
	8	30,000	,00	.	.	7	40
	9	30,000	,00	.	.	7	39
	10	30,000	,00	.	.	7	38
	11	30,000	,00	.	.	7	37
	12	30,000	,00	.	.	7	36
	13	30,000	,00	.	.	7	35
	14	30,000	,00	.	.	7	34
	15	30,000	,00	.	.	7	33
	16	30,000	,00	.	.	7	32
	17	30,000	,00	.	.	7	31
	18	30,000	,00	.	.	7	30
	19	30,000	,00	.	.	7	29
	20	30,000	,00	.	.	7	28
	21	30,000	,00	.	.	7	27
	22	30,000	,00	.	.	7	26
	23	30,000	,00	.	.	7	25
	24	30,000	,00	.	.	7	24
	25	30,000	,00	.	.	7	23
	26	30,000	,00	.	.	7	22
	27	30,000	,00	.	.	7	21
	28	30,000	,00	.	.	7	20
	29	30,000	,00	.	.	7	19
	30	30,000	,00	.	.	7	18
	31	30,000	,00	.	.	7	17
	32	30,000	,00	.	.	7	16
	33	30,000	,00	.	.	7	15
	34	30,000	,00	.	.	7	14
	35	30,000	,00	.	.	7	13
	36	30,000	,00	.	.	7	12
	37	30,000	,00	.	.	7	11
	38	30,000	,00	.	.	7	10
	39	30,000	,00	.	.	7	9
	40	30,000	,00	.	.	7	8
	41	30,000	,00	.	.	7	7
	42	30,000	,00	.	.	7	6
	43	30,000	,00	.	.	7	5
	44	30,000	,00	.	.	7	4
	45	30,000	,00	.	.	7	3
	46	30,000	,00	.	.	7	2
	47	30,000	,00	.	.	7	1
	48	30,000	,00	.	.	7	0

Survival Table

Respiratory Disease		Time	Status	Cumulative Proportion Surviving at the Time		N of Cumulative Events	N of Remaining Cases
				Estimate	Std. Error		
1,0	1	1,000	1,00	,929	,069	1	13
	2	7,000	1,00	,857	,094	2	12
	3	10,000	1,00	,786	,110	3	11
	4	28,000	1,00	,714	,121	4	10
	5	30,000	,00	.	.	4	9
	6	30,000	,00	.	.	4	8
	7	30,000	,00	.	.	4	7
	8	30,000	,00	.	.	4	6
	9	30,000	,00	.	.	4	5
	10	30,000	,00	.	.	4	4
	11	30,000	,00	.	.	4	3
	12	30,000	,00	.	.	4	2
	13	30,000	,00	.	.	4	1
	14	30,000	,00	.	.	4	0

Means and Medians for Survival Time

Respiratory Disease	Mean[a]				Median		
			95% Confidence Interval				95% …
	Estimate	Std. Error	Lower Bound	Upper Bound	Estimate	Std. Error	Lower Bound
,0	27,667	,901	25,902	29,432	.	.	.
1,0	24,714	2,656	19,508	29,920	.	.	.
Overall	27,000	,933	25,171	28,829	.	.	.

Means and Medians for Survival Time

Respiratory Disease	Median
	95% …
	Upper Bound
,0	.
1,0	.
Overall	.

a. Estimation is limited to the largest survival time if it is censored.

Overall Comparisons

	Chi-Square	df	Sig.
Log Rank (Mantel-Cox)	1,587	1	,208

Test of equality of survival distributions for the different levels of Respiratory Disease.

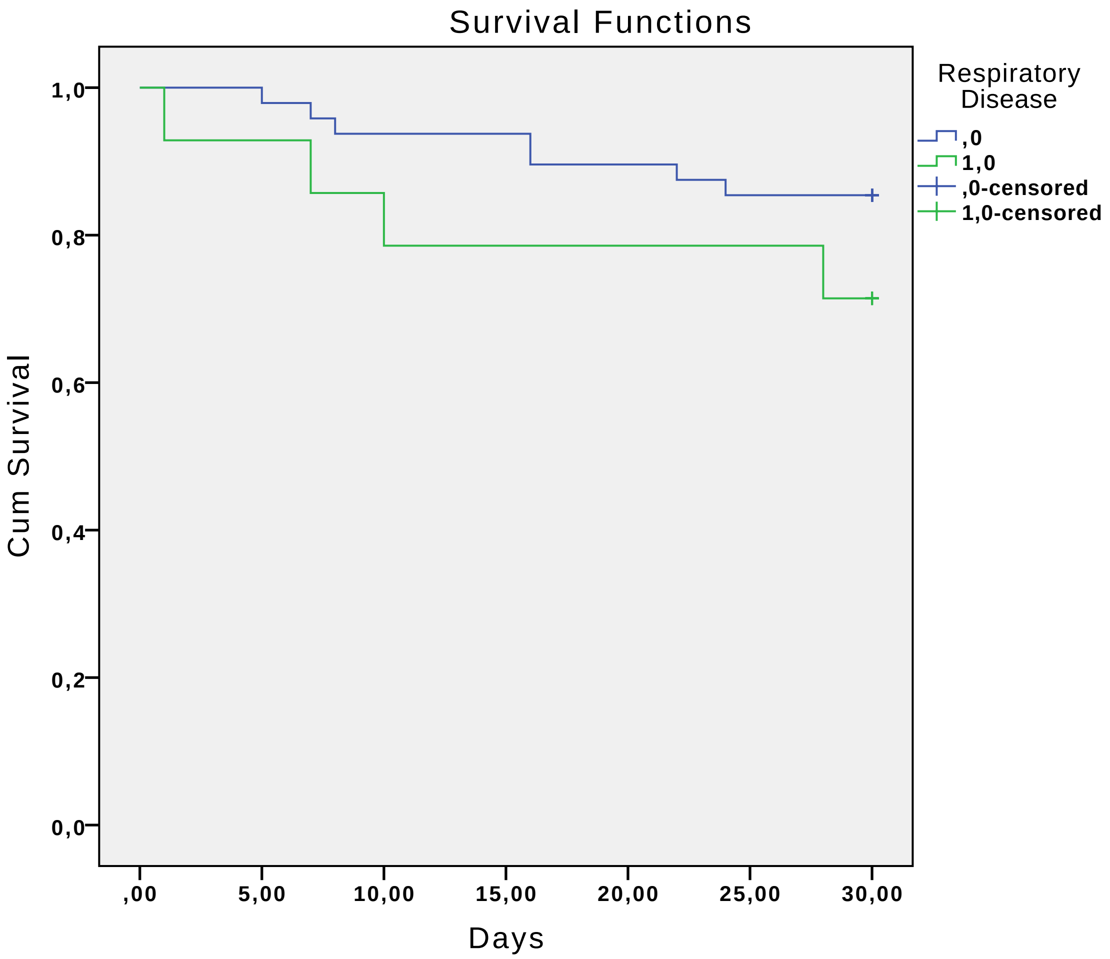

COPD (0=NO, 1=YES)

Case Processing Summary

COPD	Total N	N of Events	Censored	
			N	Percent
,0	54	9	45	83,3%
1,0	8	2	6	75,0%
Overall	62	11	51	82,3%

Survival Table

COPD		Time	Status	Cumulative Proportion Surviving at the Time		N of Cumulative Events	N of Remaining Cases
				Estimate	Std. Error		
,0	1	5,000	1,00	,981	,018	1	53
	2	7,000	1,00	.	.	2	52
	3	7,000	1,00	,944	,031	3	51
	4	8,000	1,00	,926	,036	4	50
	5	16,000	1,00	.	.	5	49
	6	16,000	1,00	,889	,043	6	48
	7	22,000	1,00	,870	,046	7	47
	8	24,000	1,00	,852	,048	8	46
	9	28,000	1,00	,833	,051	9	45
	10	30,000	,00	.	.	9	44
	11	30,000	,00	.	.	9	43
	12	30,000	,00	.	.	9	42
	13	30,000	,00	.	.	9	41
	14	30,000	,00	.	.	9	40
	15	30,000	,00	.	.	9	39
	16	30,000	,00	.	.	9	38
	17	30,000	,00	.	.	9	37
	18	30,000	,00	.	.	9	36
	19	30,000	,00	.	.	9	35
	20	30,000	,00	.	.	9	34
	21	30,000	,00	.	.	9	33
	22	30,000	,00	.	.	9	32
	23	30,000	,00	.	.	9	31
	24	30,000	,00	.	.	9	30
	25	30,000	,00	.	.	9	29
	26	30,000	,00	.	.	9	28
	27	30,000	,00	.	.	9	27
	28	30,000	,00	.	.	9	26
	29	30,000	,00	.	.	9	25
	30	30,000	,00	.	.	9	24
	31	30,000	,00	.	.	9	23
	32	30,000	,00	.	.	9	22
	33	30,000	,00	.	.	9	21
	34	30,000	,00	.	.	9	20
	35	30,000	,00	.	.	9	19
	36	30,000	,00	.	.	9	18
	37	30,000	,00	.	.	9	17
	38	30,000	,00	.	.	9	16
	39	30,000	,00	.	.	9	15
	40	30,000	,00	.	.	9	14
	41	30,000	,00	.	.	9	13
	42	30,000	,00	.	.	9	12
	43	30,000	,00	.	.	9	11
	44	30,000	,00	.	.	9	10
	45	30,000	,00	.	.	9	9
	46	30,000	,00	.	.	9	8
	47	30,000	,00	.	.	9	7
	48	30,000	,00	.	.	9	6
	49	30,000	,00	.	.	9	5

Survival Table

COPD		Time	Status	Cumulative Proportion Surviving at the Time: Estimate	Cumulative Proportion Surviving at the Time: Std. Error	N of Cumulative Events	N of Remaining Cases
	50	30,000	,00	.	.	9	4
	51	30,000	,00	.	.	9	3
	52	30,000	,00	.	.	9	2
	53	30,000	,00	.	.	9	1
	54	30,000	,00	.	.	9	0
1,0	1	1,000	1,00	,875	,117	1	7
	2	10,000	1,00	,750	,153	2	6
	3	30,000	,00	.	.	2	5
	4	30,000	,00	.	.	2	4
	5	30,000	,00	.	.	2	3
	6	30,000	,00	.	.	2	2
	7	30,000	,00	.	.	2	1
	8	30,000	,00	.	.	2	0

Means and Medians for Survival Time

COPD	Mean[a]				Median			
	Estimate	Std. Error	95% Confidence Interval: Lower Bound	95% Confidence Interval: Upper Bound	Estimate	Std. Error	95% Confidence Interval: Lower Bound	95% Confidence Interval: Upper Bound
,0	27,463	,891	25,716	29,209	.	.	.	.
1,0	23,875	3,834	16,360	31,390	.	.	.	.
Overall	27,000	,933	25,171	28,829	.	.	.	.

a. Estimation is limited to the largest survival time if it is censored.

Overall Comparisons

	Chi-Square	df	Sig.
Log Rank (Mantel-Cox)	,470	1	,493

Test of equality of survival distributions for the different levels of COPD

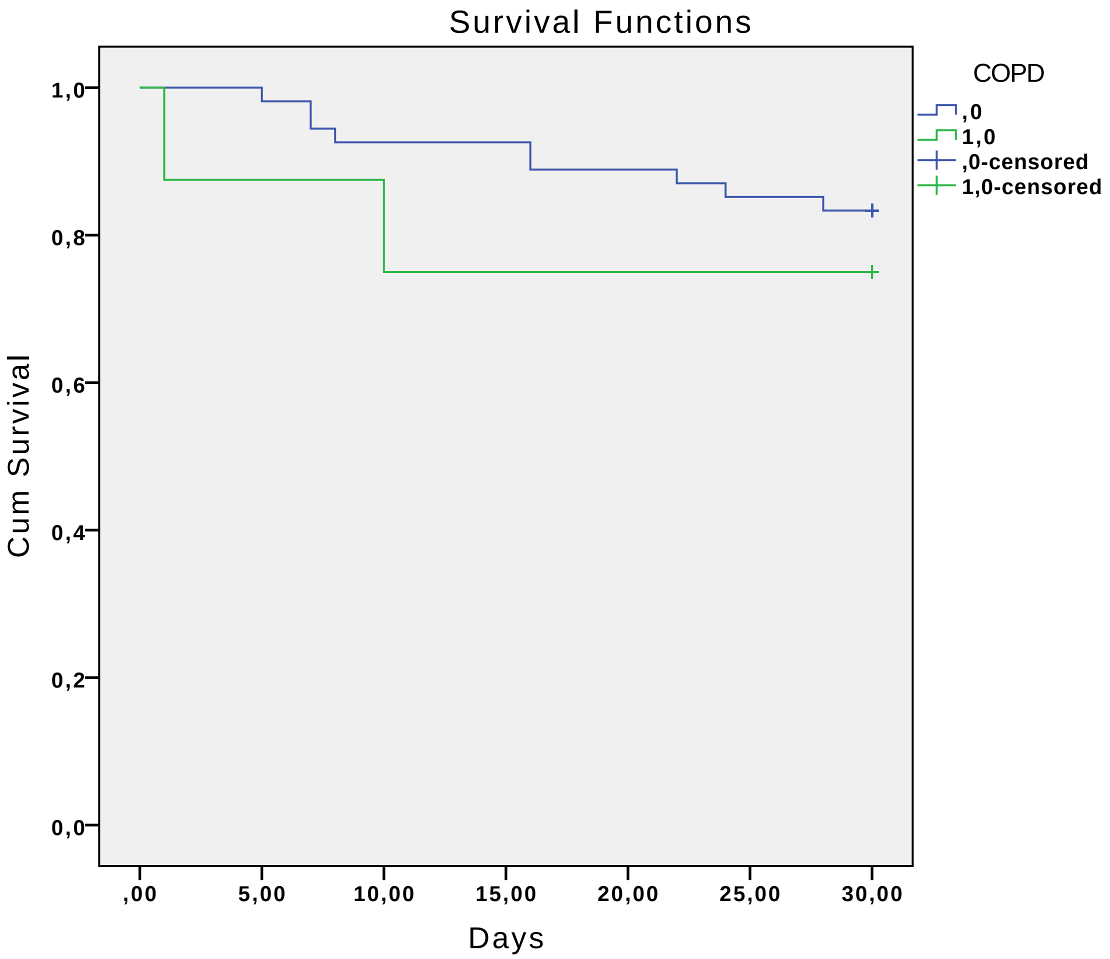

HEART DISEASE (0=NO, 1=YES)

Case Processing Summary

Heart Disease	Total N	N of Events	Censored	
			N	Percent
,0	34	4	30	88,2%
1,0	28	7	21	75,0%
Overall	62	11	51	82,3%

Survival Table

Heart Disease		Time	Status	Cumulative Proportion Surviving at the Time		N of Cumulative Events	N of Remaining Cases
				Estimate	Std. Error		
,0	1	5,000	1,00	,971	,029	1	33
	2	7,000	1,00	,941	,040	2	32
	3	8,000	1,00	,912	,049	3	31
	4	22,000	1,00	,882	,055	4	30
	5	30,000	,00	.	.	4	29
	6	30,000	,00	.	.	4	28
	7	30,000	,00	.	.	4	27
	8	30,000	,00	.	.	4	26
	9	30,000	,00	.	.	4	25
	10	30,000	,00	.	.	4	24
	11	30,000	,00	.	.	4	23
	12	30,000	,00	.	.	4	22
	13	30,000	,00	.	.	4	21
	14	30,000	,00	.	.	4	20
	15	30,000	,00	.	.	4	19
	16	30,000	,00	.	.	4	18
	17	30,000	,00	.	.	4	17
	18	30,000	,00	.	.	4	16
	19	30,000	,00	.	.	4	15
	20	30,000	,00	.	.	4	14
	21	30,000	,00	.	.	4	13
	22	30,000	,00	.	.	4	12
	23	30,000	,00	.	.	4	11
	24	30,000	,00	.	.	4	10
	25	30,000	,00	.	.	4	9
	26	30,000	,00	.	.	4	8
	27	30,000	,00	.	.	4	7
	28	30,000	,00	.	.	4	6
	29	30,000	,00	.	.	4	5
	30	30,000	,00	.	.	4	4
	31	30,000	,00	.	.	4	3
	32	30,000	,00	.	.	4	2
	33	30,000	,00	.	.	4	1
	34	30,000	,00	.	.	4	0
1,0	1	1,000	1,00	,964	,035	1	27
	2	7,000	1,00	,929	,049	2	26
	3	10,000	1,00	,893	,058	3	25
	4	16,000	1,00	.	.	4	24
	5	16,000	1,00	,821	,072	5	23
	6	24,000	1,00	,786	,078	6	22
	7	28,000	1,00	,750	,082	7	21
	8	30,000	,00	.	.	7	20
	9	30,000	,00	.	.	7	19
	10	30,000	,00	.	.	7	18
	11	30,000	,00	.	.	7	17
	12	30,000	,00	.	.	7	16
	13	30,000	,00	.	.	7	15
	14	30,000	,00	.	.	7	14
	15	30,000	,00	.	.	7	13

Survival Table

Heart Disease	Time	Status	Cumulative Proportion Surviving at the Time		N of Cumulative Events	N of Remaining Cases
			Estimate	Std. Error		
16	30,000	,00	.	.	7	12
17	30,000	,00	.	.	7	11
18	30,000	,00	.	.	7	10
19	30,000	,00	.	.	7	9
20	30,000	,00	.	.	7	8
21	30,000	,00	.	.	7	7
22	30,000	,00	.	.	7	6
23	30,000	,00	.	.	7	5
24	30,000	,00	.	.	7	4
25	30,000	,00	.	.	7	3
26	30,000	,00	.	.	7	2
27	30,000	,00	.	.	7	1
28	30,000	,00	.	.	7	0

Means and Medians for Survival Time

Heart Disease	Mean[a]				Median		
	Estimate	Std. Error	95% Confidence Interval		Estimate	Std. Error	95% …
			Lower Bound	Upper Bound			Lower Bound
,0	27,706	1,148	25,456	29,956	.	.	.
1,0	26,143	1,509	23,185	29,101	.	.	.
Overall	27,000	,933	25,171	28,829	.	.	.

Means and Medians for Survival Time

Heart Disease	Median
	95% …
	Upper Bound
,0	.
1,0	.
Overall	.

a. Estimation is limited to the largest survival time if it is censored.

Overall Comparisons

	Chi-Square	df	Sig.
Log Rank (Mantel-Cox)	1,676	1	,195

Test of equality of survival distributions for the different levels of Heart Disease

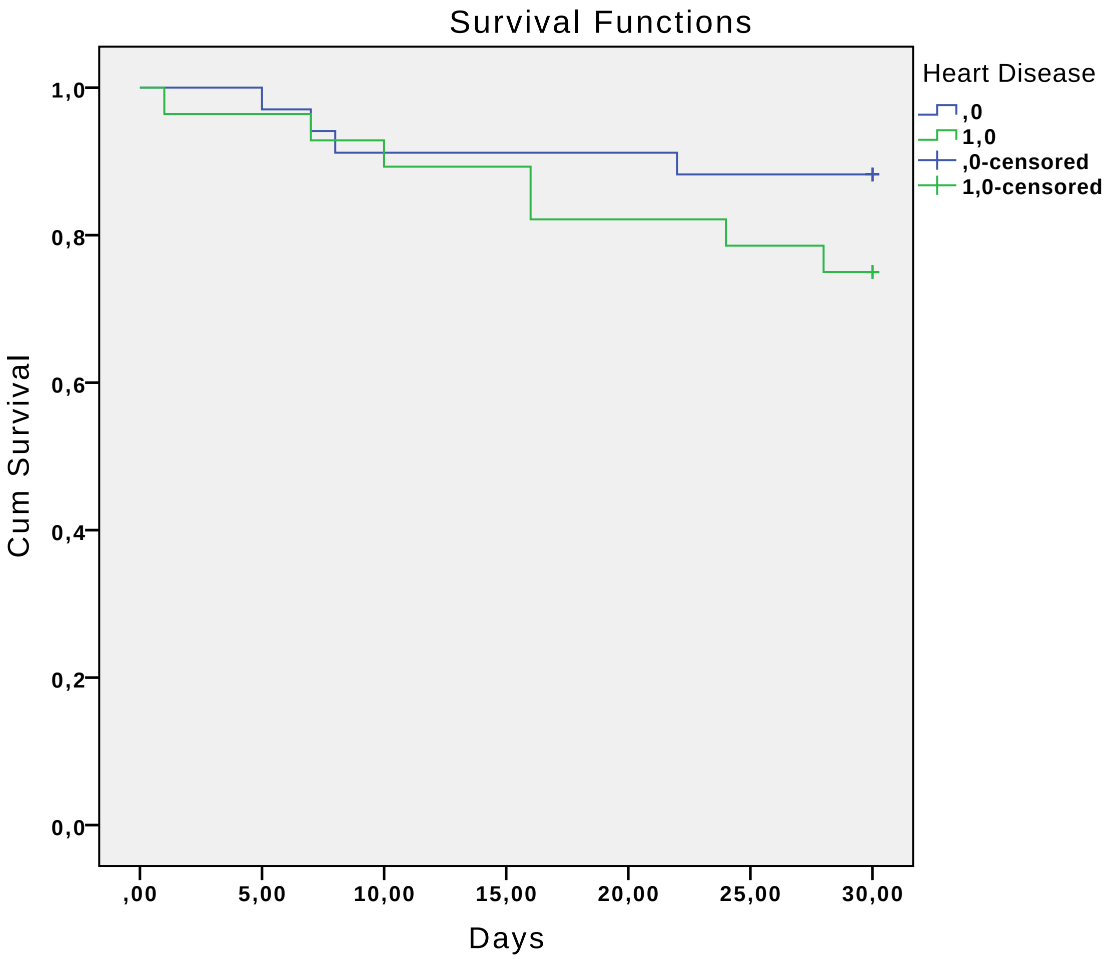

HEART FAILURE (0=NO, 1=YES)

Case Processing Summary

Heart Failure	Total N	N of Events	Censored	
			N	Percent
,0	47	6	41	87,2%
1,0	15	5	10	66,7%
Overall	62	11	51	82,3%

Survival Table

Heart Failure		Time	Status	Cumulative Proportion Surviving at the Time		N of Cumulative Events	N of Remaining Cases
				Estimate	Std. Error		
,0	1	5,000	1,00	,979	,021	1	46
	2	7,000	1,00	,957	,029	2	45
	3	8,000	1,00	,936	,036	3	44
	4	10,000	1,00	,915	,041	4	43
	5	16,000	1,00	,894	,045	5	42
	6	22,000	1,00	,872	,049	6	41
	7	30,000	,00	.	.	6	40
	8	30,000	,00	.	.	6	39
	9	30,000	,00	.	.	6	38
	10	30,000	,00	.	.	6	37
	11	30,000	,00	.	.	6	36
	12	30,000	,00	.	.	6	35
	13	30,000	,00	.	.	6	34
	14	30,000	,00	.	.	6	33
	15	30,000	,00	.	.	6	32
	16	30,000	,00	.	.	6	31
	17	30,000	,00	.	.	6	30
	18	30,000	,00	.	.	6	29
	19	30,000	,00	.	.	6	28
	20	30,000	,00	.	.	6	27
	21	30,000	,00	.	.	6	26
	22	30,000	,00	.	.	6	25
	23	30,000	,00	.	.	6	24
	24	30,000	,00	.	.	6	23
	25	30,000	,00	.	.	6	22
	26	30,000	,00	.	.	6	21
	27	30,000	,00	.	.	6	20
	28	30,000	,00	.	.	6	19
	29	30,000	,00	.	.	6	18
	30	30,000	,00	.	.	6	17
	31	30,000	,00	.	.	6	16
	32	30,000	,00	.	.	6	15
	33	30,000	,00	.	.	6	14
	34	30,000	,00	.	.	6	13
	35	30,000	,00	.	.	6	12
	36	30,000	,00	.	.	6	11
	37	30,000	,00	.	.	6	10
	38	30,000	,00	.	.	6	9
	39	30,000	,00	.	.	6	8
	40	30,000	,00	.	.	6	7
	41	30,000	,00	.	.	6	6
	42	30,000	,00	.	.	6	5
	43	30,000	,00	.	.	6	4
	44	30,000	,00	.	.	6	3
	45	30,000	,00	.	.	6	2
	46	30,000	,00	.	.	6	1
	47	30,000	,00	.	.	6	0
1,0	1	1,000	1,00	,933	,064	1	14
	2	7,000	1,00	,867	,088	2	13

Survival Table

Heart Failure	Time	Status	Cumulative Proportion Surviving at the Time		N of Cumulative Events	N of Remaining Cases
			Estimate	Std. Error		
3	16,000	1,00	,800	,103	3	12
4	24,000	1,00	,733	,114	4	11
5	28,000	1,00	,667	,122	5	10
6	30,000	,00	.	.	5	9
7	30,000	,00	.	.	5	8
8	30,000	,00	.	.	5	7
9	30,000	,00	.	.	5	6
10	30,000	,00	.	.	5	5
11	30,000	,00	.	.	5	4
12	30,000	,00	.	.	5	3
13	30,000	,00	.	.	5	2
14	30,000	,00	.	.	5	1
15	30,000	,00	.	.	5	0

Means and Medians for Survival Time

Heart Failure	Mean[a]				Median		
			95% Confidence Interval				95% …
	Estimate	Std. Error	Lower Bound	Upper Bound	Estimate	Std. Error	Lower Bound
,0	27,617	,959	25,738	29,496	.	.	.
1,0	25,067	2,348	20,464	29,670	.	.	.
Overall	27,000	,933	25,171	28,829	.	.	.

Means and Medians for Survival Time

Heart Failure	Median
	95% …
	Upper Bound
,0	.
1,0	.
Overall	.

a. Estimation is limited to the largest survival time if it is censored.

Overall Comparisons

	Chi-Square	df	Sig.
Log Rank (Mantel-Cox)	3,206	1	,073

Test of equality of survival distributions for the different levels of Heart Failure

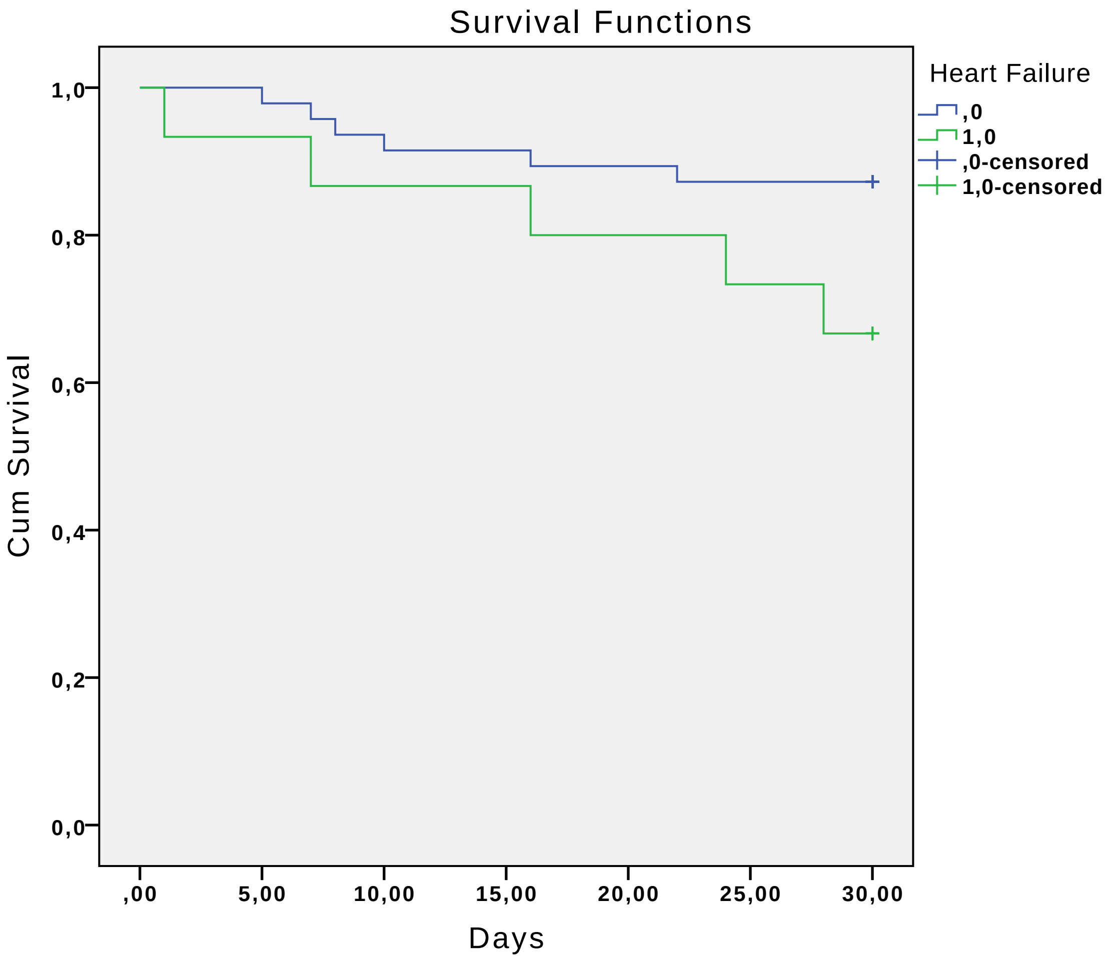

MYOCARDIAL INFARCTION (0=NO, 1=YES)

Case Processing Summary

Myocardial Infarction	Total N	N of Events	Censored	
			N	Percent
,0	52	8	44	84,6%
1,0	10	3	7	70,0%
Overall	62	11	51	82,3%

Survival Table

Myocardial Infarction		Time	Status	Cumulative Proportion Surviving at the Time		N of Cumulative Events	N of Remaining Cases
				Estimate	Std. Error		
,0	1	5,000	1,00	,981	,019	1	51
	2	7,000	1,00	.	.	2	50
	3	7,000	1,00	,942	,032	3	49
	4	8,000	1,00	,923	,037	4	48
	5	10,000	1,00	,904	,041	5	47
	6	16,000	1,00	,885	,044	6	46
	7	22,000	1,00	,865	,047	7	45
	8	28,000	1,00	,846	,050	8	44
	9	30,000	,00	.	.	8	43
	10	30,000	,00	.	.	8	42
	11	30,000	,00	.	.	8	41
	12	30,000	,00	.	.	8	40
	13	30,000	,00	.	.	8	39
	14	30,000	,00	.	.	8	38
	15	30,000	,00	.	.	8	37
	16	30,000	,00	.	.	8	36
	17	30,000	,00	.	.	8	35
	18	30,000	,00	.	.	8	34
	19	30,000	,00	.	.	8	33
	20	30,000	,00	.	.	8	32
	21	30,000	,00	.	.	8	31
	22	30,000	,00	.	.	8	30
	23	30,000	,00	.	.	8	29
	24	30,000	,00	.	.	8	28
	25	30,000	,00	.	.	8	27
	26	30,000	,00	.	.	8	26
	27	30,000	,00	.	.	8	25
	28	30,000	,00	.	.	8	24
	29	30,000	,00	.	.	8	23
	30	30,000	,00	.	.	8	22
	31	30,000	,00	.	.	8	21
	32	30,000	,00	.	.	8	20
	33	30,000	,00	.	.	8	19
	34	30,000	,00	.	.	8	18
	35	30,000	,00	.	.	8	17
	36	30,000	,00	.	.	8	16
	37	30,000	,00	.	.	8	15
	38	30,000	,00	.	.	8	14
	39	30,000	,00	.	.	8	13
	40	30,000	,00	.	.	8	12
	41	30,000	,00	.	.	8	11
	42	30,000	,00	.	.	8	10
	43	30,000	,00	.	.	8	9
	44	30,000	,00	.	.	8	8
	45	30,000	,00	.	.	8	7
	46	30,000	,00	.	.	8	6
	47	30,000	,00	.	.	8	5
	48	30,000	,00	.	.	8	4
	49	30,000	,00	.	.	8	3

Survival Table

Myocardial Infarction		Time	Status	Cumulative Proportion Surviving at the Time		N of Cumulative Events	N of Remaining Cases
				Estimate	Std. Error		
	50	30,000	,00	.	.	8	2
	51	30,000	,00	.	.	8	1
	52	30,000	,00	.	.	8	0
1,0	1	1,000	1,00	,900	,095	1	9
	2	16,000	1,00	,800	,126	2	8
	3	24,000	1,00	,700	,145	3	7
	4	30,000	,00	.	.	3	6
	5	30,000	,00	.	.	3	5
	6	30,000	,00	.	.	3	4
	7	30,000	,00	.	.	3	3
	8	30,000	,00	.	.	3	2
	9	30,000	,00	.	.	3	1
	10	30,000	,00	.	.	3	0

Means and Medians for Survival Time

Myocardial Infarction	Mean[a]				Median		
	Estimate	Std. Error	95% Confidence Interval		Estimate	Std. Error	95% …
			Lower Bound	Upper Bound			Lower Bound
,0	27,365	,956	25,492	29,239	.	.	.
1,0	25,100	2,886	19,443	30,757	.	.	.
Overall	27,000	,933	25,171	28,829	.	.	.

Means and Medians for Survival Time

Myocardial Infarction	Median
	95% …
	Upper Bound
,0	.
1,0	.
Overall	.

a. Estimation is limited to the largest survival time if it is censored.

Overall Comparisons

	Chi-Square	df	Sig.
Log Rank (Mantel-Cox)	1,240	1	,265

Test of equality of survival distributions for the different levels of Myocardial Infarction

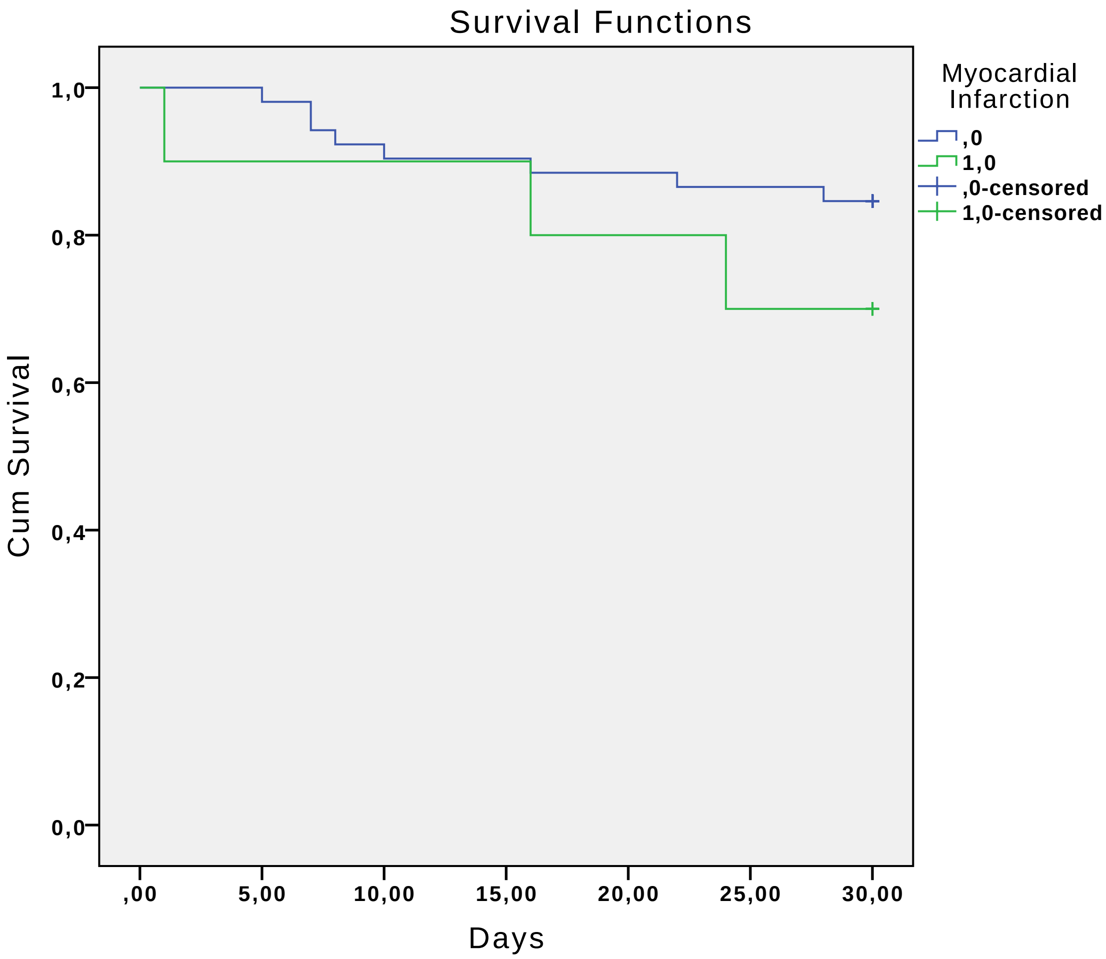

PERIPHERAL ARTERIAL DISEASE (0=NO, 1=YES)

Case Processing Summary

Peripheral Arterial Disease	Total N	N of Events	Censored	
			N	Percent
,0	51	8	43	84,3%
1,0	11	3	8	72,7%
Overall	62	11	51	82,3%

Survival Table

Peripheral Arterial Disease		Time	Status	Cumulative Proportion Surviving at the Time		N of Cumulative Events	N of Remaining Cases
				Estimate	Std. Error		
,0	1	5,000	1,00	,980	,019	1	50
	2	7,000	1,00	.	.	2	49
	3	7,000	1,00	,941	,033	3	48
	4	8,000	1,00	,922	,038	4	47
	5	10,000	1,00	,902	,042	5	46
	6	16,000	1,00	,882	,045	6	45
	7	22,000	1,00	,863	,048	7	44
	8	28,000	1,00	,843	,051	8	43
	9	30,000	,00	.	.	8	42
	10	30,000	,00	.	.	8	41
	11	30,000	,00	.	.	8	40
	12	30,000	,00	.	.	8	39
	13	30,000	,00	.	.	8	38
	14	30,000	,00	.	.	8	37
	15	30,000	,00	.	.	8	36
	16	30,000	,00	.	.	8	35
	17	30,000	,00	.	.	8	34
	18	30,000	,00	.	.	8	33
	19	30,000	,00	.	.	8	32
	20	30,000	,00	.	.	8	31
	21	30,000	,00	.	.	8	30
	22	30,000	,00	.	.	8	29
	23	30,000	,00	.	.	8	28
	24	30,000	,00	.	.	8	27
	25	30,000	,00	.	.	8	26
	26	30,000	,00	.	.	8	25
	27	30,000	,00	.	.	8	24
	28	30,000	,00	.	.	8	23
	29	30,000	,00	.	.	8	22
	30	30,000	,00	.	.	8	21
	31	30,000	,00	.	.	8	20
	32	30,000	,00	.	.	8	19
	33	30,000	,00	.	.	8	18
	34	30,000	,00	.	.	8	17
	35	30,000	,00	.	.	8	16
	36	30,000	,00	.	.	8	15
	37	30,000	,00	.	.	8	14
	38	30,000	,00	.	.	8	13
	39	30,000	,00	.	.	8	12
	40	30,000	,00	.	.	8	11
	41	30,000	,00	.	.	8	10
	42	30,000	,00	.	.	8	9
	43	30,000	,00	.	.	8	8
	44	30,000	,00	.	.	8	7
	45	30,000	,00	.	.	8	6
	46	30,000	,00	.	.	8	5
	47	30,000	,00	.	.	8	4
	48	30,000	,00	.	.	8	3
	49	30,000	,00	.	.	8	2

Survival Table

Peripheral Arterial Disease		Time	Status	Cumulative Proportion Surviving at the Time		N of Cumulative Events	N of Remaining Cases
				Estimate	Std. Error		
	50	30,000	,00	.	.	8	1
	51	30,000	,00	.	.	8	0
1,0	1	1,000	1,00	,909	,087	1	10
	2	16,000	1,00	,818	,116	2	9
	3	24,000	1,00	,727	,134	3	8
	4	30,000	,00	.	.	3	7
	5	30,000	,00	.	.	3	6
	6	30,000	,00	.	.	3	5
	7	30,000	,00	.	.	3	4
	8	30,000	,00	.	.	3	3
	9	30,000	,00	.	.	3	2
	10	30,000	,00	.	.	3	1
	11	30,000	,00	.	.	3	0

Means and Medians for Survival Time

Peripheral Arterial Disease	Mean[a]				Median		
			95% Confidence Interval				95% …
	Estimate	Std. Error	Lower Bound	Upper Bound	Estimate	Std. Error	Lower Bound
,0	27,314	,973	25,406	29,221	.	.	.
1,0	25,545	2,658	20,336	30,755	.	.	.
Overall	27,000	,933	25,171	28,829	.	.	.

Means and Medians for Survival Time

Peripheral Arterial Disease	Median
	95% …
	Upper Bound
,0	.
1,0	.
Overall	.

a. Estimation is limited to the largest survival time if it is censored.

Overall Comparisons

	Chi-Square	df	Sig.
Log Rank (Mantel-Cox)	,828	1	,363

Test of equality of survival distributions for the different levels of Peripheral Arterial Disease

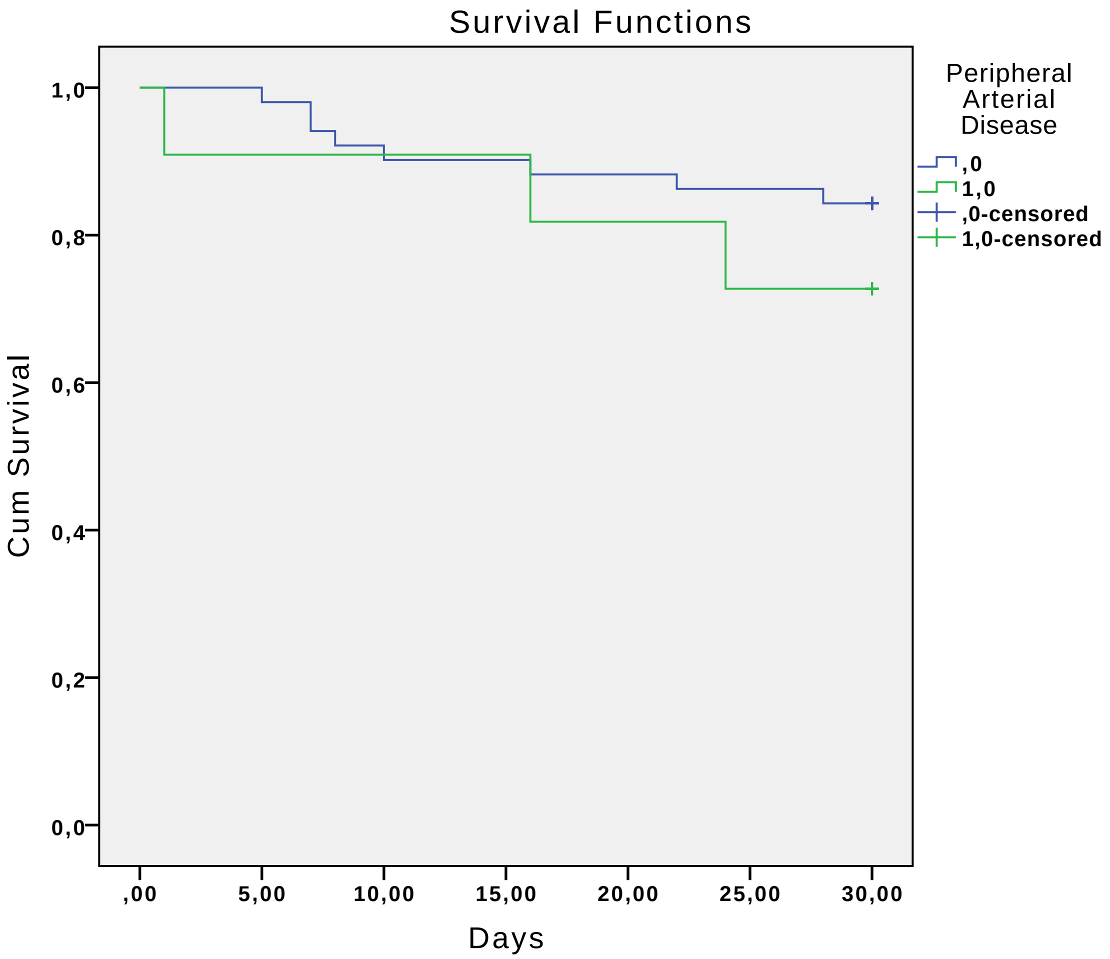

CEREBROVASCULAR DISEASE (0=NO, 1=YES)

Case Processing Summary

Cerebrovascular Disease	Total N	N of Events	Censored	
			N	Percent
,0	44	7	37	84,1%
1,0	18	4	14	77,8%
Overall	62	11	51	82,3%

Survival Table

Cerebrovascular Disease		Time	Status	Cumulative Proportion Surviving at the Time		N of Cumulative Events	N of Remaining Cases
				Estimate	Std. Error		
,0	1	1,000	1,00	,977	,022	1	43
	2	5,000	1,00	,955	,031	2	42
	3	8,000	1,00	,932	,038	3	41
	4	10,000	1,00	,909	,043	4	40
	5	16,000	1,00	,886	,048	5	39
	6	22,000	1,00	,864	,052	6	38
	7	24,000	1,00	,841	,055	7	37
	8	30,000	,00	.	.	7	36
	9	30,000	,00	.	.	7	35
	10	30,000	,00	.	.	7	34
	11	30,000	,00	.	.	7	33
	12	30,000	,00	.	.	7	32
	13	30,000	,00	.	.	7	31
	14	30,000	,00	.	.	7	30
	15	30,000	,00	.	.	7	29
	16	30,000	,00	.	.	7	28
	17	30,000	,00	.	.	7	27
	18	30,000	,00	.	.	7	26
	19	30,000	,00	.	.	7	25
	20	30,000	,00	.	.	7	24
	21	30,000	,00	.	.	7	23
	22	30,000	,00	.	.	7	22
	23	30,000	,00	.	.	7	21
	24	30,000	,00	.	.	7	20
	25	30,000	,00	.	.	7	19
	26	30,000	,00	.	.	7	18
	27	30,000	,00	.	.	7	17
	28	30,000	,00	.	.	7	16
	29	30,000	,00	.	.	7	15
	30	30,000	,00	.	.	7	14
	31	30,000	,00	.	.	7	13
	32	30,000	,00	.	.	7	12
	33	30,000	,00	.	.	7	11
	34	30,000	,00	.	.	7	10
	35	30,000	,00	.	.	7	9
	36	30,000	,00	.	.	7	8
	37	30,000	,00	.	.	7	7
	38	30,000	,00	.	.	7	6
	39	30,000	,00	.	.	7	5
	40	30,000	,00	.	.	7	4
	41	30,000	,00	.	.	7	3
	42	30,000	,00	.	.	7	2
	43	30,000	,00	.	.	7	1
	44	30,000	,00	.	.	7	0
1,0	1	7,000	1,00	.	.	1	17
	2	7,000	1,00	,889	,074	2	16
	3	16,000	1,00	,833	,088	3	15
	4	28,000	1,00	,778	,098	4	14
	5	30,000	,00	.	.	4	13

Survival Table

Cerebrovascular Disease	Time	Status	Cumulative Proportion Surviving at the Time		N of Cumulative Events	N of Remaining Cases
			Estimate	Std. Error		
6	30,000	,00	.	.	4	12
7	30,000	,00	.	.	4	11
8	30,000	,00	.	.	4	10
9	30,000	,00	.	.	4	9
10	30,000	,00	.	.	4	8
11	30,000	,00	.	.	4	7
12	30,000	,00	.	.	4	6
13	30,000	,00	.	.	4	5
14	30,000	,00	.	.	4	4
15	30,000	,00	.	.	4	3
16	30,000	,00	.	.	4	2
17	30,000	,00	.	.	4	1
18	30,000	,00	.	.	4	0

Means and Medians for Survival Time

Cerebrovascular Disease	Mean[a]				Median		
			95% Confidence Interval				95% …
	Estimate	Std. Error	Lower Bound	Upper Bound	Estimate	Std. Error	Lower Bound
,0	27,182	1,089	25,047	29,317	.	.	.
1,0	26,556	1,795	23,036	30,075	.	.	.
Overall	27,000	,933	25,171	28,829	.	.	.

Means and Medians for Survival Time

Cerebrovascular Disease	Median
	95% …
	Upper Bound
,0	.
1,0	.
Overall	.

a. Estimation is limited to the largest survival time if it is censored.

Overall Comparisons

	Chi-Square	df	Sig.
Log Rank (Mantel-Cox)	,313	1	,576

Test of equality of survival distributions for the different levels of Cerebrovascular Disease

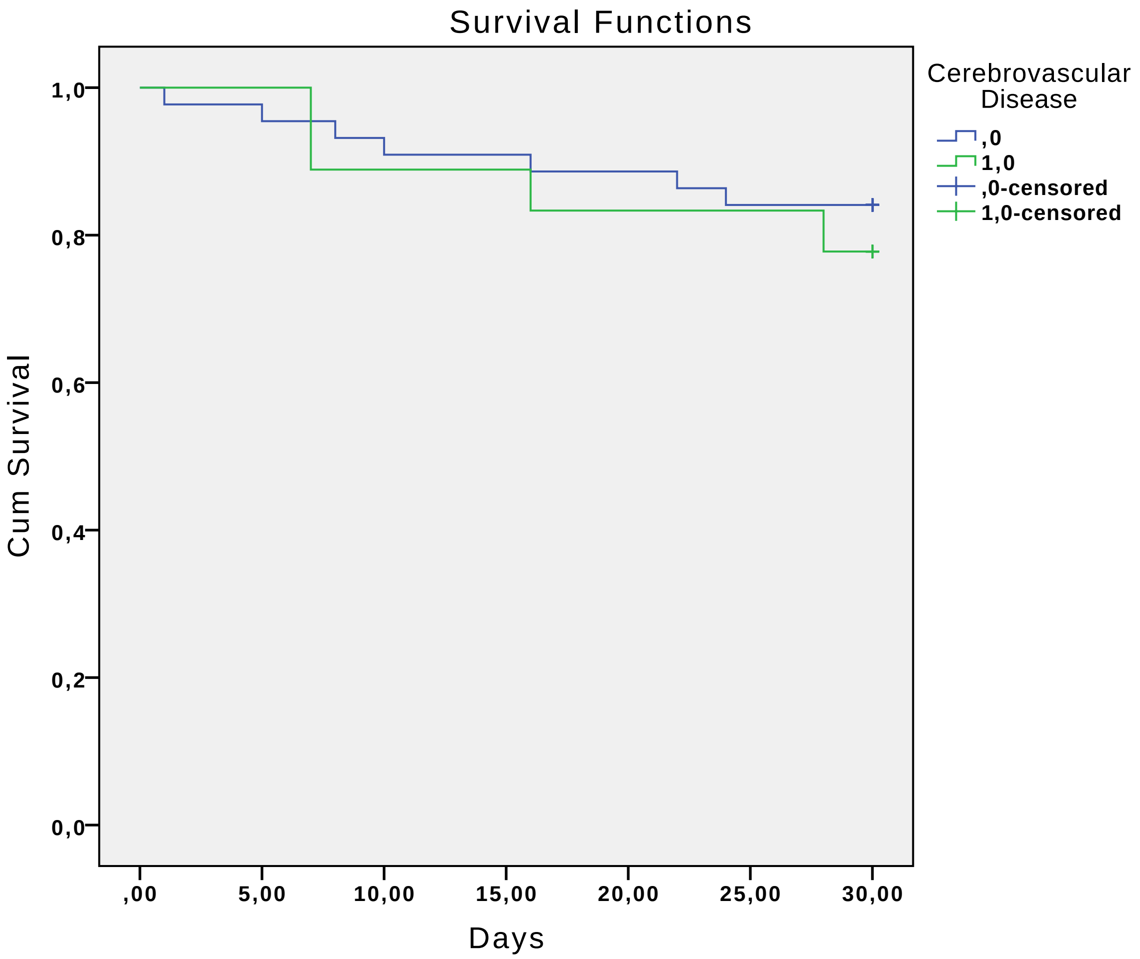

HEMIPLEGIA (0=NO, 1=YES)

Warnings

No statistics are computed because all cases are censored.

Case Processing Summary

Hemiplegia	Total N	N of Events	Censored	
			N	Percent
,0	55	11	44	80,0%
1,0	7	0	7	100,0%
Overall	62	11	51	82,3%

Survival Table

Hemiplegia		Time	Status	Cumulative Proportion Surviving at the Time		N of Cumulative Events	N of Remaining Cases
				Estimate	Std. Error		
,0	1	1,000	1,00	,982	,018	1	54
	2	5,000	1,00	,964	,025	2	53
	3	7,000	1,00	.	.	3	52
	4	7,000	1,00	,927	,035	4	51
	5	8,000	1,00	,909	,039	5	50
	6	10,000	1,00	,891	,042	6	49
	7	16,000	1,00	.	.	7	48
	8	16,000	1,00	,855	,048	8	47
	9	22,000	1,00	,836	,050	9	46
	10	24,000	1,00	,818	,052	10	45
	11	28,000	1,00	,800	,054	11	44
	12	30,000	,00	.	.	11	43
	13	30,000	,00	.	.	11	42
	14	30,000	,00	.	.	11	41
	15	30,000	,00	.	.	11	40
	16	30,000	,00	.	.	11	39
	17	30,000	,00	.	.	11	38
	18	30,000	,00	.	.	11	37
	19	30,000	,00	.	.	11	36
	20	30,000	,00	.	.	11	35
	21	30,000	,00	.	.	11	34
	22	30,000	,00	.	.	11	33
	23	30,000	,00	.	.	11	32
	24	30,000	,00	.	.	11	31
	25	30,000	,00	.	.	11	30
	26	30,000	,00	.	.	11	29
	27	30,000	,00	.	.	11	28
	28	30,000	,00	.	.	11	27
	29	30,000	,00	.	.	11	26
	30	30,000	,00	.	.	11	25
	31	30,000	,00	.	.	11	24
	32	30,000	,00	.	.	11	23
	33	30,000	,00	.	.	11	22
	34	30,000	,00	.	.	11	21
	35	30,000	,00	.	.	11	20
	36	30,000	,00	.	.	11	19
	37	30,000	,00	.	.	11	18
	38	30,000	,00	.	.	11	17
	39	30,000	,00	.	.	11	16
	40	30,000	,00	.	.	11	15
	41	30,000	,00	.	.	11	14
	42	30,000	,00	.	.	11	13
	43	30,000	,00	.	.	11	12
	44	30,000	,00	.	.	11	11
	45	30,000	,00	.	.	11	10
	46	30,000	,00	.	.	11	9
	47	30,000	,00	.	.	11	8
	48	30,000	,00	.	.	11	7
	49	30,000	,00	.	.	11	6

Survival Table

Hemiplegia		Time	Status	Cumulative Proportion Surviving at the Time		N of Cumulative Events	N of Remaining Cases
				Estimate	Std. Error		
	50	30,000	,00	.	.	11	5
	51	30,000	,00	.	.	11	4
	52	30,000	,00	.	.	11	3
	53	30,000	,00	.	.	11	2
	54	30,000	,00	.	.	11	1
	55	30,000	,00	.	.	11	0
1,0	1	30,000	,00	.	.	0	6
	2	30,000	,00	.	.	0	5
	3	30,000	,00	.	.	0	4
	4	30,000	,00	.	.	0	3
	5	30,000	,00	.	.	0	2
	6	30,000	,00	.	.	0	1

Overall Comparisons

	Chi-Square	df	Sig.
Log Rank (Mantel-Cox)	1,550	1	,213

Test of equality of survival distributions for the different levels of Hemiplegia

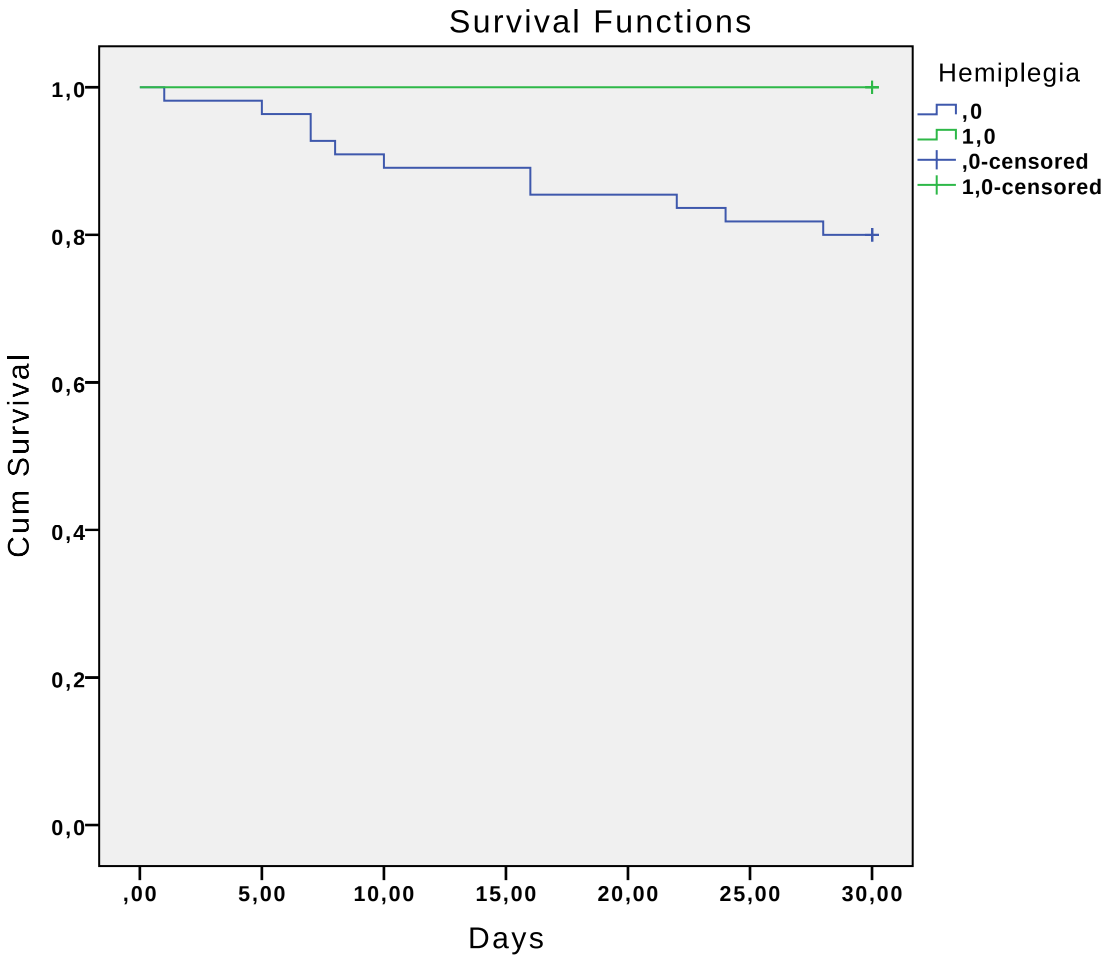

ULCUS (0=NO, 1=YES)

Case Processing Summary

Ulcus	Total N	N of Events	Censored	
			N	Percent
,0	54	10	44	81,5%
1,0	8	1	7	87,5%
Overall	62	11	51	82,3%

Survival Table

Ulcus		Time	Status	Cumulative Proportion Surviving at the Time		N of Cumulative Events	N of Remaining Cases
				Estimate	Std. Error		
,0	1	1,000	1,00	,981	,018	1	53
	2	5,000	1,00	,963	,026	2	52
	3	7,000	1,00	.	.	3	51
	4	7,000	1,00	,926	,036	4	50
	5	8,000	1,00	,907	,039	5	49
	6	10,000	1,00	,889	,043	6	48
	7	16,000	1,00	,870	,046	7	47
	8	22,000	1,00	,852	,048	8	46
	9	24,000	1,00	,833	,051	9	45
	10	28,000	1,00	,815	,053	10	44
	11	30,000	,00	.	.	10	43
	12	30,000	,00	.	.	10	42
	13	30,000	,00	.	.	10	41
	14	30,000	,00	.	.	10	40
	15	30,000	,00	.	.	10	39
	16	30,000	,00	.	.	10	38
	17	30,000	,00	.	.	10	37
	18	30,000	,00	.	.	10	36
	19	30,000	,00	.	.	10	35
	20	30,000	,00	.	.	10	34
	21	30,000	,00	.	.	10	33
	22	30,000	,00	.	.	10	32
	23	30,000	,00	.	.	10	31
	24	30,000	,00	.	.	10	30
	25	30,000	,00	.	.	10	29
	26	30,000	,00	.	.	10	28
	27	30,000	,00	.	.	10	27
	28	30,000	,00	.	.	10	26
	29	30,000	,00	.	.	10	25
	30	30,000	,00	.	.	10	24
	31	30,000	,00	.	.	10	23
	32	30,000	,00	.	.	10	22
	33	30,000	,00	.	.	10	21
	34	30,000	,00	.	.	10	20
	35	30,000	,00	.	.	10	19
	36	30,000	,00	.	.	10	18
	37	30,000	,00	.	.	10	17
	38	30,000	,00	.	.	10	16
	39	30,000	,00	.	.	10	15
	40	30,000	,00	.	.	10	14
	41	30,000	,00	.	.	10	13
	42	30,000	,00	.	.	10	12

Survival Table

Ulcus		Time	Status	Cumulative Proportion Surviving at the Time		N of Cumulative Events	N of Remaining Cases
				Estimate	Std. Error		
	43	30,000	,00	.	.	10	11
	44	30,000	,00	.	.	10	10
	45	30,000	,00	.	.	10	9
	46	30,000	,00	.	.	10	8
	47	30,000	,00	.	.	10	7
	48	30,000	,00	.	.	10	6
	49	30,000	,00	.	.	10	5
	50	30,000	,00	.	.	10	4
	51	30,000	,00	.	.	10	3
	52	30,000	,00	.	.	10	2
	53	30,000	,00	.	.	10	1
	54	30,000	,00	.	.	10	0
1,0	1	16,000	1,00	,875	,117	1	7
	2	30,000	,00	.	.	1	6
	3	30,000	,00	.	.	1	5
	4	30,000	,00	.	.	1	4
	5	30,000	,00	.	.	1	3
	6	30,000	,00	.	.	1	2
	7	30,000	,00	.	.	1	1
	8	30,000	,00	.	.	1	0

Means and Medians for Survival Time

Ulcus	Mean[a]				Median			
	Estimate	Std. Error	95% Confidence Interval		Estimate	Std. Error	95% Confidence Interval	
			Lower Bound	Upper Bound			Lower Bound	Upper Bound
,0	26,815	1,041	24,774	28,855	.	.	.	.
1,0	28,250	1,637	25,042	31,458	.	.	.	.
Overall	27,000	,933	25,171	28,829	.	.	.	.

a. Estimation is limited to the largest survival time if it is censored.

Overall Comparisons

	Chi-Square	df	Sig.
Log Rank (Mantel-Cox)	,186	1	,666

Test of equality of survival distributions for the different levels of Ulcus

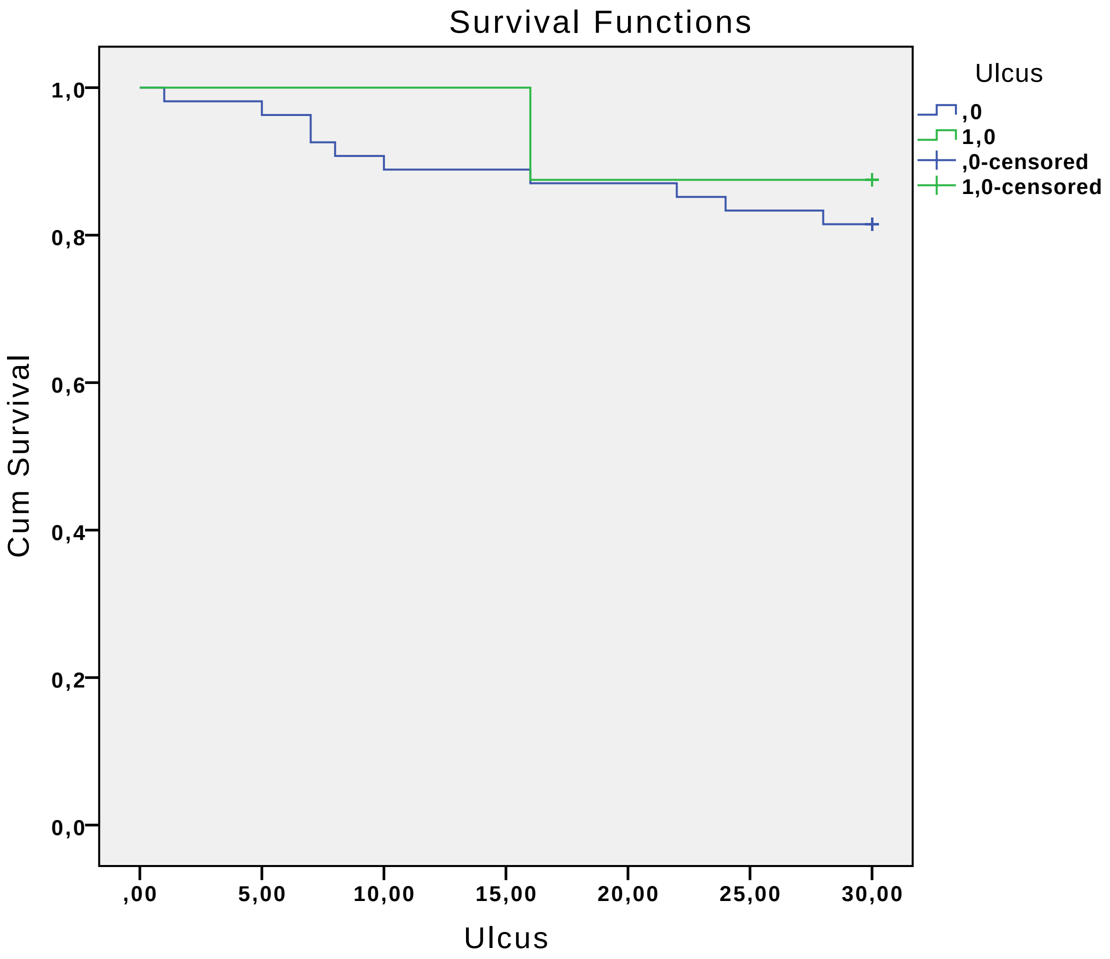

CHRONIC KIDNEY DISEASE (0=NO, 1=YES)

Case Processing Summary

Chronic Kidney Disease	Total N	N of Events	Censored	
			N	Percent
,0	49	6	43	87,8%
1,0	13	5	8	61,5%
Overall	62	11	51	82,3%

Survival Table

Chronic Kidney Disease		Time	Status	Cumulative Proportion Surviving at the Time		N of Cumulative Events	N of Remaining Cases
				Estimate	Std. Error		
,0	1	5,000	1,00	,980	,020	1	48
	2	7,000	1,00	,959	,028	2	47
	3	10,000	1,00	,939	,034	3	46
	4	16,000	1,00	,918	,039	4	45
	5	22,000	1,00	,898	,043	5	44
	6	24,000	1,00	,878	,047	6	43
	7	30,000	,00	.	.	6	42
	8	30,000	,00	.	.	6	41
	9	30,000	,00	.	.	6	40
	10	30,000	,00	.	.	6	39
	11	30,000	,00	.	.	6	38
	12	30,000	,00	.	.	6	37
	13	30,000	,00	.	.	6	36
	14	30,000	,00	.	.	6	35
	15	30,000	,00	.	.	6	34
	16	30,000	,00	.	.	6	33
	17	30,000	,00	.	.	6	32
	18	30,000	,00	.	.	6	31
	19	30,000	,00	.	.	6	30
	20	30,000	,00	.	.	6	29
	21	30,000	,00	.	.	6	28
	22	30,000	,00	.	.	6	27
	23	30,000	,00	.	.	6	26
	24	30,000	,00	.	.	6	25
	25	30,000	,00	.	.	6	24
	26	30,000	,00	.	.	6	23
	27	30,000	,00	.	.	6	22
	28	30,000	,00	.	.	6	21
	29	30,000	,00	.	.	6	20
	30	30,000	,00	.	.	6	19
	31	30,000	,00	.	.	6	18
	32	30,000	,00	.	.	6	17
	33	30,000	,00	.	.	6	16
	34	30,000	,00	.	.	6	15
	35	30,000	,00	.	.	6	14
	36	30,000	,00	.	.	6	13
	37	30,000	,00	.	.	6	12
	38	30,000	,00	.	.	6	11
	39	30,000	,00	.	.	6	10
	40	30,000	,00	.	.	6	9
	41	30,000	,00	.	.	6	8
	42	30,000	,00	.	.	6	7
	43	30,000	,00	.	.	6	6
	44	30,000	,00	.	.	6	5
	45	30,000	,00	.	.	6	4
	46	30,000	,00	.	.	6	3
	47	30,000	,00	.	.	6	2
	48	30,000	,00	.	.	6	1
	49	30,000	,00	.	.	6	0

Survival Table

Chronic Kidney Disease		Time	Status	Cumulative Proportion Surviving at the Time		N of Cumulative Events	N of Remaining Cases
				Estimate	Std. Error		
1,0	1	1,000	1,00	,923	,074	1	12
	2	7,000	1,00	,846	,100	2	11
	3	8,000	1,00	,769	,117	3	10
	4	16,000	1,00	,692	,128	4	9
	5	28,000	1,00	,615	,135	5	8
	6	30,000	,00	.	.	5	7
	7	30,000	,00	.	.	5	6
	8	30,000	,00	.	.	5	5
	9	30,000	,00	.	.	5	4
	10	30,000	,00	.	.	5	3
	11	30,000	,00	.	.	5	2
	12	30,000	,00	.	.	5	1
	13	30,000	,00	.	.	5	0

Means and Medians for Survival Time

Chronic Kidney Disease	Mean[a]				Median		
			95% Confidence Interval				95% …
	Estimate	Std. Error	Lower Bound	Upper Bound	Estimate	Std. Error	Lower Bound
,0	28,041	,832	26,410	29,671	.	.	.
1,0	23,077	2,910	17,374	28,780	.	.	.
Overall	27,000	,933	25,171	28,829	.	.	.

Means and Medians for Survival Time

Chronic Kidney Disease	Median
	95% …
	Upper Bound
,0	.
1,0	.
Overall	.

a. Estimation is limited to the largest survival time if it is censored.

Overall Comparisons

	Chi-Square	df	Sig.
Log Rank (Mantel-Cox)	5,324	1	,021

Test of equality of survival distributions for the different levels of Chronic Kidney Disease

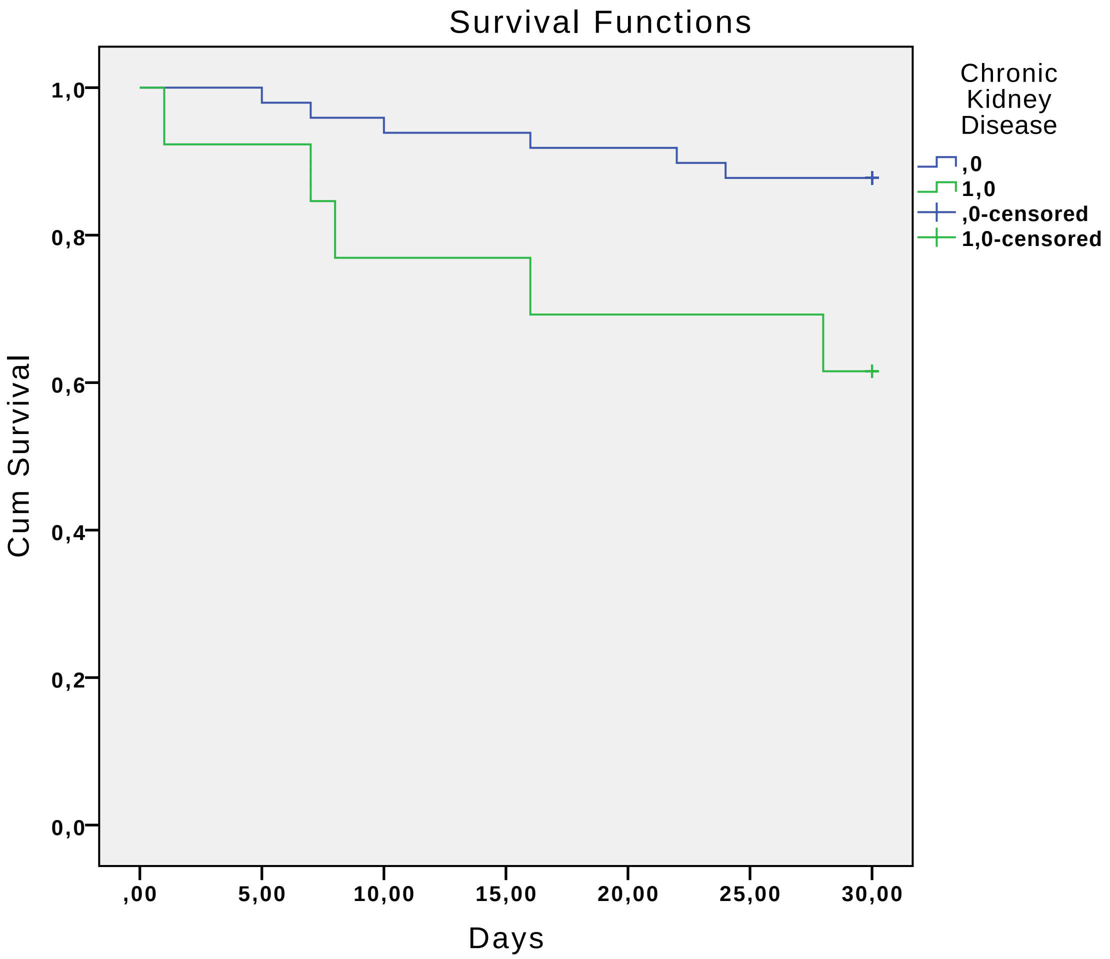

DIALYSIS (0=NO, 1=YES)

Warnings

No comparison analysis is performed because the factor variable has only one value for every stratum.

Case Processing Summary

Dialysis	Total N	N of Events	Censored	
			N	Percent
,0	62	11	51	82,3%
Overall	62	11	51	82,3%

Survival Table

Dialysis		Time	Status	Cumulative Proportion Surviving at the Time		N of Cumulative Events	N of Remaining Cases
				Estimate	Std. Error		
,0	1	1,000	1,00	,984	,016	1	61
	2	5,000	1,00	,968	,022	2	60
	3	7,000	1,00	.	.	3	59
	4	7,000	1,00	,935	,031	4	58
	5	8,000	1,00	,919	,035	5	57
	6	10,000	1,00	,903	,038	6	56
	7	16,000	1,00	.	.	7	55
	8	16,000	1,00	,871	,043	8	54
	9	22,000	1,00	,855	,045	9	53
	10	24,000	1,00	,839	,047	10	52
	11	28,000	1,00	,823	,049	11	51
	12	30,000	,00	.	.	11	50
	13	30,000	,00	.	.	11	49
	14	30,000	,00	.	.	11	48
	15	30,000	,00	.	.	11	47
	16	30,000	,00	.	.	11	46
	17	30,000	,00	.	.	11	45
	18	30,000	,00	.	.	11	44
	19	30,000	,00	.	.	11	43
	20	30,000	,00	.	.	11	42
	21	30,000	,00	.	.	11	41
	22	30,000	,00	.	.	11	40
	23	30,000	,00	.	.	11	39
	24	30,000	,00	.	.	11	38
	25	30,000	,00	.	.	11	37
	26	30,000	,00	.	.	11	36
	27	30,000	,00	.	.	11	35
	28	30,000	,00	.	.	11	34
	29	30,000	,00	.	.	11	33
	30	30,000	,00	.	.	11	32
	31	30,000	,00	.	.	11	31
	32	30,000	,00	.	.	11	30
	33	30,000	,00	.	.	11	29
	34	30,000	,00	.	.	11	28
	35	30,000	,00	.	.	11	27
	36	30,000	,00	.	.	11	26
	37	30,000	,00	.	.	11	25
	38	30,000	,00	.	.	11	24
	39	30,000	,00	.	.	11	23
	40	30,000	,00	.	.	11	22
	41	30,000	,00	.	.	11	21
	42	30,000	,00	.	.	11	20
	43	30,000	,00	.	.	11	19
	44	30,000	,00	.	.	11	18
	45	30,000	,00	.	.	11	17
	46	30,000	,00	.	.	11	16
	47	30,000	,00	.	.	11	15
	48	30,000	,00	.	.	11	14
	49	30,000	,00	.	.	11	13

Survival Table

Dialysis	Time	Status	Cumulative Proportion Surviving at the Time		N of Cumulative Events	N of Remaining Cases
			Estimate	Std. Error		
50	30,000	,00	.	.	11	12
51	30,000	,00	.	.	11	11
52	30,000	,00	.	.	11	10
53	30,000	,00	.	.	11	9
54	30,000	,00	.	.	11	8
55	30,000	,00	.	.	11	7
56	30,000	,00	.	.	11	6
57	30,000	,00	.	.	11	5
58	30,000	,00	.	.	11	4
59	30,000	,00	.	.	11	3
60	30,000	,00	.	.	11	2
61	30,000	,00	.	.	11	1
62	30,000	,00	.	.	11	0

Means and Medians for Survival Time

Dialysis	Mean[a]				Median			
	Estimate	Std. Error	95% Confidence Interval		Estimate	Std. Error	95% Confidence Interval	
			Lower Bound	Upper Bound			Lower Bound	Upper Bound
,0	27,000	,933	25,171	28,829	.	.	.	.
Overall	27,000	,933	25,171	28,829	.	.	.	.

a. Estimation is limited to the largest survival time if it is censored.

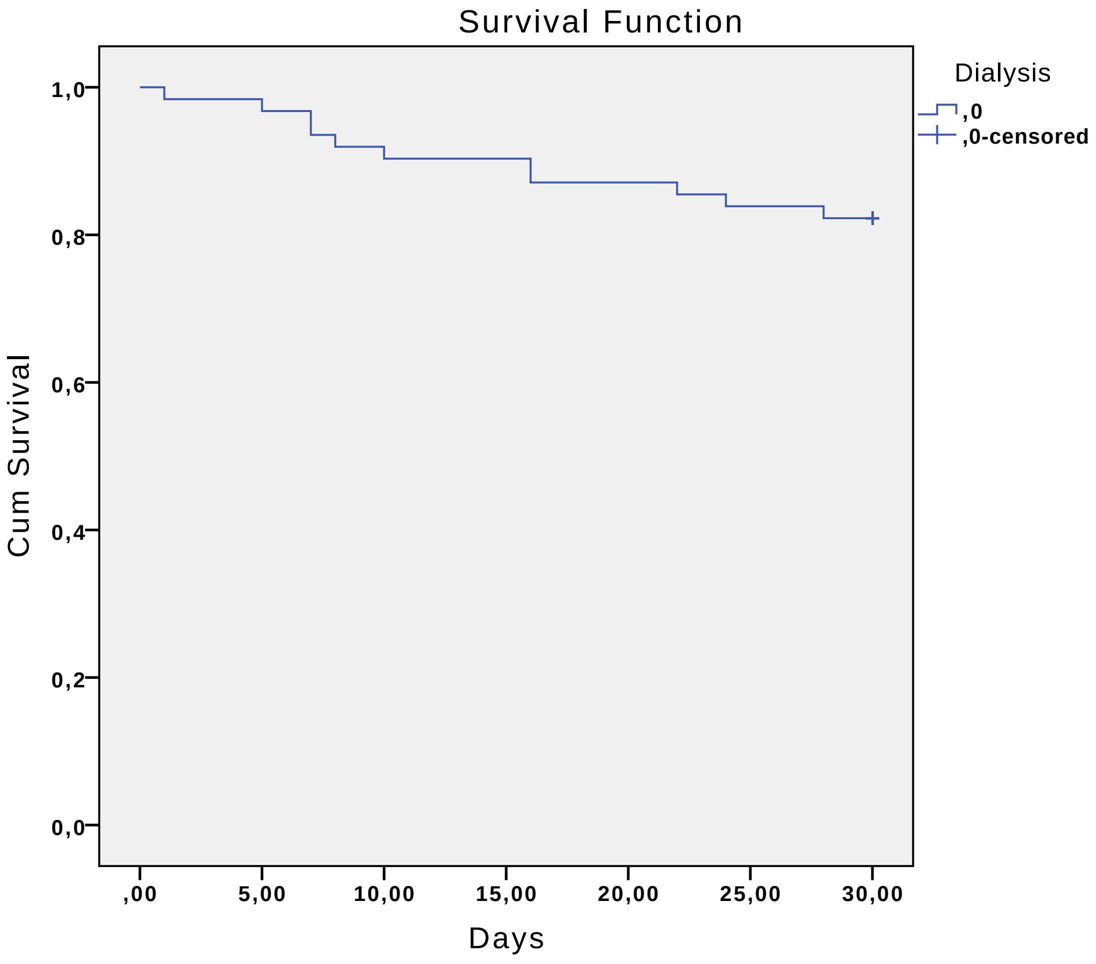

HIV (0=NO, 1=YES)

Warnings

No comparison analysis is performed because the factor variable has only one value for every stratum.

Case Processing Summary

HIV	Total N	N of Events	Censored	
			N	Percent
,0	62	11	51	82,3%
Overall	62	11	51	82,3%

Survival Table

HIV		Time	Status	Cumulative Proportion Surviving at the Time		N of Cumulative Events	N of Remaining Cases
				Estimate	Std. Error		
,0	1	1,000	1,00	,984	,016	1	61
	2	5,000	1,00	,968	,022	2	60
	3	7,000	1,00	.	.	3	59
	4	7,000	1,00	,935	,031	4	58
	5	8,000	1,00	,919	,035	5	57
	6	10,000	1,00	,903	,038	6	56
	7	16,000	1,00	.	.	7	55
	8	16,000	1,00	,871	,043	8	54
	9	22,000	1,00	,855	,045	9	53
	10	24,000	1,00	,839	,047	10	52
	11	28,000	1,00	,823	,049	11	51
	12	30,000	,00	.	.	11	50
	13	30,000	,00	.	.	11	49
	14	30,000	,00	.	.	11	48
	15	30,000	,00	.	.	11	47
	16	30,000	,00	.	.	11	46
	17	30,000	,00	.	.	11	45
	18	30,000	,00	.	.	11	44
	19	30,000	,00	.	.	11	43
	20	30,000	,00	.	.	11	42
	21	30,000	,00	.	.	11	41
	22	30,000	,00	.	.	11	40
	23	30,000	,00	.	.	11	39
	24	30,000	,00	.	.	11	38
	25	30,000	,00	.	.	11	37
	26	30,000	,00	.	.	11	36
	27	30,000	,00	.	.	11	35
	28	30,000	,00	.	.	11	34
	29	30,000	,00	.	.	11	33
	30	30,000	,00	.	.	11	32
	31	30,000	,00	.	.	11	31
	32	30,000	,00	.	.	11	30
	33	30,000	,00	.	.	11	29
	34	30,000	,00	.	.	11	28
	35	30,000	,00	.	.	11	27
	36	30,000	,00	.	.	11	26

Survival Table

HIV	Time	Status	Cumulative Proportion Surviving at the Time		N of Cumulative Events	N of Remaining Cases
			Estimate	Std. Error		
37	30,000	,00	.	.	11	25
38	30,000	,00	.	.	11	24
39	30,000	,00	.	.	11	23
40	30,000	,00	.	.	11	22
41	30,000	,00	.	.	11	21
42	30,000	,00	.	.	11	20
43	30,000	,00	.	.	11	19
44	30,000	,00	.	.	11	18
45	30,000	,00	.	.	11	17
46	30,000	,00	.	.	11	16
47	30,000	,00	.	.	11	15
48	30,000	,00	.	.	11	14
49	30,000	,00	.	.	11	13
50	30,000	,00	.	.	11	12
51	30,000	,00	.	.	11	11
52	30,000	,00	.	.	11	10
53	30,000	,00	.	.	11	9
54	30,000	,00	.	.	11	8
55	30,000	,00	.	.	11	7
56	30,000	,00	.	.	11	6
57	30,000	,00	.	.	11	5
58	30,000	,00	.	.	11	4
59	30,000	,00	.	.	11	3
60	30,000	,00	.	.	11	2
61	30,000	,00	.	.	11	1
62	30,000	,00	.	.	11	0

Means and Medians for Survival Time

HIV	Mean[a]				Median			
	Estimate	Std. Error	95% Confidence Interval		Estimate	Std. Error	95% Confidence Interval	
			Lower Bound	Upper Bound			Lower Bound	Upper Bound
,0	27,000	,933	25,171	28,829	.	.	.	.
Overall	27,000	,933	25,171	28,829	.	.	.	.

a. Estimation is limited to the largest survival time if it is censored.

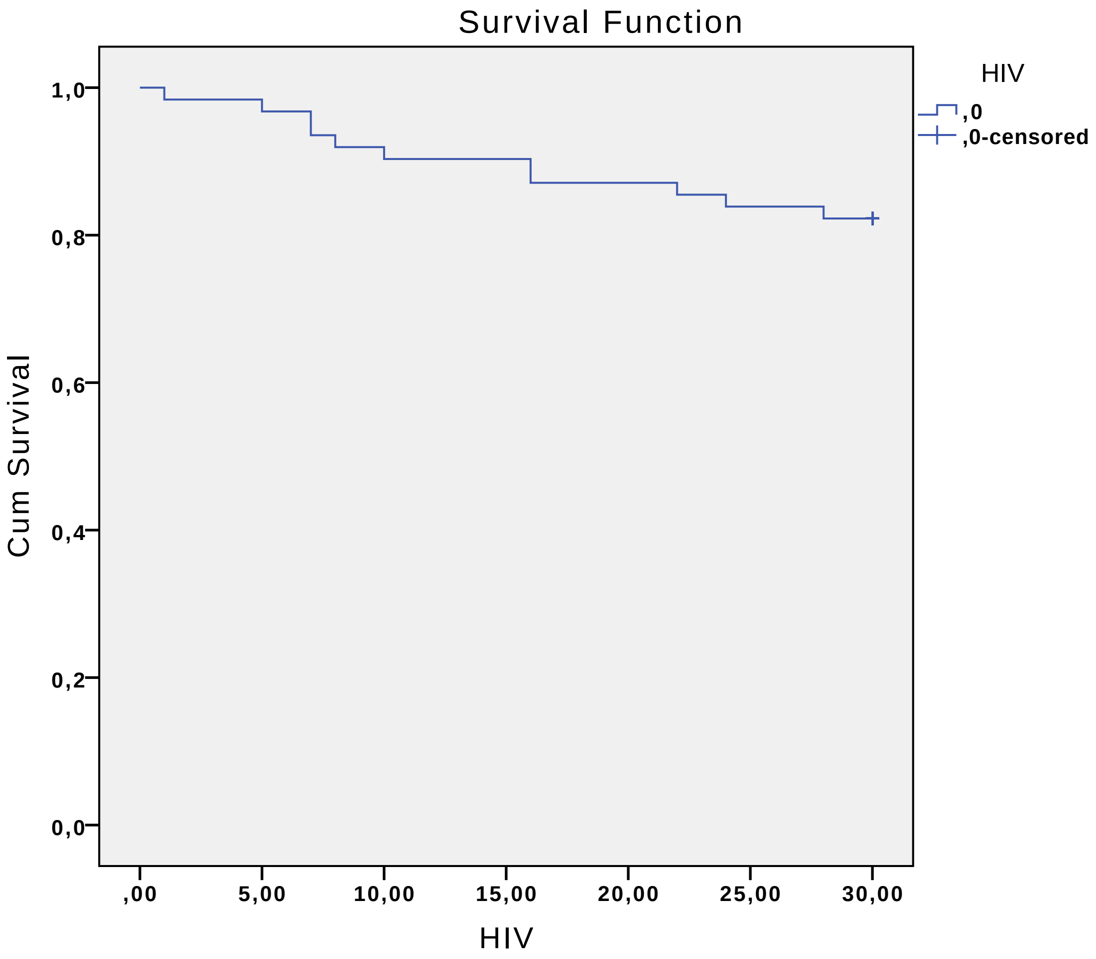

AIDS (0=NO, 1=YES)

Warnings

No comparison analysis is performed because the factor variable has only one value for every stratum.

Case Processing Summary

AIDS	Total N	N of Events	Censored	
			N	Percent
,0	62	11	51	82,3%
Overall	62	11	51	82,3%

Survival Table

AIDS		Time	Status	Cumulative Proportion Surviving at the Time		N of Cumulative Events	N of Remaining Cases
				Estimate	Std. Error		
,0	1	1,000	1,00	,984	,016	1	61
	2	5,000	1,00	,968	,022	2	60
	3	7,000	1,00	.	.	3	59
	4	7,000	1,00	,935	,031	4	58
	5	8,000	1,00	,919	,035	5	57
	6	10,000	1,00	,903	,038	6	56
	7	16,000	1,00	.	.	7	55
	8	16,000	1,00	,871	,043	8	54
	9	22,000	1,00	,855	,045	9	53
	10	24,000	1,00	,839	,047	10	52
	11	28,000	1,00	,823	,049	11	51
	12	30,000	,00	.	.	11	50
	13	30,000	,00	.	.	11	49
	14	30,000	,00	.	.	11	48
	15	30,000	,00	.	.	11	47
	16	30,000	,00	.	.	11	46
	17	30,000	,00	.	.	11	45
	18	30,000	,00	.	.	11	44
	19	30,000	,00	.	.	11	43
	20	30,000	,00	.	.	11	42
	21	30,000	,00	.	.	11	41
	22	30,000	,00	.	.	11	40
	23	30,000	,00	.	.	11	39
	24	30,000	,00	.	.	11	38
	25	30,000	,00	.	.	11	37
	26	30,000	,00	.	.	11	36
	27	30,000	,00	.	.	11	35
	28	30,000	,00	.	.	11	34
	29	30,000	,00	.	.	11	33
	30	30,000	,00	.	.	11	32
	31	30,000	,00	.	.	11	31
	32	30,000	,00	.	.	11	30
	33	30,000	,00	.	.	11	29
	34	30,000	,00	.	.	11	28
	35	30,000	,00	.	.	11	27
	36	30,000	,00	.	.	11	26
	37	30,000	,00	.	.	11	25
	38	30,000	,00	.	.	11	24
	39	30,000	,00	.	.	11	23
	40	30,000	,00	.	.	11	22
	41	30,000	,00	.	.	11	21
	42	30,000	,00	.	.	11	20
	43	30,000	,00	.	.	11	19
	44	30,000	,00	.	.	11	18
	45	30,000	,00	.	.	11	17
	46	30,000	,00	.	.	11	16
	47	30,000	,00	.	.	11	15
	48	30,000	,00	.	.	11	14
	49	30,000	,00	.	.	11	13

Survival Table

AIDS	Time	Status	Cumulative Proportion Surviving at the Time		N of Cumulative Events	N of Remaining Cases
			Estimate	Std. Error		
50	30,000	,00	.	.	11	12
51	30,000	,00	.	.	11	11
52	30,000	,00	.	.	11	10
53	30,000	,00	.	.	11	9
54	30,000	,00	.	.	11	8
55	30,000	,00	.	.	11	7
56	30,000	,00	.	.	11	6
57	30,000	,00	.	.	11	5
58	30,000	,00	.	.	11	4
59	30,000	,00	.	.	11	3
60	30,000	,00	.	.	11	2
61	30,000	,00	.	.	11	1
62	30,000	,00	.	.	11	0

Means and Medians for Survival Time

AIDS	Mean[a]				Median			
	Estimate	Std. Error	95% Confidence Interval		Estimate	Std. Error	95% Confidence Interval	
			Lower Bound	Upper Bound			Lower Bound	Upper Bound
,0	27,000	,933	25,171	28,829	.	.	.	.
Overall	27,000	,933	25,171	28,829	.	.	.	.

a. Estimation is limited to the largest survival time if it is censored.

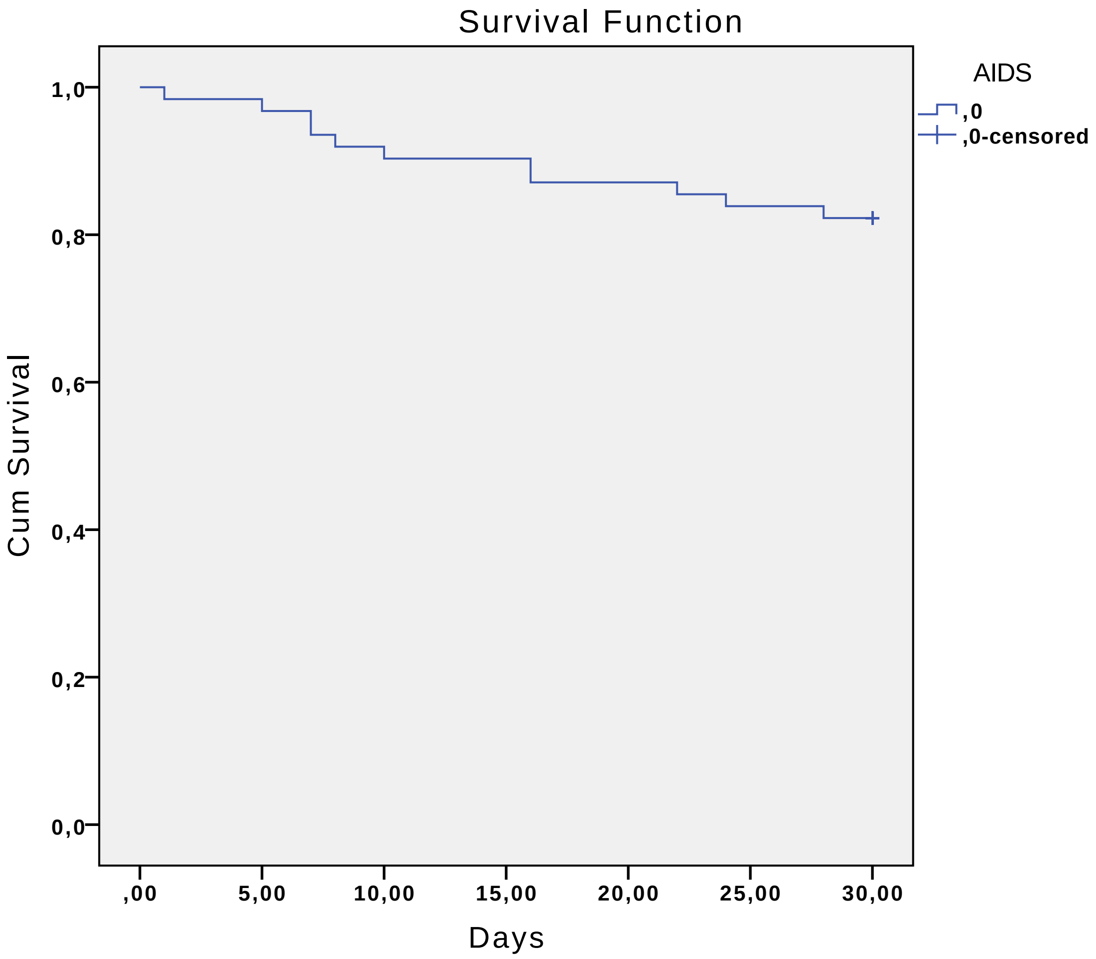

IMMUNOSUPRESSION (NEUTROPENIA/SOLID ORGAN TRANSPLANT/IMMUNOSUPRESSIVE TREATMENT) (0=NO, 1=YES)

Case Processing Summary

Immunosupression (Neutropenia/Solid Organ Transplant/Immunosupressive Treatment)	Total N	N of Events	Censored	
			N	Percent
,00	49	7	42	85,7%
1,00	13	4	9	69,2%
Overall	62	11	51	82,3%

Survival Table

Immunosupression (Neutropenia/Solid Organ Transplant/Immunosupressive Treatment)		Time	Status	Cumulative Proportion Surviving at the Time		N of Cumulative Events
				Estimate	Std. Error	
,00	1	7,000	1,00	,980	,020	1
	2	8,000	1,00	,959	,028	2
	3	10,000	1,00	,939	,034	3
	4	16,000	1,00	.	.	4
	5	16,000	1,00	,898	,043	5
	6	22,000	1,00	,878	,047	6
	7	28,000	1,00	,857	,050	7
	8	30,000	,00	.	.	7
	9	30,000	,00	.	.	7
	10	30,000	,00	.	.	7
	11	30,000	,00	.	.	7
	12	30,000	,00	.	.	7
	13	30,000	,00	.	.	7
	14	30,000	,00	.	.	7
	15	30,000	,00	.	.	7
	16	30,000	,00	.	.	7
	17	30,000	,00	.	.	7
	18	30,000	,00	.	.	7
	19	30,000	,00	.	.	7
	20	30,000	,00	.	.	7
	21	30,000	,00	.	.	7
	22	30,000	,00	.	.	7
	23	30,000	,00	.	.	7
	24	30,000	,00	.	.	7
	25	30,000	,00	.	.	7
	26	30,000	,00	.	.	7
	27	30,000	,00	.	.	7
	28	30,000	,00	.	.	7
	29	30,000	,00	.	.	7
	30	30,000	,00	.	.	7
	31	30,000	,00	.	.	7
	32	30,000	,00	.	.	7
	33	30,000	,00	.	.	7
	34	30,000	,00	.	.	7
	35	30,000	,00	.	.	7
	36	30,000	,00	.	.	7

Survival Table

Immunosupression (Neutropenia/Solid Organ Transplant/Immunosupressive Treatment)		N of Remaining Cases
,00	1	48
	2	47
	3	46
	4	45
	5	44
	6	43
	7	42
	8	41
	9	40
	10	39
	11	38
	12	37
	13	36
	14	35
	15	34
	16	33
	17	32
	18	31
	19	30
	20	29
	21	28
	22	27
	23	26
	24	25
	25	24
	26	23
	27	22
	28	21
	29	20
	30	19
	31	18
	32	17
	33	16
	34	15
	35	14
	36	13

Survival Table

Immunosupression (Neutropenia/Solid Organ Transplant/Immunosupressive Treatment)		Time	Status	Cumulative Proportion Surviving at the Time		N of Cumulative Events
				Estimate	Std. Error	
	37	30,000	,00	.	.	7
	38	30,000	,00	.	.	7
	39	30,000	,00	.	.	7
	40	30,000	,00	.	.	7
	41	30,000	,00	.	.	7
	42	30,000	,00	.	.	7
	43	30,000	,00	.	.	7
	44	30,000	,00	.	.	7
	45	30,000	,00	.	.	7
	46	30,000	,00	.	.	7
	47	30,000	,00	.	.	7
	48	30,000	,00	.	.	7
	49	30,000	,00	.	.	7
1,00	1	1,000	1,00	,923	,074	1
	2	5,000	1,00	,846	,100	2
	3	7,000	1,00	,769	,117	3
	4	24,000	1,00	,692	,128	4
	5	30,000	,00	.	.	4
	6	30,000	,00	.	.	4
	7	30,000	,00	.	.	4
	8	30,000	,00	.	.	4
	9	30,000	,00	.	.	4
	10	30,000	,00	.	.	4
	11	30,000	,00	.	.	4
	12	30,000	,00	.	.	4
	13	30,000	,00	.	.	4

Survival Table

Immunosupression (Neutropenia/Solid Organ Transplant/Immunosupressive Treatment)		N of Remaining Cases
	37	12
	38	11
	39	10
	40	9
	41	8
	42	7
	43	6
	44	5
	45	4
	46	3
	47	2
	48	1
	49	0
1,00	1	12
	2	11
	3	10
	4	9
	5	8
	6	7
	7	6
	8	5
	9	4
	10	3
	11	2
	12	1
	13	0

Means and Medians for Survival Time

Immunosupression (Neutropenia/Solid Organ Transplant/Immunosupressive Treatment)	Mean[a]				Median		
			95% Confidence Interval				95% …
	Estimate	Std. Error	Lower Bound	Upper Bound	Estimate	Std. Error	Lower Bound
,00	27,898	,831	26,270	29,526	.	.	.
1,00	23,615	2,980	17,774	29,457	.	.	.
Overall	27,000	,933	25,171	28,829	.	.	.

Means and Medians for Survival Time

Immunosupression (Neutropenia/Solid Organ Transplant/Immunosupressive Treatment)	Median
	95% …
	Upper Bound
,00	.
1,00	.
Overall	.

a. Estimation is limited to the largest survival time if it is censored.

Overall Comparisons

	Chi-Square	df	Sig.
Log Rank (Mantel-Cox)	2,378	1	,123

Test of equality of survival distributions for the different levels of Immunosupression (Neutropenia/Solid Organ Transplant/Immunosupressive Treatment)

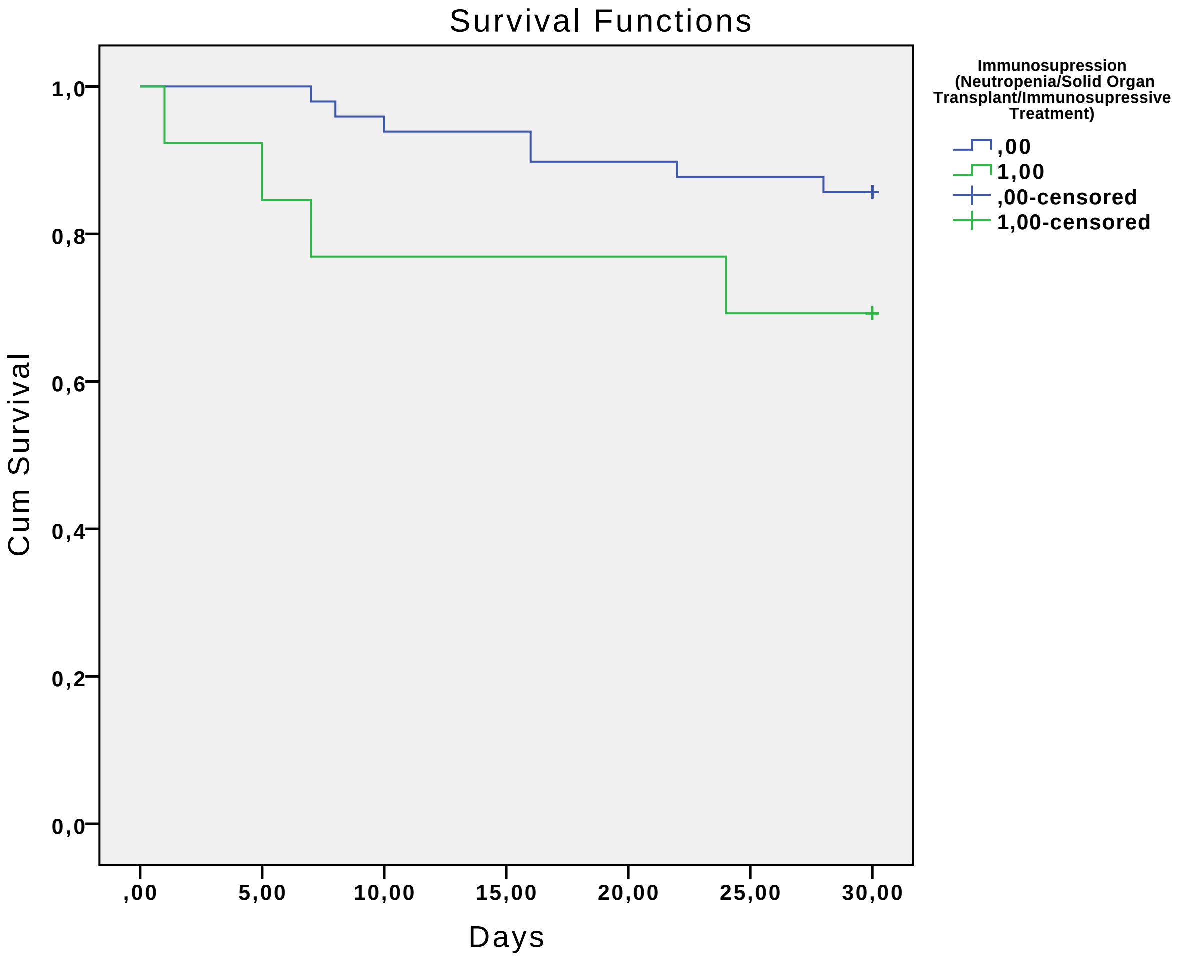

PREVIOUS 30 DAYS SURGERY (0=NO, 1=YES)

Warnings

No statistics are computed because all cases are censored.

Case Processing Summary

Previous 30 Days Surgery	Total N	N of Events	Censored	
			N	Percent
,0	46	11	35	76,1%
1,0	14	0	14	100,0%
Overall	60	11	49	81,7%

Survival Table

Previous 30 Days Surgery		Time	Status	Cumulative Proportion Surviving at the Time		N of Cumulative Events	N of Remaining Cases
				Estimate	Std. Error		
,0	1	1,000	1,00	,978	,022	1	45
	2	5,000	1,00	,957	,030	2	44
	3	7,000	1,00	.	.	3	43
	4	7,000	1,00	,913	,042	4	42
	5	8,000	1,00	,891	,046	5	41
	6	10,000	1,00	,870	,050	6	40
	7	16,000	1,00	.	.	7	39
	8	16,000	1,00	,826	,056	8	38
	9	22,000	1,00	,804	,058	9	37
	10	24,000	1,00	,783	,061	10	36
	11	28,000	1,00	,761	,063	11	35
	12	30,000	,00	.	.	11	34
	13	30,000	,00	.	.	11	33
	14	30,000	,00	.	.	11	32
	15	30,000	,00	.	.	11	31
	16	30,000	,00	.	.	11	30
	17	30,000	,00	.	.	11	29
	18	30,000	,00	.	.	11	28
	19	30,000	,00	.	.	11	27
	20	30,000	,00	.	.	11	26
	21	30,000	,00	.	.	11	25
	22	30,000	,00	.	.	11	24
	23	30,000	,00	.	.	11	23
	24	30,000	,00	.	.	11	22
	25	30,000	,00	.	.	11	21
	26	30,000	,00	.	.	11	20
	27	30,000	,00	.	.	11	19
	28	30,000	,00	.	.	11	18
	29	30,000	,00	.	.	11	17
	30	30,000	,00	.	.	11	16
	31	30,000	,00	.	.	11	15
	32	30,000	,00	.	.	11	14
	33	30,000	,00	.	.	11	13
	34	30,000	,00	.	.	11	12
	35	30,000	,00	.	.	11	11
	36	30,000	,00	.	.	11	10
	37	30,000	,00	.	.	11	9
	38	30,000	,00	.	.	11	8
	39	30,000	,00	.	.	11	7
	40	30,000	,00	.	.	11	6
	41	30,000	,00	.	.	11	5
	42	30,000	,00	.	.	11	4
	43	30,000	,00	.	.	11	3
	44	30,000	,00	.	.	11	2
	45	30,000	,00	.	.	11	1
	46	30,000	,00	.	.	11	0
1,0	1	30,000	,00	.	.	0	13
	2	30,000	,00	.	.	0	12
	3	30,000	,00	.	.	0	11

Survival Table

Previous 30 Days Surgery	Time	Status	Cumulative Proportion Surviving at the Time		N of Cumulative Events	N of Remaining Cases
			Estimate	Std. Error		
4	30,000	,00	.	.	0	10
5	30,000	,00	.	.	0	9
6	30,000	,00	.	.	0	8
7	30,000	,00	.	.	0	7
8	30,000	,00	.	.	0	6
9	30,000	,00	.	.	0	5
10	30,000	,00	.	.	0	4
11	30,000	,00	.	.	0	3
12	30,000	,00	.	.	0	2
13	30,000	,00	.	.	0	1

Overall Comparisons

	Chi-Square	df	Sig.
Log Rank (Mantel-Cox)	3,786	1	,052

Test of equality of survival distributions for the different levels of Previous 30 Days Surgery

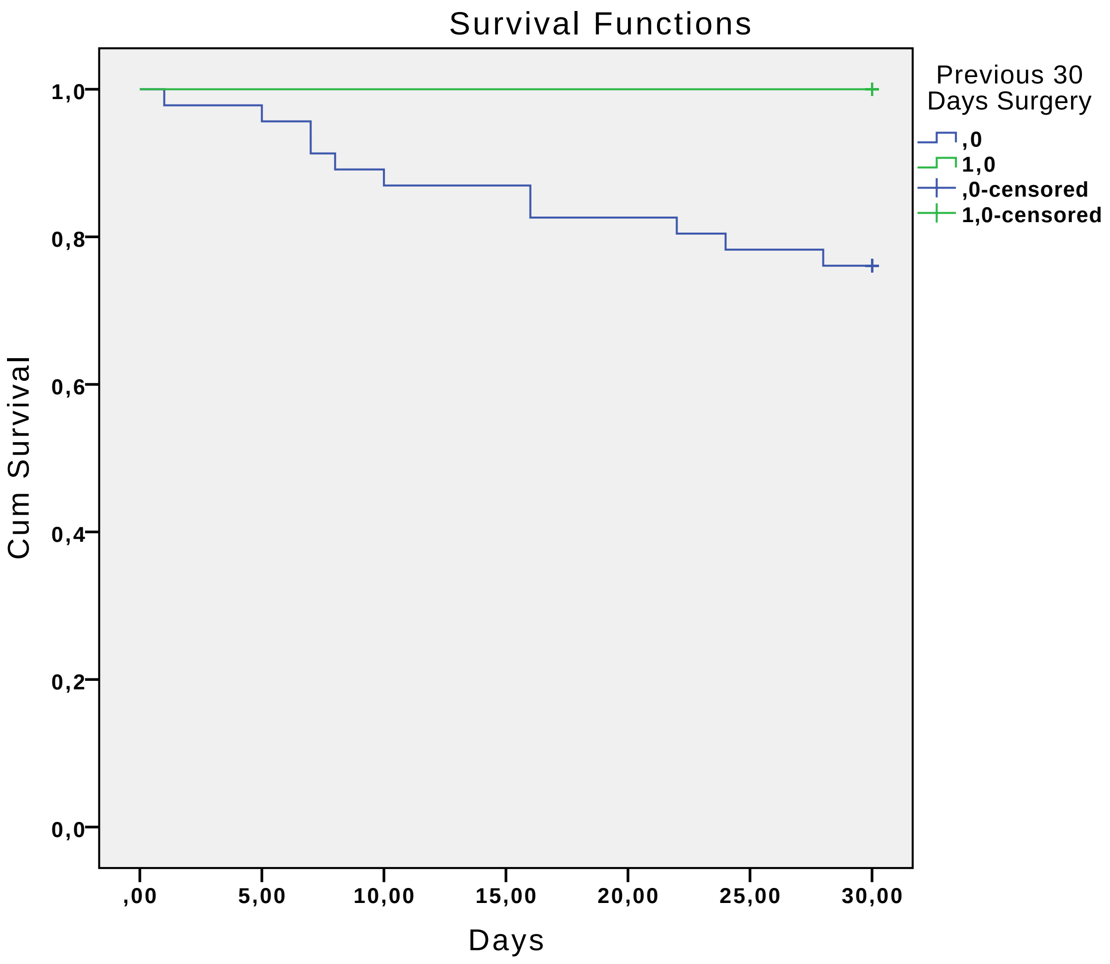

ANTIBIOTIC TREATMENT IN PREVIOUS 90 DAYS (0=NO, 1=YES)

Case Processing Summary

Antibiotic Treatment in Previous 90 Days	Total N	N of Events	Censored	
			N	Percent
,0	9	1	8	88,9%
1,0	50	10	40	80,0%
Overall	59	11	48	81,4%

Survival Table

Antibiotic Treatment in Previous 90 Days		Time	Status	Cumulative Proportion Surviving at the Time		N of Cumulative Events
				Estimate	Std. Error	
,0	1	24,000	1,00	,889	,105	1
	2	30,000	,00	.	.	1
	3	30,000	,00	.	.	1
	4	30,000	,00	.	.	1
	5	30,000	,00	.	.	1
	6	30,000	,00	.	.	1
	7	30,000	,00	.	.	1
	8	30,000	,00	.	.	1
	9	30,000	,00	.	.	1
1,0	1	1,000	1,00	,980	,020	1
	2	5,000	1,00	,960	,028	2
	3	7,000	1,00	.	.	3
	4	7,000	1,00	,920	,038	4
	5	8,000	1,00	,900	,042	5
	6	10,000	1,00	,880	,046	6
	7	16,000	1,00	.	.	7
	8	16,000	1,00	,840	,052	8
	9	22,000	1,00	,820	,054	9
	10	28,000	1,00	,800	,057	10
	11	30,000	,00	.	.	10
	12	30,000	,00	.	.	10
	13	30,000	,00	.	.	10
	14	30,000	,00	.	.	10
	15	30,000	,00	.	.	10
	16	30,000	,00	.	.	10
	17	30,000	,00	.	.	10
	18	30,000	,00	.	.	10
	19	30,000	,00	.	.	10
	20	30,000	,00	.	.	10
	21	30,000	,00	.	.	10
	22	30,000	,00	.	.	10
	23	30,000	,00	.	.	10
	24	30,000	,00	.	.	10
	25	30,000	,00	.	.	10
	26	30,000	,00	.	.	10
	27	30,000	,00	.	.	10
	28	30,000	,00	.	.	10
	29	30,000	,00	.	.	10
	30	30,000	,00	.	.	10
	31	30,000	,00	.	.	10
	32	30,000	,00	.	.	10
	33	30,000	,00	.	.	10

Survival Table

Antibiotic Treatment in Previous 90 Days		N of Remaining Cases
,0	1	8
	2	7
	3	6
	4	5
	5	4
	6	3
	7	2
	8	1
	9	0
1,0	1	49
	2	48
	3	47
	4	46
	5	45
	6	44
	7	43
	8	42
	9	41
	10	40
	11	39
	12	38
	13	37
	14	36
	15	35
	16	34
	17	33
	18	32
	19	31
	20	30
	21	29
	22	28
	23	27
	24	26
	25	25
	26	24
	27	23
	28	22
	29	21
	30	20
	31	19
	32	18
	33	17

Survival Table

Antibiotic Treatment in Previous 90 Davs	Time	Status	Cumulative Proportion Surviving at the Time		N of Cumulative Events
			Estimate	Std. Error	
34	30,000	,00	.	.	10
35	30,000	,00	.	.	10
36	30,000	,00	.	.	10
37	30,000	,00	.	.	10
38	30,000	,00	.	.	10
39	30,000	,00	.	.	10
40	30,000	,00	.	.	10
41	30,000	,00	.	.	10
42	30,000	,00	.	.	10
43	30,000	,00	.	.	10
44	30,000	,00	.	.	10
45	30,000	,00	.	.	10
46	30,000	,00	.	.	10
47	30,000	,00	.	.	10
48	30,000	,00	.	.	10
49	30,000	,00	.	.	10
50	30,000	,00	.	.	10

Survival Table

Antibiotic Treatment in Previous 90 Davs	N of Remaining Cases
34	16
35	15
36	14
37	13
38	12
39	11
40	10
41	9
42	8
43	7
44	6
45	5
46	4
47	3
48	2
49	1
50	0

Means and Medians for Survival Time

Antibiotic Treatment in Previous 90 Days	Mean[a]				Median		
			95% Confidence Interval				95% ...
	Estimate	Std. Error	Lower Bound	Upper Bound	Estimate	Std. Error	Lower Bound
,0	29,333	,629	28,101	30,565	.	.	.
1,0	26,400	1,135	24,176	28,624	.	.	.
Overall	26,847	,976	24,934	28,761	.	.	.

Means and Medians for Survival Time

Antibiotic Treatment in Previous 90 Days	Median
	95% ...
	Upper Bound
,0	.
1,0	.
Overall	.

a. Estimation is limited to the largest survival time if it is censored.

Overall Comparisons

	Chi-Square	df	Sig.
Log Rank (Mantel-Cox)	,440	1	,507

Test of equality of survival distributions for the different levels of Antibiotic Treatment in Previous 90 Days

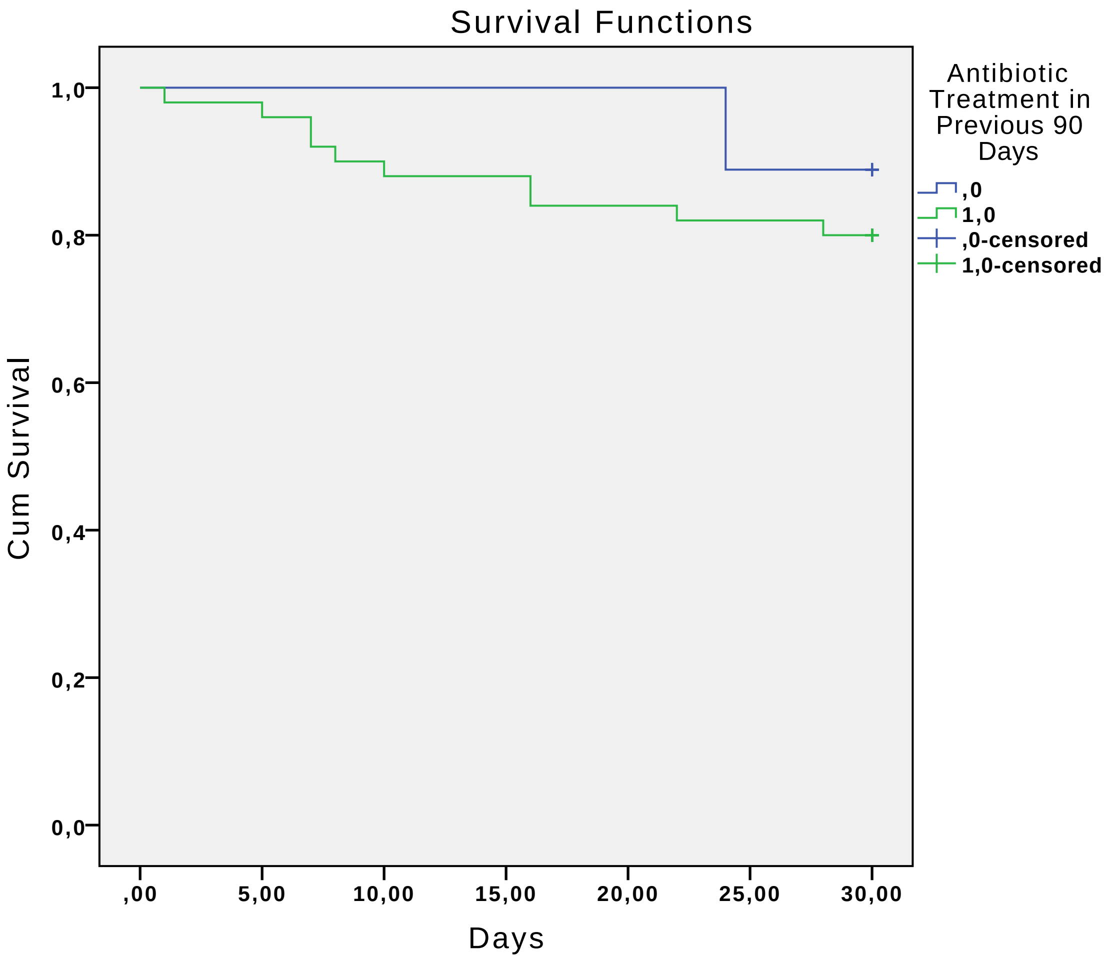

EMPIRICAL ANTIPSEUDOMONIC TREATMENT (0=NO, 1=YES)

Case Processing Summary

Empirical Antipseudomonic Treatment	Total N	N of Events	Censored	
			N	Percent
,00	38	5	33	86,8%
1,00	24	6	18	75,0%
Overall	62	11	51	82,3%

Survival Table

Empirical Antipseudomonic Treatment		Time	Status	Cumulative Proportion Surviving at the Time		N of Cumulative Events
				Estimate	Std. Error	
,00	1	5,000	1,00	,974	,026	1
	2	8,000	1,00	,947	,036	2
	3	16,000	1,00	,921	,044	3
	4	24,000	1,00	,895	,050	4
	5	28,000	1,00	,868	,055	5
	6	30,000	,00	.	.	5
	7	30,000	,00	.	.	5
	8	30,000	,00	.	.	5
	9	30,000	,00	.	.	5
	10	30,000	,00	.	.	5
	11	30,000	,00	.	.	5
	12	30,000	,00	.	.	5
	13	30,000	,00	.	.	5
	14	30,000	,00	.	.	5
	15	30,000	,00	.	.	5
	16	30,000	,00	.	.	5
	17	30,000	,00	.	.	5
	18	30,000	,00	.	.	5
	19	30,000	,00	.	.	5
	20	30,000	,00	.	.	5
	21	30,000	,00	.	.	5
	22	30,000	,00	.	.	5
	23	30,000	,00	.	.	5
	24	30,000	,00	.	.	5
	25	30,000	,00	.	.	5
	26	30,000	,00	.	.	5
	27	30,000	,00	.	.	5
	28	30,000	,00	.	.	5
	29	30,000	,00	.	.	5
	30	30,000	,00	.	.	5
	31	30,000	,00	.	.	5
	32	30,000	,00	.	.	5
	33	30,000	,00	.	.	5
	34	30,000	,00	.	.	5
	35	30,000	,00	.	.	5
	36	30,000	,00	.	.	5
	37	30,000	,00	.	.	5
	38	30,000	,00	.	.	5
1,00	1	1,000	1,00	,958	,041	1
	2	7,000	1,00	.	.	2
	3	7,000	1,00	,875	,068	3
	4	10,000	1,00	,833	,076	4
	5	16,000	1,00	,792	,083	5
	6	22,000	1,00	,750	,088	6
	7	30,000	,00	.	.	6
	8	30,000	,00	.	.	6
	9	30,000	,00	.	.	6
	10	30,000	,00	.	.	6
	11	30,000	,00	.	.	6

Survival Table

Empirical Antipseudomonic Treatment		N of Remaining Cases
,00	1	37
	2	36
	3	35
	4	34
	5	33
	6	32
	7	31
	8	30
	9	29
	10	28
	11	27
	12	26
	13	25
	14	24
	15	23
	16	22
	17	21
	18	20
	19	19
	20	18
	21	17
	22	16
	23	15
	24	14
	25	13
	26	12
	27	11
	28	10
	29	9
	30	8
	31	7
	32	6
	33	5
	34	4
	35	3
	36	2
	37	1
	38	0
1,00	1	23
	2	22
	3	21
	4	20
	5	19
	6	18
	7	17
	8	16
	9	15
	10	14
	11	13

Survival Table

Empirical Antipseudomonic Treatment	Time	Status	Cumulative Proportion Surviving at the Time		N of Cumulative Events
			Estimate	Std. Error	
12	30,000	,00	.	.	6
13	30,000	,00	.	.	6
14	30,000	,00	.	.	6
15	30,000	,00	.	.	6
16	30,000	,00	.	.	6
17	30,000	,00	.	.	6
18	30,000	,00	.	.	6
19	30,000	,00	.	.	6
20	30,000	,00	.	.	6
21	30,000	,00	.	.	6
22	30,000	,00	.	.	6
23	30,000	,00	.	.	6
24	30,000	,00	.	.	6

Survival Table

Empirical Antipseudomonic Treatment	N of Remaining Cases
12	12
13	11
14	10
15	9
16	8
17	7
18	6
19	5
20	4
21	3
22	2
23	1
24	0

Means and Medians for Survival Time

Empirical Antipseudomonic Treatment	Mean[a]				Median		
			95% Confidence Interval				95% …
	Estimate	Std. Error	Lower Bound	Upper Bound	Estimate	Std. Error	Lower Bound
,00	28,184	,919	26,383	29,986	.	.	.
1,00	25,125	1,858	21,483	28,767	.	.	.
Overall	27,000	,933	25,171	28,829	.	.	.

Means and Medians for Survival Time

Empirical Antipseudomonic Treatment	Median
	95% …
	Upper Bound
,00	.
1,00	.
Overall	.

a. Estimation is limited to the largest survival time if it is censored.

Overall Comparisons

	Chi-Square	df	Sig.
Log Rank (Mantel-Cox)	1,560	1	,212

Test of equality of survival distributions for the different levels of Empirical Antipseudomonic Treatment

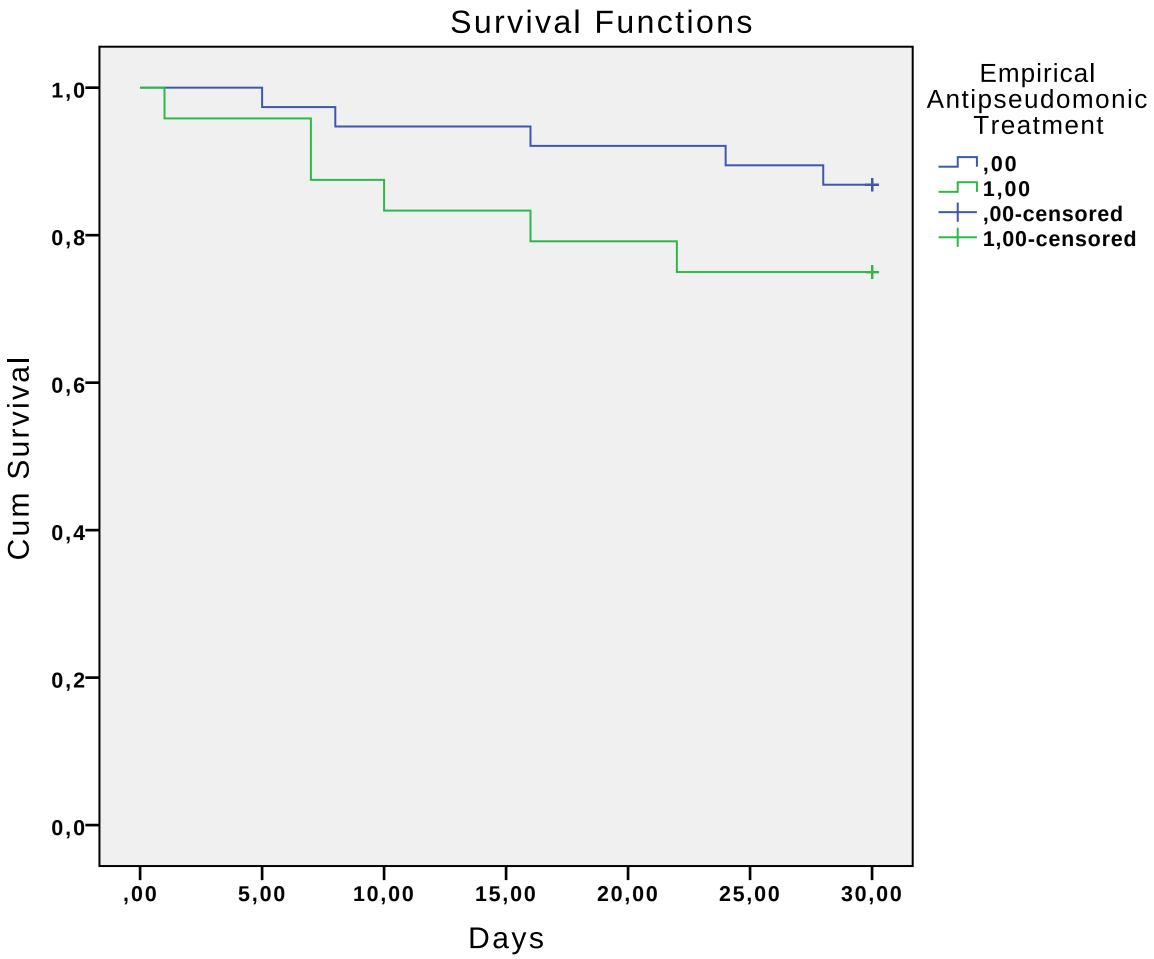

EMPIRICAL ANTIPSEUDOMONIC BITHERAPY (0=NO, 1=YES)

Warnings

No statistics are computed because all cases are censored.

Case Processing Summary

Empirical Antipseudomonic Bitherapy	Total N	N of Events	Censored	
			N	Percent
,00	59	11	48	81,4%
1,00	3	0	3	100,0%
Overall	62	11	51	82,3%

Survival Table

Empirical Antipseudomonic Bitherapy		Time	Status	Cumulative Proportion Surviving at the Time		N of Cumulative Events
				Estimate	Std. Error	
,00	1	1,000	1,00	,983	,017	1
	2	5,000	1,00	,966	,024	2
	3	7,000	1,00	.	.	3
	4	7,000	1,00	,932	,033	4
	5	8,000	1,00	,915	,036	5
	6	10,000	1,00	,898	,039	6
	7	16,000	1,00	.	.	7
	8	16,000	1,00	,864	,045	8
	9	22,000	1,00	,847	,047	9
	10	24,000	1,00	,831	,049	10
	11	28,000	1,00	,814	,051	11
	12	30,000	,00	.	.	11
	13	30,000	,00	.	.	11
	14	30,000	,00	.	.	11
	15	30,000	,00	.	.	11
	16	30,000	,00	.	.	11
	17	30,000	,00	.	.	11
	18	30,000	,00	.	.	11
	19	30,000	,00	.	.	11
	20	30,000	,00	.	.	11
	21	30,000	,00	.	.	11
	22	30,000	,00	.	.	11
	23	30,000	,00	.	.	11
	24	30,000	,00	.	.	11
	25	30,000	,00	.	.	11
	26	30,000	,00	.	.	11
	27	30,000	,00	.	.	11
	28	30,000	,00	.	.	11
	29	30,000	,00	.	.	11
	30	30,000	,00	.	.	11
	31	30,000	,00	.	.	11
	32	30,000	,00	.	.	11
	33	30,000	,00	.	.	11
	34	30,000	,00	.	.	11
	35	30,000	,00	.	.	11
	36	30,000	,00	.	.	11
	37	30,000	,00	.	.	11
	38	30,000	,00	.	.	11
	39	30,000	,00	.	.	11
	40	30,000	,00	.	.	11
	41	30,000	,00	.	.	11
	42	30,000	,00	.	.	11
	43	30,000	,00	.	.	11
	44	30,000	,00	.	.	11
	45	30,000	,00	.	.	11
	46	30,000	,00	.	.	11
	47	30,000	,00	.	.	11
	48	30,000	,00	.	.	11
	49	30,000	,00	.	.	11

Survival Table

Empirical Antipseudomonic Bitherapy		N of Remaining Cases
,00	1	58
	2	57
	3	56
	4	55
	5	54
	6	53
	7	52
	8	51
	9	50
	10	49
	11	48
	12	47
	13	46
	14	45
	15	44
	16	43
	17	42
	18	41
	19	40
	20	39
	21	38
	22	37
	23	36
	24	35
	25	34
	26	33
	27	32
	28	31
	29	30
	30	29
	31	28
	32	27
	33	26
	34	25
	35	24
	36	23
	37	22
	38	21
	39	20
	40	19
	41	18
	42	17
	43	16
	44	15
	45	14
	46	13
	47	12
	48	11
	49	10

Survival Table

Empirical Antipseudomonic Bitherapy		Time	Status	Cumulative Proportion Surviving at the Time		N of Cumulative Events
				Estimate	Std. Error	
	50	30,000	,00	.	.	11
	51	30,000	,00	.	.	11
	52	30,000	,00	.	.	11
	53	30,000	,00	.	.	11
	54	30,000	,00	.	.	11
	55	30,000	,00	.	.	11
	56	30,000	,00	.	.	11
	57	30,000	,00	.	.	11
	58	30,000	,00	.	.	11
	59	30,000	,00	.	.	11
1,00	1	30,000	,00	.	.	0
	2	30,000	,00	.	.	0

Survival Table

Empirical Antipseudomonic Bitherapy		N of Remaining Cases
	50	9
	51	8
	52	7
	53	6
	54	5
	55	4
	56	3
	57	2
	58	1
	59	0
1,00	1	2
	2	1

Overall Comparisons

	Chi-Square	df	Sig.
Log Rank (Mantel-Cox)	,615	1	,433

Test of equality of survival distributions for the different levels of Empirical Antipseudomonic Bitherapy

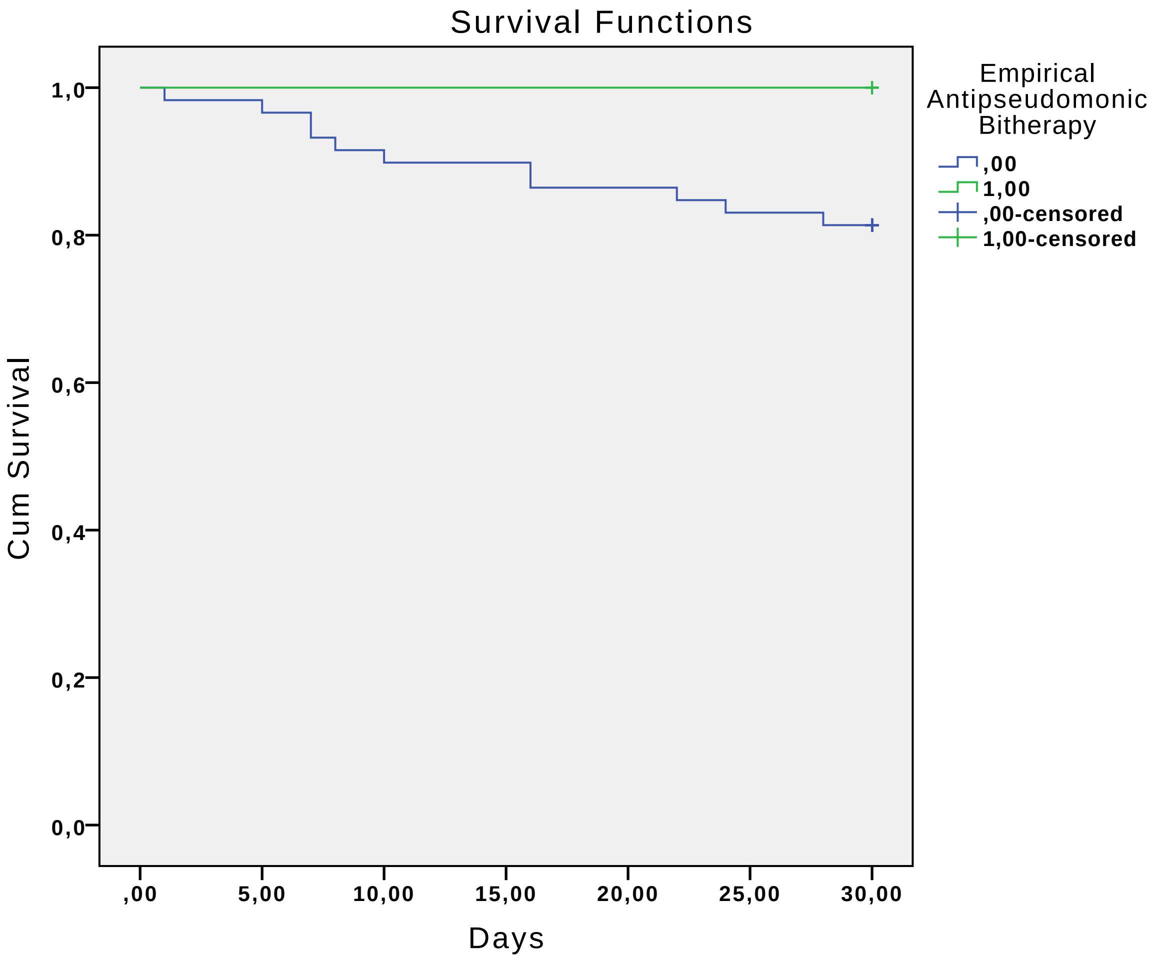

ANTIBIOTIC BITHERAPY (0=NO, 1=YES)

Case Processing Summary

Antibiotic Bitherapy	Total N	N of Events	Censored	
			N	Percent
,00	48	10	38	79,2%
1,00	14	1	13	92,9%
Overall	62	11	51	82,3%

Survival Table

Antibiotic Bitherapy		Time	Status	Cumulative Proportion Surviving at the Time		N of Cumulative Events	N of Remaining Cases
				Estimate	Std. Error		
,00	1	1,000	1,00	,979	,021	1	47
	2	5,000	1,00	,958	,029	2	46
	3	7,000	1,00	.	.	3	45
	4	7,000	1,00	,917	,040	4	44
	5	8,000	1,00	,896	,044	5	43
	6	16,000	1,00	.	.	6	42
	7	16,000	1,00	,854	,051	7	41
	8	22,000	1,00	,833	,054	8	40
	9	24,000	1,00	,813	,056	9	39
	10	28,000	1,00	,792	,059	10	38
	11	30,000	,00	.	.	10	37
	12	30,000	,00	.	.	10	36
	13	30,000	,00	.	.	10	35
	14	30,000	,00	.	.	10	34
	15	30,000	,00	.	.	10	33
	16	30,000	,00	.	.	10	32
	17	30,000	,00	.	.	10	31
	18	30,000	,00	.	.	10	30
	19	30,000	,00	.	.	10	29
	20	30,000	,00	.	.	10	28
	21	30,000	,00	.	.	10	27
	22	30,000	,00	.	.	10	26
	23	30,000	,00	.	.	10	25
	24	30,000	,00	.	.	10	24
	25	30,000	,00	.	.	10	23
	26	30,000	,00	.	.	10	22
	27	30,000	,00	.	.	10	21
	28	30,000	,00	.	.	10	20
	29	30,000	,00	.	.	10	19
	30	30,000	,00	.	.	10	18
	31	30,000	,00	.	.	10	17
	32	30,000	,00	.	.	10	16
	33	30,000	,00	.	.	10	15
	34	30,000	,00	.	.	10	14
	35	30,000	,00	.	.	10	13
	36	30,000	,00	.	.	10	12
	37	30,000	,00	.	.	10	11
	38	30,000	,00	.	.	10	10
	39	30,000	,00	.	.	10	9
	40	30,000	,00	.	.	10	8
	41	30,000	,00	.	.	10	7
	42	30,000	,00	.	.	10	6
	43	30,000	,00	.	.	10	5
	44	30,000	,00	.	.	10	4
	45	30,000	,00	.	.	10	3
	46	30,000	,00	.	.	10	2
	47	30,000	,00	.	.	10	1
	48	30,000	,00	.	.	10	0

Survival Table

Antibiotic Bitherapy		Time	Status	Cumulative Proportion Surviving at the Time: Estimate	Cumulative Proportion Surviving at the Time: Std. Error	N of Cumulative Events	N of Remaining Cases
1,00	1	10,000	1,00	,929	,069	1	13
	2	30,000	,00	.	.	1	12
	3	30,000	,00	.	.	1	11
	4	30,000	,00	.	.	1	10
	5	30,000	,00	.	.	1	9
	6	30,000	,00	.	.	1	8
	7	30,000	,00	.	.	1	7
	8	30,000	,00	.	.	1	6
	9	30,000	,00	.	.	1	5
	10	30,000	,00	.	.	1	4
	11	30,000	,00	.	.	1	3
	12	30,000	,00	.	.	1	2
	13	30,000	,00	.	.	1	1
	14	30,000	,00	.	.	1	0

Means and Medians for Survival Time

Antibiotic Bitherapy	Mean[a]				Median		
			95% Confidence Interval				95% ...
	Estimate	Std. Error	Lower Bound	Upper Bound	Estimate	Std. Error	Lower Bound
,00	26,542	1,128	24,331	28,752	.	.	.
1,00	28,571	1,377	25,873	31,270	.	.	.
Overall	27,000	,933	25,171	28,829	.	.	.

Means and Medians for Survival Time

Antibiotic Bitherapy	Median
	95% ...
	Upper Bound
,00	.
1,00	.
Overall	.

a. Estimation is limited to the largest survival time if it is censored.

Overall Comparisons

	Chi-Square	df	Sig.
Log Rank (Mantel-Cox)	1,310	1	,252

Test of equality of survival distributions for the different levels of Antibiotic Bitherapy

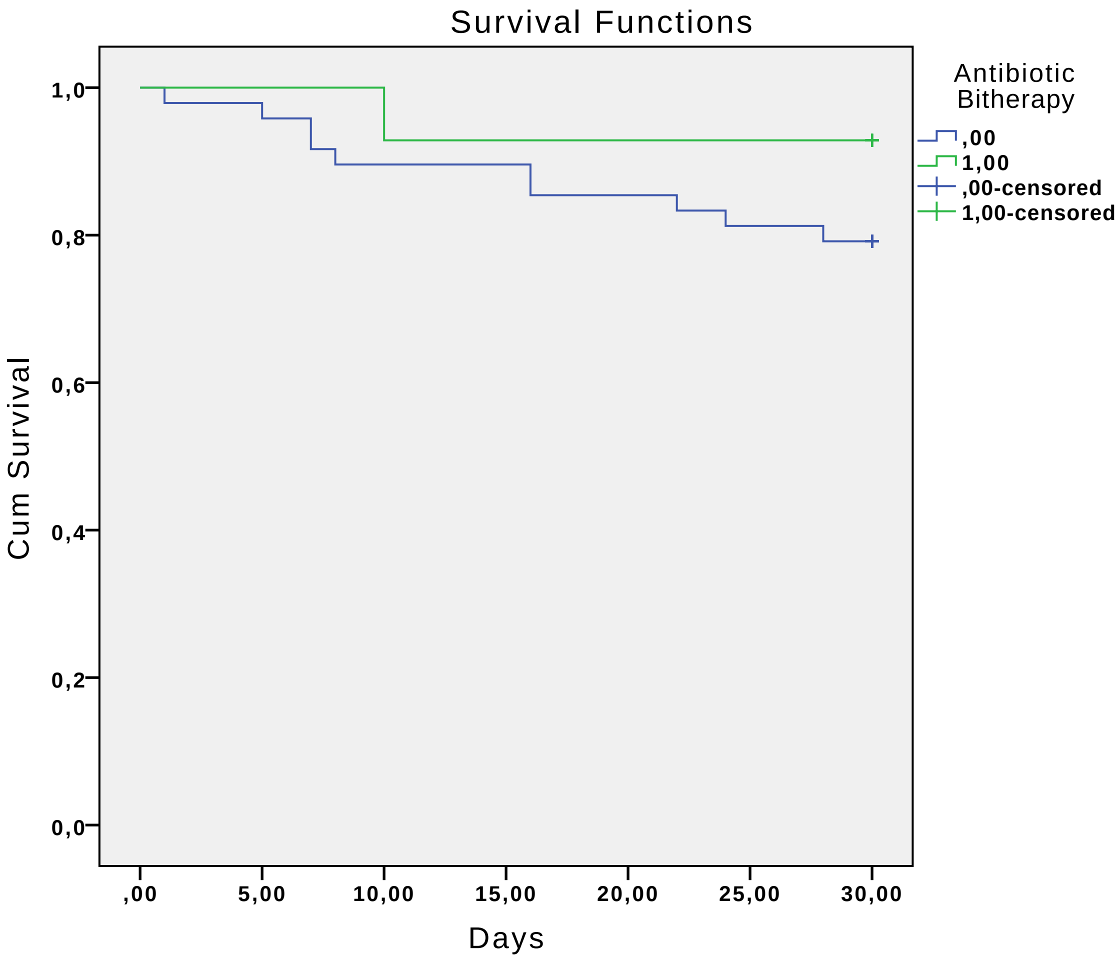

HYPOTENSION (0=NO, 1=YES)

Case Processing Summary

Hypotension	Total N	N of Events	Censored	
			N	Percent
,0	50	8	42	84,0%
1,0	11	3	8	72,7%
Overall	61	11	50	82,0%

Survival Table

Hypotension		Time	Status	Cumulative Proportion Surviving at the Time		N of Cumulative Events	N of Remaining Cases
				Estimate	Std. Error		
,0	1	1,000	1,00	,980	,020	1	49
	2	7,000	1,00	.	.	2	48

Survival Table

Hypotension	Time	Status	Cumulative Proportion Surviving at the Time		N of Cumulative Events	N of Remaining Cases
			Estimate	Std. Error		
3	7,000	1,00	,940	,034	3	47
4	10,000	1,00	,920	,038	4	46
5	16,000	1,00	.	.	5	45
6	16,000	1,00	,880	,046	6	44
7	22,000	1,00	,860	,049	7	43
8	24,000	1,00	,840	,052	8	42
9	30,000	,00	.	.	8	41
10	30,000	,00	.	.	8	40
11	30,000	,00	.	.	8	39
12	30,000	,00	.	.	8	38
13	30,000	,00	.	.	8	37
14	30,000	,00	.	.	8	36
15	30,000	,00	.	.	8	35
16	30,000	,00	.	.	8	34
17	30,000	,00	.	.	8	33
18	30,000	,00	.	.	8	32
19	30,000	,00	.	.	8	31
20	30,000	,00	.	.	8	30
21	30,000	,00	.	.	8	29
22	30,000	,00	.	.	8	28
23	30,000	,00	.	.	8	27
24	30,000	,00	.	.	8	26
25	30,000	,00	.	.	8	25
26	30,000	,00	.	.	8	24
27	30,000	,00	.	.	8	23
28	30,000	,00	.	.	8	22
29	30,000	,00	.	.	8	21
30	30,000	,00	.	.	8	20
31	30,000	,00	.	.	8	19
32	30,000	,00	.	.	8	18
33	30,000	,00	.	.	8	17
34	30,000	,00	.	.	8	16
35	30,000	,00	.	.	8	15
36	30,000	,00	.	.	8	14
37	30,000	,00	.	.	8	13
38	30,000	,00	.	.	8	12
39	30,000	,00	.	.	8	11
40	30,000	,00	.	.	8	10
41	30,000	,00	.	.	8	9
42	30,000	,00	.	.	8	8
43	30,000	,00	.	.	8	7
44	30,000	,00	.	.	8	6
45	30,000	,00	.	.	8	5
46	30,000	,00	.	.	8	4
47	30,000	,00	.	.	8	3
48	30,000	,00	.	.	8	2
49	30,000	,00	.	.	8	1
50	30,000	,00	.	.	8	0

Survival Table

Hypotension		Time	Status	Cumulative Proportion Surviving at the Time		N of Cumulative Events	N of Remaining Cases
				Estimate	Std. Error		
1,0	1	5,000	1,00	,909	,087	1	10
	2	8,000	1,00	,818	,116	2	9
	3	28,000	1,00	,727	,134	3	8
	4	30,000	,00	.	.	3	7
	5	30,000	,00	.	.	3	6
	6	30,000	,00	.	.	3	5
	7	30,000	,00	.	.	3	4
	8	30,000	,00	.	.	3	3
	9	30,000	,00	.	.	3	2
	10	30,000	,00	.	.	3	1
	11	30,000	,00	.	.	3	0

Means and Medians for Survival Time

Hypotension	Mean[a]				Median		
			95% Confidence Interval				95% …
	Estimate	Std. Error	Lower Bound	Upper Bound	Estimate	Std. Error	Lower Bound
,0	27,260	,983	25,333	29,187	.	.	.
1,0	25,545	2,719	20,216	30,875	.	.	.
Overall	26,951	,947	25,095	28,807	.	.	.

Means and Medians for Survival Time

Hypotension	Median
	95% …
	Upper Bound
,0	.
1,0	.
Overall	.

a. Estimation is limited to the largest survival time if it is censored.

Overall Comparisons

	Chi-Square	df	Sig.
Log Rank (Mantel-Cox)	,801	1	,371

Test of equality of survival distributions for the different levels of Hypotension

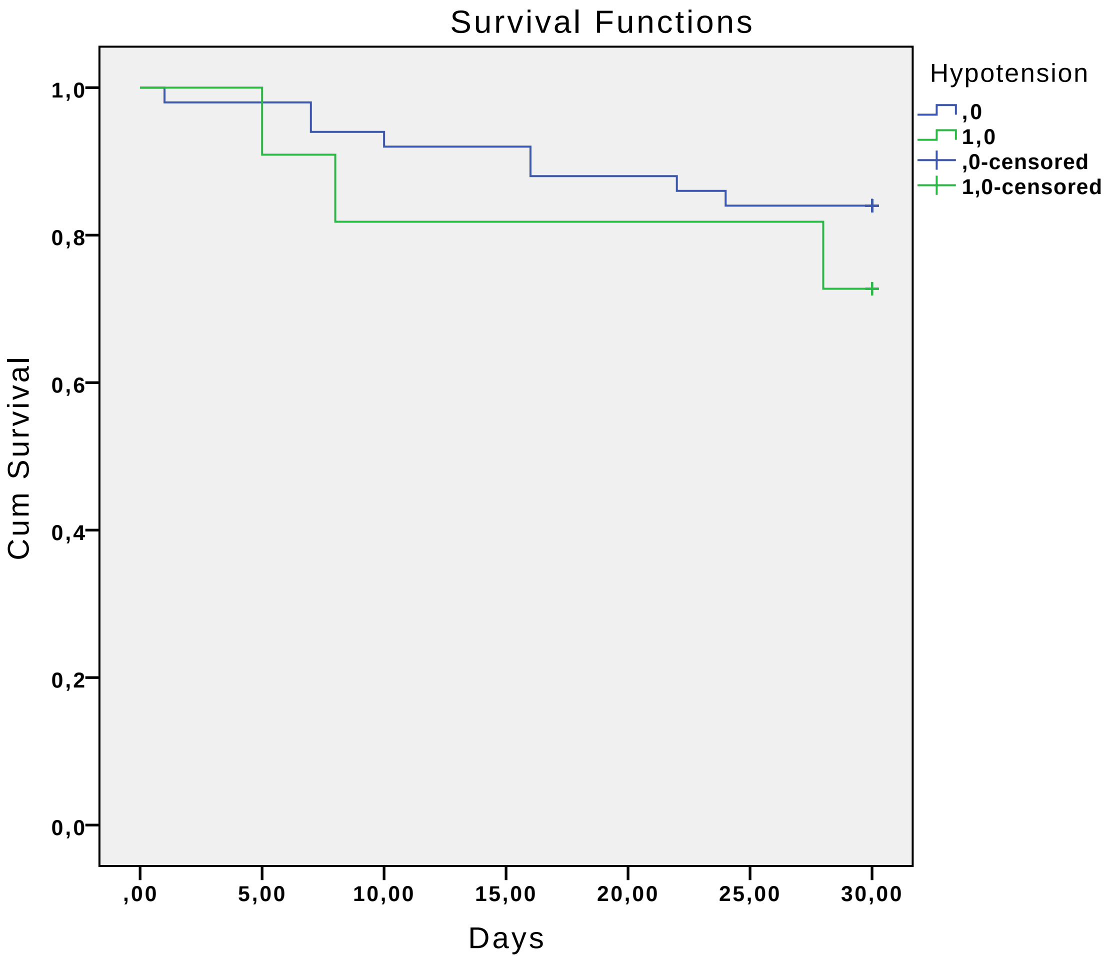

TACHYCARDIA (0=NO, 1=YES)

Case Processing Summary

Tachycardia	Total N	N of Events	Censored	
			N	Percent
,0	41	7	34	82,9%
1,0	14	3	11	78,6%
Overall	55	10	45	81,8%

Survival Table

Tachycardia		Time	Status	Cumulative Proportion Surviving at the Time		N of Cumulative Events	N of Remaining Cases
				Estimate	Std. Error		
,0	1	7,000	1,00	,976	,024	1	40
	2	8,000	1,00	,951	,034	2	39
	3	10,000	1,00	,927	,041	3	38
	4	16,000	1,00	.	.	4	37
	5	16,000	1,00	,878	,051	5	36
	6	22,000	1,00	,854	,055	6	35
	7	28,000	1,00	,829	,059	7	34
	8	30,000	,00	.	.	7	33
	9	30,000	,00	.	.	7	32
	10	30,000	,00	.	.	7	31
	11	30,000	,00	.	.	7	30
	12	30,000	,00	.	.	7	29
	13	30,000	,00	.	.	7	28
	14	30,000	,00	.	.	7	27
	15	30,000	,00	.	.	7	26
	16	30,000	,00	.	.	7	25
	17	30,000	,00	.	.	7	24
	18	30,000	,00	.	.	7	23
	19	30,000	,00	.	.	7	22
	20	30,000	,00	.	.	7	21
	21	30,000	,00	.	.	7	20
	22	30,000	,00	.	.	7	19
	23	30,000	,00	.	.	7	18
	24	30,000	,00	.	.	7	17
	25	30,000	,00	.	.	7	16
	26	30,000	,00	.	.	7	15
	27	30,000	,00	.	.	7	14
	28	30,000	,00	.	.	7	13
	29	30,000	,00	.	.	7	12
	30	30,000	,00	.	.	7	11
	31	30,000	,00	.	.	7	10
	32	30,000	,00	.	.	7	9
	33	30,000	,00	.	.	7	8
	34	30,000	,00	.	.	7	7
	35	30,000	,00	.	.	7	6
	36	30,000	,00	.	.	7	5
	37	30,000	,00	.	.	7	4
	38	30,000	,00	.	.	7	3
	39	30,000	,00	.	.	7	2
	40	30,000	,00	.	.	7	1
	41	30,000	,00	.	.	7	0
1,0	1	1,000	1,00	,929	,069	1	13
	2	7,000	1,00	,857	,094	2	12
	3	24,000	1,00	,786	,110	3	11
	4	30,000	,00	.	.	3	10
	5	30,000	,00	.	.	3	9
	6	30,000	,00	.	.	3	8
	7	30,000	,00	.	.	3	7
	8	30,000	,00	.	.	3	6

Survival Table

Tachycardia	Time	Status	Cumulative Proportion Surviving at the Time		N of Cumulative Events	N of Remaining Cases
			Estimate	Std. Error		
9	30,000	,00	.	.	3	5
10	30,000	,00	.	.	3	4
11	30,000	,00	.	.	3	3
12	30,000	,00	.	.	3	2
13	30,000	,00	.	.	3	1
14	30,000	,00	.	.	3	0

Means and Medians for Survival Time

Tachycardia	Mean[a]				Median			
	Estimate	Std. Error	95% Confidence Interval		Estimate	Std. Error	95% Confidence Interval	
			Lower Bound	Upper Bound			Lower Bound	Upper Bound
,0	27,488	,980	25,567	29,408	.	.	.	.
1,0	25,857	2,439	21,077	30,637	.	.	.	.
Overall	27,073	,963	25,184	28,961	.	.	.	.

a. Estimation is limited to the largest survival time if it is censored.

Overall Comparisons

	Chi-Square	df	Sig.
Log Rank (Mantel-Cox)	,180	1	,671

Test of equality of survival distributions for the different levels of Tachycardia

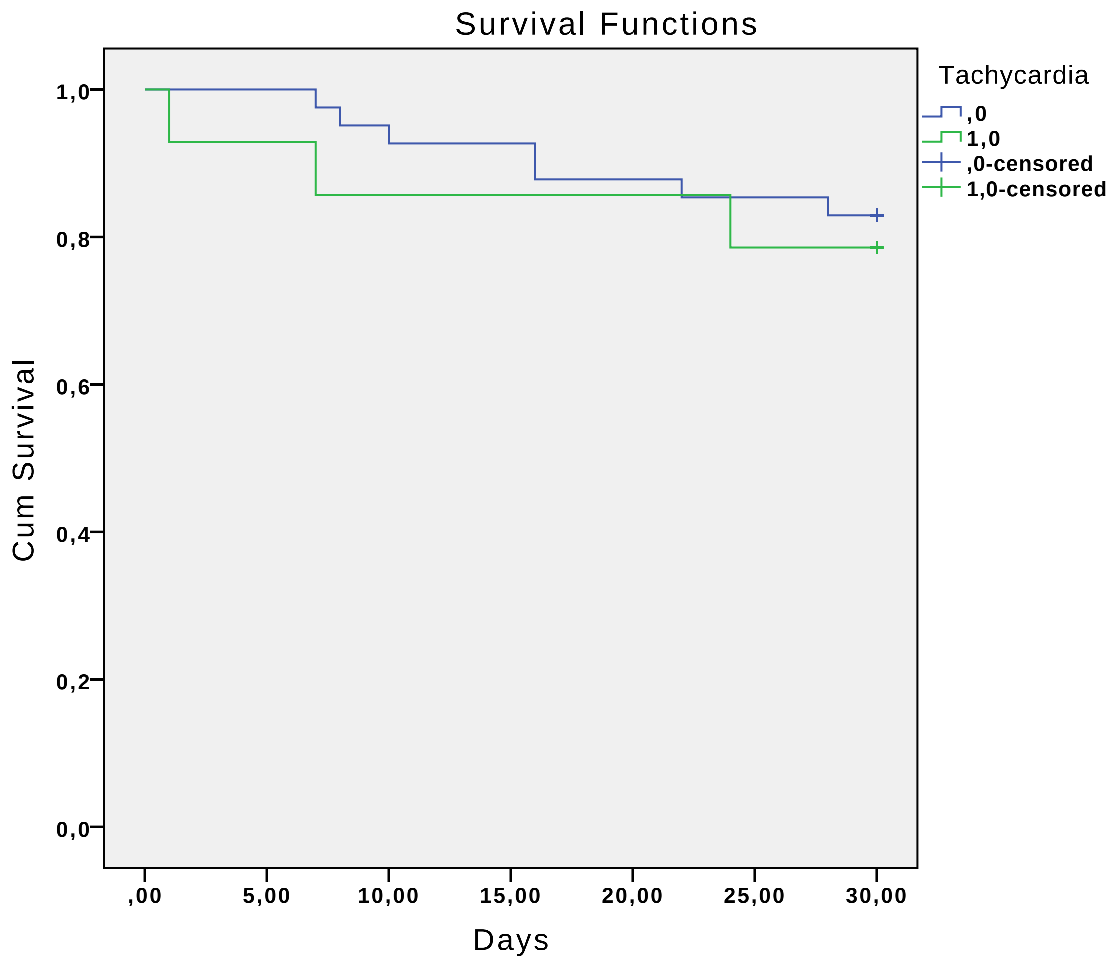

FEVER (0=NO, 1=YES)

Case Processing Summary

Fever	Total N	N of Events	Censored	
			N	Percent
,0	36	9	27	75,0%
1,0	26	2	24	92,3%
Overall	62	11	51	82,3%

Survival Table

Fever		Time	Status	Cumulative Proportion Surviving at the Time		N of Cumulative Events	N of Remaining Cases
				Estimate	Std. Error		
,0	1	1,000	1,00	,972	,027	1	35
	2	7,000	1,00	.	.	2	34
	3	7,000	1,00	,917	,046	3	33
	4	8,000	1,00	,889	,052	4	32
	5	10,000	1,00	,861	,058	5	31
	6	16,000	1,00	,833	,062	6	30
	7	22,000	1,00	,806	,066	7	29
	8	24,000	1,00	,778	,069	8	28
	9	28,000	1,00	,750	,072	9	27
	10	30,000	,00	.	.	9	26
	11	30,000	,00	.	.	9	25
	12	30,000	,00	.	.	9	24
	13	30,000	,00	.	.	9	23
	14	30,000	,00	.	.	9	22
	15	30,000	,00	.	.	9	21
	16	30,000	,00	.	.	9	20
	17	30,000	,00	.	.	9	19
	18	30,000	,00	.	.	9	18
	19	30,000	,00	.	.	9	17
	20	30,000	,00	.	.	9	16
	21	30,000	,00	.	.	9	15
	22	30,000	,00	.	.	9	14
	23	30,000	,00	.	.	9	13
	24	30,000	,00	.	.	9	12
	25	30,000	,00	.	.	9	11
	26	30,000	,00	.	.	9	10
	27	30,000	,00	.	.	9	9
	28	30,000	,00	.	.	9	8
	29	30,000	,00	.	.	9	7
	30	30,000	,00	.	.	9	6
	31	30,000	,00	.	.	9	5
	32	30,000	,00	.	.	9	4
	33	30,000	,00	.	.	9	3
	34	30,000	,00	.	.	9	2
	35	30,000	,00	.	.	9	1
	36	30,000	,00	.	.	9	0
1,0	1	5,000	1,00	,962	,038	1	25
	2	16,000	1,00	,923	,052	2	24
	3	30,000	,00	.	.	2	23

Survival Table

Fever	Time	Status	Cumulative Proportion Surviving at the Time		N of Cumulative Events	N of Remaining Cases
			Estimate	Std. Error		
4	30,000	,00	.	.	2	22
5	30,000	,00	.	.	2	21
6	30,000	,00	.	.	2	20
7	30,000	,00	.	.	2	19
8	30,000	,00	.	.	2	18
9	30,000	,00	.	.	2	17
10	30,000	,00	.	.	2	16
11	30,000	,00	.	.	2	15
12	30,000	,00	.	.	2	14
13	30,000	,00	.	.	2	13
14	30,000	,00	.	.	2	12
15	30,000	,00	.	.	2	11
16	30,000	,00	.	.	2	10
17	30,000	,00	.	.	2	9
18	30,000	,00	.	.	2	8
19	30,000	,00	.	.	2	7
20	30,000	,00	.	.	2	6
21	30,000	,00	.	.	2	5
22	30,000	,00	.	.	2	4
23	30,000	,00	.	.	2	3
24	30,000	,00	.	.	2	2
25	30,000	,00	.	.	2	1
26	30,000	,00	.	.	2	0

Means and Medians for Survival Time

Fever	Mean[a]				Median			
	Estimate	Std. Error	95% Confidence Interval		Estimate	Std. Error	95% Confidence Interval	
			Lower Bound	Upper Bound			Lower Bound	Upper Bound
,0	25,917	1,384	23,204	28,629	.	.	.	.
1,0	28,500	1,062	26,418	30,582	.	.	.	.
Overall	27,000	,933	25,171	28,829	.	.	.	.

a. Estimation is limited to the largest survival time if it is censored.

Overall Comparisons

	Chi-Square	df	Sig.
Log Rank (Mantel-Cox)	2,919	1	,088

Test of equality of survival distributions for the different levels of Fever

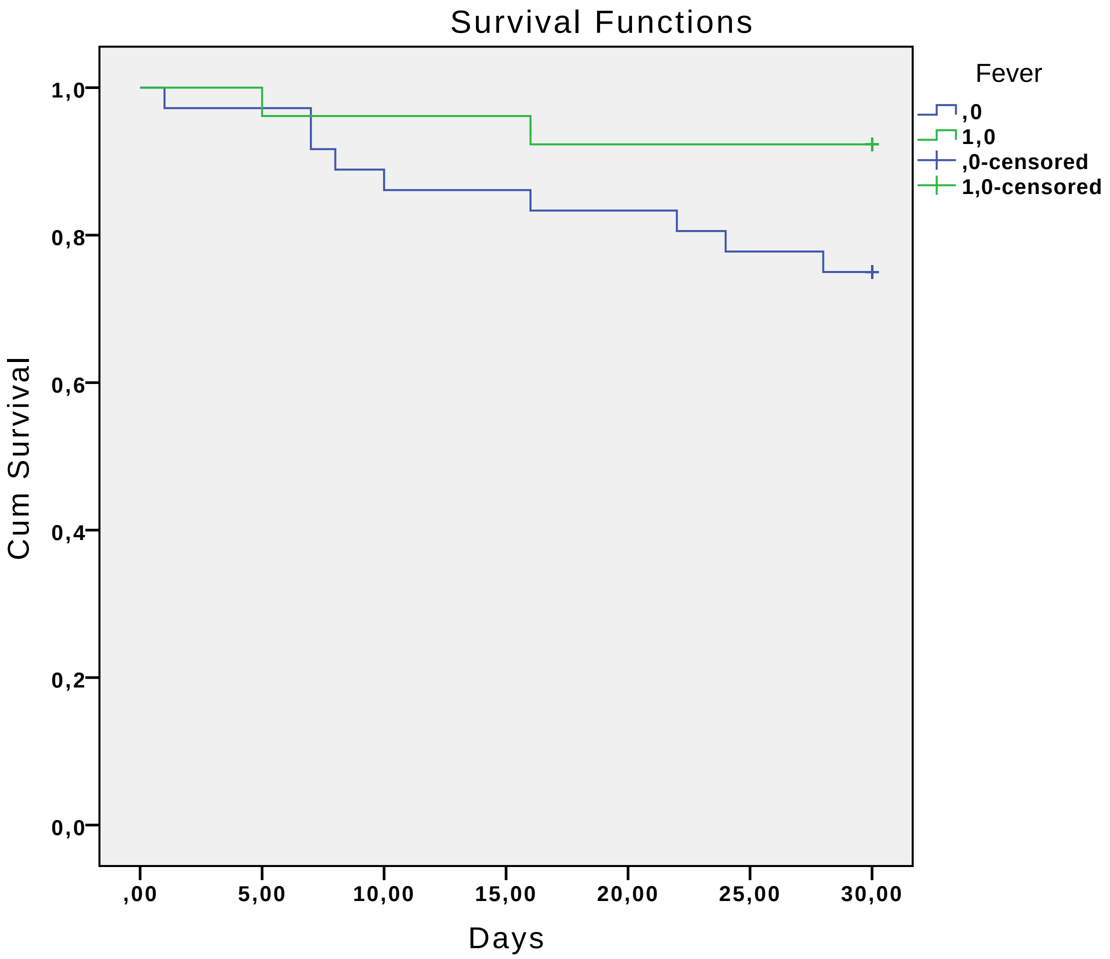

MULTIRESISTANT PSEUDOMONAS (0=NO, 1=YES)

Case Processing Summary

Multirresistant Pseudomonas	Total N	N of Events	Censored	
			N	Percent
,0	44	6	38	86,4%
1,0	13	3	10	76,9%
Overall	57	9	48	84,2%

Survival Table

Multirresistant Pseudomonas		Time	Status	Cumulative Proportion Surviving at the Time		N of Cumulative Events	N of Remaining Cases
				Estimate	Std. Error		
,0	1	5,000	1,00	,977	,022	1	43
	2	7,000	1,00	.	.	2	42
	3	7,000	1,00	,932	,038	3	41
	4	22,000	1,00	,909	,043	4	40
	5	24,000	1,00	,886	,048	5	39
	6	28,000	1,00	,864	,052	6	38
	7	30,000	,00	.	.	6	37
	8	30,000	,00	.	.	6	36
	9	30,000	,00	.	.	6	35
	10	30,000	,00	.	.	6	34
	11	30,000	,00	.	.	6	33
	12	30,000	,00	.	.	6	32
	13	30,000	,00	.	.	6	31
	14	30,000	,00	.	.	6	30
	15	30,000	,00	.	.	6	29
	16	30,000	,00	.	.	6	28
	17	30,000	,00	.	.	6	27
	18	30,000	,00	.	.	6	26
	19	30,000	,00	.	.	6	25
	20	30,000	,00	.	.	6	24
	21	30,000	,00	.	.	6	23
	22	30,000	,00	.	.	6	22
	23	30,000	,00	.	.	6	21
	24	30,000	,00	.	.	6	20
	25	30,000	,00	.	.	6	19
	26	30,000	,00	.	.	6	18
	27	30,000	,00	.	.	6	17
	28	30,000	,00	.	.	6	16
	29	30,000	,00	.	.	6	15
	30	30,000	,00	.	.	6	14
	31	30,000	,00	.	.	6	13
	32	30,000	,00	.	.	6	12
	33	30,000	,00	.	.	6	11
	34	30,000	,00	.	.	6	10
	35	30,000	,00	.	.	6	9
	36	30,000	,00	.	.	6	8
	37	30,000	,00	.	.	6	7
	38	30,000	,00	.	.	6	6
	39	30,000	,00	.	.	6	5
	40	30,000	,00	.	.	6	4
	41	30,000	,00	.	.	6	3
	42	30,000	,00	.	.	6	2
	43	30,000	,00	.	.	6	1
	44	30,000	,00	.	.	6	0
1,0	1	1,000	1,00	,923	,074	1	12
	2	16,000	1,00	.	.	2	11
	3	16,000	1,00	,769	,117	3	10
	4	30,000	,00	.	.	3	9
	5	30,000	,00	.	.	3	8

Survival Table

Multirresistant Pseudomonas	Time	Status	Cumulative Proportion Surviving at the Time		N of Cumulative Events	N of Remaining Cases
			Estimate	Std. Error		
6	30,000	,00	.	.	3	7
7	30,000	,00	.	.	3	6
8	30,000	,00	.	.	3	5
9	30,000	,00	.	.	3	4
10	30,000	,00	.	.	3	3
11	30,000	,00	.	.	3	2
12	30,000	,00	.	.	3	1
13	30,000	,00	.	.	3	0

Means and Medians for Survival Time

Multirresistant Pseudomonas	Mean[a]				Median		
			95% Confidence Interval				95% …
	Estimate	Std. Error	Lower Bound	Upper Bound	Estimate	Std. Error	Lower Bound
,0	28,023	,913	26,233	29,813	.	.	.
1,0	25,615	2,412	20,888	30,343	.	.	.
Overall	27,474	,904	25,701	29,246	.	.	.

Means and Medians for Survival Time

Multirresistant Pseudomonas	Median
	95% …
	Upper Bound
,0	.
1,0	.
Overall	.

a. Estimation is limited to the largest survival time if it is censored.

Overall Comparisons

	Chi-Square	df	Sig.
Log Rank (Mantel-Cox)	,759	1	,384

Test of equality of survival distributions for the different levels of Multirresistant Pseudomonas

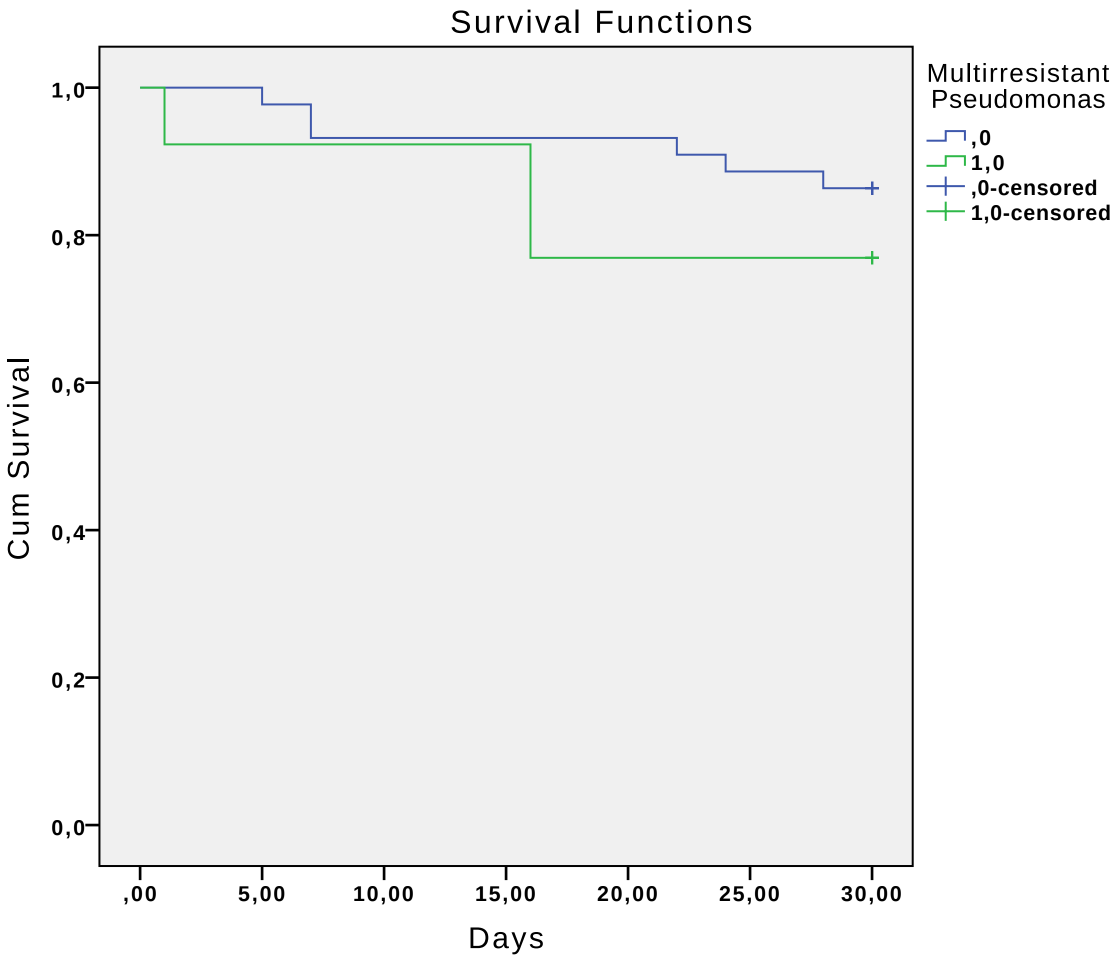

CHARLSON INDEX>2 (0=NO, 1=YES)

Case Processing Summary

Charlson Index>2	Total N	N of Events	Censored	
			N	Percent
,00	23	2	21	91,3%
1,00	37	9	28	75,7%
Overall	60	11	49	81,7%

Survival Table

Charlson Index>2		Time	Status	Cumulative Proportion Surviving at the Time		N of Cumulative Events	N of Remaining Cases
				Estimate	Std. Error		
,00	1	8,000	1,00	,957	,043	1	22
	2	16,000	1,00	,913	,059	2	21
	3	30,000	,00	.	.	2	20
	4	30,000	,00	.	.	2	19
	5	30,000	,00	.	.	2	18
	6	30,000	,00	.	.	2	17
	7	30,000	,00	.	.	2	16
	8	30,000	,00	.	.	2	15
	9	30,000	,00	.	.	2	14
	10	30,000	,00	.	.	2	13
	11	30,000	,00	.	.	2	12
	12	30,000	,00	.	.	2	11
	13	30,000	,00	.	.	2	10
	14	30,000	,00	.	.	2	9
	15	30,000	,00	.	.	2	8
	16	30,000	,00	.	.	2	7
	17	30,000	,00	.	.	2	6
	18	30,000	,00	.	.	2	5
	19	30,000	,00	.	.	2	4
	20	30,000	,00	.	.	2	3
	21	30,000	,00	.	.	2	2
	22	30,000	,00	.	.	2	1
	23	30,000	,00	.	.	2	0
1,00	1	1,000	1,00	,973	,027	1	36
	2	5,000	1,00	,946	,037	2	35
	3	7,000	1,00	.	.	3	34
	4	7,000	1,00	,892	,051	4	33
	5	10,000	1,00	,865	,056	5	32
	6	16,000	1,00	,838	,061	6	31
	7	22,000	1,00	,811	,064	7	30
	8	24,000	1,00	,784	,068	8	29
	9	28,000	1,00	,757	,071	9	28
	10	30,000	,00	.	.	9	27
	11	30,000	,00	.	.	9	26
	12	30,000	,00	.	.	9	25
	13	30,000	,00	.	.	9	24
	14	30,000	,00	.	.	9	23
	15	30,000	,00	.	.	9	22
	16	30,000	,00	.	.	9	21
	17	30,000	,00	.	.	9	20
	18	30,000	,00	.	.	9	19
	19	30,000	,00	.	.	9	18
	20	30,000	,00	.	.	9	17
	21	30,000	,00	.	.	9	16
	22	30,000	,00	.	.	9	15
	23	30,000	,00	.	.	9	14
	24	30,000	,00	.	.	9	13
	25	30,000	,00	.	.	9	12
	26	30,000	,00	.	.	9	11

Survival Table

Charlson Index>2	Time	Status	Cumulative Proportion Surviving at the Time		N of Cumulative Events	N of Remaining Cases
			Estimate	Std. Error		
27	30,000	,00	.	.	9	10
28	30,000	,00	.	.	9	9
29	30,000	,00	.	.	9	8
30	30,000	,00	.	.	9	7
31	30,000	,00	.	.	9	6
32	30,000	,00	.	.	9	5
33	30,000	,00	.	.	9	4
34	30,000	,00	.	.	9	3
35	30,000	,00	.	.	9	2
36	30,000	,00	.	.	9	1
37	30,000	,00	.	.	9	0

Means and Medians for Survival Time

Charlson Index>2	Mean[a]				Median		
			95% Confidence Interval				95% …
	Estimate	Std. Error	Lower Bound	Upper Bound	Estimate	Std. Error	Lower Bound
,00	28,435	1,086	26,307	30,563	.	.	.
1,00	25,946	1,382	23,237	28,655	.	.	.
Overall	26,900	,961	25,016	28,784	.	.	.

Means and Medians for Survival Time

Charlson Index>2	Median
	95% …
	Upper Bound
,00	.
1,00	.
Overall	.

a. Estimation is limited to the largest survival time if it is censored.

Overall Comparisons

	Chi-Square	df	Sig.
Log Rank (Mantel-Cox)	2,243	1	,134

Test of equality of survival distributions for the different levels of Charlson Index>2

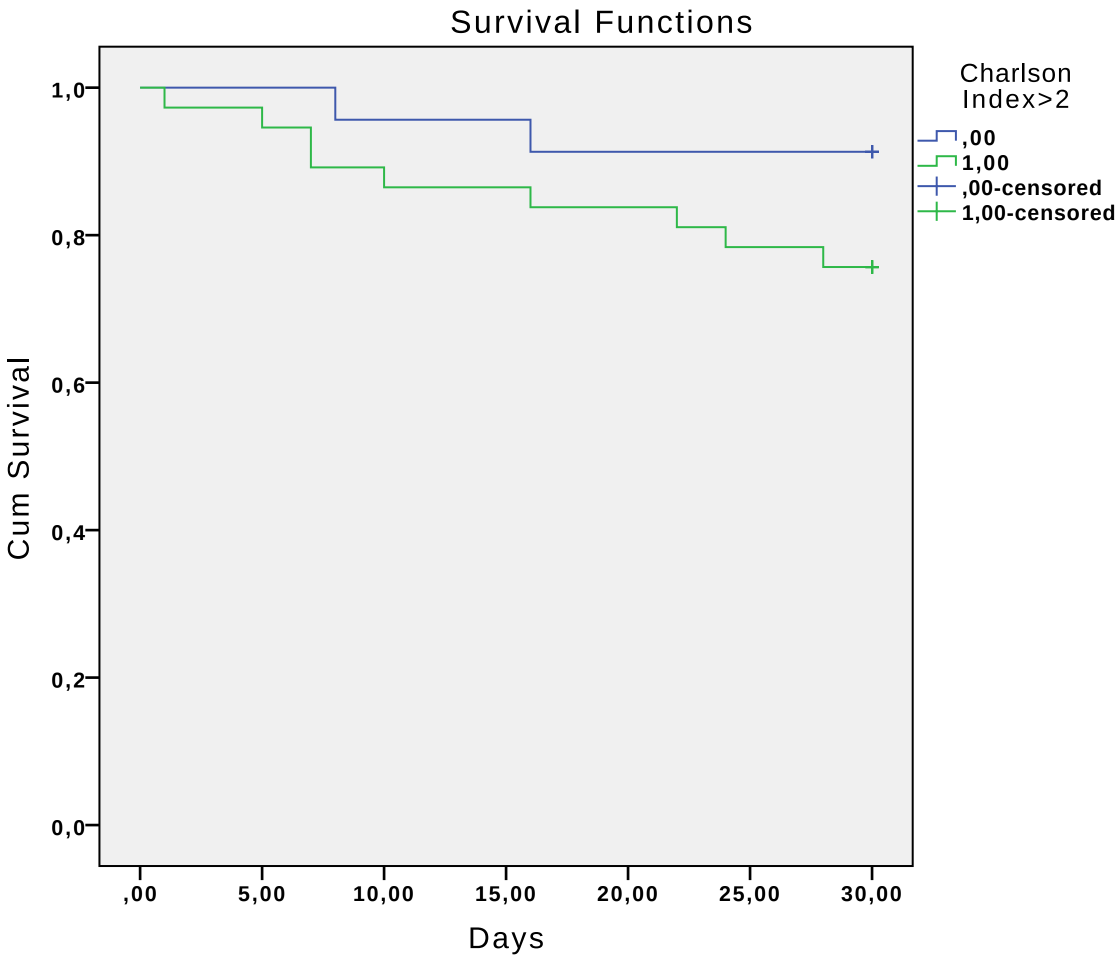

NOSOCOMIAL AND HEALTHCARE ASSOCIATED (0=NO, 1=YES)

Case Processing Summary

Nosocomial and Healthcare Associated	Total N	N of Events	Censored	
			N	Percent
,00	14	1	13	92,9%
1,00	46	9	37	80,4%
Overall	60	10	50	83,3%

Survival Table

Nosocomial and Healthcare Associated		Time	Status	Cumulative Proportion Surviving at the Time		N of Cumulative Events
				Estimate	Std. Error	
,00	1	22,000	1,00	,929	,069	1
	2	30,000	,00	.	.	1
	3	30,000	,00	.	.	1
	4	30,000	,00	.	.	1
	5	30,000	,00	.	.	1
	6	30,000	,00	.	.	1
	7	30,000	,00	.	.	1
	8	30,000	,00	.	.	1
	9	30,000	,00	.	.	1
	10	30,000	,00	.	.	1
	11	30,000	,00	.	.	1
	12	30,000	,00	.	.	1
	13	30,000	,00	.	.	1
	14	30,000	,00	.	.	1
1,00	1	1,000	1,00	,978	,022	1
	2	7,000	1,00	.	.	2
	3	7,000	1,00	,935	,036	3
	4	8,000	1,00	,913	,042	4
	5	10,000	1,00	,891	,046	5
	6	16,000	1,00	.	.	6
	7	16,000	1,00	,848	,053	7
	8	24,000	1,00	,826	,056	8
	9	28,000	1,00	,804	,058	9
	10	30,000	,00	.	.	9
	11	30,000	,00	.	.	9
	12	30,000	,00	.	.	9
	13	30,000	,00	.	.	9
	14	30,000	,00	.	.	9
	15	30,000	,00	.	.	9
	16	30,000	,00	.	.	9
	17	30,000	,00	.	.	9
	18	30,000	,00	.	.	9
	19	30,000	,00	.	.	9
	20	30,000	,00	.	.	9
	21	30,000	,00	.	.	9
	22	30,000	,00	.	.	9
	23	30,000	,00	.	.	9
	24	30,000	,00	.	.	9
	25	30,000	,00	.	.	9
	26	30,000	,00	.	.	9
	27	30,000	,00	.	.	9
	28	30,000	,00	.	.	9
	29	30,000	,00	.	.	9
	30	30,000	,00	.	.	9
	31	30,000	,00	.	.	9
	32	30,000	,00	.	.	9
	33	30,000	,00	.	.	9
	34	30,000	,00	.	.	9
	35	30,000	,00	.	.	9

Survival Table

Nosocomial and Healthcare Associated		N of Remaining Cases
,00	1	13
	2	12
	3	11
	4	10
	5	9
	6	8
	7	7
	8	6
	9	5
	10	4
	11	3
	12	2
	13	1
	14	0
1,00	1	45
	2	44
	3	43
	4	42
	5	41
	6	40
	7	39
	8	38
	9	37
	10	36
	11	35
	12	34
	13	33
	14	32
	15	31
	16	30
	17	29
	18	28
	19	27
	20	26
	21	25
	22	24
	23	23
	24	22
	25	21
	26	20
	27	19
	28	18
	29	17
	30	16
	31	15
	32	14
	33	13
	34	12
	35	11

Survival Table

Nosocomial and Healthcare Associated	Time	Status	Cumulative Proportion Surviving at the Time		N of Cumulative Events
			Estimate	Std. Error	
36	30,000	,00	.	.	9
37	30,000	,00	.	.	9
38	30,000	,00	.	.	9
39	30,000	,00	.	.	9
40	30,000	,00	.	.	9
41	30,000	,00	.	.	9
42	30,000	,00	.	.	9
43	30,000	,00	.	.	9
44	30,000	,00	.	.	9
45	30,000	,00	.	.	9
46	30,000	,00	.	.	9

Survival Table

Nosocomial and Healthcare Associated	N of Remaining Cases
36	10
37	9
38	8
39	7
40	6
41	5
42	4
43	3
44	2
45	1
46	0

Means and Medians for Survival Time

Nosocomial and Healthcare Associated	Mean[a]				Median		
			95% Confidence Interval				95% …
	Estimate	Std. Error	Lower Bound	Upper Bound	Estimate	Std. Error	Lower Bound
,00	29,429	,551	28,349	30,508	.	.	.
1,00	26,674	1,131	24,457	28,890	.	.	.
Overall	27,317	,889	25,574	29,060	.	.	.

Means and Medians for Survival Time

Nosocomial and Healthcare Associated	Median
	95% …
	Upper Bound
,00	.
1,00	.
Overall	.

a. Estimation is limited to the largest survival time if it is censored.

Overall Comparisons

	Chi-Square	df	Sig.
Log Rank (Mantel-Cox)	1,186	1	,276

Test of equality of survival distributions for the different levels of Nosocomial and Healthcare Associated

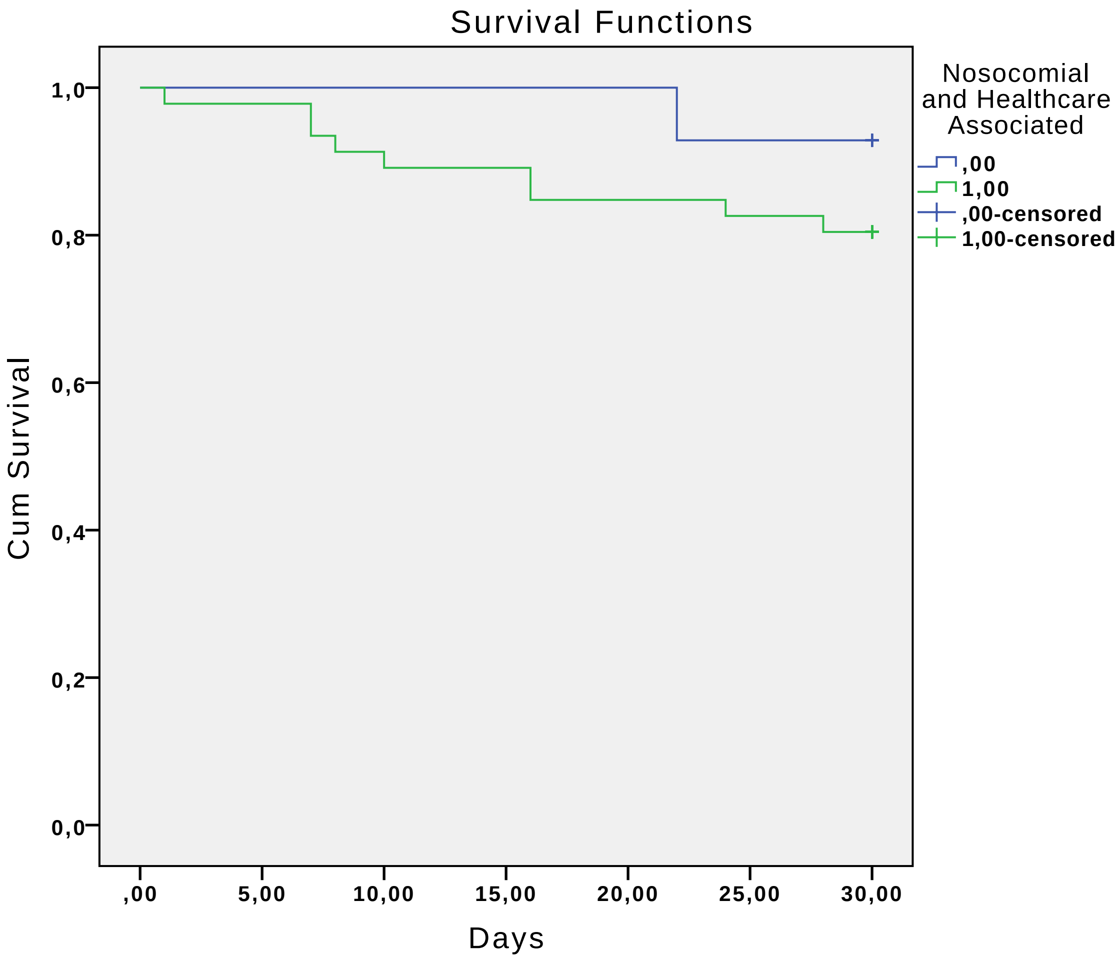

AGE>65 YEARS (0=NO, 1=YES)

Case Processing Summary

Age>65 years	Total N	N of Events	Censored	
			N	Percent
,00	12	1	11	91,7%
1,00	50	10	40	80,0%
Overall	62	11	51	82,3%

Survival Table

Age>65 years		Time	Status	Cumulative Proportion Surviving at the Time		N of Cumulative Events	N of Remaining Cases
				Estimate	Std. Error		
,00	1	5,000	1,00	,917	,080	1	11
	2	30,000	,00	.	.	1	10
	3	30,000	,00	.	.	1	9
	4	30,000	,00	.	.	1	8
	5	30,000	,00	.	.	1	7
	6	30,000	,00	.	.	1	6
	7	30,000	,00	.	.	1	5
	8	30,000	,00	.	.	1	4
	9	30,000	,00	.	.	1	3
	10	30,000	,00	.	.	1	2
	11	30,000	,00	.	.	1	1
	12	30,000	,00	.	.	1	0
1,00	1	1,000	1,00	,980	,020	1	49
	2	7,000	1,00	.	.	2	48
	3	7,000	1,00	,940	,034	3	47
	4	8,000	1,00	,920	,038	4	46
	5	10,000	1,00	,900	,042	5	45
	6	16,000	1,00	.	.	6	44
	7	16,000	1,00	,860	,049	7	43
	8	22,000	1,00	,840	,052	8	42
	9	24,000	1,00	,820	,054	9	41
	10	28,000	1,00	,800	,057	10	40
	11	30,000	,00	.	.	10	39
	12	30,000	,00	.	.	10	38
	13	30,000	,00	.	.	10	37
	14	30,000	,00	.	.	10	36
	15	30,000	,00	.	.	10	35
	16	30,000	,00	.	.	10	34
	17	30,000	,00	.	.	10	33
	18	30,000	,00	.	.	10	32
	19	30,000	,00	.	.	10	31
	20	30,000	,00	.	.	10	30
	21	30,000	,00	.	.	10	29
	22	30,000	,00	.	.	10	28
	23	30,000	,00	.	.	10	27
	24	30,000	,00	.	.	10	26
	25	30,000	,00	.	.	10	25
	26	30,000	,00	.	.	10	24
	27	30,000	,00	.	.	10	23
	28	30,000	,00	.	.	10	22
	29	30,000	,00	.	.	10	21
	30	30,000	,00	.	.	10	20
	31	30,000	,00	.	.	10	19
	32	30,000	,00	.	.	10	18
	33	30,000	,00	.	.	10	17
	34	30,000	,00	.	.	10	16
	35	30,000	,00	.	.	10	15
	36	30,000	,00	.	.	10	14
	37	30,000	,00	.	.	10	13

Survival Table

Age>65 years	Time	Status	Cumulative Proportion Surviving at the Time		N of Cumulative Events	N of Remaining Cases
			Estimate	Std. Error		
38	30,000	,00	.	.	10	12
39	30,000	,00	.	.	10	11
40	30,000	,00	.	.	10	10
41	30,000	,00	.	.	10	9
42	30,000	,00	.	.	10	8
43	30,000	,00	.	.	10	7
44	30,000	,00	.	.	10	6
45	30,000	,00	.	.	10	5
46	30,000	,00	.	.	10	4
47	30,000	,00	.	.	10	3
48	30,000	,00	.	.	10	2
49	30,000	,00	.	.	10	1
50	30,000	,00	.	.	10	0

Means and Medians for Survival Time

Age>65 years	Mean[a]				Median		
			95% Confidence Interval				95% …
	Estimate	Std. Error	Lower Bound	Upper Bound	Estimate	Std. Error	Lower Bound
,00	27,917	1,995	24,007	31,826	.	.	.
1,00	26,780	1,051	24,720	28,840	.	.	.
Overall	27,000	,933	25,171	28,829	.	.	.

Means and Medians for Survival Time

Age>65 years	Median
	95% …
	Upper Bound
,00	.
1,00	.
Overall	.

a. Estimation is limited to the largest survival time if it is censored.

Overall Comparisons

	Chi-Square	df	Sig.
Log Rank (Mantel-Cox)	,775	1	,379

Test of equality of survival distributions for the different levels of Age>65 years

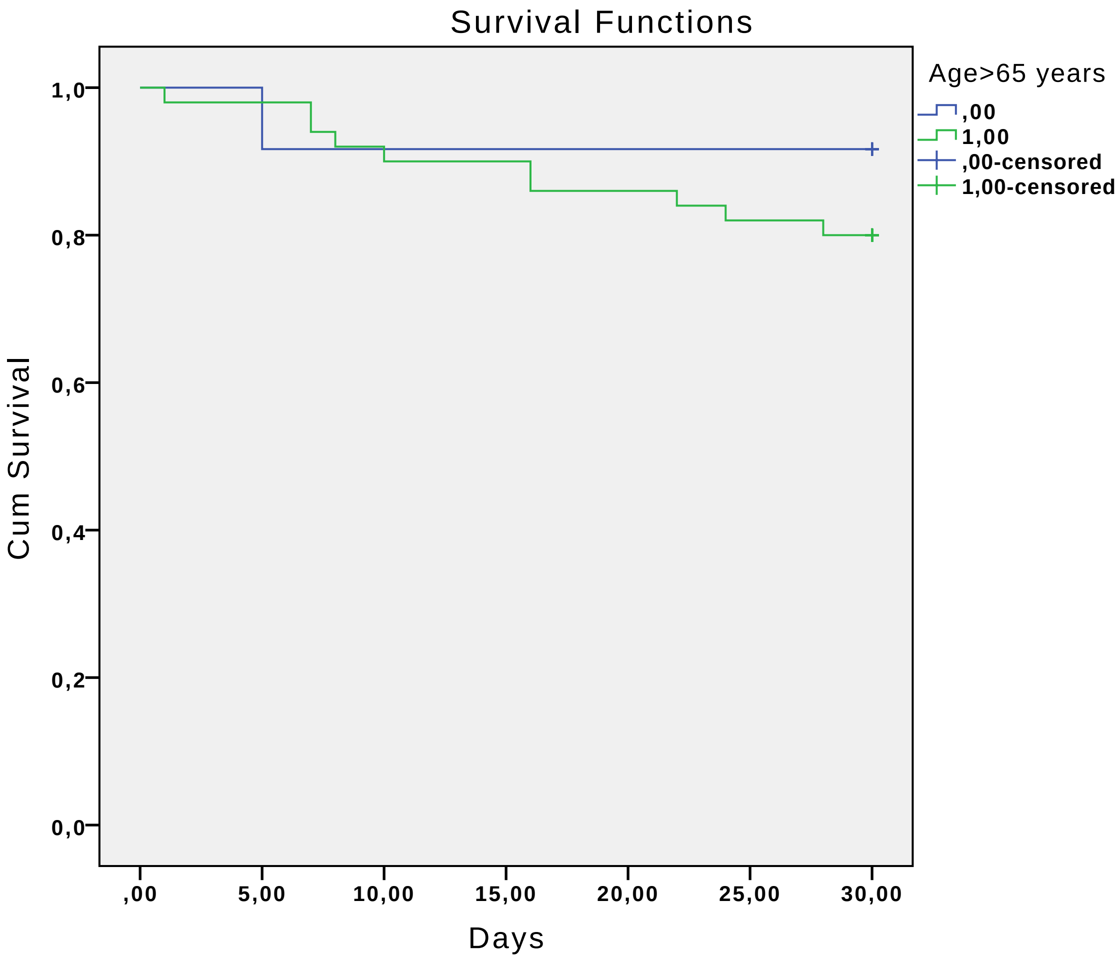

AGE>75 YEARS (0=NO, 1=YES)

Case Processing Summary

Age>75 years	Total N	N of Events	Censored	
			N	Percent
,00	26	2	24	92,3%
1,00	36	9	27	75,0%
Overall	62	11	51	82,3%

Survival Table

Age>75 years		Time	Status	Cumulative Proportion Surviving at the Time		N of Cumulative Events	N of Remaining Cases
				Estimate	Std. Error		
,00	1	5,000	1,00	,962	,038	1	25
	2	10,000	1,00	,923	,052	2	24
	3	30,000	,00	.	.	2	23
	4	30,000	,00	.	.	2	22
	5	30,000	,00	.	.	2	21
	6	30,000	,00	.	.	2	20
	7	30,000	,00	.	.	2	19
	8	30,000	,00	.	.	2	18
	9	30,000	,00	.	.	2	17
	10	30,000	,00	.	.	2	16
	11	30,000	,00	.	.	2	15
	12	30,000	,00	.	.	2	14
	13	30,000	,00	.	.	2	13
	14	30,000	,00	.	.	2	12
	15	30,000	,00	.	.	2	11
	16	30,000	,00	.	.	2	10
	17	30,000	,00	.	.	2	9
	18	30,000	,00	.	.	2	8
	19	30,000	,00	.	.	2	7
	20	30,000	,00	.	.	2	6
	21	30,000	,00	.	.	2	5
	22	30,000	,00	.	.	2	4
	23	30,000	,00	.	.	2	3
	24	30,000	,00	.	.	2	2
	25	30,000	,00	.	.	2	1
	26	30,000	,00	.	.	2	0
1,00	1	1,000	1,00	,972	,027	1	35
	2	7,000	1,00	.	.	2	34
	3	7,000	1,00	,917	,046	3	33
	4	8,000	1,00	,889	,052	4	32
	5	16,000	1,00	.	.	5	31
	6	16,000	1,00	,833	,062	6	30
	7	22,000	1,00	,806	,066	7	29
	8	24,000	1,00	,778	,069	8	28
	9	28,000	1,00	,750	,072	9	27
	10	30,000	,00	.	.	9	26
	11	30,000	,00	.	.	9	25
	12	30,000	,00	.	.	9	24
	13	30,000	,00	.	.	9	23
	14	30,000	,00	.	.	9	22
	15	30,000	,00	.	.	9	21
	16	30,000	,00	.	.	9	20
	17	30,000	,00	.	.	9	19
	18	30,000	,00	.	.	9	18
	19	30,000	,00	.	.	9	17
	20	30,000	,00	.	.	9	16
	21	30,000	,00	.	.	9	15
	22	30,000	,00	.	.	9	14
	23	30,000	,00	.	.	9	13

Survival Table

Age>75 years	Time	Status	Cumulative Proportion Surviving at the Time		N of Cumulative Events	N of Remaining Cases
			Estimate	Std. Error		
24	30,000	,00	.	.	9	12
25	30,000	,00	.	.	9	11
26	30,000	,00	.	.	9	10
27	30,000	,00	.	.	9	9
28	30,000	,00	.	.	9	8
29	30,000	,00	.	.	9	7
30	30,000	,00	.	.	9	6
31	30,000	,00	.	.	9	5
32	30,000	,00	.	.	9	4
33	30,000	,00	.	.	9	3
34	30,000	,00	.	.	9	2
35	30,000	,00	.	.	9	1
36	30,000	,00	.	.	9	0

Means and Medians for Survival Time

Age>75 years	Mean[a]				Median		
			95% Confidence Interval				95% ...
	Estimate	Std. Error	Lower Bound	Upper Bound	Estimate	Std. Error	Lower Bound
,00	28,269	1,184	25,949	30,589	.	.	.
1,00	26,083	1,340	23,457	28,710	.	.	.
Overall	27,000	,933	25,171	28,829	.	.	.

Means and Medians for Survival Time

Age>75 years	Median
	95% ...
	Upper Bound
,00	.
1,00	.
Overall	.

a. Estimation is limited to the largest survival time if it is censored.

Overall Comparisons

	Chi-Square	df	Sig.
Log Rank (Mantel-Cox)	2,850	1	,091

Test of equality of survival distributions for the different levels of Age>75 years

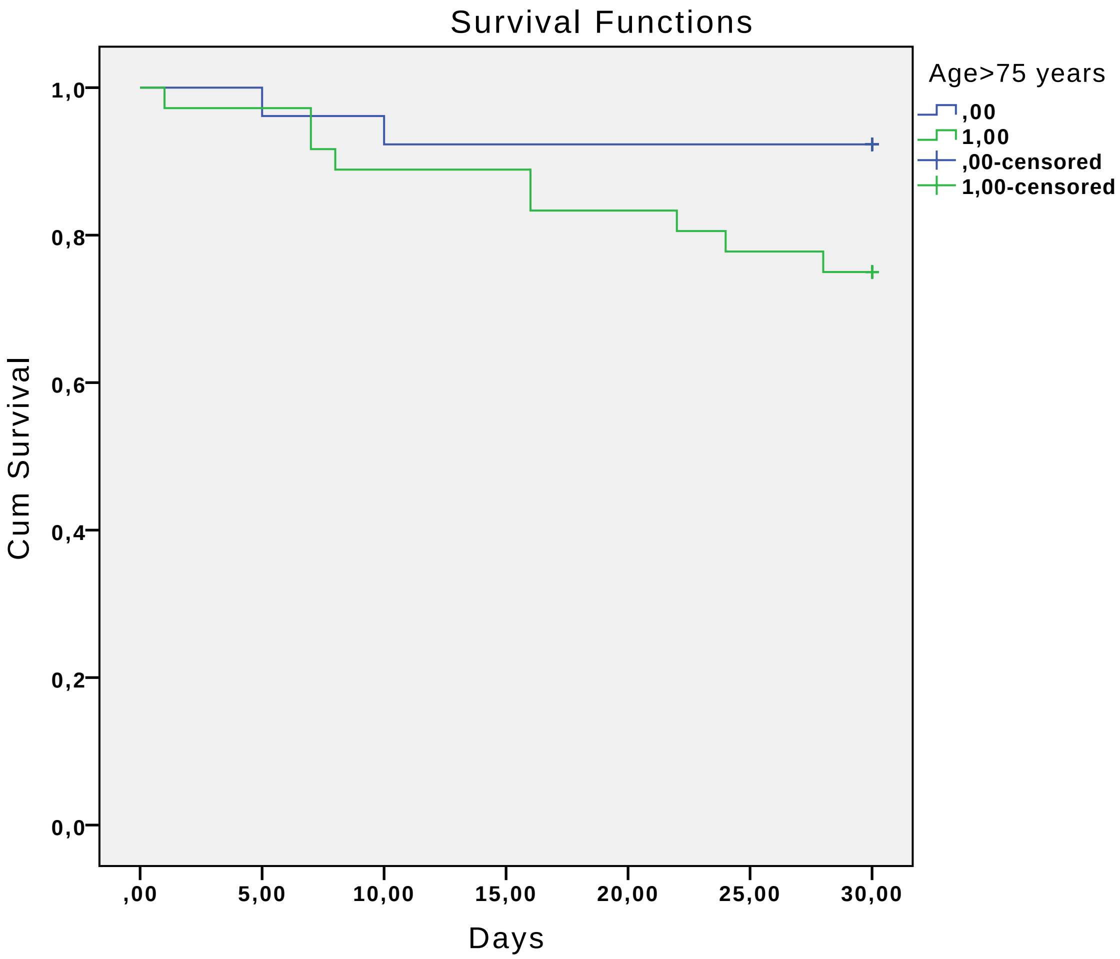

SEBERE SEPSIS/SEPTIC SHOCK (0=NO, 1=YES)

Case Processing Summary

Severe Sepsis/Septic Shock	Total N	N of Events	Censored	
			N	Percent
,00	57	8	49	86,0%
1,00	5	3	2	40,0%
Overall	62	11	51	82,3%

Survival Table

Severe Sepsis/Septic Shock		Time	Status	Cumulative Proportion Surviving at the Time		N of Cumulative Events	N of Remaining Cases
				Estimate	Std. Error		
,00	1	1,000	1,00	,982	,017	1	56
	2	7,000	1,00	.	.	2	55
	3	7,000	1,00	,947	,030	3	54
	4	16,000	1,00	.	.	4	53
	5	16,000	1,00	,912	,037	5	52
	6	22,000	1,00	,895	,041	6	51
	7	24,000	1,00	,877	,043	7	50
	8	28,000	1,00	,860	,046	8	49
	9	30,000	,00	.	.	8	48
	10	30,000	,00	.	.	8	47
	11	30,000	,00	.	.	8	46
	12	30,000	,00	.	.	8	45
	13	30,000	,00	.	.	8	44
	14	30,000	,00	.	.	8	43
	15	30,000	,00	.	.	8	42
	16	30,000	,00	.	.	8	41
	17	30,000	,00	.	.	8	40
	18	30,000	,00	.	.	8	39
	19	30,000	,00	.	.	8	38
	20	30,000	,00	.	.	8	37
	21	30,000	,00	.	.	8	36
	22	30,000	,00	.	.	8	35
	23	30,000	,00	.	.	8	34
	24	30,000	,00	.	.	8	33
	25	30,000	,00	.	.	8	32
	26	30,000	,00	.	.	8	31
	27	30,000	,00	.	.	8	30
	28	30,000	,00	.	.	8	29
	29	30,000	,00	.	.	8	28
	30	30,000	,00	.	.	8	27
	31	30,000	,00	.	.	8	26
	32	30,000	,00	.	.	8	25
	33	30,000	,00	.	.	8	24
	34	30,000	,00	.	.	8	23
	35	30,000	,00	.	.	8	22
	36	30,000	,00	.	.	8	21
	37	30,000	,00	.	.	8	20
	38	30,000	,00	.	.	8	19
	39	30,000	,00	.	.	8	18
	40	30,000	,00	.	.	8	17
	41	30,000	,00	.	.	8	16
	42	30,000	,00	.	.	8	15
	43	30,000	,00	.	.	8	14
	44	30,000	,00	.	.	8	13
	45	30,000	,00	.	.	8	12
	46	30,000	,00	.	.	8	11
	47	30,000	,00	.	.	8	10
	48	30,000	,00	.	.	8	9
	49	30,000	,00	.	.	8	8

Survival Table

Severe Sepsis/Septic Shock		Time	Status	Cumulative Proportion Surviving at the Time		N of Cumulative Events	N of Remaining Cases
				Estimate	Std. Error		
	50	30,000	,00	.	.	8	7
	51	30,000	,00	.	.	8	6
	52	30,000	,00	.	.	8	5
	53	30,000	,00	.	.	8	4
	54	30,000	,00	.	.	8	3
	55	30,000	,00	.	.	8	2
	56	30,000	,00	.	.	8	1
	57	30,000	,00	.	.	8	0
1,00	1	5,000	1,00	,800	,179	1	4
	2	8,000	1,00	,600	,219	2	3
	3	10,000	1,00	,400	,219	3	2
	4	30,000	,00	.	.	3	1
	5	30,000	,00	.	.	3	0

Means and Medians for Survival Time

Severe Sepsis/Septic Shock	Mean[a]				Median		
			95% Confidence Interval				95% …
	Estimate	Std. Error	Lower Bound	Upper Bound	Estimate	Std. Error	Lower Bound
,00	27,912	,813	26,319	29,505	.	.	.
1,00	16,600	4,944	6,909	26,291	10,000	2,191	5,706
Overall	27,000	,933	25,171	28,829	.	.	.

Means and Medians for Survival Time

Severe Sepsis/Septic Shock	Median
	95% …
	Upper Bound
,00	.
1,00	14,294
Overall	.

a. Estimation is limited to the largest survival time if it is censored.

Overall Comparisons

	Chi-Square	df	Sig.
Log Rank (Mantel-Cox)	10,223	1	,001

Test of equality of survival distributions for the different levels of Severe Sepsis/Septic Shock

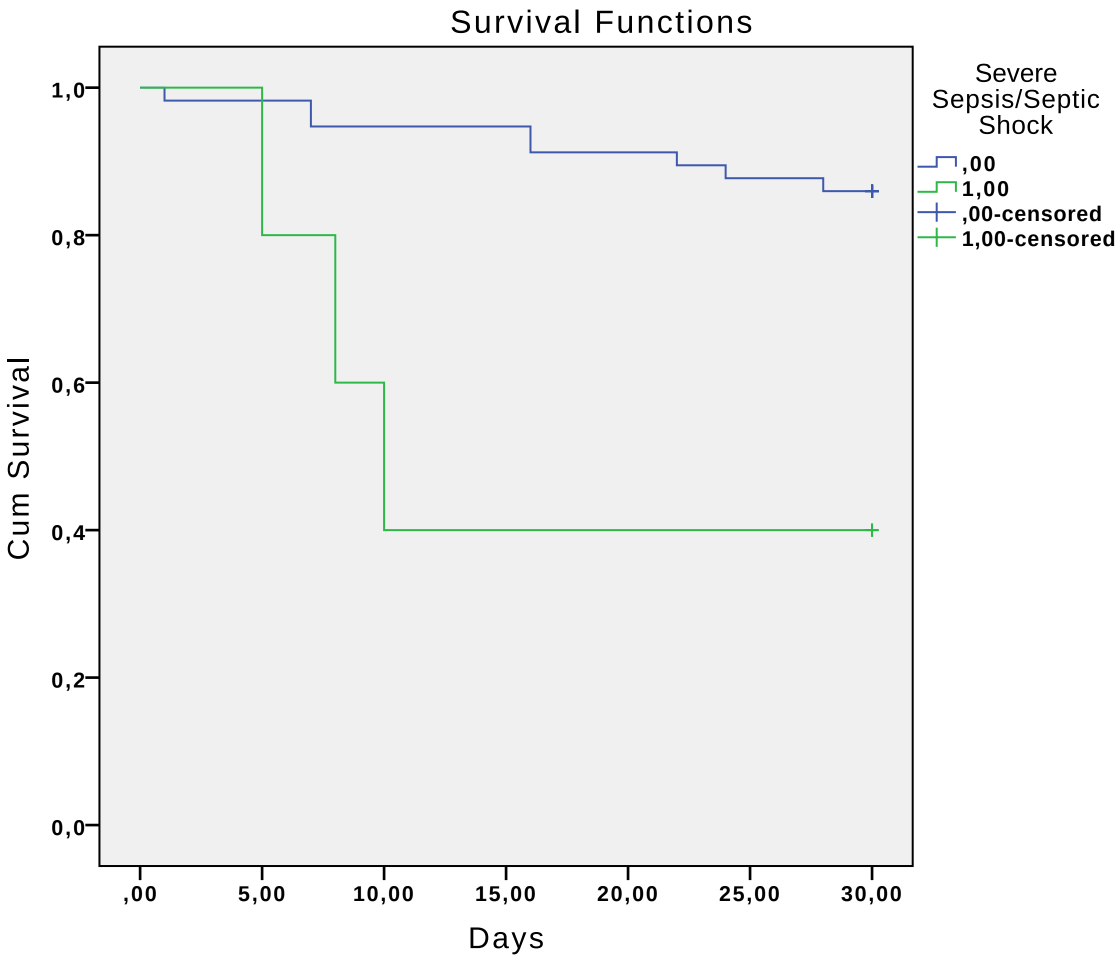

CHARLSON INDEX>3 (0=NO, 1=YES)

Case Processing Summary

Charlson Index>3	Total N	N of Events	Censored	
			N	Percent
,00	30	2	28	93,3%
1,00	30	9	21	70,0%
Overall	60	11	49	81,7%

Survival Table

Charlson Index>3		Time	Status	Cumulative Proportion Surviving at the Time		N of Cumulative Events	N of Remaining Cases
				Estimate	Std. Error		
,00	1	8,000	1,00	,967	,033	1	29
	2	16,000	1,00	,933	,046	2	28
	3	30,000	,00	.	.	2	27
	4	30,000	,00	.	.	2	26
	5	30,000	,00	.	.	2	25
	6	30,000	,00	.	.	2	24
	7	30,000	,00	.	.	2	23
	8	30,000	,00	.	.	2	22
	9	30,000	,00	.	.	2	21
	10	30,000	,00	.	.	2	20
	11	30,000	,00	.	.	2	19
	12	30,000	,00	.	.	2	18
	13	30,000	,00	.	.	2	17
	14	30,000	,00	.	.	2	16
	15	30,000	,00	.	.	2	15
	16	30,000	,00	.	.	2	14
	17	30,000	,00	.	.	2	13
	18	30,000	,00	.	.	2	12
	19	30,000	,00	.	.	2	11
	20	30,000	,00	.	.	2	10
	21	30,000	,00	.	.	2	9
	22	30,000	,00	.	.	2	8
	23	30,000	,00	.	.	2	7
	24	30,000	,00	.	.	2	6
	25	30,000	,00	.	.	2	5
	26	30,000	,00	.	.	2	4
	27	30,000	,00	.	.	2	3
	28	30,000	,00	.	.	2	2
	29	30,000	,00	.	.	2	1
	30	30,000	,00	.	.	2	0
1,00	1	1,000	1,00	,967	,033	1	29
	2	5,000	1,00	,933	,046	2	28
	3	7,000	1,00	.	.	3	27
	4	7,000	1,00	,867	,062	4	26
	5	10,000	1,00	,833	,068	5	25
	6	16,000	1,00	,800	,073	6	24
	7	22,000	1,00	,767	,077	7	23
	8	24,000	1,00	,733	,081	8	22
	9	28,000	1,00	,700	,084	9	21
	10	30,000	,00	.	.	9	20
	11	30,000	,00	.	.	9	19
	12	30,000	,00	.	.	9	18
	13	30,000	,00	.	.	9	17
	14	30,000	,00	.	.	9	16
	15	30,000	,00	.	.	9	15
	16	30,000	,00	.	.	9	14
	17	30,000	,00	.	.	9	13
	18	30,000	,00	.	.	9	12
	19	30,000	,00	.	.	9	11

Survival Table

Charlson Index>3	Time	Status	Cumulative Proportion Surviving at the Time		N of Cumulative Events	N of Remaining Cases
			Estimate	Std. Error		
20	30,000	,00	.	.	9	10
21	30,000	,00	.	.	9	9
22	30,000	,00	.	.	9	8
23	30,000	,00	.	.	9	7
24	30,000	,00	.	.	9	6
25	30,000	,00	.	.	9	5
26	30,000	,00	.	.	9	4
27	30,000	,00	.	.	9	3
28	30,000	,00	.	.	9	2
29	30,000	,00	.	.	9	1
30	30,000	,00	.	.	9	0

Means and Medians for Survival Time

Charlson Index>3	Mean[a]				Median		
			95% Confidence Interval				95% …
	Estimate	Std. Error	Lower Bound	Upper Bound	Estimate	Std. Error	Lower Bound
,00	28,800	,841	27,151	30,449	.	.	.
1,00	25,000	1,658	21,750	28,250	.	.	.
Overall	26,900	,961	25,016	28,784	.	.	.

Means and Medians for Survival Time

Charlson Index>3	Median
	95% …
	Upper Bound
,00	.
1,00	.
Overall	.

a. Estimation is limited to the largest survival time if it is censored.

Overall Comparisons

	Chi-Square	df	Sig.
Log Rank (Mantel-Cox)	5,388	1	,020

Test of equality of survival distributions for the different levels of Charlson Index>3

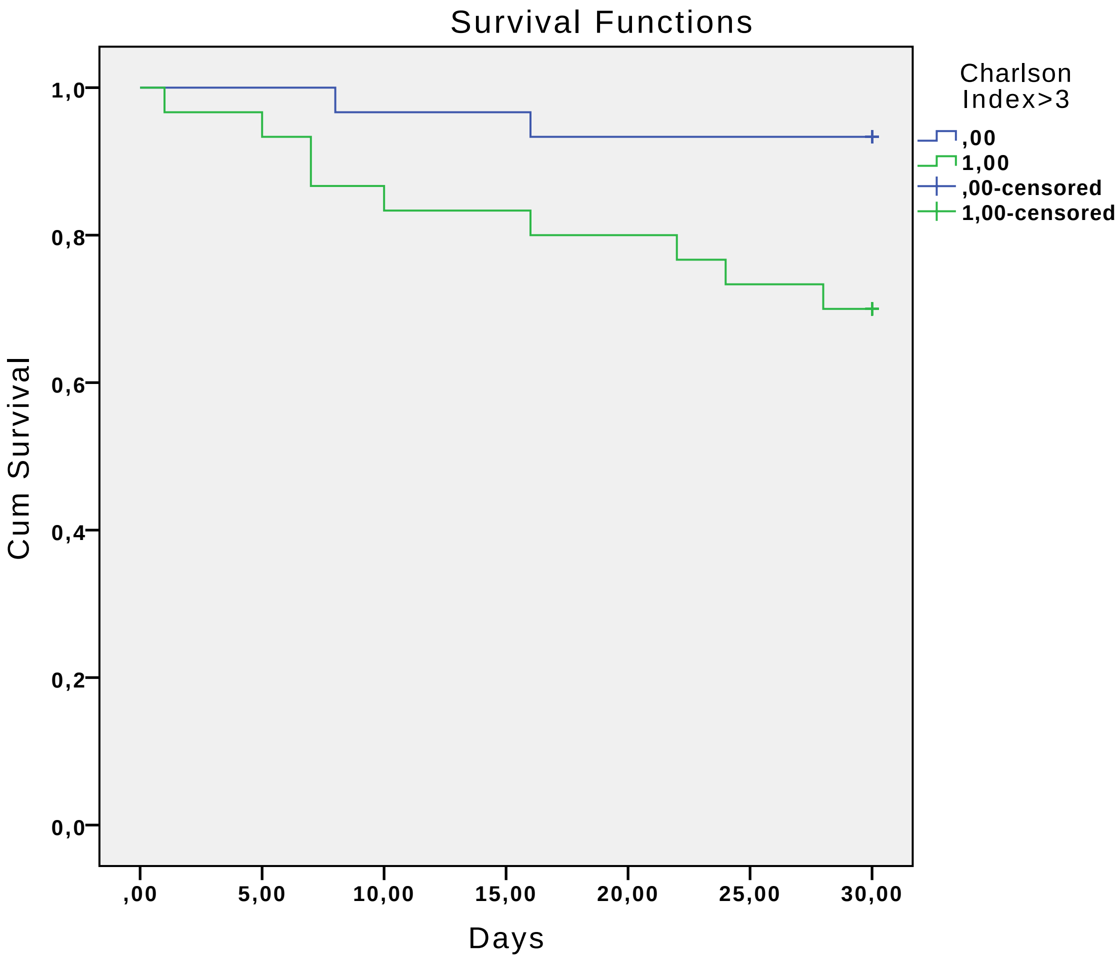

INADEQUATED DEFINITIVE ANTIBIOTIC TREATMENT (0=NO, 1=YES)

Case Processing Summary

Inadequated Definitive Antibiotic Treatment	Total N	N of Events	Censored	
			N	Percent
,0	45	5	40	88,9%
1,0	15	6	9	60,0%
Overall	60	11	49	81,7%

Survival Table

Inadequated Definitive Antibiotic Treatment		Time	Status	Cumulative Proportion Surviving at the Time		N of Cumulative Events
				Estimate	Std. Error	
,0	1	7,000	1,00	,978	,022	1
	2	10,000	1,00	,956	,031	2
	3	16,000	1,00	,933	,037	3
	4	22,000	1,00	,911	,042	4
	5	24,000	1,00	,889	,047	5
	6	30,000	,00	.	.	5
	7	30,000	,00	.	.	5
	8	30,000	,00	.	.	5
	9	30,000	,00	.	.	5
	10	30,000	,00	.	.	5
	11	30,000	,00	.	.	5
	12	30,000	,00	.	.	5
	13	30,000	,00	.	.	5
	14	30,000	,00	.	.	5
	15	30,000	,00	.	.	5
	16	30,000	,00	.	.	5
	17	30,000	,00	.	.	5
	18	30,000	,00	.	.	5
	19	30,000	,00	.	.	5
	20	30,000	,00	.	.	5
	21	30,000	,00	.	.	5
	22	30,000	,00	.	.	5
	23	30,000	,00	.	.	5
	24	30,000	,00	.	.	5
	25	30,000	,00	.	.	5
	26	30,000	,00	.	.	5
	27	30,000	,00	.	.	5
	28	30,000	,00	.	.	5
	29	30,000	,00	.	.	5
	30	30,000	,00	.	.	5
	31	30,000	,00	.	.	5
	32	30,000	,00	.	.	5
	33	30,000	,00	.	.	5
	34	30,000	,00	.	.	5
	35	30,000	,00	.	.	5
	36	30,000	,00	.	.	5
	37	30,000	,00	.	.	5
	38	30,000	,00	.	.	5
	39	30,000	,00	.	.	5
	40	30,000	,00	.	.	5
	41	30,000	,00	.	.	5
	42	30,000	,00	.	.	5
	43	30,000	,00	.	.	5
	44	30,000	,00	.	.	5
	45	30,000	,00	.	.	5
1,0	1	1,000	1,00	,933	,064	1
	2	5,000	1,00	,867	,088	2
	3	7,000	1,00	,800	,103	3
	4	8,000	1,00	,733	,114	4

Survival Table

Inadequated Definitive Antibiotic Treatment		N of Remaining Cases
,0	1	44
	2	43
	3	42
	4	41
	5	40
	6	39
	7	38
	8	37
	9	36
	10	35
	11	34
	12	33
	13	32
	14	31
	15	30
	16	29
	17	28
	18	27
	19	26
	20	25
	21	24
	22	23
	23	22
	24	21
	25	20
	26	19
	27	18
	28	17
	29	16
	30	15
	31	14
	32	13
	33	12
	34	11
	35	10
	36	9
	37	8
	38	7
	39	6
	40	5
	41	4
	42	3
	43	2
	44	1
	45	0
1,0	1	14
	2	13
	3	12
	4	11

Survival Table

Inadequated Definitive Antibiotic Treatment	Time	Status	Cumulative Proportion Surviving at the Time		N of Cumulative Events
			Estimate	Std. Error	
5	16,000	1,00	,667	,122	5
6	28,000	1,00	,600	,126	6
7	30,000	,00	.	.	6
8	30,000	,00	.	.	6
9	30,000	,00	.	.	6
10	30,000	,00	.	.	6
11	30,000	,00	.	.	6
12	30,000	,00	.	.	6
13	30,000	,00	.	.	6
14	30,000	,00	.	.	6
15	30,000	,00	.	.	6

Survival Table

Inadequated Definitive Antibiotic Treatment	N of Remaining Cases
5	10
6	9
7	8
8	7
9	6
10	5
11	4
12	3
13	2
14	1
15	0

Means and Medians for Survival Time

Inadequated Definitive Antibiotic Treatment	Mean[a]				Median		
			95% Confidence Interval				95% …
	Estimate	Std. Error	Lower Bound	Upper Bound	Estimate	Std. Error	Lower Bound
,0	28,422	,741	26,969	29,875	.	.	.
1,0	22,333	2,826	16,794	27,873	.	.	.
Overall	26,900	,961	25,016	28,784	.	.	.

Means and Medians for Survival Time

Inadequated Definitive Antibiotic Treatment	Median
	95% …
	Upper Bound
,0	.
1,0	.
Overall	.

a. Estimation is limited to the largest survival time if it is censored.

Overall Comparisons

	Chi-Square	df	Sig.
Log Rank (Mantel-Cox)	7,298	1	,007

Test of equality of survival distributions for the different levels of Inadequated Definitive Antibiotic Treatment

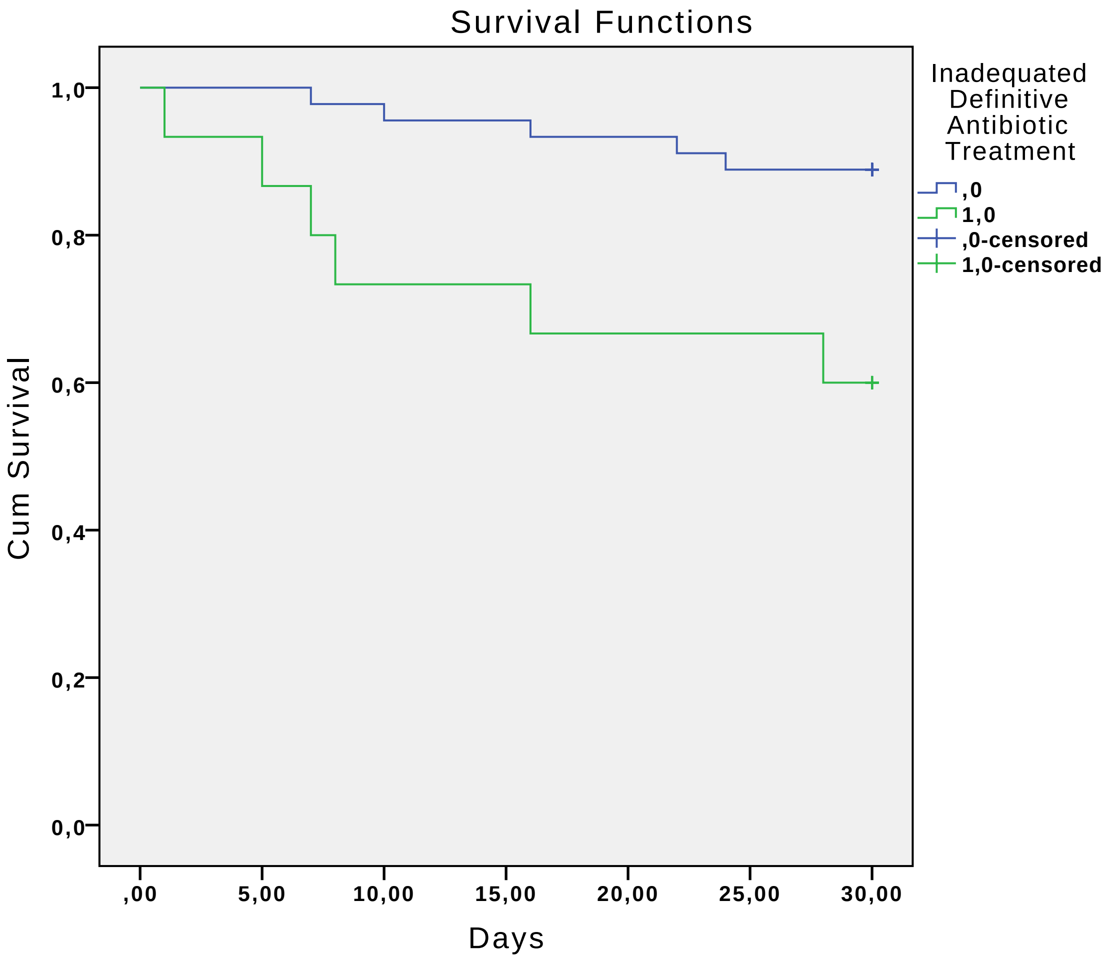

Supplement: S3 File — (PDF) [file pone.0178178.s003.pdf]
